# Chemodivergent Synthesis of 1,4-Benzo[*b*]dithiins and 1,4-Benzodithiafulvenes

**DOI:** 10.1021/acs.orglett.5c05364

**Published:** 2026-01-27

**Authors:** Douglas B. Paixão, Anita B. Kessler, Fabiano S. Rodembusch, Rafael Stieler, Daniel S. Rampon, Paulo H. Schneider

**Affiliations:** † Instituto de Química, Departamento de Química Orgânica, Universidade Federal do Rio Grande do Sul (UFRGS), Post Office Box 15003, Porto Alegre, Rio Grande do Sul 91501-970, Brazil; ‡ Laboratório de Polímeros e Catálise (LAPOCA), Departamento de Química, Universidade Federal do Paraná (UFPR), Post Office Box 19061, Curitiba, Paraná 81531-990, Brazil

## Abstract

We report a chemodivergent synthesis of 1,4-benzo­[*b*]­dithiins and 1,4-benzodithiafulvenes from 2-iodoaryl alkynyl sulfides using a NaSH·*x*H_2_O/KOH system. In DMF, the reaction affords six-membered 1,4-dithiins, whereas in DMSO, these intermediates undergo a base-promoted ring contraction to the corresponding 1,4-dithiafulvenes. Photophysical and mass spectrometry studies support the idea that this chemodivergence arises from the interplay between S^2–^/S_3_^•^ ^–^ equilibrium and base availability in each solvent, which governs whether the 1,4-dithiin framework is preserved or undergoes ring contraction to 1,4-benzodithiafulvenes.

## Graphical abstract

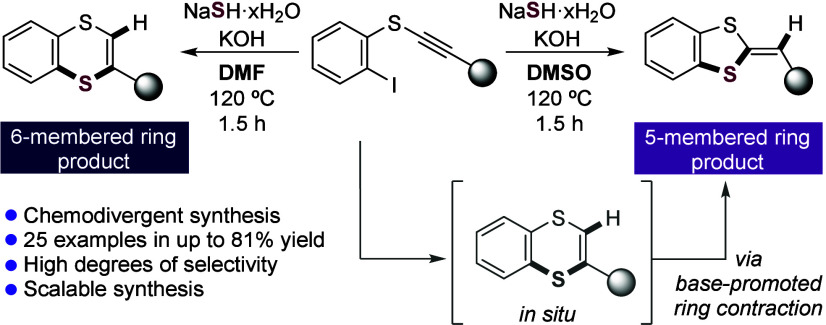

1,4-Dithiins are six-membered, sulfur-rich heterocycles that have emerged as key building blocks in organic materials due to their reversible redox properties and structural flexibility.^1^ Aromatic-fused 1,4-dithiins can form radical cations and undergo switchable conformational changes, making them valuable for applications in photocatalysis,^2^ supramolecular and host–guest chemistry,^3^ chiroptical systems,^4^ and redox-active materials.^5^ Similarly, 1,4-dithiafulvenes (DTFs), structural isomers of 1,4-dithiins composed of a five-membered 1,3-dithiole ring and an exocyclic C=C bond (Scheme 1A), are recognized as “half units” of tetrathiafulvalenes (TTFs),^6^ combining electron-donating and -withdrawing properties driven by zwitterionic resonance.^7^ Their incorporation into π-extended systems or oxidative dimerization into tetrathiafulvalene vinylogues (TTFVs) has enabled conjugated organic materials,^8^ switchable processes,^9^ and chemosensors.^10^

**Scheme 1:**
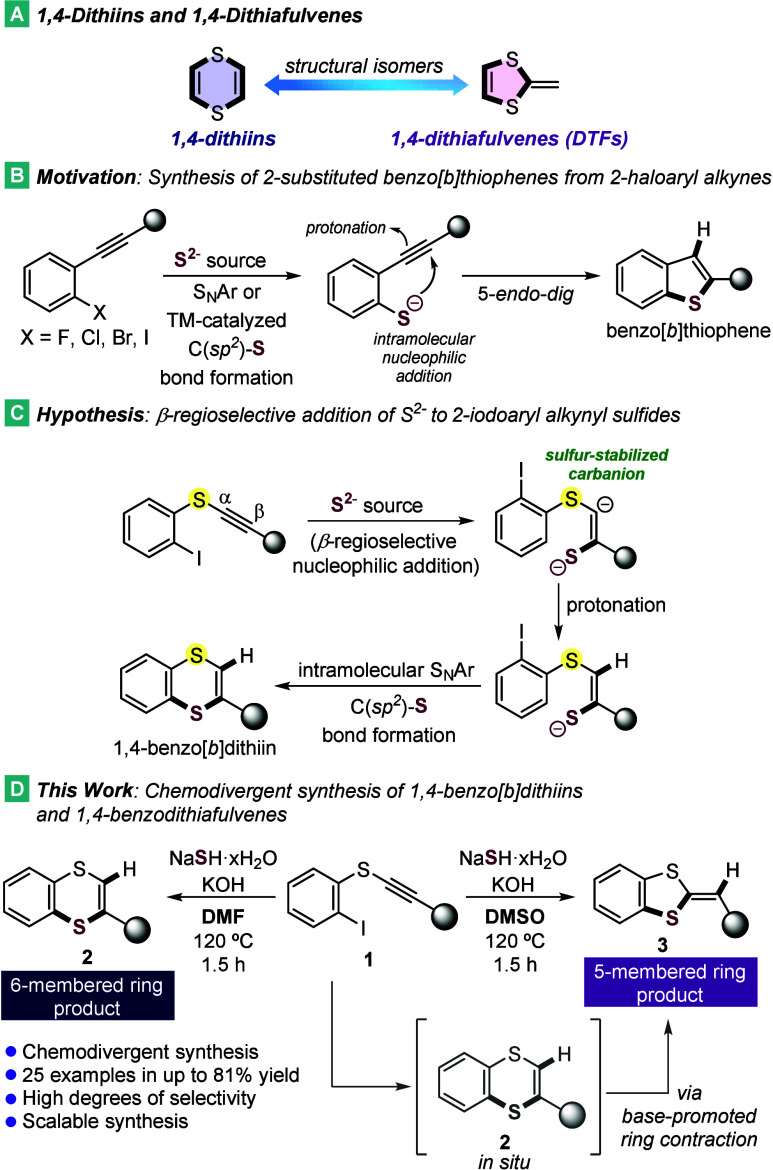
Previous Reports and Our Reaction Design

The synthesis of benzo-fused analogues of these sulfur-rich heterocycles remains limited and challenging. Early examples of 1,4-benzo­[*b*]­dithiins involved the reaction of 1,8-diketones with Lawesson’s reagent, ring expansion of 1,3-dithiolanes, or the use of *in situ* generated benzodithiete with alkynes.^11^ More recently, Miyake and co-workers employed ethynylbenziodoxolone (EBX) reagents and thiols to access three 1,4-benzo­[*b*]­dithiin derivatives,^12^ while Xie’s group reported a Cu-catalyzed diarylthiolation of ynones.^13^ In contrast, 1,4-benzodithiafulvenes are predominantly synthesized by carbonyl olefination,^14^ with only a few reports involving dehalogenation reactions^15^ or base-promoted ring contraction of 1,4-benzodithiins.^16^

This led us to hypothesize that the unique reactivity of alkynyl sulfides^17^ could provide an efficient platform for synthesizing 1,4-benzo­[*b*]­dithiins. Inspired by the well-established synthesis of benzo­[*b*]­thiophenes from 2-haloaryl alkynes and S^2–^ sources (Scheme 1B),^18^ we considered 2-iodoaryl alkynyl sulfides as suitable substrates for a mechanistically “inverse” pathway (Scheme 1C). The iodine substituent was designed to prevent undesired S_N_Ar-type initiation, commonly associated with cyclizations of 2-bromo-,^19^ 2-chloro-,^20^ or 2-fluoroaryl alkynes^21^ in the synthesis of benzo­[*b*]­thiophenes under transition-metal-free conditions. Thus, we proposed to proceed via a chemo- and β-regioselective addition of S^2–^ species to the C≡C triple bond, generating a vinylic carbanion intermediate potentially stabilized by the adjacent sulfur atom.^22^ Subsequent protonation, followed by an intramolecular S_N_Ar reaction, afforded the six-membered 1,4-dithiin ring.

During our studies on the synthesis of six-membered 1,4-benzo­[*b*]­dithiins (**2**) from 2-iodoaryl alkynyl sulfides (**1**) using a NaSH·*x*H_2_O/KOH system in DMSO, we consistently observed the formation of the five-membered product, 1,4-benzodithiafulvenes (**3**). This outcome prompted us to explore whether the formation of either **2** or **3** could be controlled by adjusting the reaction conditions. Notably, complete chemodivergence was achieved by changing the solvent from DMSO to DMF, motivating a detailed investigation of the origin of this selectivity (Scheme 1D).

Despite multiple reactive sites, 2-Iodoaryl alkynyl sulfides (**1**) have been explored almost exclusively as precursors to substrates used in cycloaddition reactions.^23^ The reported syntheses of these compounds are usually limited in scope and require harsh conditions; therefore, we adopted an alternative strategy via oxidative C­(sp)–S coupling of 2-aminophenyl disulfides with terminal alkynes, followed by diazotization/iodination (see section 2 of the Supporting Information for details).^24^ Our optimization studies started with NaSH·*x*H_2_O (1.25 equiv),^25^ KOH (2.0 equiv), and **1a** (1.0 equiv) as model substrates (Table 1). The five-membered product **3a** was obtained in 78% yield after 1.5 h at 120 °C in DMSO, with only negligible amounts of six-membered product **2a** detected by GC–MS (Table 1, entry 1). Using NaOH or higher amounts of KOH (3.0 equiv) maintained high selectivity for **3a** without yield improvement (Table 1, entries 2 and 3). Interestingly, lower amounts of KOH (0.5 equiv) shifted from 1:99 to 89:11 (**2a**/**3a**), and **2a** was isolated in 43% yield (Table 1, entry 4). In the absence of a base, complete selectivity toward **2a** was observed, which was obtained in 40% yield (Table 1, entry 5). These results suggest that the reaction initially forms the six-membered product **2a**, which subsequently undergoes a base-promoted ring contraction to afford the five-membered product **3a**.^16^ They also demonstrate that the base plays a crucial role not only in promoting ring contraction but also in enhancing the overall reaction efficiency. To improve the yield of **2a** while suppressing ring contraction, milder bases were evaluated (Table 1, entries 6–8). Among them, Cs_2_CO_3_ (1.0 equiv) provided an optimal balance of reactivity and selectivity, fully inhibiting ring contraction and affording **2a** in 62% yield (Table 1, entry 8). Alternative sulfur sources, including K_2_S_*x*_,^26^ EtOCS_2_K,^27^ and S,^28^ or increased amounts of NaSH·*x*H_2_O, selectively afforded **3a** but without yield improvement (Table 1, entries 9–12). Lowering the temperature to 80 °C reduced both efficiency and selectivity (48:52, **2a**/**3a**), indicating a strong temperature dependence of the ring contraction (Table 1, entry 13). Remarkably, performing the reaction in DMF with the same base loading employed in DMSO almost completely suppressed the formation of **3a**, affording **2a** in 76% yield with 99:1 selectivity (**2a**/**3a**) (Table 1, entry 14).

**Table 1:**
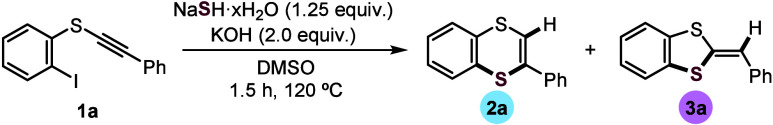
Optimization Studies^a^

entry	variation from initial conditions	**2a**/**3a** ^b^	yield of **2a** (%)^c^	yield of **3a** (%)^c^
**1**	**none**	**1:99**	**trace**	**78**
2	NaOH as the base	1:99	trace	70
3	3.0 equiv of KOH	1:99	trace	78
4	0.5 equiv of KOH	89:11	43	9
5	no base	100:0	40	
6	K_2_CO_3_ as the base	100:0	44	
7	Cs_2_CO_3_ as the base	85:15	53	13
8	1.0 equiv of Cs_2_CO_3_ as the base	100:0	62	
9	K_2_S_*x*_ as the sulfur source	5:95	trace	52
10	EtOCS_2_K as the sulfur source	1:99		26
11	S^0^ as the sulfur source	1:99		25
12	2.0 equiv of NaSH·*x*H_2_O and 4.0 equiv of KOH	1:99	trace	55
13	80 °C as the temperature	48:52	26	30
**14**	**DMF as the solvent**	**99:1**	**76**	**trace**

a Initial conditions: **1a** (0.2 mmol, 1.0 equiv), NaSH·*x*H_2_O (0.25 mmol, 1.25 equiv), KOH (0.4 mmol, 2.0 equiv), and DMSO (1 mL) at 120 °C for 1.5 h under an argon atmosphere.

b The ratio was determined from the crude reaction mixture by GC–MS.

c Isolated yields.

To investigate the mechanism and solvent-dependent selectivity, we performed a series of controlled experiments (Scheme 2). The optimized reaction conditions for the synthesis of **3a** (Table 1, entry 1) were monitored by GC–MS, confirming the initial formation of **2a** and its gradual conversion into **3a**, with a 1:99 (**2a**/**3a**) ratio observed after 1.5 h (Scheme 2A). The base-promoted ring contraction was validated by treating **2a** with KOH in DMSO at 120 °C, furnishing **3a** in 70% yield (Scheme 2B). Notably, this transformation also occurred in DMF, even at lower KOH loading (Scheme 2B), ruling out the possibility that the selective formation of **2a** in DMF (Table 1, entry 14) arises from a superbase effect unique to DMSO.^29^ This conclusion was further supported by an experiment using a crown ether to enhance basicity,^16^ which still failed to promote the formation of **3a** in DMF (99:1 **2a**/**3a**; Scheme 2C). However, using a large excess of KOH (5.0 equiv) significantly increased the formation of **3a** (65:35 **2a**/**3a**), indicating that hydroxide availability is likely limited under the optimized DMF conditions, thereby disfavoring the ring contraction pathway.

**Scheme 2:**
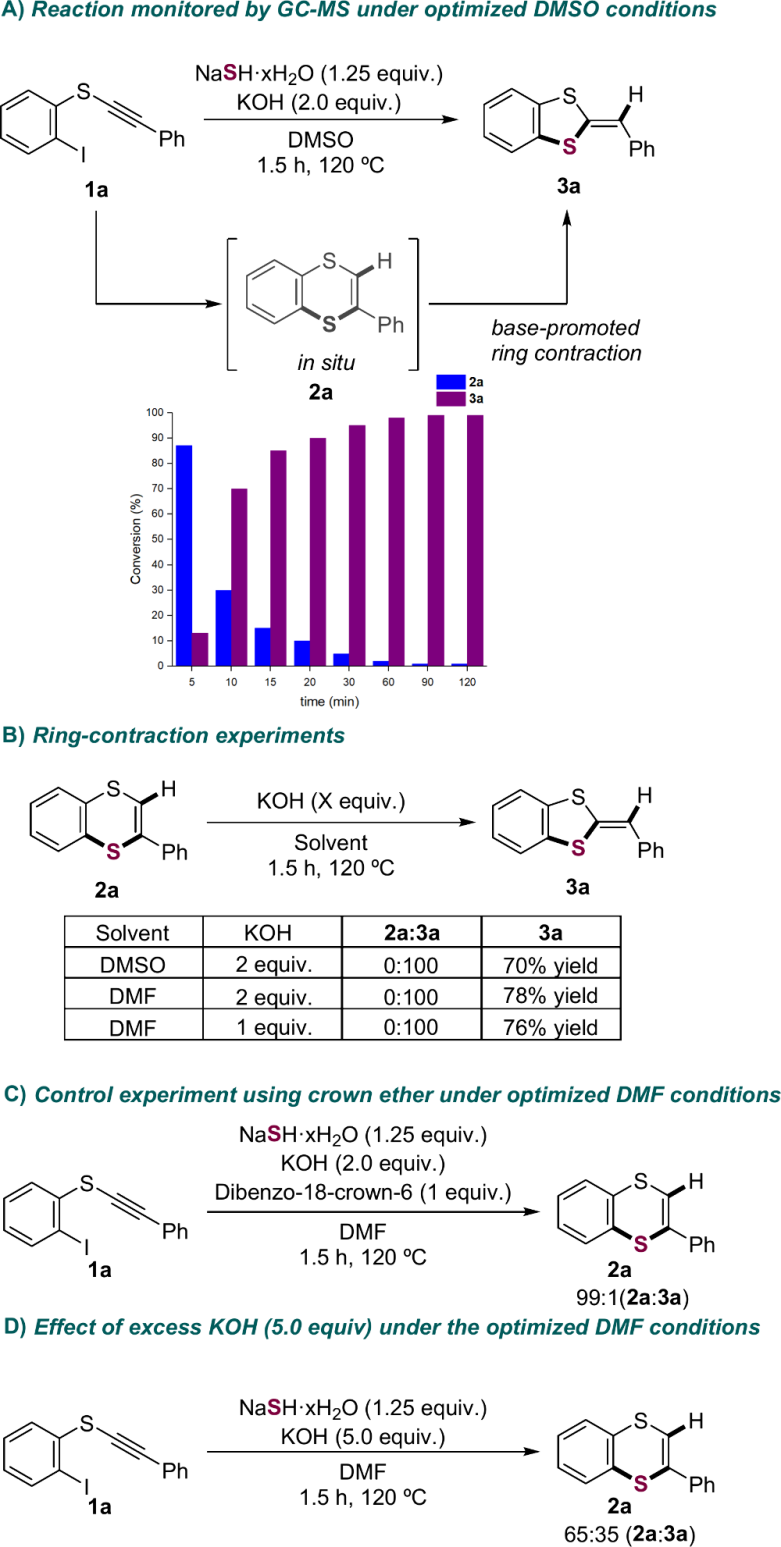
Control Experiments

Considering that HS^–^/S^2–^ species can disproportionate to S_3_^•^ ^–^ in both DMF and DMSO (Scheme 3A and B),^26,30^ we carried out colorimetric and UV–vis experiments to evaluate the influence of KOH on the S^2–^/S_3_^•^ ^–^ equilibrium in these solvents. The presence of sulfur species in solution was evidenced by characteristic color changes and spectroscopic analyses (see sections 5 and 6 of the Supporting Information for details). These studies revealed that S_3_^•^ ^–^ exhibits a significantly longer lifetime in DMF, consistent with its reported higher stability in this solvent.^31^ Our results further indicate that S_3_^•^ ^–^ is gradually trapped by a DMF-derived radical generated via solvent deprotonation followed by single-electron oxidation^32^ (Scheme 3C), as suggested by photophysical analyses and HRMS detection of adducts **5** and **6**. This trapping process consumes hydroxide, thereby reducing its availability and suppressing the ring contraction of **2a** in DMF. In contrast, in DMSO, the basic medium rapidly converts sulfur radicals into S_*x*_^2–^ species^33^ and no trapping of S_3_^•^ ^–^ was observed, indicating that free hydroxide remains available to promote the ring contraction of **2a** (Scheme 3D).

**Scheme 3:**
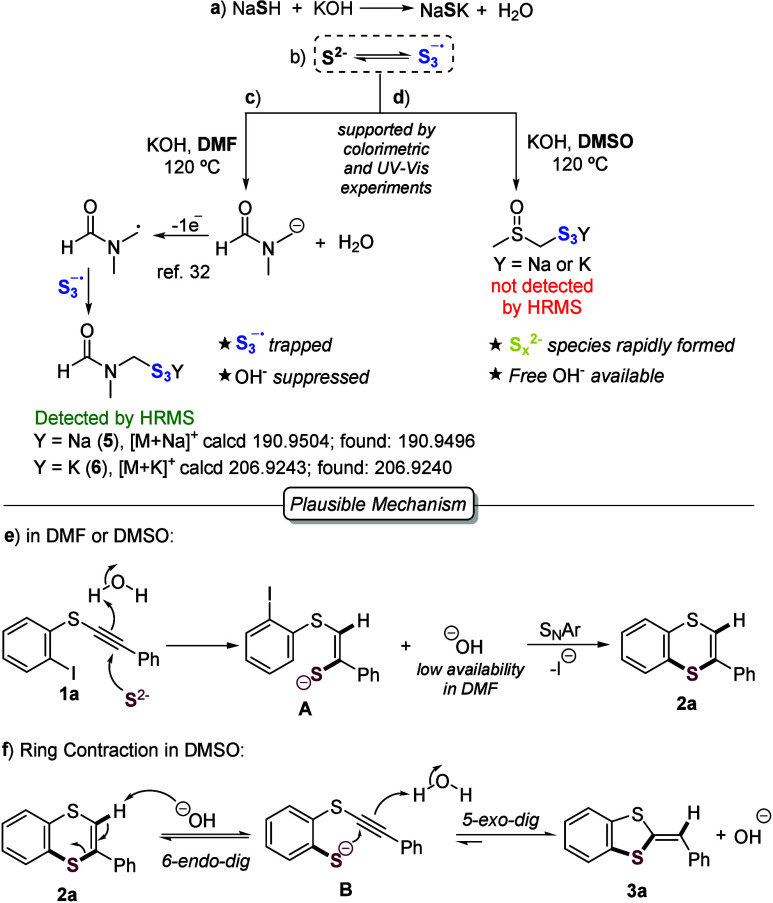
Mechanistic Considerations and Plausible Pathway

Based on these results, along with control experiments employing TEMPO that ruled out a radical pathway (see section 8 of the Supporting Information for details), a plausible mechanism is proposed (Scheme 3E). In both solvents, the reaction is initiated with a β-regioselective addition of S^2–^ to the C≡C triple bond of **1a**, followed by protonation to give intermediate **A**. This intermediate then undergoes an intramolecular S_N_Ar reaction to afford six-membered product **2a**. In DMSO, hydroxide ions promote the ring opening of *in situ* generated **2a** by abstracting vinylic hydrogen adjacent to sulfur,^16^ forming intermediate **B**. This intermediate preferentially undergoes a 5-*exo*-*dig* cyclization rather than a 6-*endo*-*dig* process, driven by the greater thermodynamic stability of the 1,3-dithiole ring and the lower acidity of dithiafulvenes,^16b^ leading to the formation of the five-membered product **3a** (Scheme 3F).

We next investigated the scope and limitations of the reactions (Scheme 4, methods A and B). The scalability was demonstrated using 1.0 mmol of **1a** under method A, yielding **2a** in 81% with 99:1 selectivity (**2a**/**3a**) (see the Supporting Information for details). Initial scope studies showed that both methods proceeded smoothly with substrates bearing alkyl groups, such as methyl and butyl, at various positions on the benzene ring, affording the corresponding six- and five-membered products in good yields and excellent selectivity (**2b**–**2d** and **3b**–**3d**). A 2,3-dimethyl substitution pattern was also well-tolerated, indicating compatibility with steric hindrance (**2e** and **3e**).

**Scheme 4:**
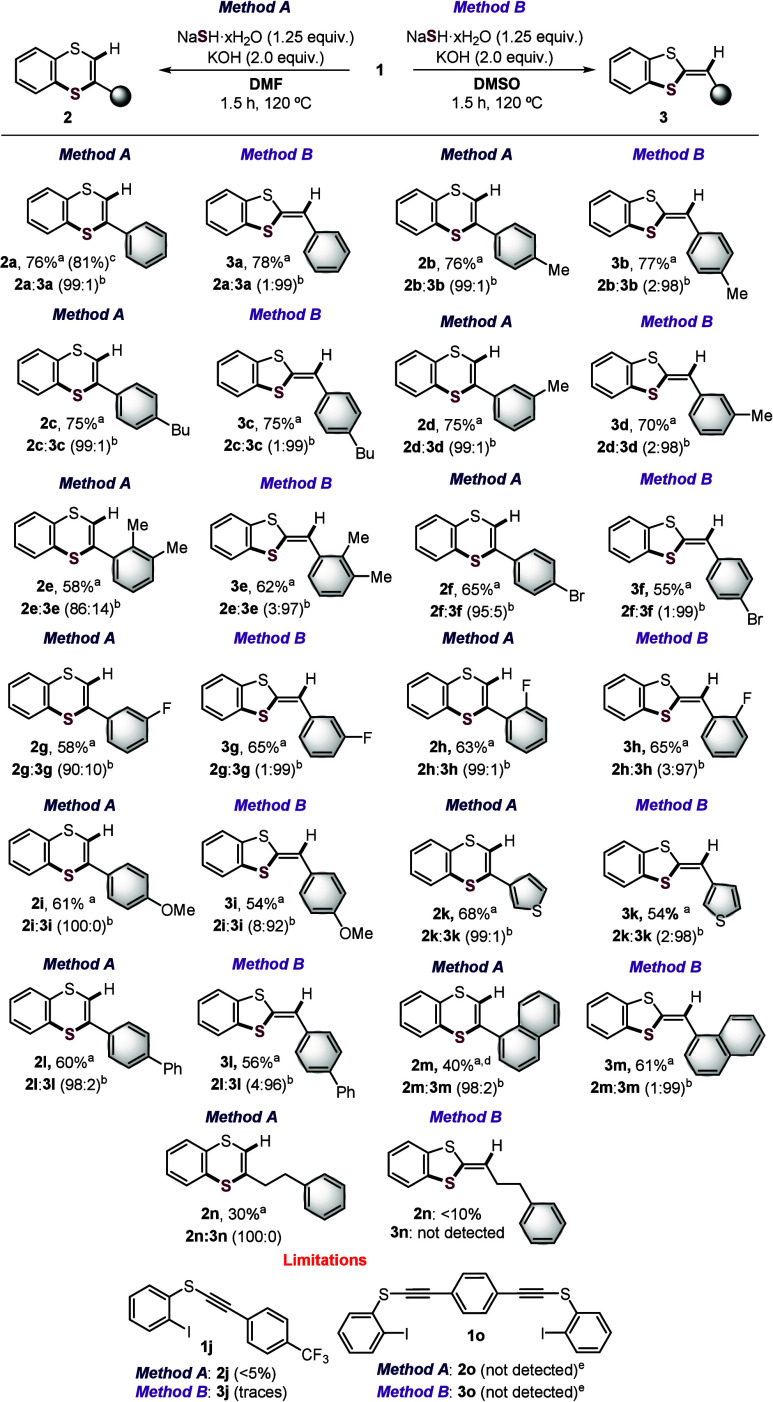
Reaction Scope^a^

* Reaction conditions: **1** (0.2 mmol), NaSH·*x*H_2_O (0.25 mmol), KOH (0.4 mmol), and solvent (1 mL) at 120 °C for 1.5 h under an argon atmosphere.
a Isolated yields.
b The ratio was determined by GC–MS from the crude reaction mixture before purification.
c The reaction was performed on a 1.0 mmol scale.
d The reaction was performed at 90 °C.
e NaSH·*x*H_2_O (0.5 mmol) and KOH (0.8 mmol).

Methods A and B were similarly effective and selective for substrates bearing weak electron-withdrawing groups, such as bromo and fluoro substituents, at different positions (**2f**–**2h** and **3f**–**3h**). Importantly, the bromo substituent in **2f** and **3f** offers a valuable handle for further functionalizations via cross-coupling reactions,^34^ while the successful cyclizations using 2-fluorinated substrate **1h** (**2h** and **3h**) highlight the chemoselectivity of the addition of S^2–^ species to the C≡C triple bond, with no evidence of competing benzo­[*b*]­thiophene formation.^21^ Additionally, the protocols also proved effective for 4-methoxy-substituted substrate **1i** (**2i** and **3i**), which contains a key functional group for developing push–pull dithiafulvene derivatives,^35^ affording moderate to good yields with exclusive formation of **2i** (100:0) and high selectivity for **3i** (8:92 **2i**/**3i**).

The scope was successfully expanded to heteroaromatic and π-extended systems, such as thienyl, biphenyl, and naphtyl groups, which are important motifs in the design of conjugated materials (**2k**–**2m** and **3k**–**3m**). Interestingly, an inseparable low-selectivity mixture (48:52 **2m**/**3m**) was obtained for **2m** under optimized method A, whereas method B furnished the five-membered product **3m** in 61% yield with excellent selectivity (1:99 **2m**/**3m**). Gratifyingly, **2m** could be accessed with high selectivity at a lower reaction temperature (90 °C, 98:2 **2m**/**3m**), which suppressed ring opening but resulted in a modest yield.

Method A also enabled the synthesis of compound **2n** bearing an alkyl substituent with complete selectivity but in only 30% yield, highlighting the importance of an adjacent π system to facilitate the nucleophilic attack to the C≡C bond. In contrast, method B produced less than 10% **2n** and no detectable formation of **3n**, suggesting that the ring-opening process is also disfavored in the absence of an aryl substituent. Finally, the *p*-CF_3_-substituted substrate **1j** proved incompatible with both methods (**2j**, <5% yield; **3j**, trace), and attempts to cyclize bifunctionalized alkynyl sulfide **1o** resulted in complex mixtures of unidentified products under both conditions.

Considering the relevance of TTFV derivatives and the applications of their benzo-fused analogues in electroluminescent devices,^36^ the synthetic utility of **3a** was demonstrated through its oxidative dimerization^37^ to afford dibenzo-fused tetrathiafulvalene vinylogue (DB-TTFV) **4a** in 42% yield (Scheme 5). Structural analysis by X-ray diffraction confirmed that **4a** adopts a pseudo-cisoid conformation, which reduces steric repulsion between the phenyl substituents (see the Supporting Information for details).^8c^

**Scheme 5:**
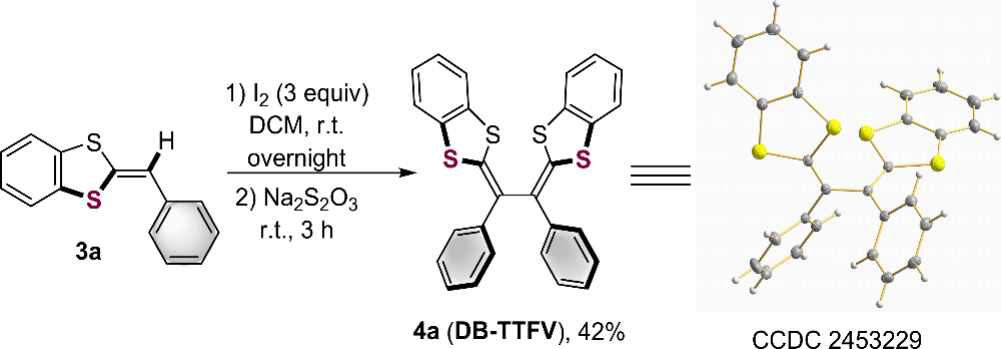
Synthesis of a DB-TTFV Derivative

We report a chemodivergent protocol for the selective synthesis of 1,4-benzo­[*b*]­dithiins and 1,4-benzodithiafulvenes from readily accessible 2-iodoaryl alkynyl sulfides. Six- or five-membered sulfur-rich heterocycles are obtained simply by changing the reaction medium from DMF to DMSO. Preliminary mechanistic studies suggest that differences in hydroxide availability, together with shifts in the S^2–^/S_3_^•^ ^–^ equilibrium under basic conditions, play a decisive role in enabling the observed chemodivergence between ring retention and ring contraction. The methodology demonstrates good functional group tolerance and operational simplicity, affording 25 examples in moderate to good yields with excellent selectivity. This study provides a useful platform for accessing structurally diverse 1,4-dithiins and 1,4-dithiafulvenes, offering valuable insights into sulfur-mediated cyclizations.

## Supplementary Material



## Data Availability

The data underlying this study are available in the published article and its Supporting Information.

## Notes

Experimental procedures, characterization data, NMR spectra for new products, and crystallographic data of **4a** (PDF)
