# Supplementary material for: Chemodivergent Synthesis of 1,4-Benzo[b]dithiins and 1,4-Benzodithiafulvenes
Source: Org Lett. 2026 Jan 27;28(5):1803–9. doi: 10.1021/acs.orglett.5c05364 (PMC12888010; doi:10.1021/acs.orglett.5c05364)
Supplement: Supplementary file 1 [file ol5c05364_si_001.pdf]

Supporting Information

Chemodivergent Synthesis of 1,4-Benzo[*b*]dithiins and 1,4-Benzodithiafulvenes

Douglas B. Paixão,[a] Anita B. Kessler,[a] Fabiano S. Rodembusch,[a] Rafael Stieler,[a] Daniel S. Rampon,[b*] Paulo H. Schneider[a*]

[a]Instituto de Química, Departamento de Química Orgânica, Universidade Federal do Rio Grande do Sul (UFRGS), P.O. Box 15003, 91501-970 Porto Alegre-RS, Brazil.

[b]Laboratório de Polímeros e Catálise (LAPOCA), Departamento de Química, Universidade Federal do Paraná-UFPR, P.O. Box 19061, Curitiba, PR, 81531-990, Brazil.

Corresponding Author: *paulos@iq.ufrgs.br*

Corresponding Author: *danielrampon@ufpr.br*

Table of Contents

1. General Information 2

2. Synthesis of Starting Materials 3

2.1. *General procedure A*: synthesis of compounds S1-S15. 3

2.1.1. Characterization data of compounds S4, S5, S7, S8, S11 and S14. 4

2.2. *General procedure B*: synthesis of compounds 1a-1o. 7

2.2.1. Characterization data of compounds 1a-1o. 8

3. Synthesis of 1,4-benzo[*b*]dithiins and 1,4-benzodithiafulvenes 13

3.1. *General Procedure C*: Synthesis of 1,4-benzo[*b*]dithiins 2. 13

3.2. *General Procedure C2*: Synthesis of 2a on a 1.0 mmol scale. 14

3.3. *General Procedure D*: Synthesis of 1,4-benzodithiafulvenes 3. 14

3.4. Characterization data of compounds 2 and 3. 16

3.5. *General procedure E*: base-promoted ring contraction of 2a into 3a. 52

4. *General procedure F*: synthesis of 4a through dimerization of 3a. 53

5. Colorimetric Analyses 54

5.1. Colorimetric studies of NaSH·xH_2O in DMF and DMSO before and after KOH addition.......... 54
6. UV-Vis Analyses.......... 59
6.1 UV-Vis spectra studies of NaSH·xH_2O in DMF and DMSO before and after KOH addition.......... 59
7. HRMS analysis of NaSH·xH_2O in DMF and DMSO after KOH addition.......... 61
8. Radical Trapping Experiments.......... 63
9. Single crystal X-ray diffraction.......... 64
10. NMR Spectra.......... 66
11. References.......... 113

1. General Information

Unless otherwise stated, all reagents were purchased from commercial suppliers and used without further purification. *N,N*-Dimethylformamide (DMF) and dimethyl sulfoxide (DMSO) were stored over activated 4 Å molecular sieves (pre-heated at 180 ºC for 5 h) in argon-purged flask prior to use. Sodium hydrosulfide hydrate (NaSH·xH_2O) was purchased from Sigma Aldrich® (CAS 207683-19-0; LOT # SHBL0535, 60.3% NaSH, flakes) and used as received. Potassium hydroxide (KOH) was manually ground into a fine powder and dried under vacuum prior to use. Reaction progress was monitored by thin-layer chromatography (TLC) on silica gel 60 F_{254} aluminum sheets. Spots were visualized under UV light (254 nm) or by staining with a 5% vanillin solution in 10% H_2SO_4, followed by heating. Purification of compounds was performed by flash column chromatography on silica gel using a Biotage® Selekt system. ^1H NMR and $^{13}C\{^1H\}$ NMR were recorded at 298 K on a Bruker Avance 400 spectrometer. Chemical shifts (δ) are reported in parts per million (ppm) relative to tetramethylsilane (TMS) as the internal standard for ^1H NMR spectra recorded in $CDCl_3$ and referenced to the residual solvent signal of $CDCl_3$ at δ = 77.0 for $^{13}C\{^1H\}$ NMR. Coupling constants (J) are reported in hertz (Hz). Hydrogen coupling patterns are described as singlet (s), doublet (d), triplet (t), quartet (q), quintet (quint), sextet (sext), doublet of doublets (dd), doublet of doublet of doublets (ddd), doublet of triplets (dt), and broad signal (bs). Splitting patterns that could not be unambiguously assigned are reported as multiplet (m). Melting points were determined using a Büchi Melting Point M-560 equipment. Gas

chromatography-mass spectrometry (GC-MS) analyses were performed using a Shimadzu GC-2010 gas chromatograph equipped with a J&W HP-5MS column and coupled to a Shimadzu GCMS-QP2010 mass spectrometer. UV-Vis absorption spectra were obtained on a Shimadzu UV-2450 spectrophotometer. The obtained chromatograms were processed using Origin 2017 software. HRMS analyses were performed on a Bruker Daltonics Impact II QTOF mass spectrometer. Spectra were acquired in positive ion mode.

2. Synthesis of Starting Materials

The starting materials **1** were synthesized according to the following sequence:

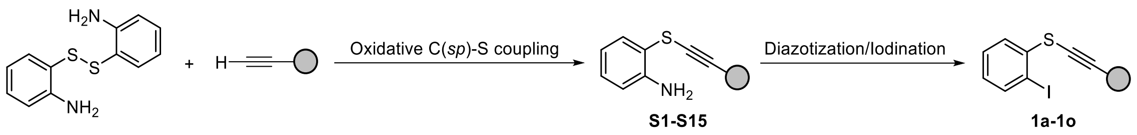

2.1. ***General procedure A***: synthesis of compounds **S1-S15**.[1]

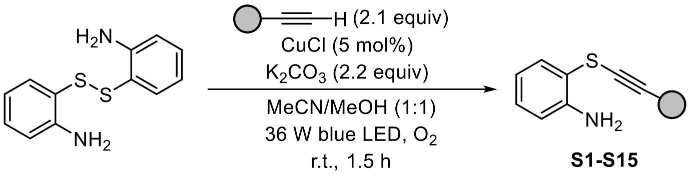

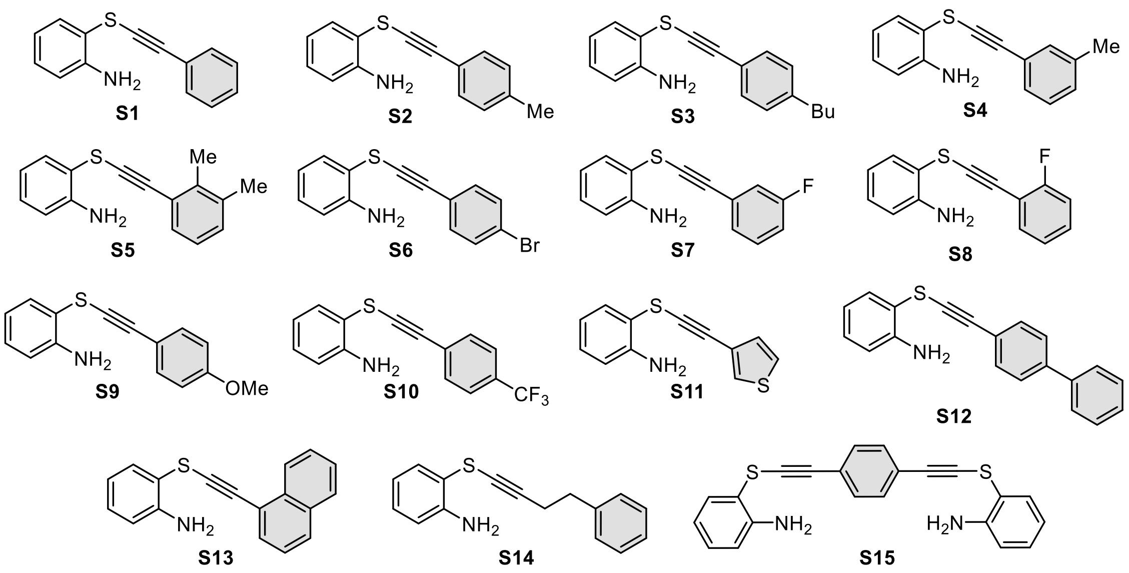

A 250 mL single-neck flask, equipped with a magnetic stirring bar, was charged with a mixture of acetonitrile (60 mL) and methanol (60 mL), followed by bubbling

oxygen through the solution for 10 minutes. Subsequently, 2-aminophenyl disulfide (0.745 g, 3 mmol), the corresponding terminal alkyne (6.3 mmol), K_2CO_3 (0.912 g, 6.6 mmol), and CuCl (0.015 g, 0.15 mmol) were added. The reaction mixture was stirred at room temperature under irradiation with a 36 W Kessil H150 blue LED lamp for 1.5 h (the lamp was positioned approximately 4 cm from the flask, and the temperature was controlled by a fan). After completion of the reaction, the mixture was filtered using Celite, and the solvent was removed under reduced pressure. The crude product was purified by automated flash chromatography on silica gel using the Biotage® Selekt flash chromatography system.

*Note 1: Compound **S1** was isolated as a light-yellow oil, becoming dark after weeks at room temperature without light protection. Based on these observations, all compounds **S1-S15** were stored under refrigeration (4 ºC) and protected from light after isolation.*

*Note 2: Compounds **S1, S2, S3, S6, S9, S12, S13,** and **S15** have been previously reported in the literature.[1,2] Compounds **S4, S5, S7, S8, S11** and **S14** are new compounds, and are fully characterized below. Compound **S10** was used in the next step without prior purification.*

2.1.1. Characterization data of compounds **S4**, **S5**, **S7**, **S8**, **S11** and **S14**.

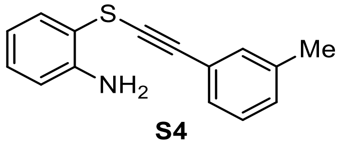

S4

2-((m-tolylethynyl)thio)aniline (**S4**). Following ***General Procedure A***, the product was isolated as a yellow oil in 68% yield (0.976 g) using a gradient elution starting from hexane to hexane/ethyl acetate (94:6). **^{1}H NMR** (400 MHz, $CDCl_3$): δ 7.51 (dd, *J* = 7.7, 1.3 Hz, 1H), 7.27 – 7.20 (m, 2H), 7.20 – 7.13 (m, 2H), 7.13 – 7.07 (m, 1H), 6.80 – 6.71 (m, 2H), 4.26 (bs, 2H), 2.30 (s, 3H). **^{13}C{^{1}H} NMR** (100 MHz, $CDCl_3$): δ 146.5, 138.0, 132.8, 132.2, 130.1, 129.3, 128.7, 128.2, 122.7, 119.0, 115.9, 114.2, 93.6, 76.1, 21.1. **HRMS** (ESI) *m/z*: $[M + H]^+$ calculated for $C_{15}H_{14}NS$ 240.0841; found 240.0842.

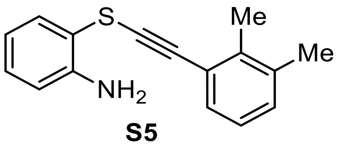

S5

2-(((2,3-dimethylphenyl)ethynyl)thio)aniline (**S5**). Following ***General Procedure A***, the product was isolated as a yellow oil in 45% yield (0.684 g) using a gradient elution starting from hexane to hexane/ethyl acetate (94:6). **^{1}H NMR** (400 MHz, $CDCl_3$): δ 7.52 (dd, J = 7.7, 1.5 Hz, 1H), 7.28 – 7.23 (m, 1H), 7.15 (td, J = 7.7, 1.5 Hz, 1H), 7.10 – 7.06 (m, 1H), 7.03 – 6.98 (m, 1H), 6.79 – 6.71 (m, 2H), 4.26 (bs, 2H), 2.34 (s, 3H), 2.25 (s, 3H). **^{13}C{^{1}H} NMR** (100 MHz, $CDCl_3$) δ 146.3, 138.7, 136.7, 132.6, 130.0, 129.94, 129.86, 125.3, 122.9, 119.0, 115.8, 114.6, 93.3, 79.2, 20.3, 17.5. **HRMS** (ESI) m/z: $[M + H]^+$ calculated for $C_{16}H_{16}NS$ 254.0998; found 254.1006.

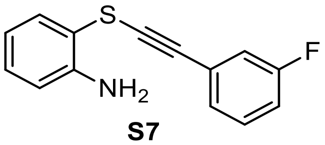

S7

2-(((3-fluorophenyl)ethynyl)thio)aniline (**S7**). Following ***General Procedure A***, the product was isolated as a yellow oil in 59% yield (0.861 g) using a gradient elution starting from hexane to hexane/ethyl acetate (94:6). **^{1}H NMR** (400 MHz, $CDCl_3$): δ 7.52 – 7.48 (m, 1H), 7.29 – 7.21 (m, 1H), 7.21 – 7.14 (m, 2H), 7.13 – 7.07 (m, 1H), 7.03 – 6.96 (m, 1H), 6.80 – 6.73 (m, 2H), 4.28 (bs, 2H). **^{13}C{^{1}H} NMR** (100 MHz, $CDCl_3$): δ 162.2 (d, $^1J_{C–F}$ = 246.7 Hz), 146.6, 133.2, 130.4, 129.8 (d, $^3J_{C–F}$ = 8.7 Hz), 127.4 (d, $^4J_{C–F}$ = 3.0 Hz), 124.7 (d, $^3J_{C–F}$ = 9.6 Hz), 119.1, 118.2 (d, $^2J_{C–F}$ = 22.8 Hz), 115.9, 115.6 (d, $^2J_{C–F}$ = 21.2 Hz), 113.5, 91.9 (d, $^4J_{C–F}$ = 3.4 Hz), 78.3. **HRMS** (ESI) m/z: $[M + H]^+$ calculated for $C_{14}H_{11}FNS$ 244.0591; found 244.0592.

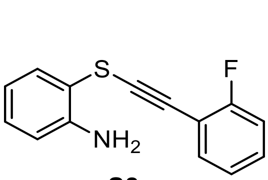

S8

2-(((2-fluorophenyl)ethynyl)thio)aniline (**S8**). Following ***General Procedure A***, the product was isolated as a light-yellow oil in 37% yield (0.540 g) using a gradient elution starting from hexane to hexane/ethyl acetate (96:4). **^{1}H NMR**

(400 MHz, $CDCl_3$): δ 7.55 – 7.48 (m, 1H), 7.40 (td, J = 7.5, 1.9 Hz, 1H), 7.32 – 7.22 (m, 1H), 7.17 (td, J = 7.6, 1.5 Hz, 1H), 7.10 – 6.99 (m, 2H), 6.80 – 6.71 (m, 2H), 4.29 (bs, 2H). **^{13}C{^{1}H} NMR** (100 MHz, $CDCl_3$): δ 162.8 (d, $^1J_{C\text{-}F}$ = 251.7 Hz), 146.6, 133.5 (d, $^4J_{C\text{-}F}$ = 0.7 Hz), 133.0, 130.3, 130.1 (d, $^3J_{C\text{-}F}$ = 7.9 Hz), 123.9 (d, $^3J_{C\text{-}F}$ = 3.7 Hz), 119.1, 116.0, 115.4 (d, $^2J_{C\text{-}F}$ = 20.9 Hz), 113.6, 111.6 (d, $^2J_{C\text{-}F}$ = 15.7 Hz), 86.6, 82.2 (d, $^3J_{C\text{-}F}$ = 3.3 Hz). **HRMS** (ESI) *m*/*z*: $[M + H]^+$ calculated for $C_{14}H_{11}FNS$ 244.0591; found 244.0590.

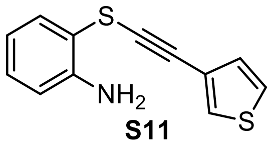

S11

2-((thiophen-3-ylethynyl)thio)aniline (**S11**). Following ***General Procedure A***, the product was isolated as a yellow oil in 50% yield (0.694 g) using a gradient elution starting from hexane to hexane/ethyl acetate (96:4). **^{1}H NMR** (400 MHz, $CDCl_3$): δ 7.52 – 7.48 (m, 1H), 7.46 (dd, J = 3.0, 1.2 Hz, 1H), 7.27 – 7.22 (m, 1H), 7.19 – 7.13 (m, 1H), 7.10 (dd, J = 5.0, 1.2 Hz, 1H), 6.79 – 6.72 (m, 2H), 4.27 (bs, 2H). **^{13}C{^{1}H} NMR** (100 MHz, $CDCl_3$): δ 146.5, 133.0, 130.2, 130.0, 129.7, 125.2, 121.9, 119.0, 115.9, 114.0, 88.3, 76.1. **HRMS** (ESI) *m*/*z*: $[M + H]^+$ calculated for $C_{12}H_{10}NS_2$ 232.0249; found 232.0250.

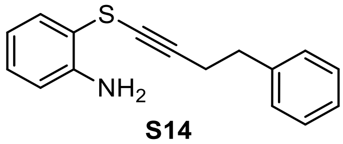

S14

2-((4-phenylbut-1-yn-1-yl)thio)aniline (**S14**). Following ***General Procedure A***, the product was isolated as a yellow oil in 30% yield (0.456 g) using a gradient elution starting from hexane to hexane/ethyl acetate (94:6). **^{1}H NMR** (400 MHz, $CDCl_3$) δ 7.38 (dd, J = 7.7, 1.5 Hz, 1H), 7.31 – 7.24 (m, 2H), 7.24 – 7.16 (m, 3H), 7.12 (td, J = 7.7, 1.5 Hz, 1H), 6.75 – 6.66 (m, 2H), 4.08 (s, 2H), 2.83 (t, J = 7.4 Hz, 2H), 2.62 (t, J = 7.4 Hz, 2H). **^{13}C{^{1}H} NMR** (100 MHz, $CDCl_3$): δ 146.3, 140.4, 132.6, 129.8, 128.5, 128.4, 126.3, 118.9, 115.7, 114.7, 94.3, 66.8, 34.9, 22.2. **HRMS** (ESI) *m*/*z*: $[M + H]^+$ calculated for $C_{16}H_{16}NS$ 254.0998; found 254.1000.

2.2. ***General procedure B***: synthesis of compounds **1a-1o**.[3]

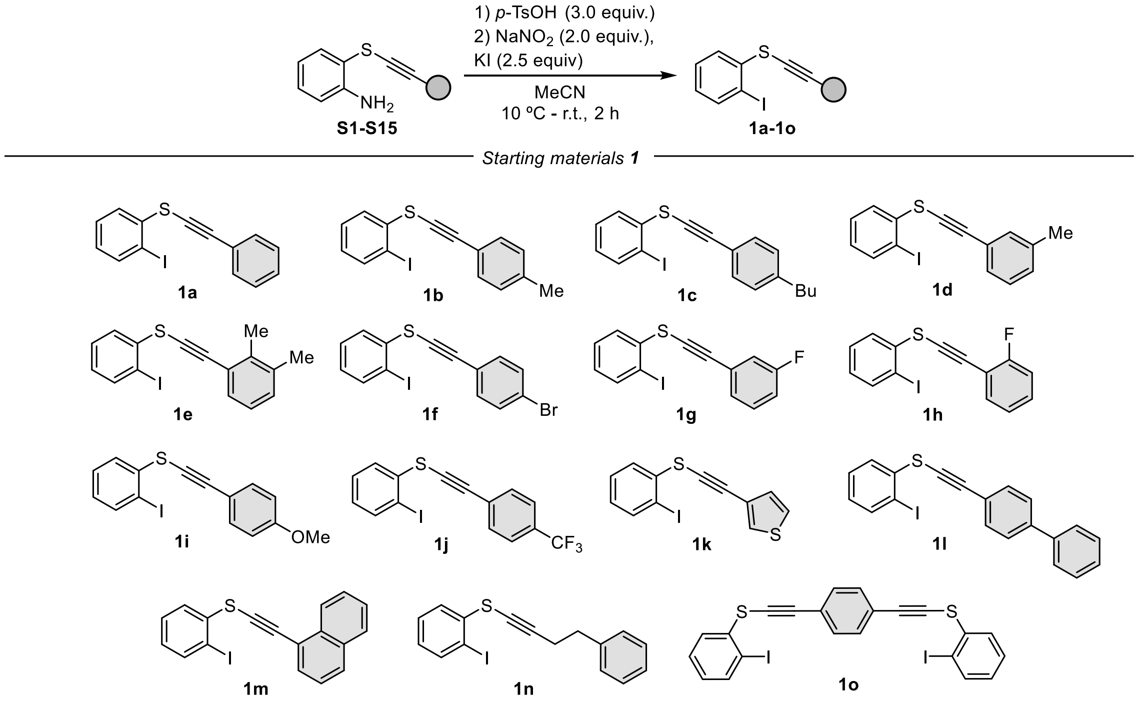

A solution of *p*-TsOH·H_2O (1.71 g, 9.0 mmol) in MeCN (25 mL) was prepared in a 50 mL round-bottom flask equipped with a magnetic stirring bar. To this solution, the respective 2-aminoaryl alkynyl sulfide (**S1**-**S15**) (3.0 mmol) was added. The suspension was stirred at room temperature for 10 minutes and then cooled to 10 °C. A solution of $NaNO_2$ (0.414 g, 6.0 mmol) and KI (1.245 g, 7.5 mmol) in water (2.0 mL) was gradually added, and the reaction mixture was stirred at 10 °C for 10 minutes before being warmed to room temperature and stirred for an additional 2 h. The mixture was diluted with ethyl acetate (100 mL) and washed sequentially with saturated aqueous $Na_2S_2O_3$ (60 mL) and $NaHCO_3$ (60 mL) solutions. The organic layer was dried over anhydrous $MgSO_4$, filtered and concentrated under reduced pressure. The crude product was purified by automated flash chromatography on silica gel using a Biotage® Selekt flash chromatography system, yielding the 2-iodoaryl alkynyl sulfides **1**.

Note 1: For the synthesis of **1o**, MeCN (40 mL), *p*-TsOH·H_2O (3.42 g, 18 mmol) $NaNO_2$ (0.828 g, 12 mmol), KI (2.49 g, 15 mmol), and water (4 mL) were employed.

Note 2: All compounds **1a**-**1o** were stored under refrigeration (4 °C) after isolation.

2.2.1. Characterization data of compounds **1a-1o**.

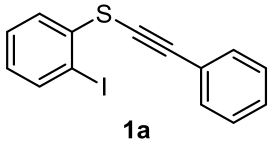

1a

(2-iodophenyl)(phenylethynyl)sulfane (**1a**). Following ***General Procedure B,*** the product was isolated as a white solid in 70% yield (0.706 g) using hexane as eluent. **mp** 47–49 ºC. **^{1}H NMR** (400 MHz, $CDCl_3$): δ 7.80 – 7.71 (m, 2H), 7.58 – 7.51 (m, 2H), 7.45 – 7.32 (m, 4H), 6.97 – 6.90 (m, 1H). **^{13}C{^{1}H} NMR** (100 MHz, $CDCl_3$): δ 139.3, 138.3, 131.9, 129.03, 128.96, 128.5, 127.5, 126.6, 122.5, 99.5, 93.6, 76.2. **HRMS** (ESI) *m*/*z*: $[M]^+$ calculated for $C_{14}H_9IS$, 335.9464; found, 335.9463.

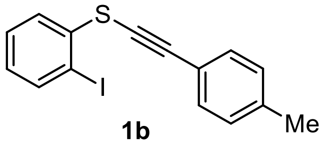

1b

(2-iodophenyl)(*p*-tolylethynyl)sulfane (**1b**). Following ***General Procedure B***, the product was isolated as a white solid in 68% yield (0.714 g) using hexane as eluent. **mp** 50–52 ºC. **^{1}H NMR** (400 MHz, $CDCl_3$): **^{1}H NMR** (400 MHz, $CDCl_3$) δ 7.79 – 7.70 (m, 2H), 7.47 – 7.36 (m, 3H), 7.16 (d, *J* = 8.0 Hz, 2H), 6.92 (td, *J* = 7.6, 1.4 Hz, 1H), 2.37 (s, 3H). **^{13}C{^{1}H} NMR** (100 MHz, $CDCl_3$): δ 139.4, 139.3, 138.5, 131.9, 129.2, 129.0, 127.4, 126.6, 119.5, 99.7, 93.5, 75.3, 21.6. **HRMS** (ESI) *m*/*z*: $[M]^+$ calculated for $C_{15}H_{11}IS$, 349.9621; found, 349.9611.

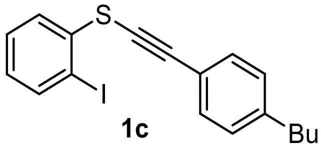

1c

(2-iodophenyl)(p-tolylethynyl)sulfane (**1c**). Following ***General Procedure B***, the product was isolated as a colorless oil in 50% yield (0.588 g) using hexane as eluent. **^{1}H NMR** (400 MHz, $CDCl_3$): δ 7.77 – 7.71 (m, 2H), 7.46 (d, *J* = 8.4 Hz, 2H), 7.43 – 7.36 (m, 1H), 7.17 (d, *J* = 8.4 Hz, 2H), 6.92 (td, *J* = 7.6, 1.2 Hz, 1H), 2.67 – 2.58 (m, 2H), 1.66 – 1.53 (m, 2H), 1.41 – 1.28 (m, 2H), 0.93 (t, *J* = 7.2 Hz, 3H). **^{13}C{^{1}H} NMR** (100 MHz, $CDCl_3$): δ 144.4, 139.2, 138.6, 132.0, 129.0, 128.6,

127.4, 126.6, 119.6, 99.8, 93.5, 75.3, 35.6, 33.3, 22.3, 13.9. **HRMS** (ESI) *m*/*z*: $[M]^+$ calculated for $C_{18}H_{17}IS$, 392.0090; found, 392.0077.

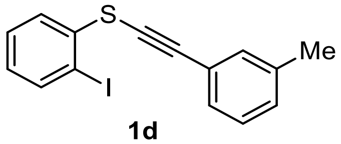

1d

(2-iodophenyl)(*m*-tolylethynyl)sulfane (**1d**). Following ***General Procedure B***, the product was isolated as a colorless oil in 60% yield (0.630 g) using hexane as eluent. **^{1}H NMR** (400 MHz, $CDCl_3$): δ 7.79 – 7.71 (m, 2H), 7.44 – 7.32 (m, 3H), 7.28 – 7.16 (m, 2H), 6.93 (td, *J* = 7.7, 1.5 Hz, 1H), 2.36 (s, 3H). **^{13}C{^{1}H} NMR** (100 MHz, $CDCl_3$): δ 139.3, 138.4, 138.2, 132.4,129.9, 129.02, 128.99, 128.3, 127.5, 126.6, 122.3, 99.8, 93.5, 75.7, 21.2. **HRMS** (ESI) *m*/*z*: $[M+H]^+$ calculated for $C_{15}H_{12}IS$ 350.9699; found, 350.9703.

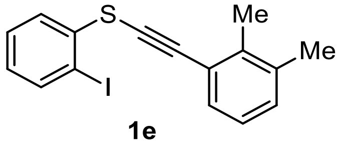

1e

((2,3-dimethylphenyl)ethynyl)(2-iodophenyl)sulfane (**1e**). Following ***General Procedure B*** on a 2.5 mmol scale, the product was isolated as a white solid in 64% yield (0.583 g) using hexane as eluent. mp 88–90 ºC. **^{1}H NMR** (400 MHz, $CDCl_3$): δ 7.81 – 7.72 (m, 2H), 7.43 – 7.36 (m, 2H), 7.19 – 7.12 (m, 1H), 7.08 (t, *J* = 7.6 Hz, 1H), 6.93 (td, *J* = 7.6, 1.5 Hz, 1H), 2.44 (s, 3H), 2.30 (s, 3H). **^{13}C{^{1}H} NMR** (100 MHz, $CDCl_3$): δ 139.3, 139.1, 138.7, 137.0, 130.6, 130.3, 129.0, 127.4, 126.6, 125.5, 122.5, 99.2, 93.4, 78.9, 20.3, 17.7. **HRMS** (ESI) *m*/*z*: $[M]^+$ calculated for $C_{16}H_{13}IS$ 363.9777; found, 363.9767.

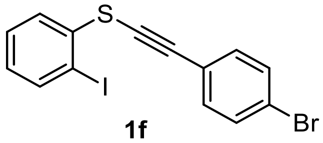

1f

((4-bromophenyl)ethynyl)(2-iodophenyl)sulfane (**1f**). Following ***General Procedure B***, the product was isolated as a white solid in 61% yield (0.760 g) using hexane as eluent. mp 87–89 ºC. **^{1}H NMR** (400 MHz, $CDCl_3$) δ 7.77 (d, *J* = 7.8 Hz, 1H), 7.74 – 7.67 (m, 1H), 7.49 (d, *J* = 8.4 Hz, 2H), 7.45 – 7.34 (m, 3H),

6.99 – 6.91 (m, 1H). **^{13}C{^{1}H} NMR** (100 MHz, $CDCl_3$) δ 139.4, 137.9, 133.2, 131.7, 129.1, 127.7, 126.7, 123.3, 121.5, 98.3, 93.8, 77.8. **HRMS** (ESI) *m*/*z*: $[M]^+$ calculated for $C_{14}H_8BrIS$ 413.8569; found, 413.8567.

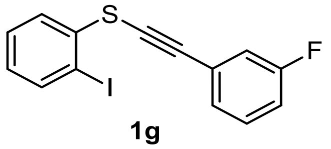

1g

((3-fluorophenyl)ethynyl)(2-iodophenyl)sulfane (**1g**). Following ***General Procedure B***, the product was isolated as a white solid in 63% yield (0.670 g) using hexane as eluent. mp 40–42 ºC. **^{1}H NMR** (400 MHz, $CDCl_3$): δ 7.77 (dd, *J* = 7.7, 1.2 Hz, 1H), 7.72 (dd, *J* = 8.0, 1.5 Hz, 1H), 7.42 (td, *J* = 8.0, 1.2 Hz, 1H), 7.36 – 7.28 (m, 2H), 7.25 – 7.19 (m, 1H), 7.13 – 7.03 (m, 1H), 6.95 (td, *J* = 7.7, 1.5 Hz, 1H). **^{13}C{^{1}H} NMR** (100 MHz, $CDCl_3$): δ 162.3 (d, $^{1}J_{C-F}$ = 247.2 Hz), 139.4, 137.8, 130.1 (d, $^{3}J_{C-F}$ = 8.7 Hz), 129.1, 127.7, 127.6 (d, $^{4}J_{C-F}$ = 3.1 Hz), 126.7, 124.3 (d, $^{3}J_{C-F}$ = 9.5 Hz), 118.4 (d, $^{2}J_{C-F}$ = 22.9 Hz), 116.3 (d, $^{2}J_{C-F}$ = 21.2 Hz), 98.1 (d, $^{4}J_{C-F}$ = 3.4 Hz), 93.8, 77.7. **HRMS** (ESI) *m*/*z*: $[M]^+$ calculated for $C_{14}H_8FIS$ 353.9375; found, 353.9368.

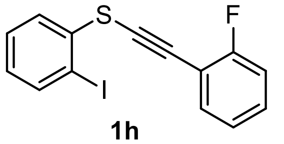

1h

((2-fluorophenyl)ethynyl)(2-iodophenyl)sulfane (**1h**). Following ***General Procedure B*** on a 2.0 mmol scale, the product was isolated as a white solid in 65% yield (0.460 g) using hexane as eluent. mp 48–50 ºC. **^{1}H NMR** (400 MHz, $CDCl_3$): δ 7.84 – 7.72 (m, 2H), 7.55 – 7.47 (m, 1H), 7.46 – 7.39 (m, 1H), 7.39 – 7.30 (m, 1H), 7.17 – 7.08 (m, 2H), 6.94 (td, *J* = 7.7, 1.5 Hz, 1H). **^{13}C{^{1}H} NMR** (100 MHz, $CDCl_3$): δ 162.8 (d, $^{1}J_{C-F}$ = 252.3 Hz), 139.3, 137.8, 133.3, 130.5 (d, $^{3}J_{C-F}$ = 8.0 Hz), 129.1, 127.7, 126.7, 124.1 (d, $^{3}J_{C-F}$ = 3.8 Hz), 115.6 (d, $^{2}J_{C-F}$ = 20.7 Hz), 111.3 (d, $^{2}J_{C-F}$ = 15.6 Hz), 93.6, 92.9, 81.8 (d, $^{3}J_{C-F}$ = 3.2 Hz). **HRMS** (ESI) *m*/*z*: $[M]^+$ calculated for $C_{14}H_8FIS$ 353.9370; found, 353.9364.

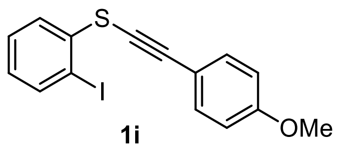

1i

(2-iodophenyl)((4-methoxyphenyl)ethynyl)sulfane (**1i**). Following ***General Procedure B***, the product was isolated as a white solid in 21% yield (0.231 g) using a gradient elution starting from hexane to hexane/ethyl acetate (94:6). mp 83–85 ºC. **^{1}H NMR** (400 MHz, $CDCl_3$) δ 7.79 – 7.70 (m, 2H), 7.50 (d, *J* = 8.8 Hz, 2H), 7.43 – 7.35 (m, 1H), 6.95 – 6.86 (m, 3H), 3.84 (s, 3H). **^{13}C{^{1}H} NMR** (100 MHz, $CDCl_3$): δ 160.3, 139.2, 138.7, 133.9, 129.0, 127.4, 126.6, 114.6, 114.1, 99.6, 93.4, 74.4, 55.3. **HRMS** (ESI) *m*/*z*: $[M]^+$ calculated for $C_{15}H_{11}IOS$ 365.9570; found, 365.9566.

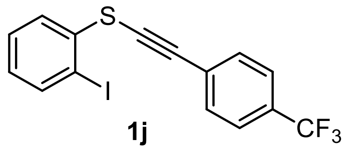

1j

(2-iodophenyl)((4-(trifluoromethyl)phenyl)ethynyl)sulfane (**1j**). Following ***General Procedure B***, the product was isolated as a white solid in 21% global yield (0.509 g) using *n*-hexane as eluent. mp 51–53 ºC. **^{1}H NMR** (400 MHz, $CDCl_3$) δ 7.78 (dd, *J* = 7.8, 1.3 Hz, 1H), 7.73 (dd, *J* = 8.0, 1.5 Hz, 1H), 7.65 – 7.58 (m, 4H), 7.47 – 7.40 (m, 1H), 6.97 (td, *J* = 7.8, 1.5 Hz, 1H). **^{13}C{^{1}H} NMR** (100 MHz, $CDCl_3$): δ 139.5, 137.5, 131.7, 130.4 (q, $^{2}J_{C-F}$ = 32.8 Hz), 129.1, 127.9, 126.8, 126.3, 125.4 (q, $^{3}J_{C-F}$ = 3.8 Hz), 123.8 (q, $^{1}J_{C-F}$ = 272.3 Hz), 98.0, 94.0, 79.6. **HRMS** (ESI) *m*/*z*: $[M]^+$ calculated for $C_{15}H_8F_3IS$ 403.9338; found, 403.9337.

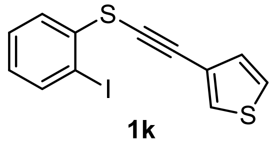

1k

3-(((2-iodophenyl)thio)ethynyl)thiophene (**1k**). Following ***General Procedure B***, the product was isolated as white solid in 68% yield (0.698 g) using hexane as eluent. mp 42–44 ºC. **^{1}H NMR** (400 MHz, $CDCl_3$) δ 7.78 – 7.68 (m, 2H), 7.61 (dd, *J* = 3.0, 1.2 Hz, 1H), 7.42 – 7.36 (m, 1H), 7.31 (dd, *J* = 5.0, 3.0 Hz, 1H), 7.21 (dd, *J* = 5.0, 1.2 Hz, 1H), 6.93 (td, 7.7, 1.5 Hz, 1H). **^{13}C{^{1}H} NMR** (100 MHz, $CDCl_3$):

δ 139.3, 138.3, 130.7, 130.1, 129.0, 127.5, 126.7, 125.6, 121.6, 94.4, 93.5, 75.8. **HRMS** (ESI) *m*/*z*: $[M]^+$ calculated for $C_{12}H_7IS_2$ 341.9028; found, 341.9029.

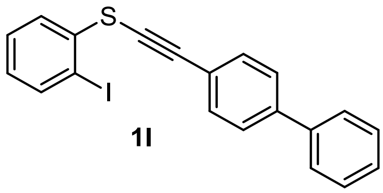

1l

([1,1'-biphenyl]-4-ylethynyl)(2-iodophenyl)sulfane (**1l**). Following ***General Procedure B***, the product was isolated as a white solid in 71% yield (0.878 g) using hexane as eluent. mp 77–79 ºC. **^{1}H NMR** (400 MHz, $CDCl_3$) δ 7.80 – 7.74 (m, 2H), 7.65 – 7.56 (m, 6H), 7.51 – 7.34 (m, 4H), 6.94 (td, *J* = 7.6, 1.5 Hz, 1H). **^{13}C{^{1}H} NMR** (100 MHz, $CDCl_3$): δ 141.7, 140.1, 139.3, 138.3, 132.3, 129.0, 128.9, 127.8, 127.6, 127.1, 127.0, 126.7, 121.4, 99.4, 93.6, 76.9. **HRMS** (ESI) *m*/*z*: $[M]^+$ calculated for $C_{20}H_{13}IS$ 411.9777; found, 411.9772.

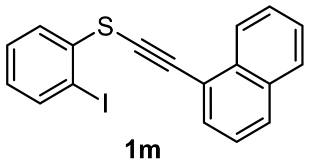

1m

(2-iodophenyl)(naphthalen-1-ylethynyl)sulfane (**1m**). Following ***General Procedure B***, the product was isolated as a white solid in 67% yield (0.776 g) using hexane as eluent. mp 60–62 ºC. **^{1}H NMR** (400 MHz, $CDCl_3$) δ 8.36 (d, *J* = 8.2 Hz, 1H), 7.94 – 7.83 (m, 3H), 7.83 – 7.76 (m, 2H), 7.63 – 7.51 (m, 2H), 7.51 – 7.38 (m, 2H), 6.95 (td, *J* = 7.7, 1.4 Hz, 1H). **^{13}C{^{1}H} NMR** (100 MHz, $CDCl_3$): δ 139.4, 138.5, 133.5, 133.2, 131.3, 129.6, 129.1, 128.4, 127.6, 127.1, 126.8, 126.6, 126.0, 125.2, 120.2, 97.6, 93.6, 80.8. **HRMS** (ESI) *m*/*z*: $[M+H]^+$ calculated for $C_{18}H_{12}IS$ 386.9699; found, 386.9685.

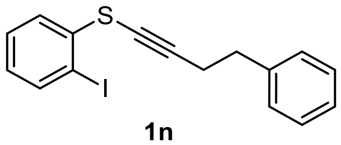

1n

(2-iodophenyl)(4-phenylbut-1-yn-1-yl)sulfane (**1n**). Following ***General Procedure B*** on a 1.8 mmol scale, the product was isolated as a colorless oil in 61% yield (0.400 g) using hexane as eluent. **^{1}H NMR** (400 MHz, $CDCl_3$) δ 7.70

(dd, J = 7.7, 1.3 Hz, 1H), 7.41 (dd, J = 8.0, 1.5 Hz, 1H), 7.35 – 7.29 (m, 2H), 7.29 – 7.22 (m, 4H), 6.91 – 6.84 (m, 1H), 2.93 (t, J = 7.2 Hz, 2H), 2.78 (t, J = 7.2 Hz, 2H). **$^{13}C\{^1H\}$ NMR** (100 MHz, $CDCl_3$): δ 140.2, 139.1, 138.7, 128.9, 128.54, 128.48, 127.1, 126.45, 126.39, 100.8, 93.2, 66.9, 34.8, 22.4. **HRMS** (ESI) *m*/*z*: $[M+H]^+$ calculated for $C_{16}H_{14}IS$ 364.9855; found, 364.9852.

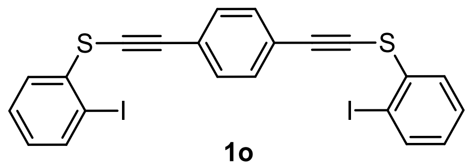

1o

1,4-bis(((2-iodophenyl)thio)ethynyl)benzene (**1o**). Following ***General Procedure B,*** the product was isolated as a yellow solid in 17% yield (0.303 g) using hexane/ethyl acetate (99:1) as eluent. mp 182–184 °C. **^1H NMR** (400 MHz, $CDCl_3$) δ 7.80 – 7.71 (m, 4H), 7.50 (s, 4H), 7.46 – 7.39 (m, 2H), 7.00 – 6.92 (m, 2H). **$^{13}C\{^1H\}$ NMR** (100 MHz, $CDCl_3$): δ 139.4, 137.9, 131.6, 129.1, 127.7, 126.7, 122.9, 98.9, 93.8, 79.0. **HRMS** (ESI) *m*/*z*: $[M]^+$ calculated for $C_{22}H_{12}I_2S_2$ 593.8464; found, 593.8458.

3. Synthesis of 1,4-benzo[*b*]dithiins and 1,4-benzodithiafulvenes

3.1. *General Procedure C*: Synthesis of 1,4-benzo[*b*]dithiins 2.

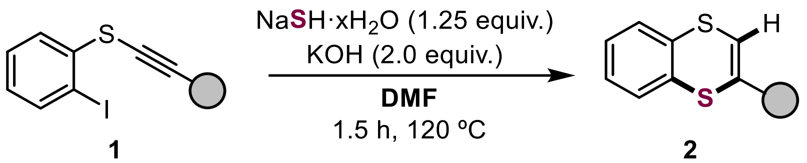

1 **2**

In a flame-dried 25 mL Schlenk tube equipped with a magnetic stirring bar and under an argon atmosphere, $NaSH \cdot xH_2O$ (60.3% flakes, 0.023 g, 0.25 mmol), KOH powder (0.022 g, 0.4 mmol), 2-iodoaryl alkynyl sulfide **1** (0.2 mmol), and DMF (1 mL) were added. The Schlenk tube was placed in a preheated oil bath at 120 °C and stirred for 1.5 h under an argon atmosphere. Afterward, the reaction was cooled to room temperature and diluted with ethyl acetate (90 mL). The mixture was washed with saturated aqueous NaCl solution (2 × 60 mL). The organic layer was dried over anhydrous $MgSO_4$, filtered, and concentrated under reduced pressure. The crude product was further purified by automated flash

chromatography on silica gel using a Biotage® Selekt flash chromatography system to afford 1,4-benzo[*b*]dithiins **2**.

3.2. ***General Procedure C2***: Synthesis of **2a** on a 1.0 mmol scale.

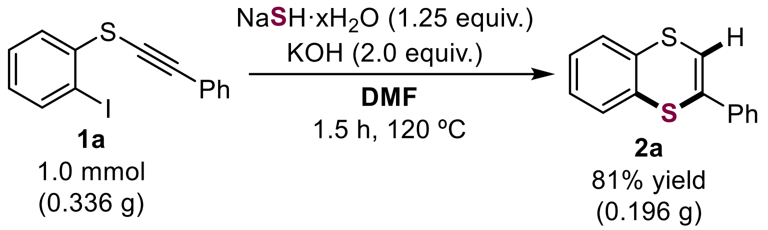

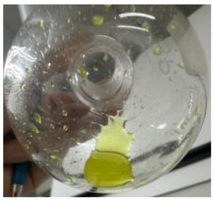

In a flame-dried 25 mL Schlenk tube equipped with a magnetic stirring bar and under an argon atmosphere, $NaSH\cdot xH_2O$ (60.3% flakes, 0.116 g, 1.25 mmol), KOH powder (0.112 g, 2.0 mmol), **1a** (0.336 g, 1.0 mmol), and DMF (5 mL) were added. The Schlenk tube was then placed in a preheated oil bath at 120 ºC and stirred for 1.5 h under an argon atmosphere. Afterward, the reaction was cooled to room temperature and diluted with ethyl acetate (200 mL). The mixture was washed with saturated aqueous NaCl solution (2 × 120 mL). The organic layer was dried over anhydrous $MgSO_4$, filtered, and concentrated under reduced pressure. The crude product was further purified by automated flash chromatography on silica gel using a Biotage® Selekt flash chromatography system to afford 1,4-benzo[*b*]dithiin **2a** as a yellow oil in 81% yield (0.196 g).

3.3. ***General Procedure D***: Synthesis of 1,4-benzodithiafulvenes **3**.

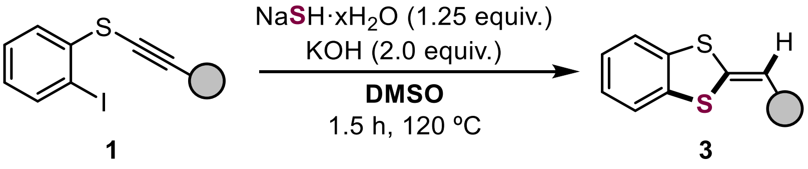

In a flame-dried 25 mL Schlenk tube equipped with a magnetic stirring bar and under an argon atmosphere, $NaSH\cdot xH_2O$ (60.3% flakes, 0.023 g, 0.25 mmol), KOH powder (0.022 g, 0.4 mmol), 2-iodoaryl alkynyl sulfide **1** (0.2 mmol), and DMSO (1 mL) were added. The Schlenk tube was then placed in a preheated oil bath at 120 ºC and stirred for 1.5 h under an argon atmosphere. Afterward, the reaction was cooled to room temperature and diluted with ethyl acetate (90 mL). The mixture was washed with saturated aqueous NaCl solution (2 × 60 mL). The

organic layer was dried over anhydrous $MgSO_4$, filtered, and concentrated under reduced pressure. The crude product was purified by automated flash chromatography on silica gel using a Biotage® Selekt flash chromatography system to afford 1,4-benzodithiafulvenes **3**.

*<u>Note</u>: Compound **3a**, isolated as a light-yellow solid during optimization studies, gradually turned deep purple after weeks at room temperature without light protection. Based on these observations, all compounds **2** and **3** were stored under refrigeration (4 ºC) and protected from light after isolation.*

*<u>Experimental observations</u>**:** The obtained 1,4-dithiins **2** typically develop a moss-green coloration upon visualization on TLC plates using an acidic vanillin solution in ethanol followed by heating, whereas the corresponding 1,4-dithiafulvenes **3** usually exhibit a characteristic purple color under the same conditions (Figure S1):*

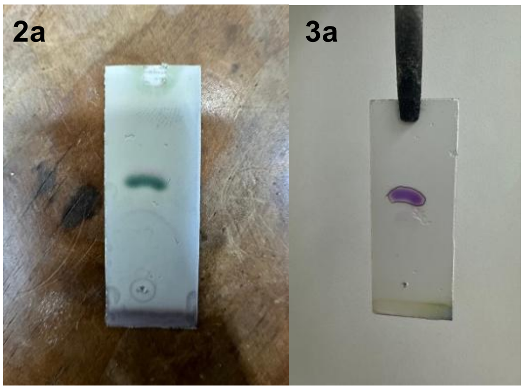

Figure S1. Representative TLC plates of compounds **2a** and **3a,** eluted with hexane and developed using an acidic vanillin solution in ethanol followed by heating.

3.4. Characterization data of compounds **2** and **3**.

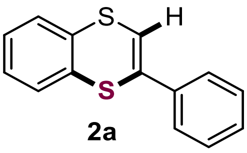

2a

2-phenylbenzo[*b*][1,4]dithiine (**2a**).[4] Following ***General Procedure C***, the product was isolated as a light-yellow oil in 76% yield (37 mg) using hexane as eluent. ^{1}**H NMR** (400 MHz, $CDCl_3$): δ 7.64 – 7.57 (m, 2H), 7.47 – 7.41 (m, 1H), 7.40 – 7.30 (m, 4H), 7.27 – 7.21 (m, 2H), 6.66 (s, 1H). ^{13}**C{^{1}H} NMR** (100 MHz, $CDCl_3$): δ 140.5, 137.1, 134.9, 134.2, 128.6, 128.57, 128.56, 127.85, 127.82, 127.6, 126.9, 118.3. **HRMS** (ESI) *m*/*z*: $[M]^+$ calculated for $C_{14}H_{10}S_2$, 242.0218; found, 242.0212.

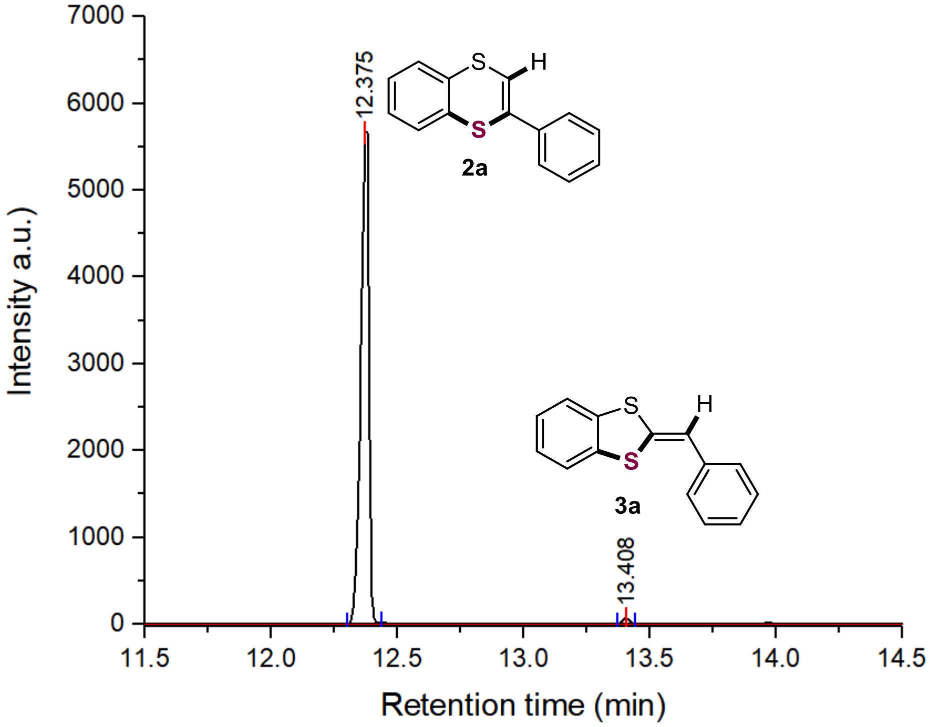

Figure S2. GC-MS expanded chromatogram of the crude reaction mixture for the synthesis of **2a**.

Peak#	Retention time (min)	Area	Area %	Height	Height %
**2a**	12.375	214523	98.98	5667	98.87
**3a**	13.408	2205	1.02	65	1.13
		216728	100	5732	100

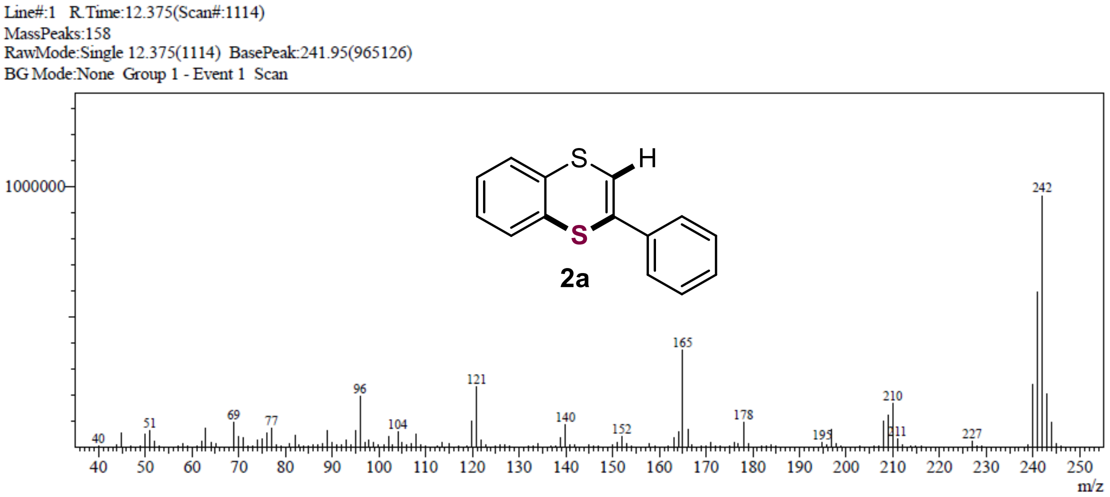

Figure S3. Mass spectrum of **2a**.

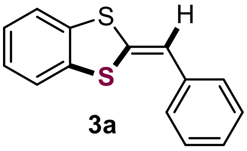

3a

2-benzylidenebenzo[*d*][1,3]dithiole (**3a**).[5,6] Following ***General Procedure D***, the product was isolated as a light-yellow solid in 78% yield (38 mg) using hexane as eluent. mp 118–120 °C. **^{1}H NMR** (400 MHz, $CDCl_3$): δ 7.41 – 7.30 (m, 4H), 7.26 – 7.16 (m, 3H), 7.14 – 7.06 (m, 2H), 6.56 (s, 1H). **^{13}C{^{1}H} NMR** (100 MHz, $CDCl_3$): δ 136.5, 136.3, 134.7, 132.4, 128.5, 126.9, 126.0, 125.9, 125.5, 121.6, 120.9, 114.6. **HRMS** (ESI) *m*/*z*: [M]$^+$ calculated for $C_{14}H_{10}S_2$, 242.0218; found, 242.0224.

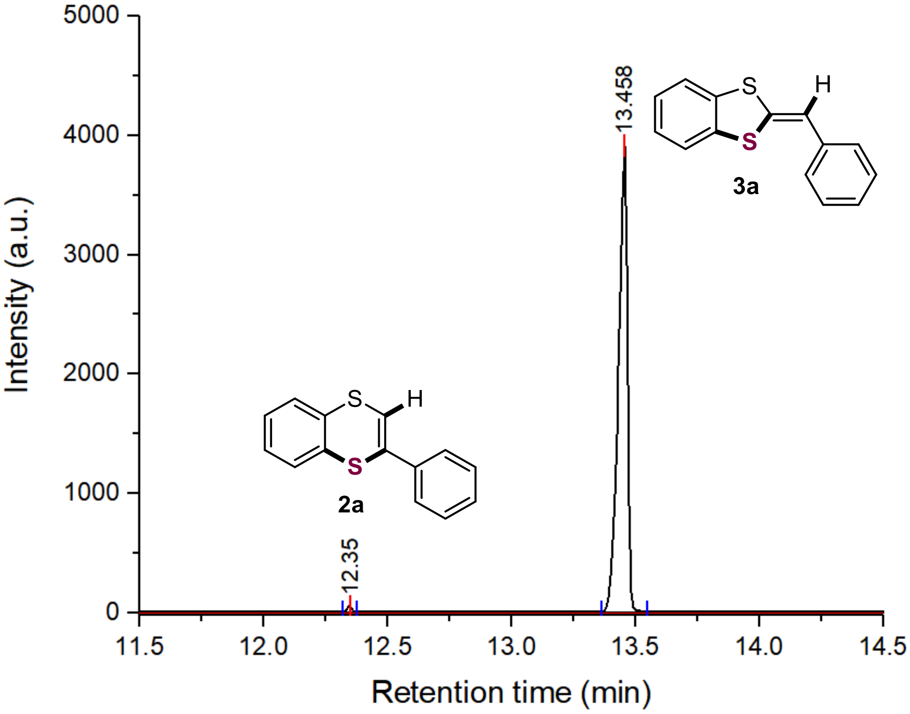

Figure S4. GC-MS expanded chromatogram of the crude reaction mixture for the synthesis of **3a**.

Peak#	Retention time (min)	Area	Area %	Height	Height %
**2a**	12.350	1517	0.91	60	1.51
**3a**	13.458	165501	99.09	3918	98.49
		167018	100	3978	100

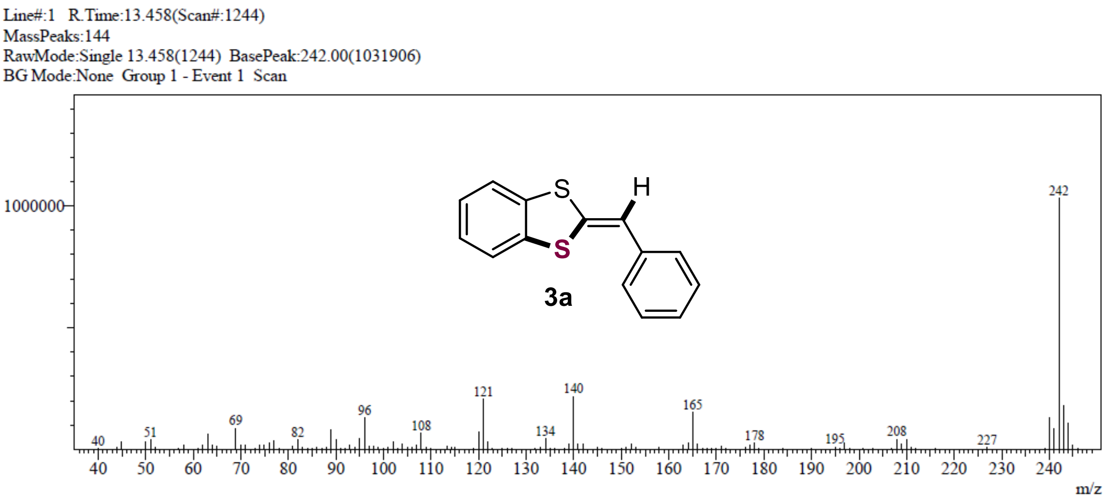

Figure S5. Mass spectrum of **3a**.

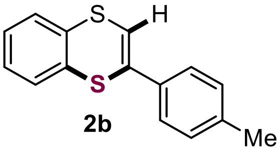

2-(*p*-tolyl)benzo[*b*][1,4]dithiine (**2b**).[4,7] Following ***General Procedure C**,* the product was isolated as a light-yellow oil in 76% yield (39 mg) using hexane as eluent. **^{1}H NMR** (400 MHz, $CDCl_3$): δ 7.50 (d, *J* = 8.1 Hz, 2H), 7.44 (dd, *J* = 5.8, 3.3 Hz, 1H), 7.36 (dd, *J* = 5.7, 3.4 Hz, 1H), 7.28 – 7.20 (m, 2H), 7.15 (d, *J* = 8.1 Hz, 2H), 6.61 (s, 1H), 2.35 (s, 3H). **^{13}C{^{1}H} NMR** (100 MHz, $CDCl_3$) δ 140.7, 138.7, 135.1, 134.42, 134.37, 129.3, 128.6, 127.9, 127.8, 127.6, 126.8, 117.1, 21.2. **HRMS** (ESI) *m*/*z*: $[M]^+$ calculated for $C_{15}H_{12}S_2$ 256.0375; found, 256.0370.

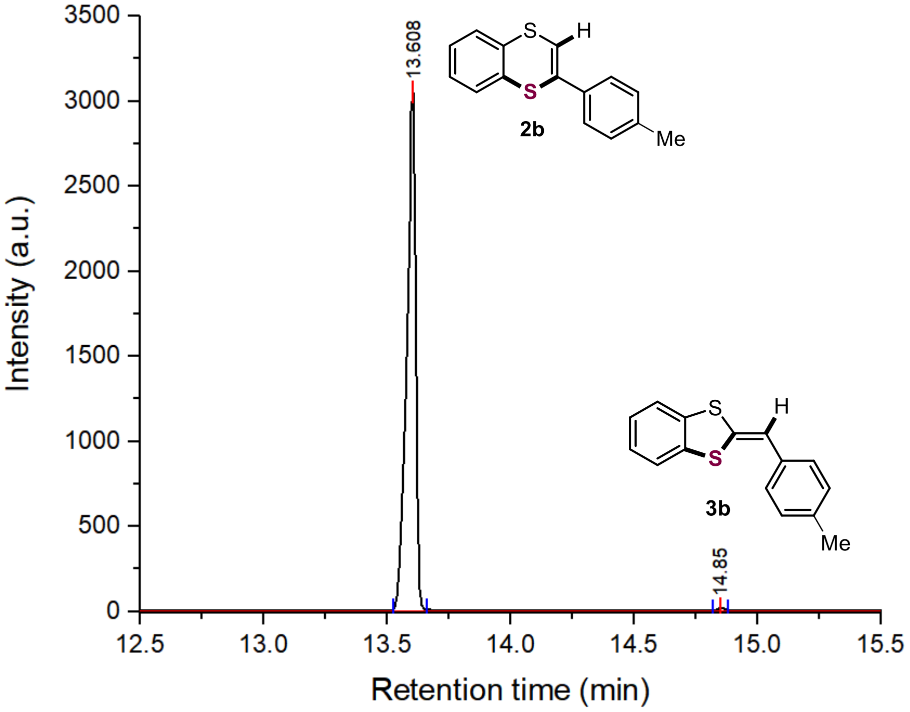

Figure S6. GC-MS expanded chromatogram of the crude reaction mixture for the synthesis of **2b**.

Peak#	Retention time (min)	Area	Area %	Height	Height %
**2b**	13.608	133321	99.44	3035	99.2
**3b**	14.850	744	0.56	25	0.8
		134065	100	3060	100

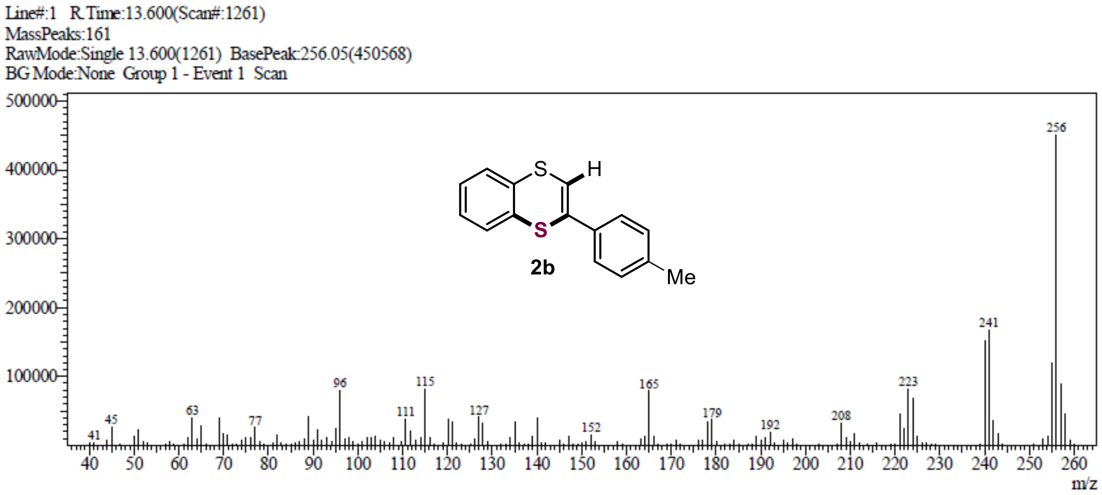

Figure S7. Mass spectrum of **2b**.

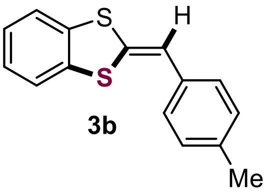

3b

2-(4-methylbenzylidene)benzo[*d*][1,3]dithiole (**3b**). Following ***General Procedure D***, the product was isolated as a white solid in 77% yield (39.5 mg) using hexane as eluent. mp 134–136 ºC. **^{1}H NMR** (400 MHz, $CDCl_3$): δ 7.27 – 7.16 (m, 6H), 7.13 – 7.06 (m, 2H), 6.53 (s, 1H), 2.35 (s, 3H). **^{13}C{^{1}H} NMR** (100 MHz, $CDCl_3$): δ 136.4, 135.8, 134.8, 133.8, 131.1, 129.2, 126.9, 125.9, 125.5, 121.6, 120.9, 114.8, 21.2. **HRMS** (ESI) *m*/*z*: $[M]^+$ calculated for $C_{15}H_{12}S_2$, 256.0375; found, 256.0374.

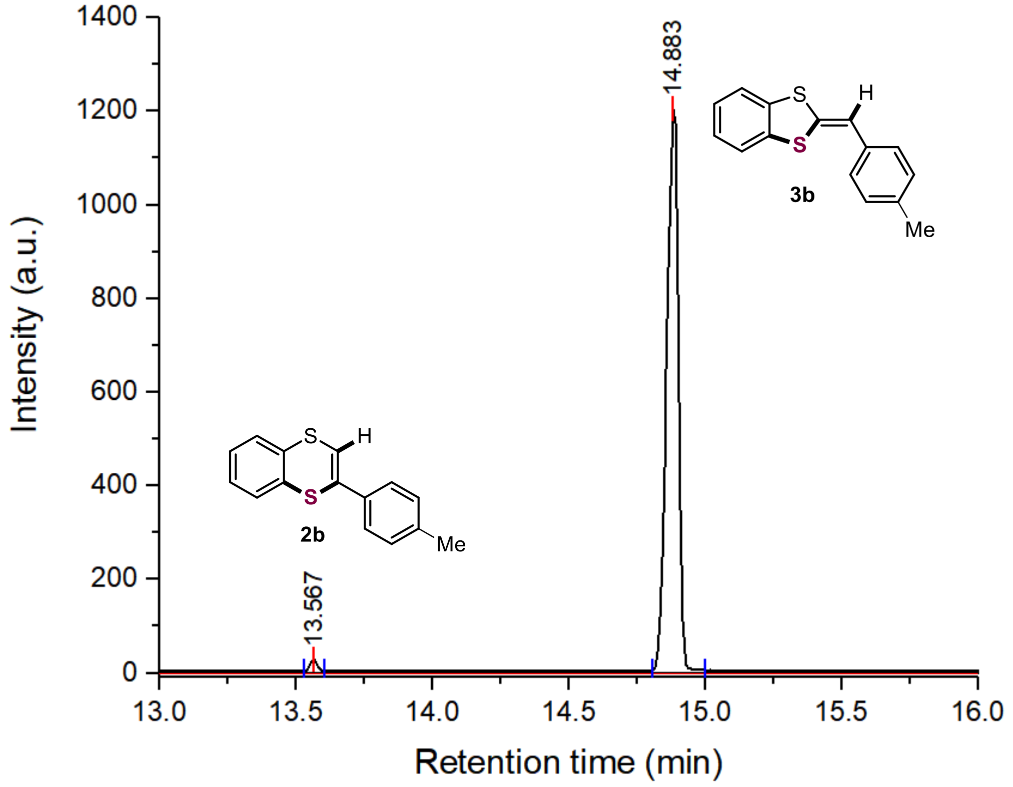

Figure S8. GC-MS expanded chromatogram of the crude reaction mixture for the synthesis of **3b**.

Peak#	Retention time (min)	Area	Area %	Height	Height %
**2b**	13.567	991	1.63	26	2.12
**3b**	14.883	59807	98.37	1201	97.88
		60798	100	1227	100

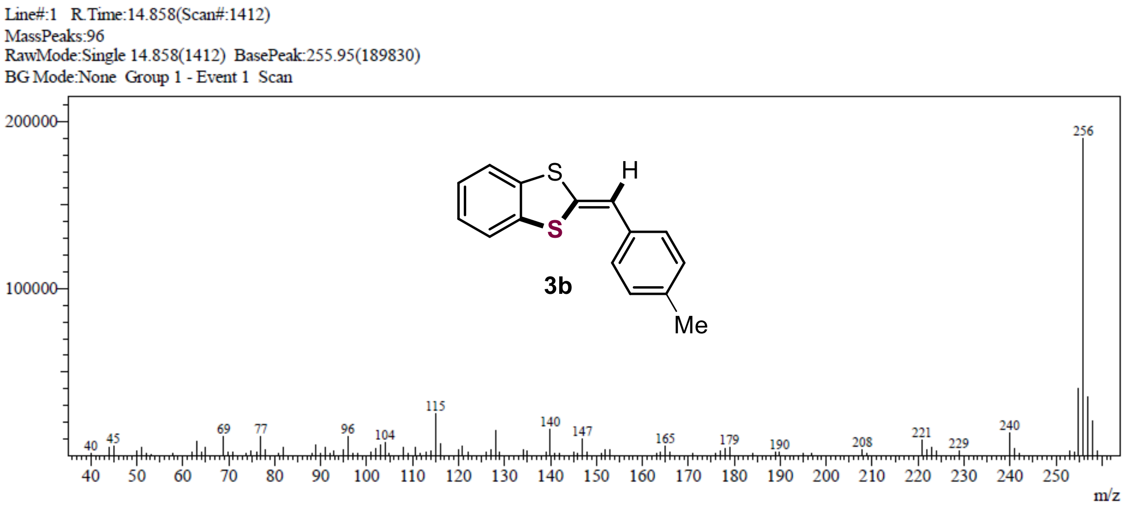

Figure S9. Mass spectrum of **3b**.

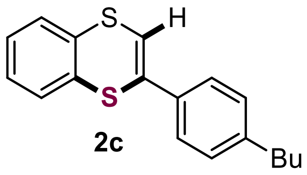

2c

2-(4-butylphenyl)benzo[*b*][1,4]dithiine (**2c**). Following ***General Procedure C**,* the product was isolated as a light-yellow oil in 75% yield (45 mg) using hexane as eluent. **^{1}H NMR** (400 MHz, $CDCl_3$): δ 7.52 (d, *J* = 8.3 Hz, 2H), 7.46 – 7.41 (m, 1H), 7.39 – 7.34 (m, 1H), 7.26 – 7.21 (m, 2H), 7.16 (d, *J* = 8.3 Hz, 2H), 6.62 (s, 1H), 2.65 – 2.55 (m, 2H), 1.64 – 1.53 (m, 2H), 1.35 (sext., *J* = 7.3 Hz, 2H), 0.92 (t, *J* = 7.3 Hz, 3H). **^{13}C{^{1}H} NMR** (100 MHz, $CDCl_3$): δ 143.7, 140.7, 135.1, 134.5, 134.4, 128.62, 128.56, 127.84, 127.76, 127.5, 126.8, 117.1, 35.3, 33.4, 22.3, 13.9. **HRMS** (ESI) *m*/*z*: $[M]^+$ calculated for $C_{18}H_{18}S_2$ 298.0844; found 298.0847.

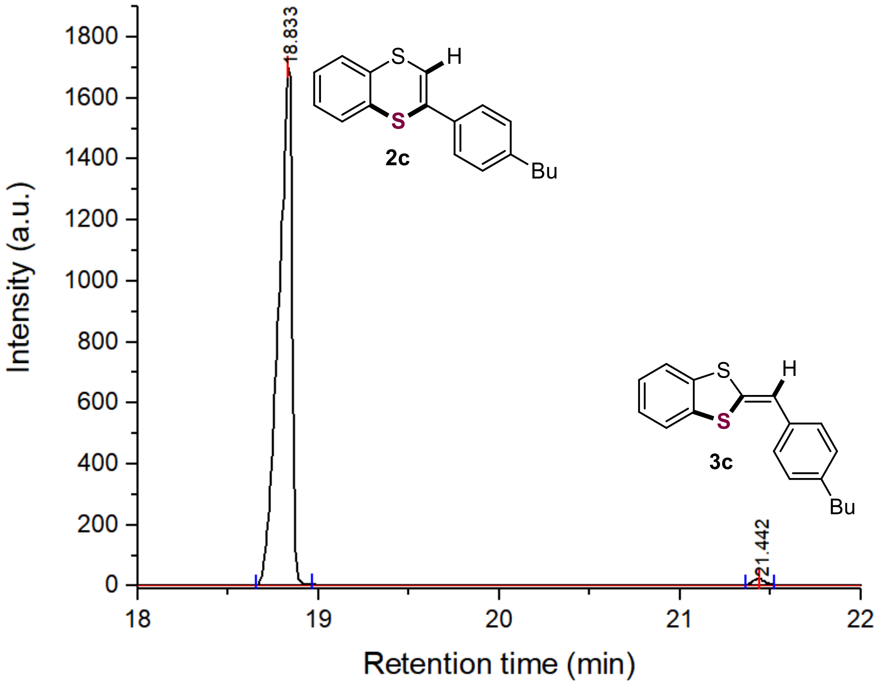

Figure S10. GC-MS expanded chromatogram of the crude reaction mixture for the synthesis of **2c**.

Peak#	Retention time (min)	Area	Area %	Height	Height %
**2c**	18.833	149634	98.66	1698	98.38
**3c**	21.442	2037	1.34	28	1.62
		151671	100	1726	100

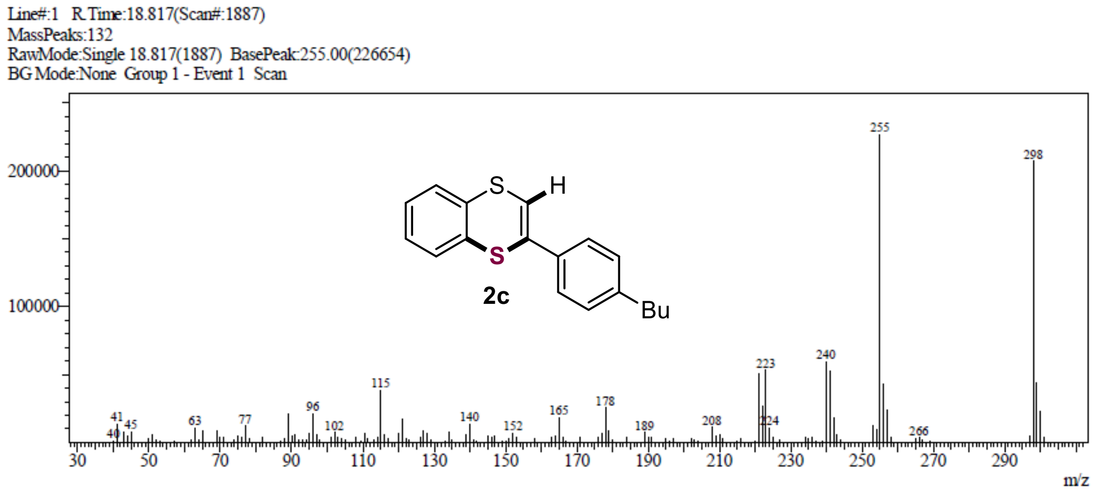

Figure S11. Mass spectrum of **2c**.

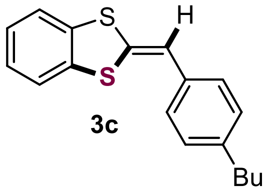

3c

2-(4-butylbenzylidene)benzo[*d*][1,3]dithiole (**3c**). Following ***General Procedure D***, the product was isolated as a white solid in 75% yield (45 mg) using hexane as eluent. mp 96–98 ºC. **^{1}H NMR** (400 MHz, $CDCl_3$): δ 7.28 – 7.17 (m, 6H), 7.14 – 7.07 (m, 2H), 6.54 (s, 1H), 2.68 – 2.55 (m, 2H), 1.66 – 1.55 (m, 2H), 1.37 (sext., *J* = 7.3 Hz, 2H), 0.93 (t, *J* = 7.3 Hz, 3H). **^{13}C{^{1}H} NMR** (100 MHz, $CDCl_3$): δ 140.9, 136.5, 134.8, 134.0, 131.0, 128.5, 126.9, 125.9, 125.5, 121.6, 120.9, 114.8, 35.4, 33.5, 22.3, 13.9. **HRMS** (ESI) *m*/*z*: $[M]^+$ calculated for $C_{18}H_{18}S_2$ 298.0844; found 298.0832.

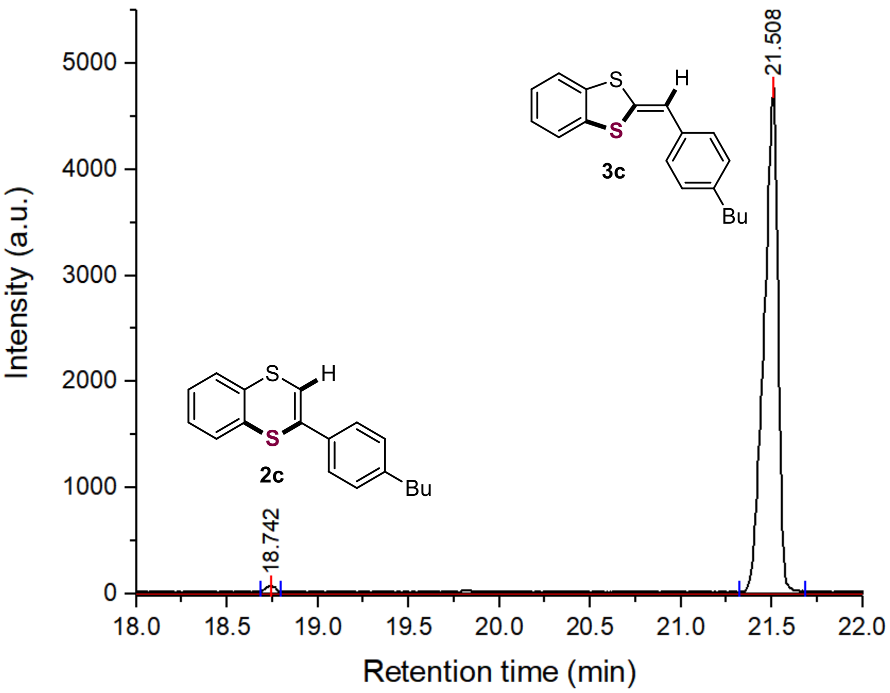

Figure S12. GC-MS expanded chromatogram of the crude reaction mixture for the synthesis of **3c**.

Peak#	Retention time (min)	Area	Area %	Height	Height %
**2c**	18.742	537	1.23	73	1.52
**3c**	21.508	43208	98.77	4718	98.48
		43745	100	4791	100

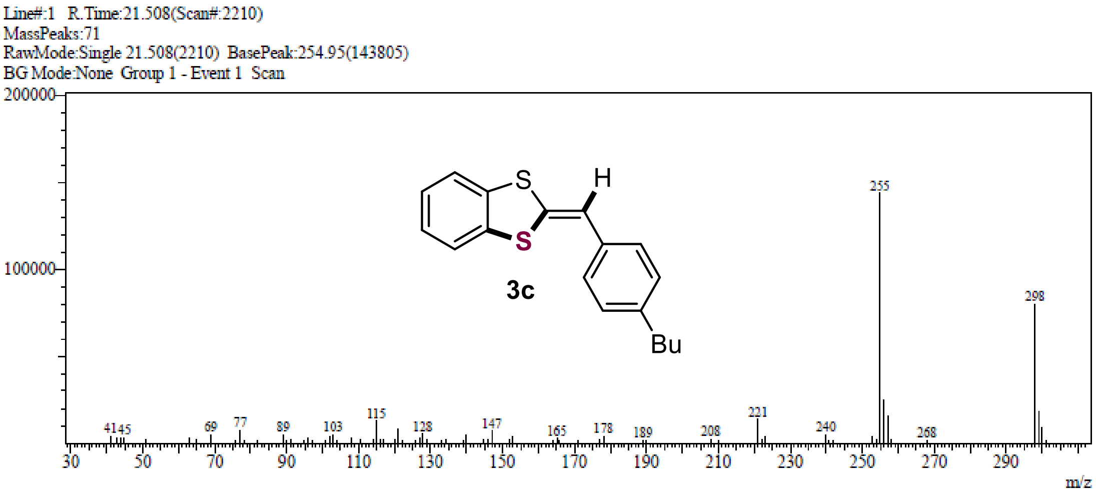

Figure S13. Mass spectrum of **3c**.

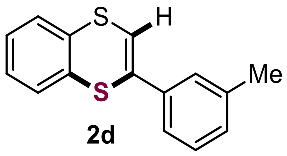

2d

2-(*m*-tolyl)benzo[*b*][1,4]dithiine (**2d**). Following ***General Procedure C***, the product was isolated as a light-yellow oil in 75% yield (38.5 mg) using hexane as eluent. **^{1}H NMR** (400 MHz, $CDCl_3$): δ 7.48 – 7.34 (m, 4H), 7.29 – 7.20 (m, 3H), 7.14 (d, *J* = 7.5 Hz, 1H), 6.65 (s, 1H), 2.37 (s, 3H). **^{13}C{^{1}H} NMR** (100 MHz, $CDCl_3$): δ 140.6, 138.3, 137.2, 135.0, 134.3, 129.4, 128.6, 128.5, 127.8, 127.8, 127.62, 127.59, 124.1, 118.0, 21.4. **HRMS** (ESI) *m*/*z*: $[M + H]^+$ calculated for $C_{15}H_{13}S_2$ 257.0453; found, 257.0453.

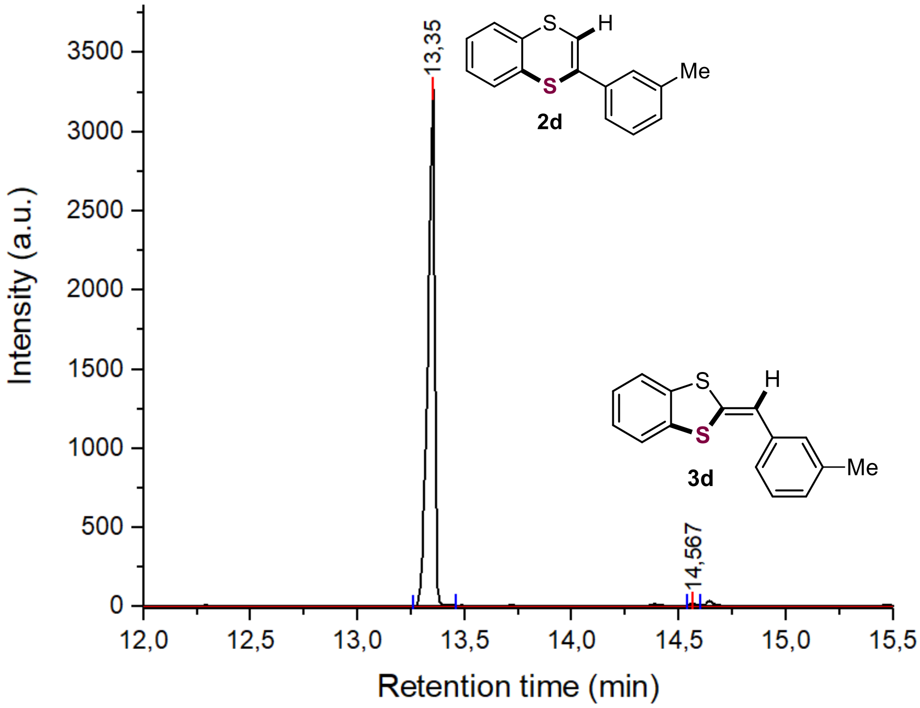

Figure S14. GC-MS expanded chromatogram of the crude reaction mixture for the synthesis of **2d**.

Peak#	Retention time (min)	Area	Area %	Height	Height %
**2d**	13.350	121740	99.39	3227	99.08
**3d**	14.567	752	0.61	30	0.92
		122492	100	3257	100

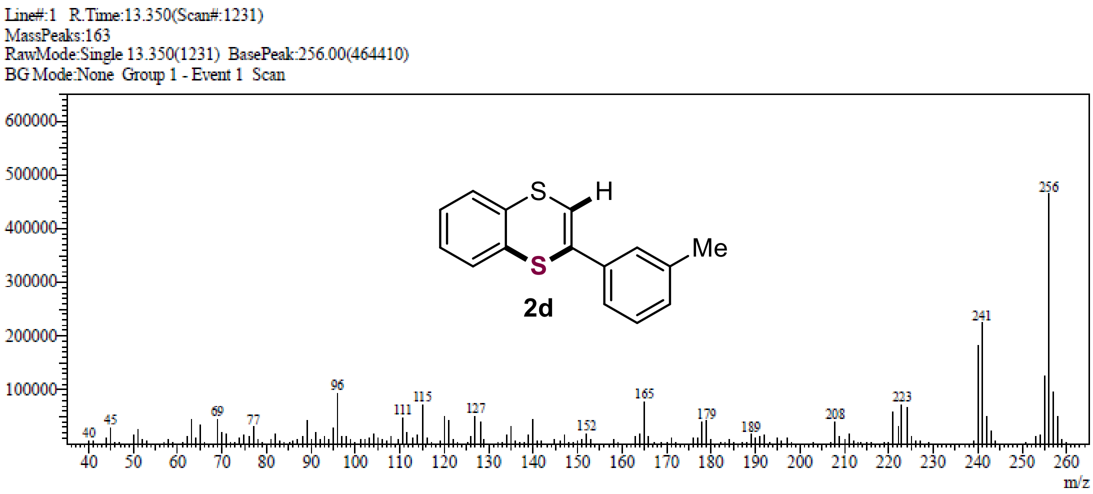

Figure S15. Mass spectrum of **2d**.

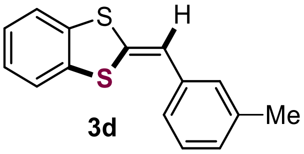

3d

2-(3-methylbenzylidene)benzo[*d*][1,3]dithiole (**3d**). Following ***General Procedure D***, the product was isolated as a white solid in 70% yield (36 mg) using hexane as eluent. mp 102–104 °C. **^{1}H NMR** (400 MHz, $CDCl_3$): δ 7.32 – 7.20 (m, 3H), 7.20 – 7.07 (m, 4H), 7.01 (d, *J* = 7.4 Hz, 1H), 6.53 (s, 1H), 2.38 (s, 3H). **^{13}C{^{1}H} NMR** (100 MHz, $CDCl_3$): δ 138.1, 136.5, 136.4, 134.7, 132.1, 128.4, 127.7, 126.9, 125.9, 125.5, 124.1, 121.6, 120.9, 114.8, 21.5. **HRMS** (ESI) *m*/*z*: $[2M-2H]^+$ calculated for $C_{30}H_{22}S_4$, 510.0599; found, 510.0598. *<u>Note</u>: the observed ion corresponds to the dimer of **3d**, indicating dimerization during the HRMS analysis.*

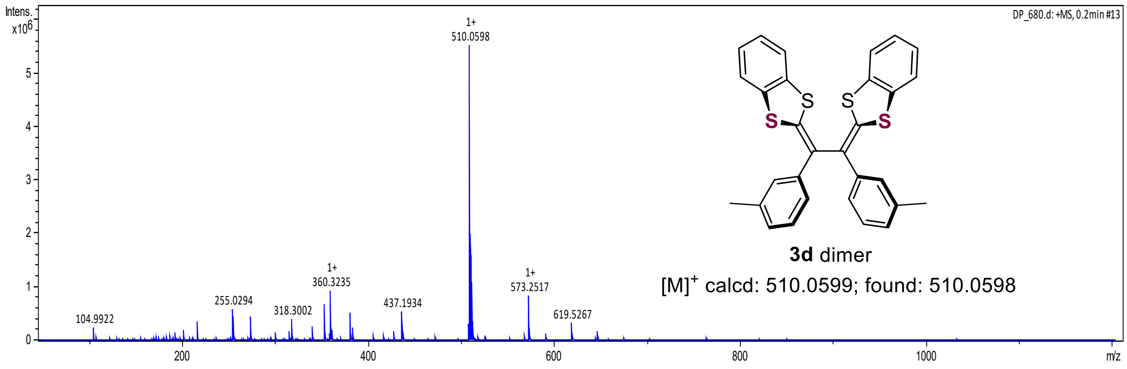

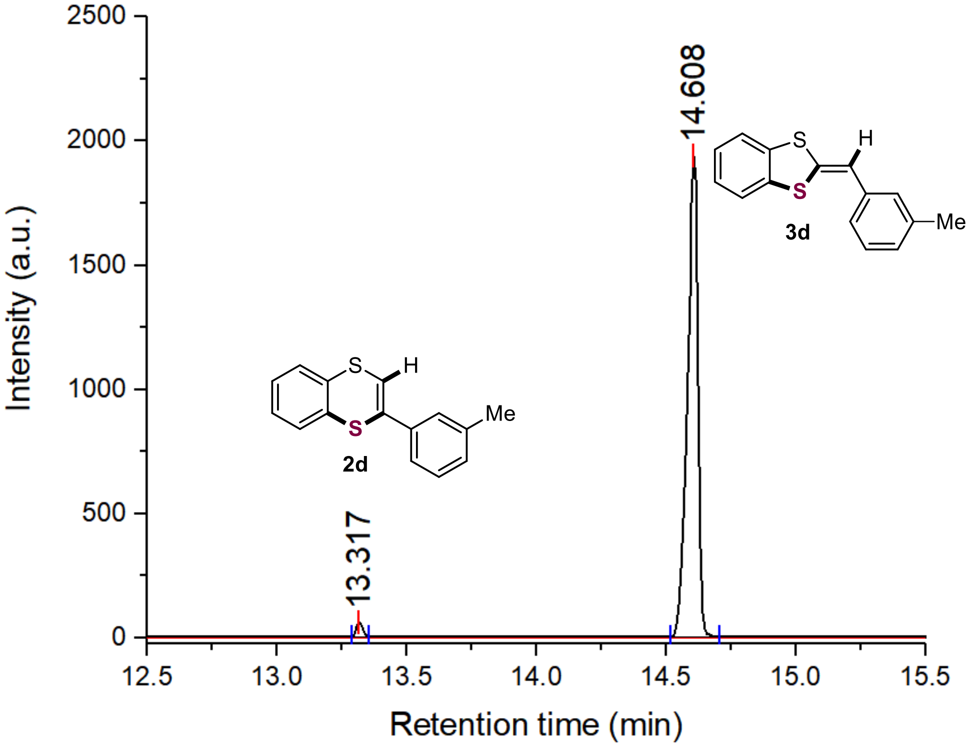

Figure S16. GC-MS expanded chromatogram of the crude reaction mixture for the synthesis of **3d**.

Peak#	Retention time (min)	Area	Area %	Height	Height %
**2d**	13.317	1927	2.01	64	3.2
**3d**	14.608	93732	97.98	1936	96.8
		95659	100	2000	100

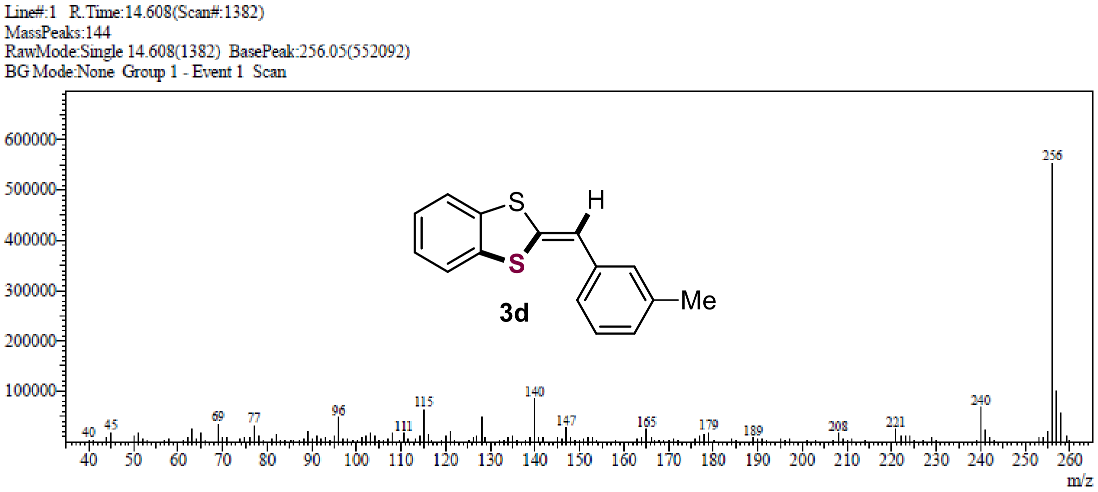

Figure S17. Mass spectrum of **3d**.

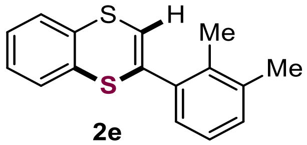

2e

2-(2,3-dimethylphenyl)benzo[*b*][1,4]dithiine (**2e**). Following ***General Procedure C***, the product was isolated as a colorless oil in 58% yield (31.5 mg) using hexane as eluent. **^{1}H NMR** (400 MHz, $CDCl_3$) δ 7.43 – 7.32 (m, 2H), 7.29 – 7.20 (m, 2H), 7.17 – 7.02 (m, 3H), 6.33 (s, 1H), 2.28 (s, 3H), 2.19 (s, 3H). **^{13}C{^{1}H} NMR** (100 MHz, $CDCl_3$): δ 140.6, 138.0, 137.4, 135.0, 134.9, 134.5, 130.1, 128.3, 128.0, 127.7, 127.65, 127.60, 125.4, 120.4, 20.4, 16.9. **HRMS** (ESI) *m*/*z*: $[M+H]^+$ calculated for $C_{16}H_{15}S_2$, 271.0610; found, 271.0604.

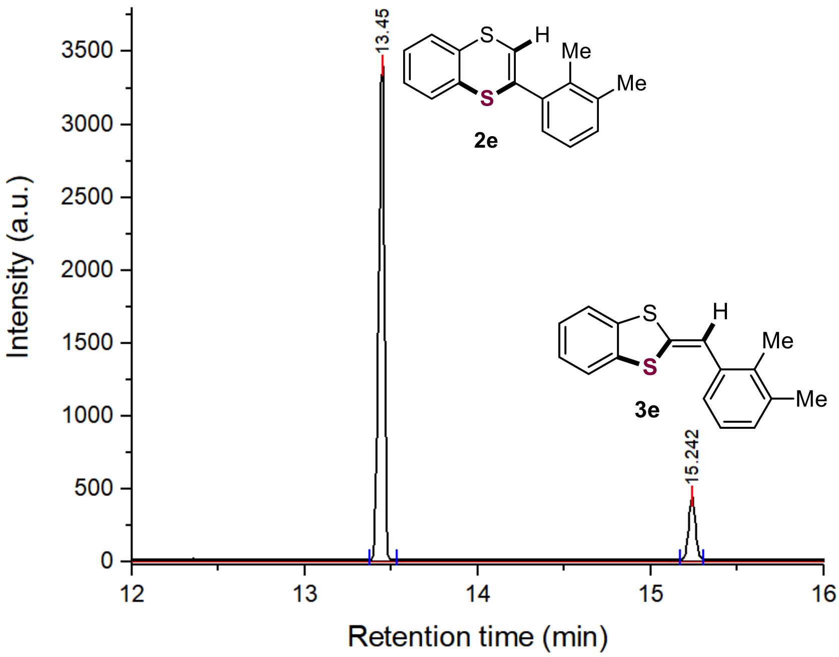

Figure S18. GC-MS expanded chromatogram of the crude reaction mixture for the synthesis of **2e**.

Peak#	Retention time (min)	Area	Area %	Height	Height %
**2e**	13.450	127825	85.62	3390	88.28
**3e**	15.242	21466	14.38	450	11.72
		149291	100	3840	100

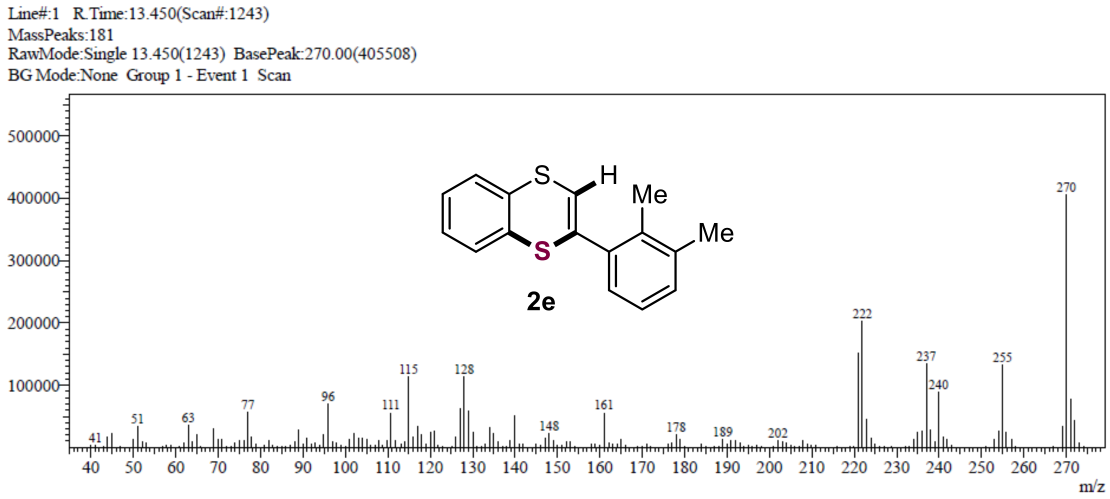

Figure S19. Mass spectrum of **2e**.

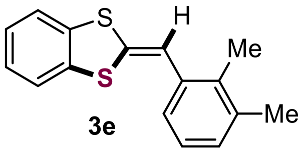

3e

2-(2,3-dimethylbenzylidene)benzo[*d*][1,3]dithiole (**3e**). Following ***General Procedure D***, the product was isolated as a light-yellow oil in 62% yield (33.5 mg) using hexane as eluent. ^{1}**H NMR** (400 MHz, $CDCl_3$) δ 7.30 (d, *J* = 7.6 Hz, 1H), 7.23 – 7.16 (m, 1H), 7.17 – 7.11 (m, 2H), 7.09 – 7.03 (m, 3H), 6.61 (s, 1H), 2.29 (s, 3H), 2.20 (s, 3H). ^{13}**C{**^{1}**H} NMR** (100 MHz, $CDCl_3$): δ 136.9, 136.3, 136.0, 135.2, 134.0, 133.5, 128.9, 125.6, 125.5, 125.4, 124.7, 121.5, 121.0, 114.7, 20.6, 15.7. **HRMS** (ESI) *m*/*z*: $[M+H]^+$ calculated for $C_{16}H_{15}S_2$, 271.0610; found, 271.0614.

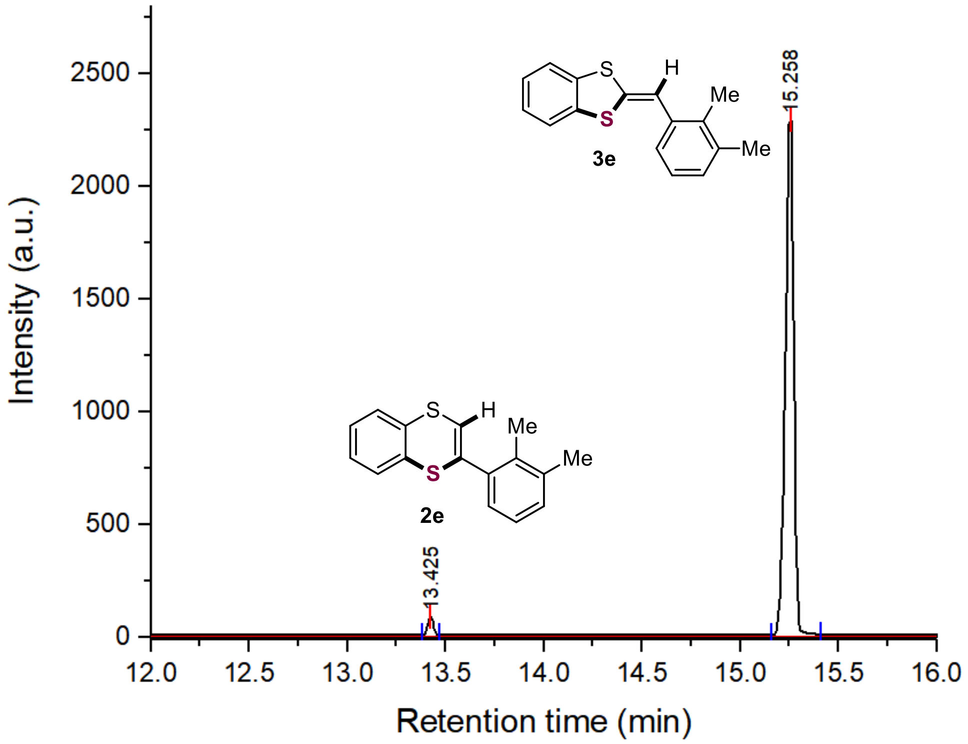

Figure S20. GC-MS expanded chromatogram of the crude reaction mixture for the synthesis of **3e**.

Peak#	Retention time (min)	Area	Area %	Height	Height %
**2e**	13.425	3177	2.67	96	4.02
**3e**	15.258	115843	97.33	2291	95.97
		119020	100	2387	100

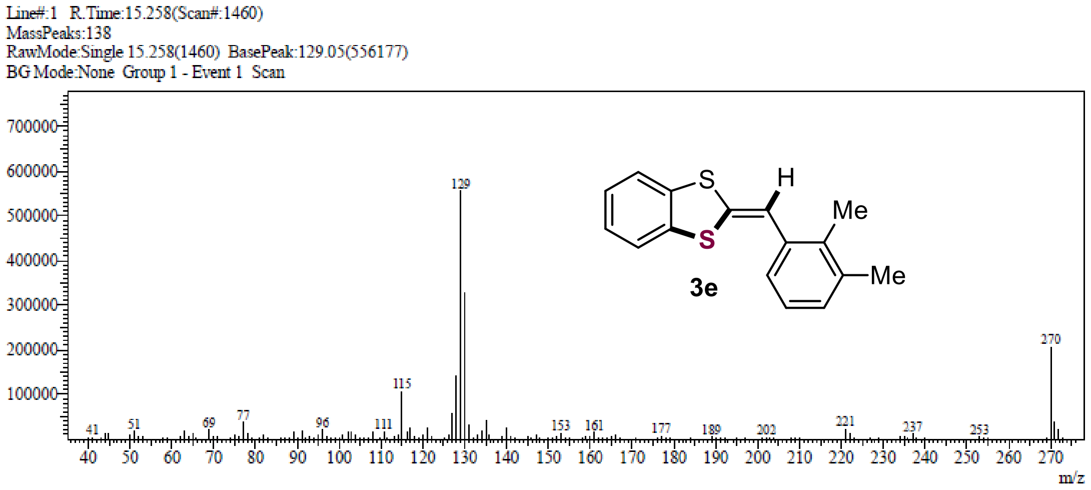

Figure S21. Mass spectrum of **3e**.

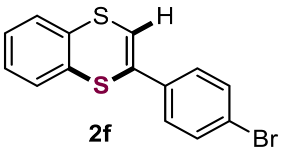

2f

2-(4-bromophenyl)benzo[*b*][1,4]dithiine (**2f**). Following ***General Procedure C***, the product was isolated as a light-yellow solid in 65% yield (42 mg) using hexane as eluent. mp 84–86 ºC. **^{1}H NMR** (400 MHz, $CDCl_3$) δ 7.51 – 7.45 (m, 4H), 7.45 – 7.42 (m, 1H), 7.37 (dd, J = 5.8, 3.3 Hz, 1H), 7.27 – 7.22 (m, 2H), 6.67 (s, 1H). **^{13}C{^{1}H} NMR** (100 MHz, $CDCl_3$): δ 139.3, 136.1, 134.7, 133.9, 131.7, 128.6, 128.5, 128.0, 127.9, 127.7, 122.7, 119.1. **HRMS** (ESI) *m*/*z*: $[M]^+$ calculated for $C_{14}H_9BrS_2$, 319.9324; found, 319.9317.

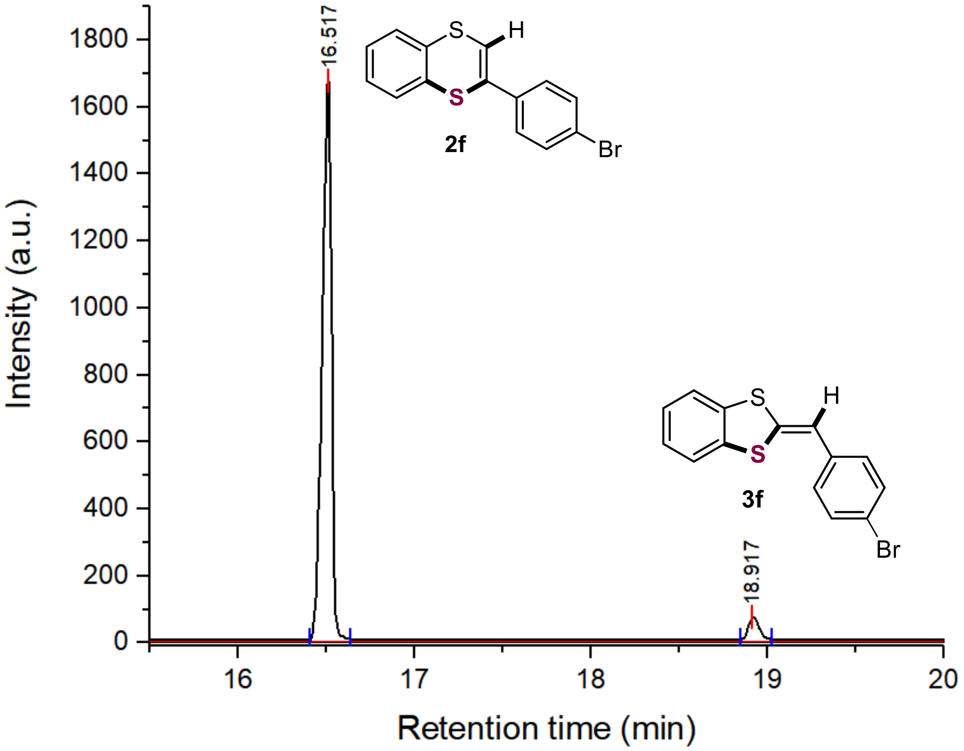

Figure S22. GC-MS expanded chromatogram of the crude reaction mixture for the synthesis of **2f**.

Peak#	Retention time (min)	Area	Area %	Height	Height %
**2f**	16.517	104334	95.18	1674	95.66
**3f**	18.917	5279	4.82	76	4.34
		109613	100	1750	100

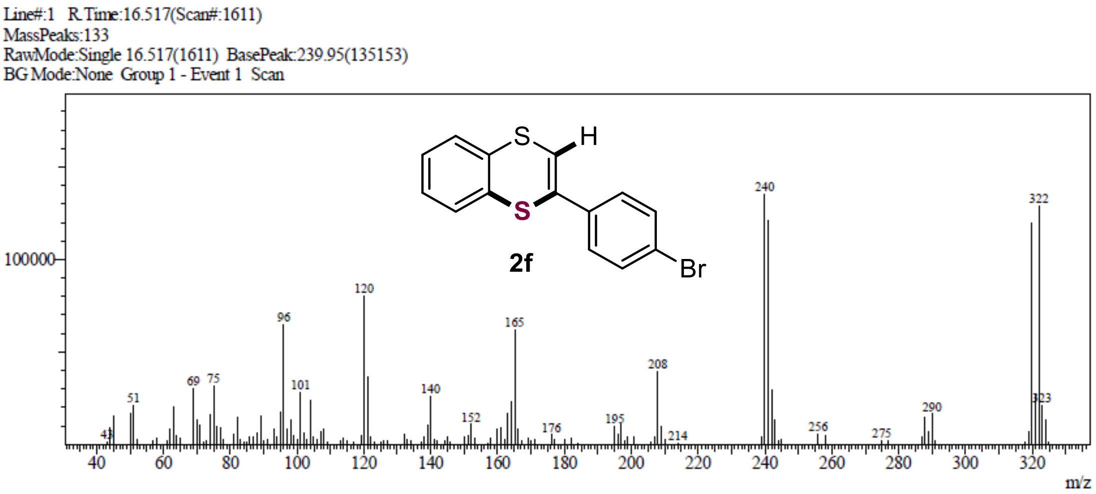

Figure S23. Mass spectrum of **2f**.

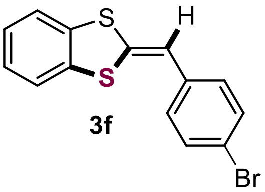

3f

2-(4-bromobenzylidene)benzo[*d*][1,3]dithiole (**3f**). Following ***General Procedure D***, the product was isolated as a light-yellow solid in 55% yield (35.5 mg) using hexane as eluent. mp 168–170 ºC. **^{1}H NMR** (400 MHz, $CDCl_3$): δ 7.48 (d, *J* = 8.5 Hz, 2H), 7.28 – 7.22 (m, 2H), 7.20 (d, *J* = 8.5 Hz, 2H), 7.16 – 7.09 (m, 2H), 6.48 (s, 1H). **^{13}C{^{1}H} NMR** (100 MHz, $CDCl_3$): δ 136.0, 135.4, 134.6, 133.8, 131.6, 128.4, 126.1, 125.7, 121.8, 121.0, 119.4, 113.2. **HRMS** (ESI) *m*/*z*: $[M]^+$ calculated for $C_{14}H_9BrS_2$, 319.9324; found, 319.9313.

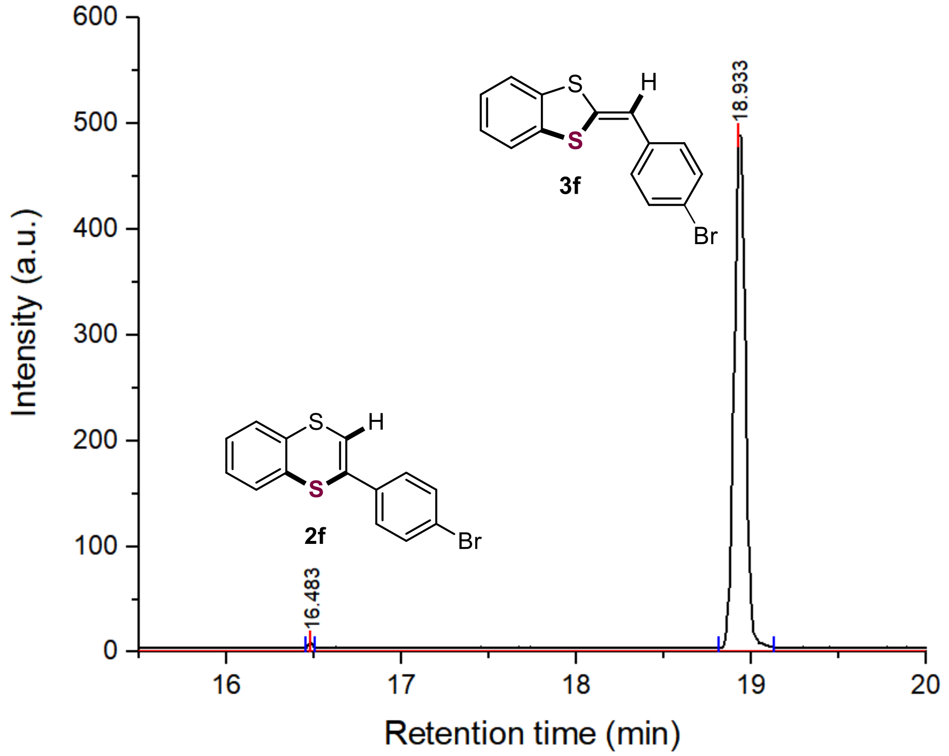

Figure S24. GC-MS expanded chromatogram of the crude reaction mixture for the synthesis of **3f**.

Peak#	Retention time (min)	Area	Area %	Height	Height %
**2f**	16.483	235	0.64	8	1.62
**3f**	18.933	36546	99.36	487	98.38
		36781	100	495	100

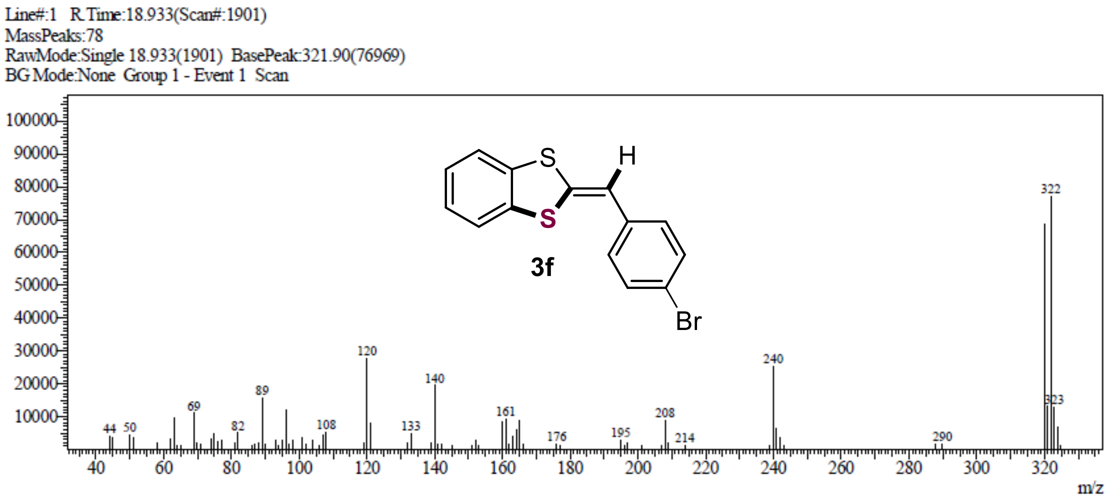

Figure S25. Mass spectrum of **3f**.

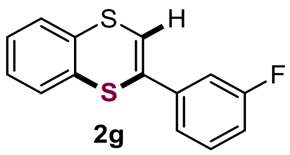

2g

2-(3-fluorophenyl)benzo[*b*][1,4]dithiine (**2g**). Following ***General Procedure C***, the product was isolated as a light-yellow oil in 58% yield (30 mg) using hexane as eluent. **^{1}H NMR** (400 MHz, $CDCl_3$) δ 7.47 – 7.42 (m, 1H), 7.42 – 7.29 (m, 4H), 7.28 – 7.22 (m, 2H), 7.06 – 6.98 (m, 1H), 6.72 (s, 1H). **^{13}C{^{1}H} NMR** (100 MHz, $CDCl_3$): δ 162.8 (d, $^1J_{C\text{-}F}$ = 246.4 Hz), 139.3 (d, $^3J_{C\text{-}F}$ = 7.8 Hz), 139.1 (d, $^4J_{C\text{-}F}$ = 2.5 Hz), 134.7, 133.8, 130.1 (d, $^3J_{C\text{-}F}$ = 8.4 Hz), 128.6, 128.0, 127.9, 127.8, 122.6 (d, $^4J_{C\text{-}F}$ = 2.9 Hz), 119.8, 115.5 (d, $^2J_{C\text{-}F}$ = 21.3 Hz), 113.9 (d, $^2J_{C\text{-}F}$ = 23.1 Hz). **HRMS** (ESI) *m*/*z*: $[M]^+$ calculated for $C_{14}H_9FS_2$, 260.0124; found, 260.0123.

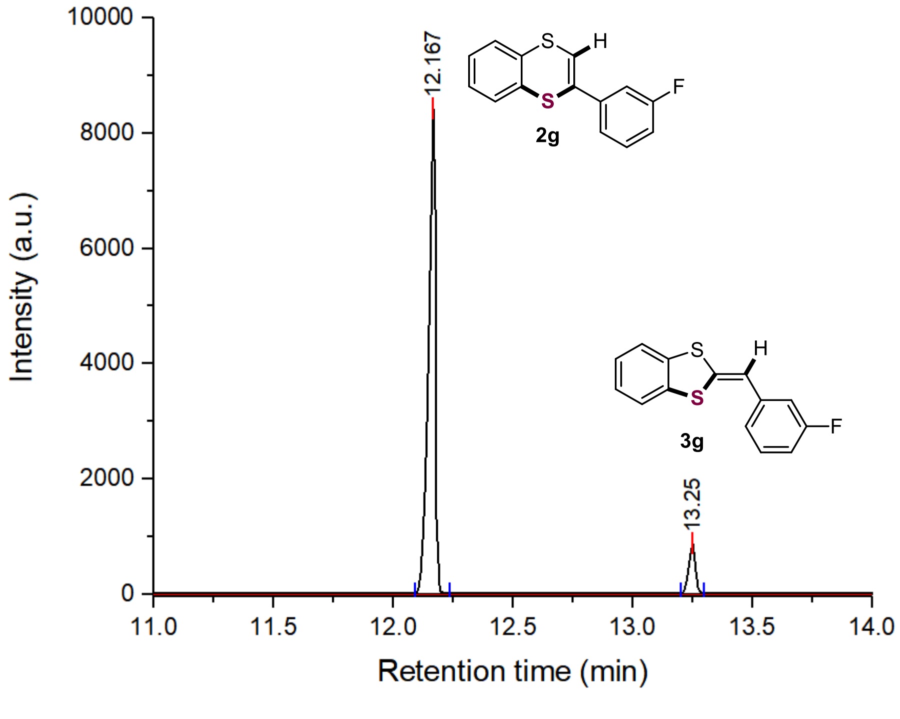

Figure S26. GC-MS expanded chromatogram of the crude reaction mixture for the synthesis of **2g**.

Peak#	Retention time (min)	Area	Area %	Height	Height %
**2g**	12.167	283612	90.31	8422	90.70
**3g**	13.250	30433	9.69	863	9.30
		314045	100	9285	100

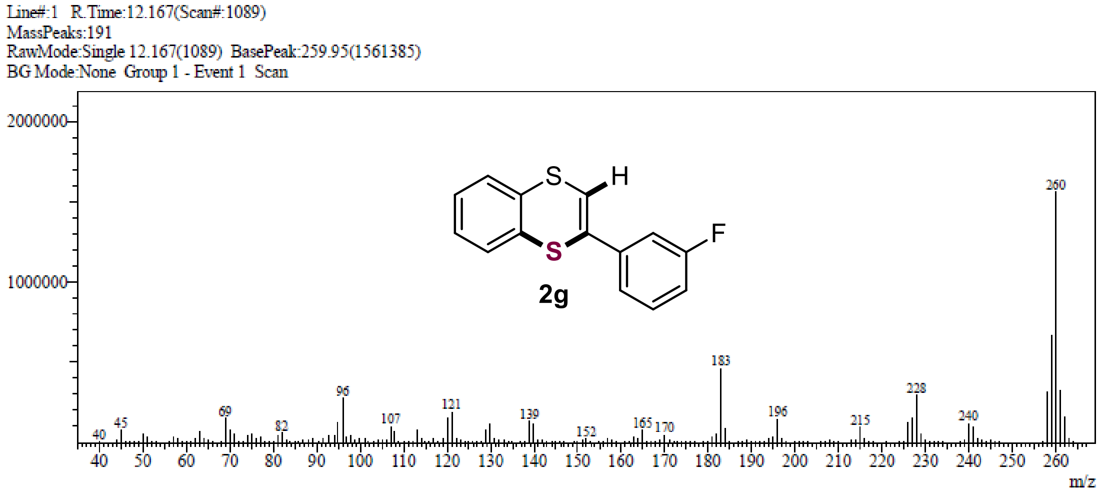

Figure S27. Mass spectrum of **2g**.

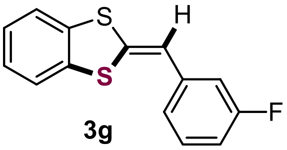

3g

2-(3-fluorobenzylidene)benzo[*d*][1,3]dithiole (**3g**). Following ***General Procedure D***, the product was isolated as a light-yellow solid in 65% yield (34 mg) using hexane as eluent. mp 110–112 ºC. **^{1}H NMR** (400 MHz, $CDCl_3$): δ 7.39 – 7.24 (m, 3H), 7.20 – 7.05 (m, 4H), 6.95 – 6.87 (m, 1H), 6.55 (s, 1H). **^{13}C{^{1}H} NMR** (100 MHz, $CDCl_3$): δ 162.9 (d, $^{1}J_{C-F}$ = 245.1 Hz), 138.6 (d, $^{3}J_{C-F}$ = 8.1 Hz), 136.1, 134.6, 134.5, 129.9 (d, $^{3}J_{C-F}$ = 8.7 Hz), 126.1, 125.7, 122.7 (d, $^{4}J_{C-F}$ = 2.7 Hz), 121.8, 121.0, 113.3 (d, $^{2}J_{C-F}$ = 22.5 Hz), 113.2 (d, $^{4}J_{C-F}$ = 2.7 Hz), 112.7 (d, $^{2}J_{C-F}$ = 21.4 Hz). **HRMS** (ESI) *m*/*z*: $[M]^+$ calculated for $C_{14}H_9FS_2$, 260.0124; found, 260.0115.

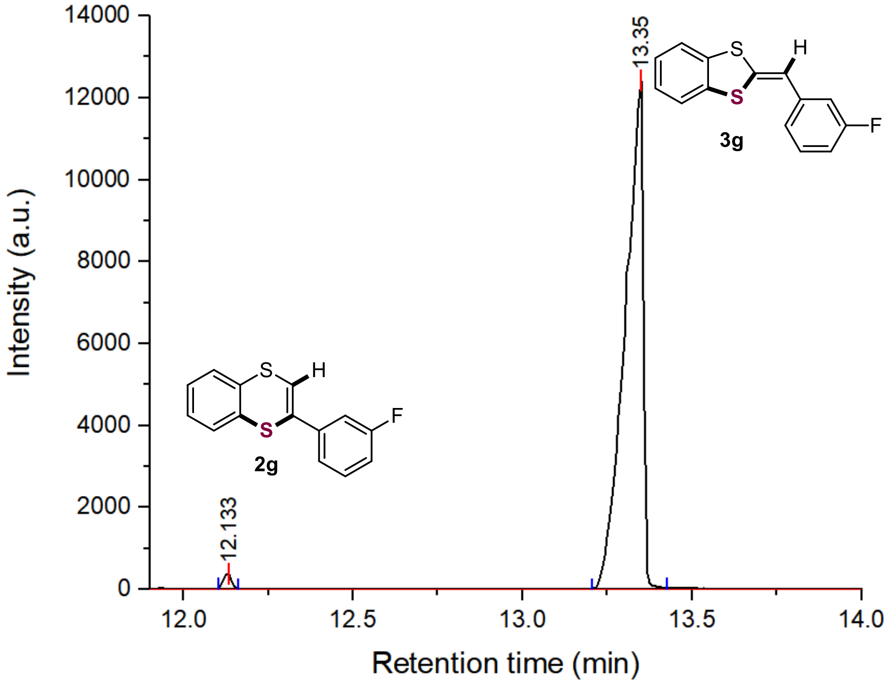

Figure S28. GC-MS expanded chromatogram of the crude reaction mixture for the synthesis of **3g**.

Peak#	Retention time (min)	Area	Area %	Height	Height %
**2g**	12.133	10448	1.36	382	3.00
**3g**	13.350	756239	98.64	12343	97.00
		766687	100	12725	100

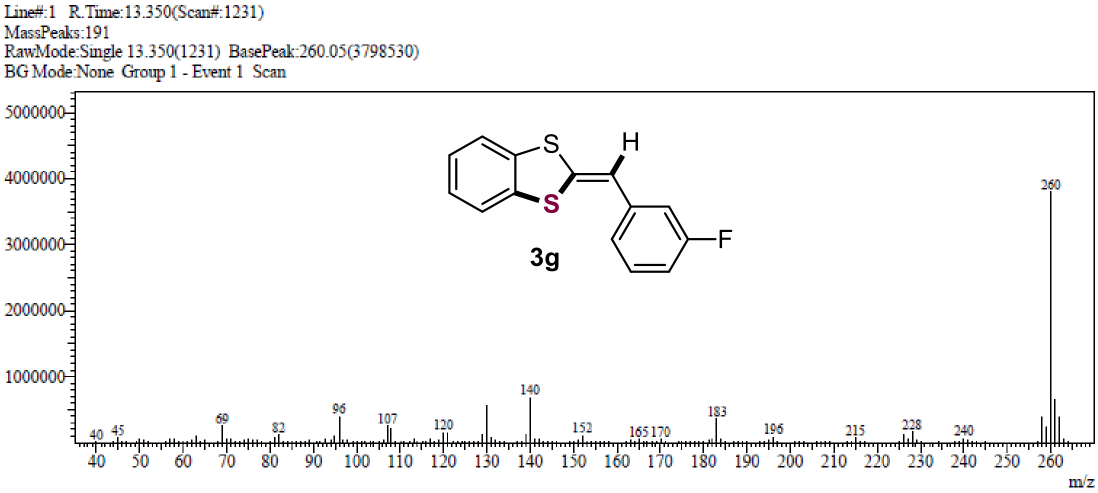

Figure S29. Mass spectrum of **3g**.

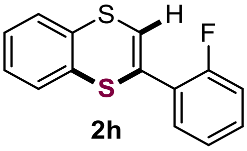

2-(2-fluorophenyl)benzo[*b*][1,4]dithiine (**2h**). Following ***General Procedure C***, the product was isolated as a light-yellow solid in 63% yield (33 mg) using hexane as eluent. mp 98–100 ºC. **^{1}H NMR** (400 MHz, $CDCl_3$) δ 7.58 (t, *J* = 7.7 Hz, 1H), 7.47 – 7.36 (m, 2H), 7.33 – 7.21 (m, 3H), 7.18 – 7.04 (m, 2H), 6.85 (s, 1H). **^{13}C{^{1}H} NMR** (100 MHz, $CDCl_3$): δ 160.0 (d, $^{1}J_{C\text{-}F}$ = 250.6 Hz),134.9, 134.3, 133.2 (d, $^{3}J_{C\text{-}F}$ = 2.6 Hz), 130.0 (d, $^{4}J_{C\text{-}F}$ = 2.5 Hz), 129.9 (d, $^{3}J_{C\text{-}F}$ = 8.4 Hz), 128.6, 127.9, 127.9, 127.7, 125.2 (d, $^{2}J_{C\text{-}F}$ = 12.6 Hz), 124.1 (d, $^{4}J_{C\text{-}F}$ = 3.7 Hz), 123.8 (d, $^{3}J_{C\text{-}F}$ = 7.0 Hz), 116.1 (d, $^{2}J_{C\text{-}F}$ = 22.5 Hz). **HRMS** (ESI) *m*/*z*: $[M]^+$ calculated for $C_{14}H_9FS_2$, 260.0124; found, 260.0125.

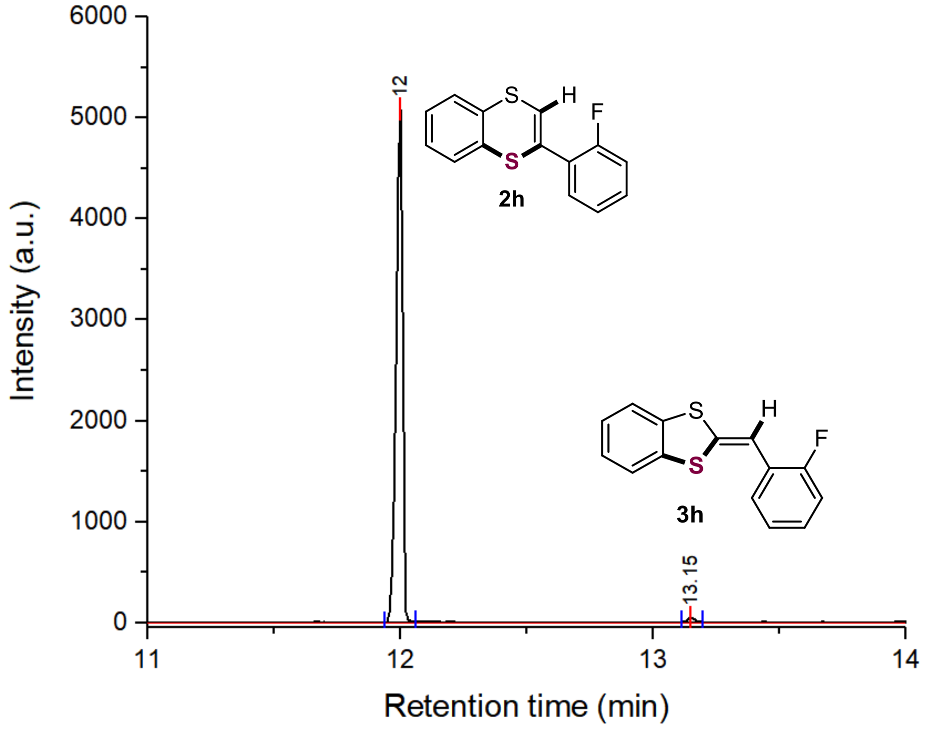

Figure S30. GC-MS expanded chromatogram of the crude reaction mixture for the synthesis of **2h**.

Peak#	Retention time (min)	Area	Area %	Height	Height %
**2h**	12.000	147436	98.77	5081	98.97
**3h**	13.150	1830	1.23	53	1.03

		149266	100	5134	100

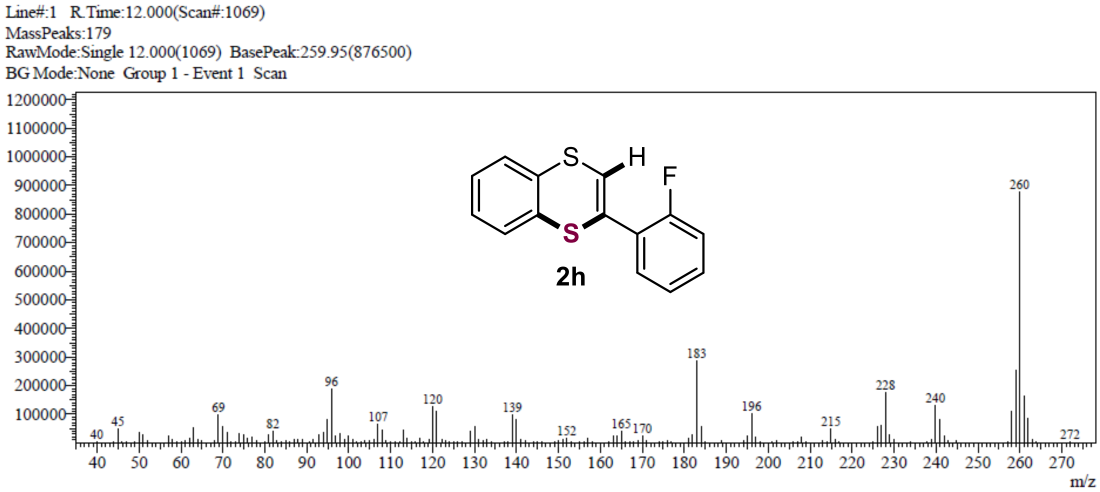

Figure S31. Mass spectrum of **2h**.

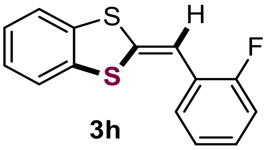

3h

2-(2-fluorobenzylidene)benzo[*d*][1,3]dithiole (**3h**). Following ***General Procedure D***, the product was isolated as a light-yellow solid in 65% yield (34 mg) using hexane as eluent. mp 82–84 ºC. **^{1}H NMR** (400 MHz, $CDCl_3$): δ 7.59 – 7.52 (m, 1H), 7.27 – 7.21 (m, 2H), 7.21 – 7.15 (m, 2H), 7.15 – 7.09 (m, 2H), 7.09 – 7.01 (m, 1H), 6.70 (s, 1H). **^{13}C{^{1}H} NMR** (100 MHz, $CDCl_3$): δ 158.9 (d, $^1J_{C\text{-}F}$ = 248.9 Hz), 136.0, 135.1 (d, $^4J_{C\text{-}F}$ = 1.5 Hz), 134.8, 127.4 (d, $^3J_{C\text{-}F}$ = 8.3 Hz), 126.9 (d, $^4J_{C\text{-}F}$ = 2.7 Hz), 126.0, 125.6, 124.7 (d, $^2J_{C\text{-}F}$ = 12.4 Hz), 123.9 (d, $^3J_{C\text{-}F}$ = 3.7 Hz), 121.7, 121.0, 115.2 (d, $^2J_{C\text{-}F}$ = 22.0 Hz), 106.1 (d, $^3J_{C\text{-}F}$ = 7.2 Hz). **HRMS** (ESI) *m*/*z*: $[M+H]^+$ calculated for $C_{14}H_{10}FS_2$, 261.0202; found, 261.0191.

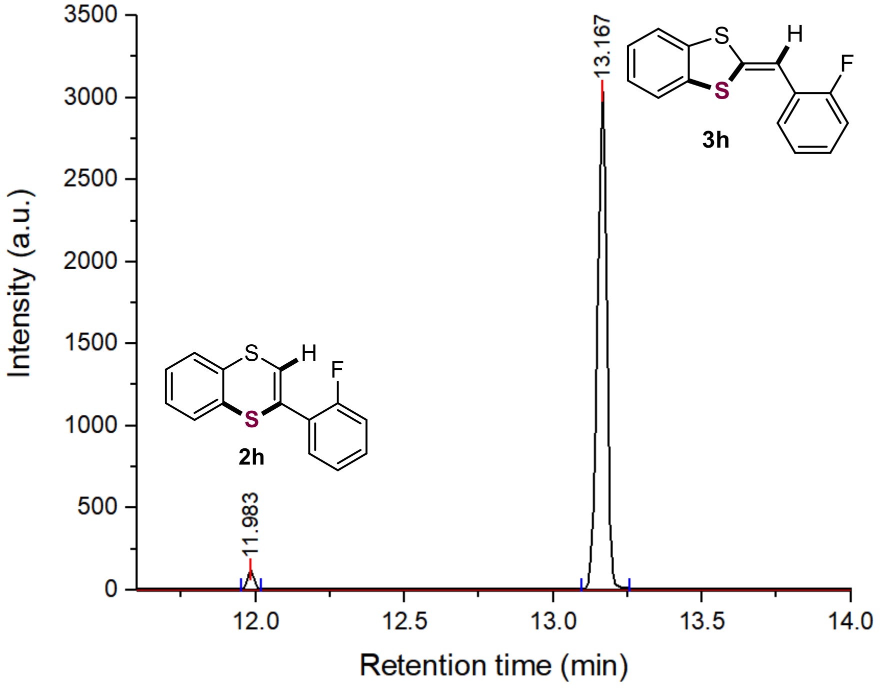

Figure S32. GC-MS expanded chromatogram of the crude reaction mixture for the synthesis of **3h**.

Peak#	Retention time (min)	Area	Area %	Height	Height %
**2h**	11.983	3143	2.72	121	3.83
**3h**	13.167	112582	97.28	3037	96.17
		115725	100	3158	100

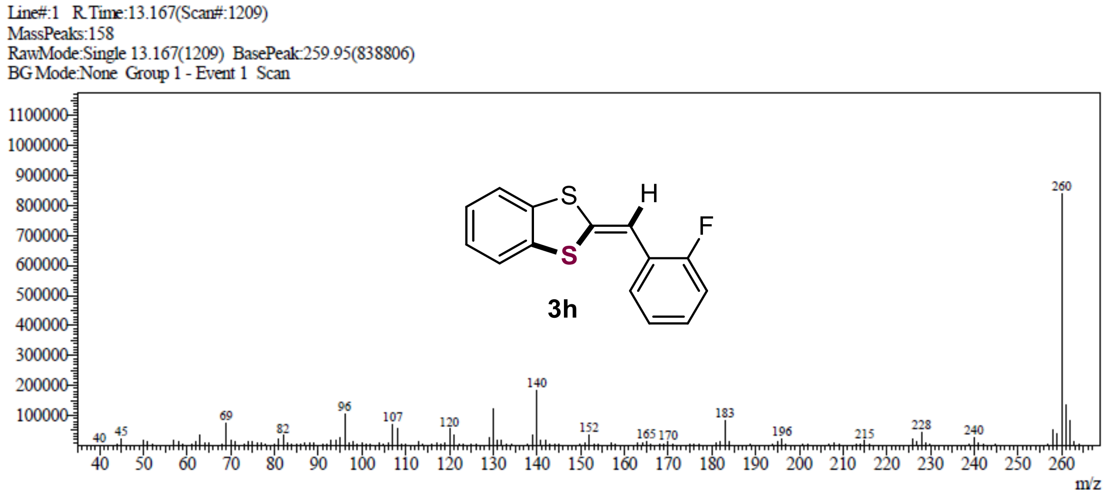

Figure S33. Mass spectrum of **3h**.

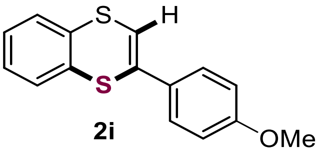

2-(4-methoxyphenyl)benzo[*b*][1,4]dithiine (**2i**). Following ***General Procedure C***, the product was isolated as a light-yellow solid in 61% yield (33.5 mg) using a gradient elution starting from hexane to hexane/ethyl acetate (95:5). mp 103–105 ºC. **^{1}H NMR** (400 MHz, $CDCl_3$) δ 7.55 (d, *J* = 8.9 Hz, 2H), 7.44 (dd, *J* = 5.8, 3.3 Hz, 1H), 7.36 (dd, *J* = 5.8, 3.2 Hz, 1H), 7.26 – 7.20 (m, 2H), 6.87 (d, *J* = 8.9 Hz, 2H), 6.53 (s, 1H), 3.81 (s, 3H). **^{13}C{^{1}H} NMR** (100 MHz, $CDCl_3$): δ 160.0, 140.6, 135.3, 134.5, 129.7, 128.5, 128.3, 127.9, 127.8, 127.5, 115.9, 113.9, 55.3. **HRMS** (ESI) *m*/*z*: $[M+H]^+$ calculated for $C_{15}H_{13}OS_2$, 273.0402; found, 273.0402.

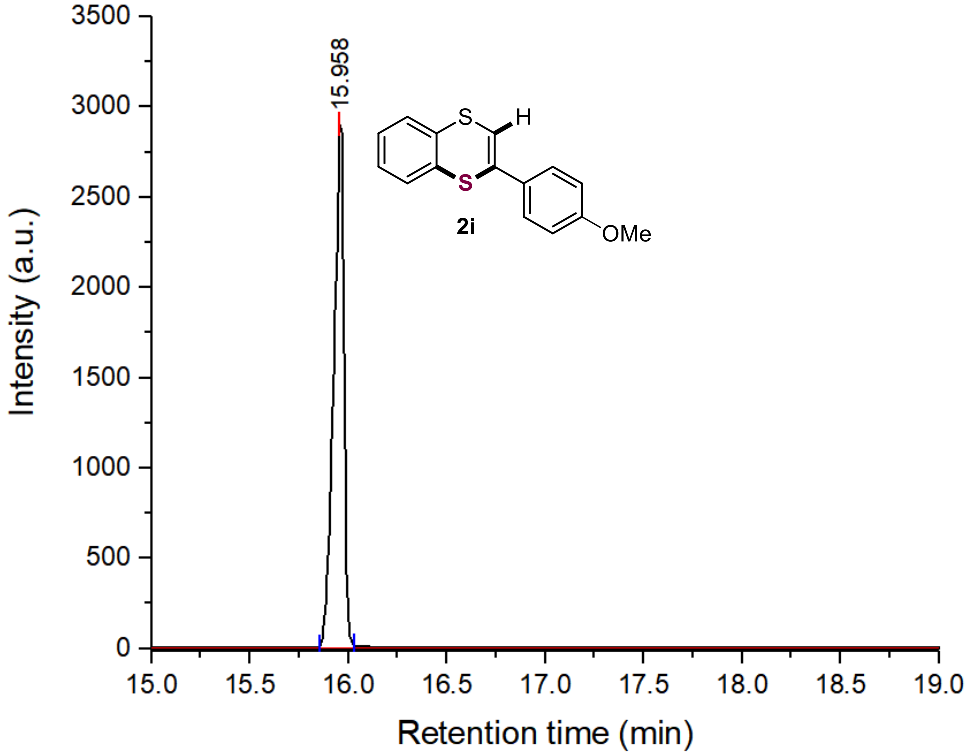

Figure S34. GC-MS expanded chromatogram of the crude reaction mixture for the synthesis of **2i**.

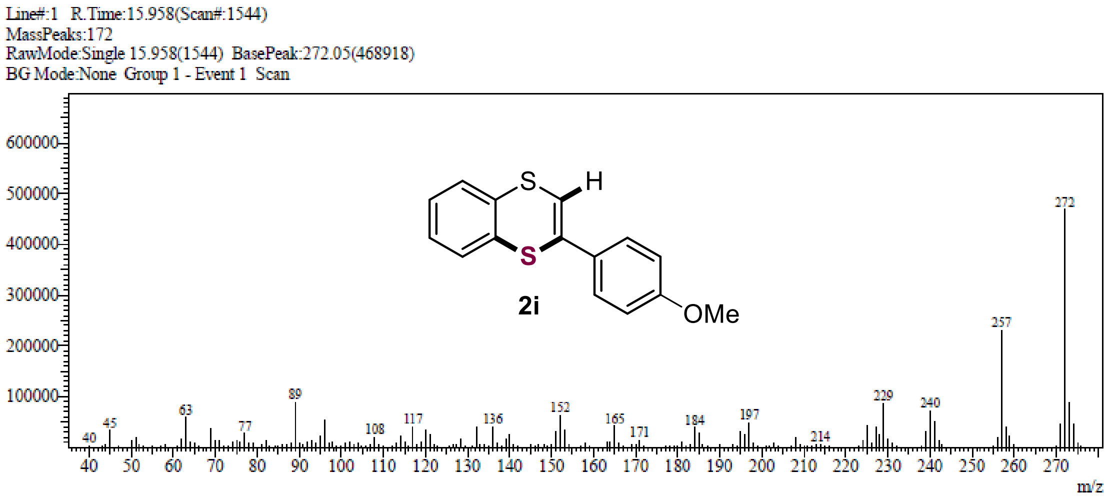

Figure S35. Mass spectrum of **2i**.

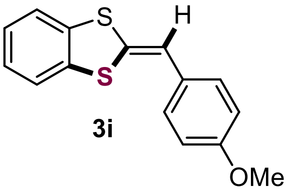

3i

2-(4-methoxybenzylidene)benzo[*d*][1,3]dithiole (**3i**). Following ***General Procedure D***, the product was isolated as a light-yellow solid in 54% yield (29.5 mg) using a gradient elution starting from hexane to hexane/ethyl acetate (95:5). mp 135–137 °C. **^{1}H NMR** (400 MHz, $CDCl_3$): δ 7.30 – 7.26 (m, 2H), 7.25 – 7.19 (m, 2H), 7.13 – 7.06 (m, 2H), 6.92 (d, *J* = 8.8 Hz, 2H), 6.50 (s, 1H), 3.82 (s, 3H). **^{13}C{^{1}H} NMR** (100 MHz, $CDCl_3$): δ 157.8, 136.3, 134.9, 129.6, 129.5, 128.3, 125.8, 125.5, 121.6, 120.9, 114.6, 113.9, 55.3. **HRMS** (ESI) *m*/*z*: $[M+H]^+$ calculated for $C_{15}H_{13}OS_2$, 273.0402; found, 273.0397.

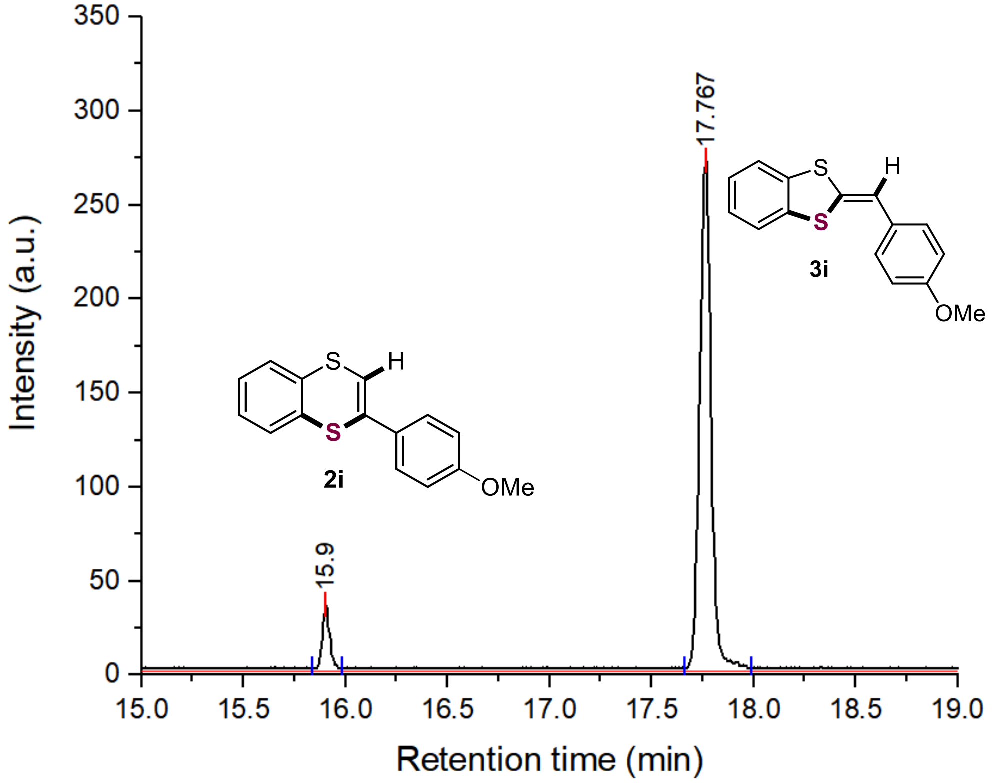

Figure S36. GC-MS expanded chromatogram of the crude reaction mixture for the synthesis of **3i**.

Peak#	Retention time (min)	Area	Area %	Height	Height %
**2i**	15.900	1609	8.11	36	11.69
**3i**	17.767	18219	91.88	272	88.31
		19828	100	308	100

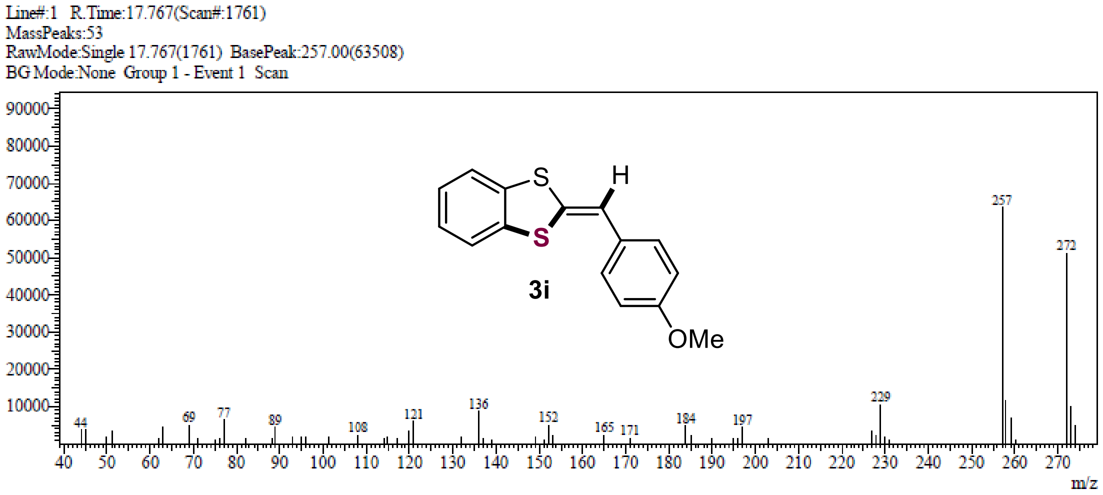

Figure S37. Mass spectrum of **3i**.

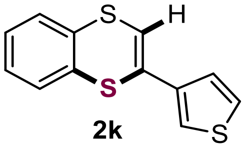

2k

2-(thiophen-3-yl)benzo[*b*][1,4]dithiine (**2k**). Following ***General Procedure C***, the product was isolated as a light-yellow oil in 68% yield (34 mg) using hexane as eluent. **^{1}H NMR** (400 MHz, $CDCl_3$): δ 7.56 (dd, *J* = 3.0, 1.3 Hz, 1H), 7.46 – 7.40 (m, 1H), 7.39 – 7.34 (m, 1H), 7.31 (dd, *J* = 5.1, 3.0 Hz, 1H), 7.28 – 7.20 (m, 3H), 6.71 (s, 1H). **^{13}C{^{1}H} NMR** (100 MHz, $CDCl_3$): δ 138.4, 135.3, 134.9, 134.0, 128.6, 127.9, 127.8, 127.6, 126.6, 125.5, 122.7, 117.3. **HRMS** (ESI) *m*/*z*: $[M]^+$ calculated for $C_{12}H_8S_3$, 247.9783; found, 247.9786.

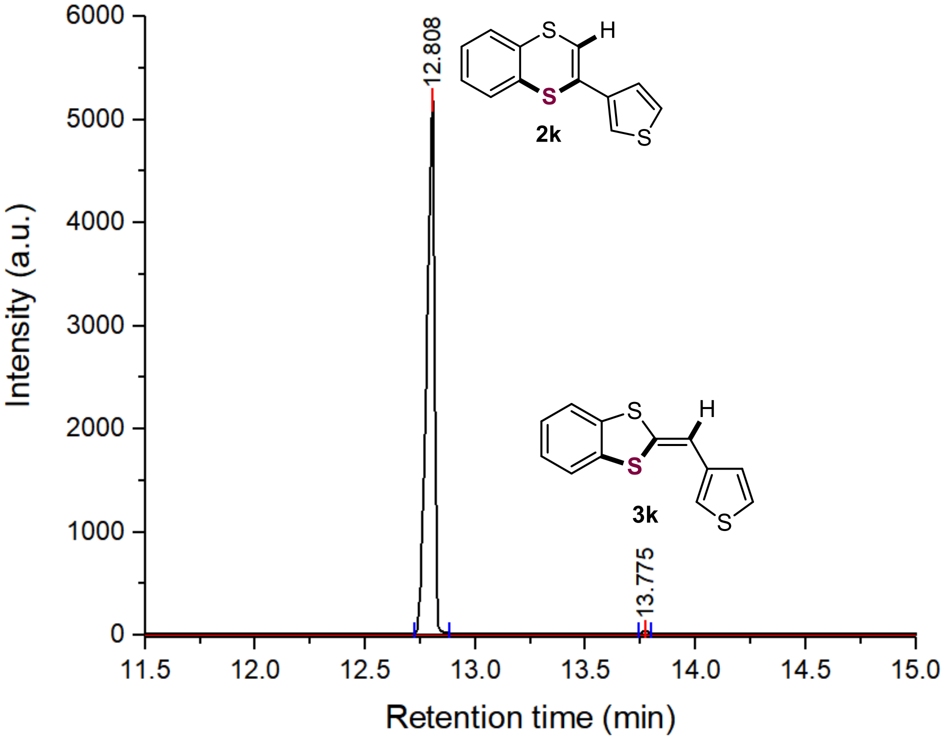

Figure S38. GC-MS expanded chromatogram of the crude reaction mixture for the synthesis of **2k**.

Peak#	Retention time (min)	Area	Area %	Height	Height %
**2k**	12.808	213401	99.33	5205	98.78
**3k**	13.775	1434	0.67	64	1.22
		214835	100	5269	100

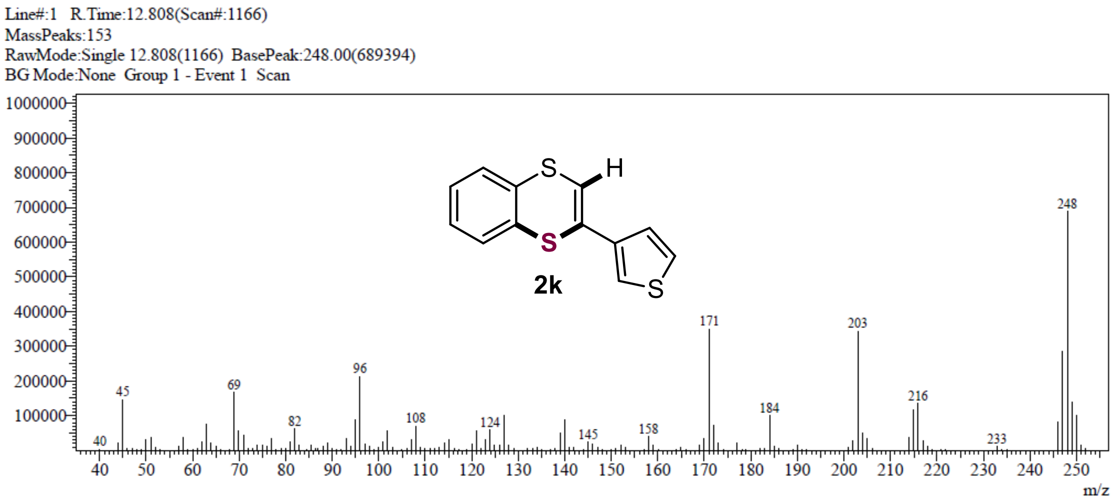

Figure S39. Mass spectrum of **2k**.

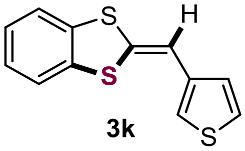

3k

2-(thiophen-3-ylmethylene)benzo[*d*][1,3]dithiole (**3k**). Following ***General Procedure D***, the product was isolated as a white solid in 54% yield (27 mg) using hexane as eluent. mp 144–146 ºC. **^{1}H NMR** (400 MHz, $CDCl_3$): δ 7.31 (dd, *J* = 5.0, 2.9 Hz, 1H), 7.28 – 7.18 (m, 3H), 7.15 – 7.08 (m, 3H), 6.59 (s, 1H). **^{13}C{^{1}H} NMR** (100 MHz, $CDCl_3$): δ 137.9, 136.2, 135.1, 131.6, 127.5, 125.9, 125.5, 125.2, 121.7, 121.1, 120.2, 109.2. **HRMS** (ESI) *m*/*z*: $[M]^+$ calculated for $C_{12}H_8S_3$, 247.9783; found, 247.9787.

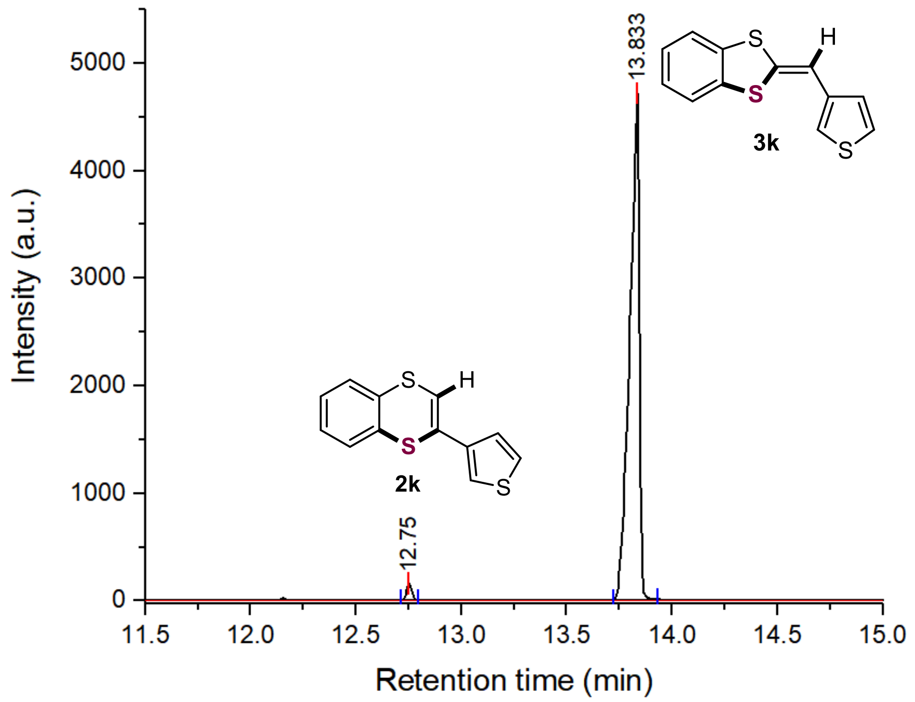

Figure S40. GC-MS expanded chromatogram of the crude reaction mixture for the synthesis of **3k**.

Peak#	Retention time (min)	Area	Area %	Height	Height %
**2k**	12.750	5199	2.02	164	3.43
**3k**	13.833	252783	97.98	4619	96.57
		257982	100	4783	100

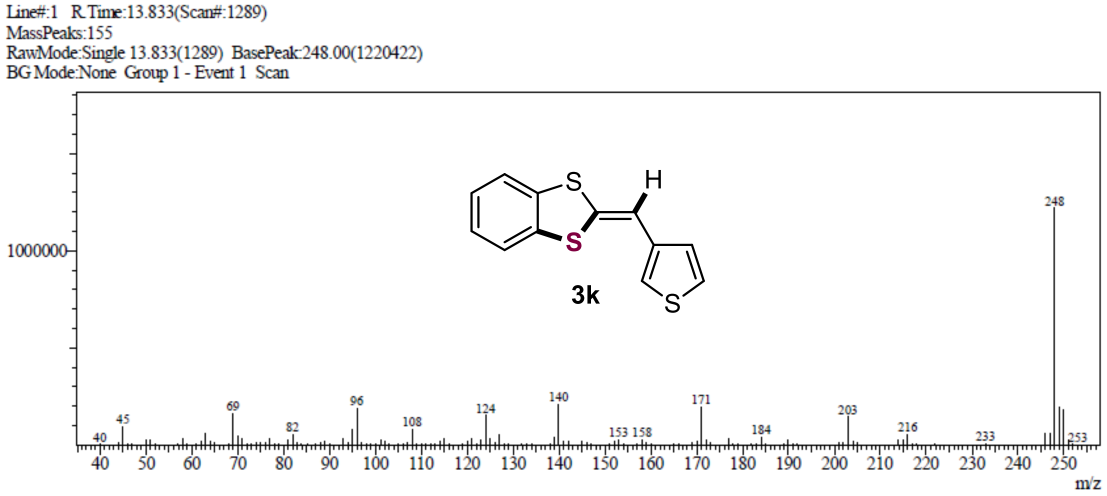

Figure S41. Mass spectrum of **3k**.

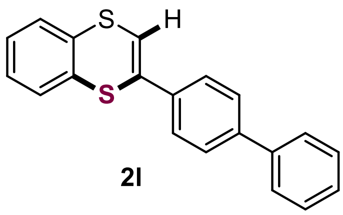

2l

2-([1,1'-biphenyl]-4-yl)benzo[*b*][1,4]dithiine (**2l**). Following ***General Procedure C***, the product was isolated as light-yellow crystals in 60% yield (38 mg) using hexane as eluent. mp 138–140 ºC. **^{1}H NMR** (400 MHz, $CDCl_3$): δ 7.73 – 7.65 (m, 2H), 7.64 – 7.54 (m, 4H), 7.50 – 7.41 (m, 3H), 7.40 – 7.32 (m, 2H), 7.30 – 7.18 (m, 2H), 6.72 (s, 1H). **^{13}C{^{1}H} NMR** (100 MHz, $CDCl_3$): δ 141.4, 140.2, 140.1, 136.0, 135.0, 134.2, 128.8, 128.6, 127.88, 127.87, 127.7, 127.6, 127.3, 127.2, 127.0, 118.2. **HRMS** (ESI) *m*/*z*: $[M]^+$ calculated for $C_{20}H_{14}S_2$, 318.0531; found, 318.0546.

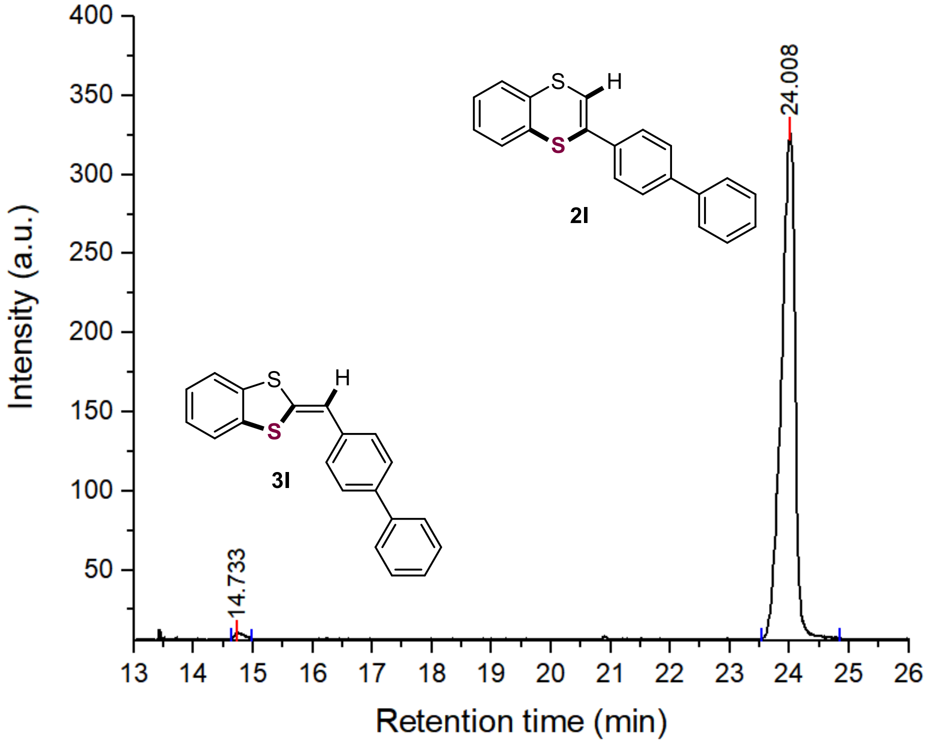

Figure S42. GC-MS expanded chromatogram of the crude reaction mixture for the synthesis of **2l**.

Peak#	Retention time (min)	Area	Area %	Height	Height %
**2l**	14.733	1942	2.11	10	2.96

**3l**	24.008	90044	97.89	328	97.04
		91986	100	338	100

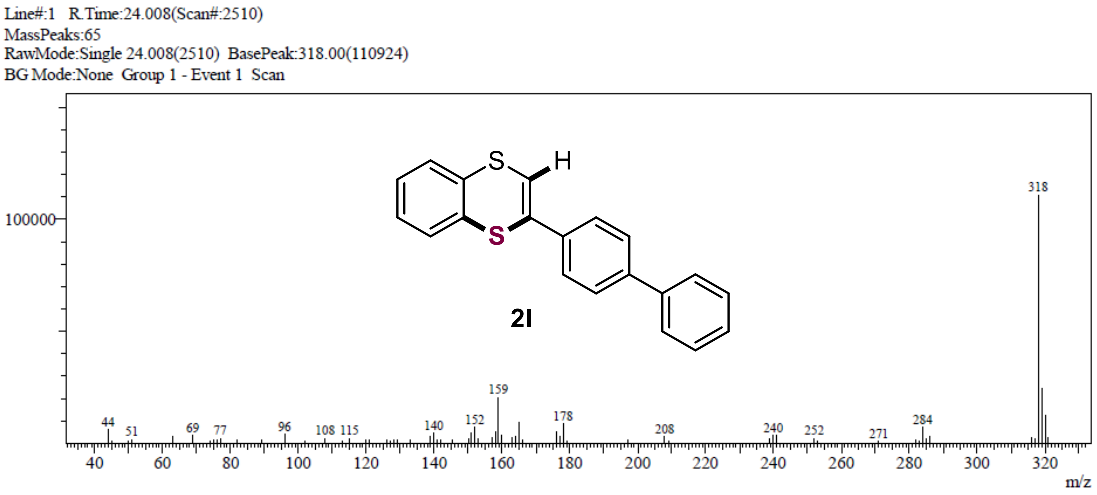

Figure S43. Mass spectrum of **2l**.

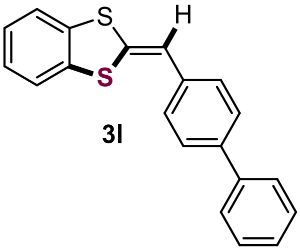

3l

2-([1,1'-biphenyl]-4-ylmethylene)benzo[*d*][1,3]dithiole (**3l**). Following ***General Procedure D***, the product was isolated as a white solid in 56% yield (36 mg) using hexane as eluent. mp 167–169 ºC. **^{1}H NMR** (400 MHz, $CDCl_3$): δ 7.67 – 7.58 (m, 4H), 7.49 – 7.38 (m, 4H), 7.34 (t, *J* = 7.3 Hz, 1H), 7.29 – 7.22 (m, 2H), 7.16 – 7.08 (m, 2H), 6.60 (s, 1H). **^{13}C{^{1}H} NMR** (100 MHz, $CDCl_3$): δ 140.6, 138.5, 136.4, 135.6, 134.7, 132.6, 128.8, 127.3, 127.2, 127.1, 126.9, 126.0, 125.6, 121.7, 120.9, 114.2. **HRMS** (ESI) *m*/*z*: $[M+H]^+$ calculated for $C_{20}H_{15}S_2$, 319.0610; found, 319.0602.

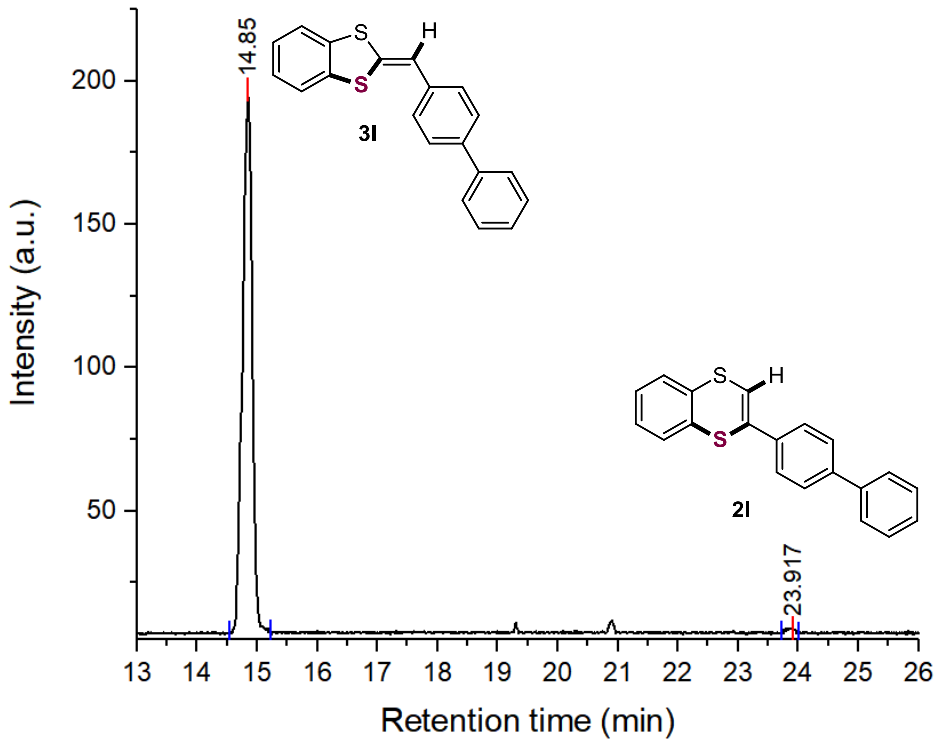

Figure S44. GC-MS expanded chromatogram of the crude reaction mixture for the synthesis of **3l**.

Peak#	Retention time (min)	Area	Area %	Height	Height %
**2l**	14.850	38463	96.10	198	95.65
**3l**	23.917	1561	3.90	9	4.35
		40024	100	207	100

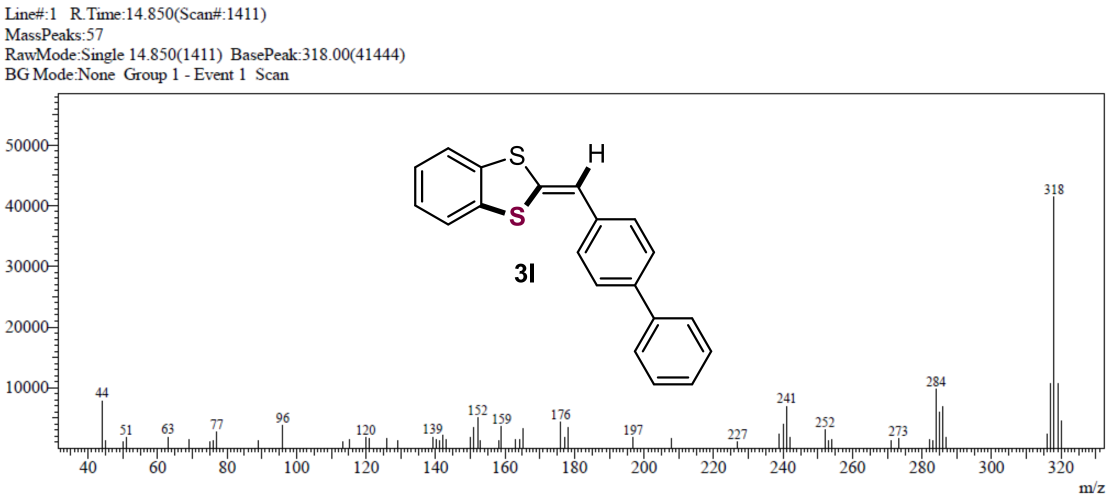

Figure S45. Mass spectrum of **3l**.

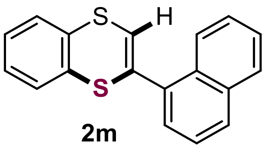

2m

2-(naphthalen-1-yl)benzo[*b*][1,4]dithiine (**2m**). Following ***General Procedure C*** at 90 °C, the product was isolated as light-yellow oil in 40% yield (23.5 mg) using *n*-hexane as eluent. **^{1}H NMR** (400 MHz, $CDCl_3$): δ 8.02 – 7.95 (m, 1H), 7.88 – 7.80 (m, 2H), 7.52 – 7.36 (m, 6H), 7.32 – 7.25 (m, 2H), 6.57 (s, 1H). **^{13}C{^{1}H} NMR** (100 MHz, $CDCl_3$): δ 138.6, 135.9, 134.9, 134.5, 133.7, 131.3, 129.1, 128.39, 128.36, 128.1, 127.9, 127.8, 127.3, 126.5, 126.2, 125.5, 125.2, 122.0. **HRMS** (ESI) *m*/*z*: $[M]^+$ calculated for $C_{18}H_{12}S_2$, 292.0375; found, 292.0375.

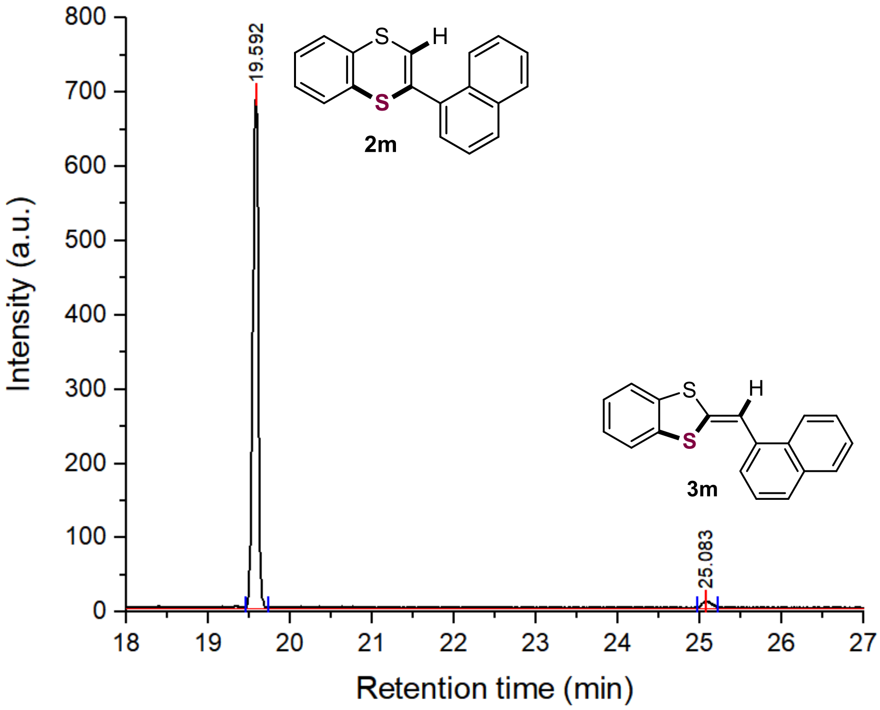

Figure S46. GC-MS expanded chromatogram of the crude reaction mixture for the synthesis of **2m**.

Peak#	Retention time (min)	Area	Area %	Height	Height %
**2m**	19.592	52890	97.58	696	97.89
**3m**	25.083	1311	2.42	15	2.11
		54201	100	711	100

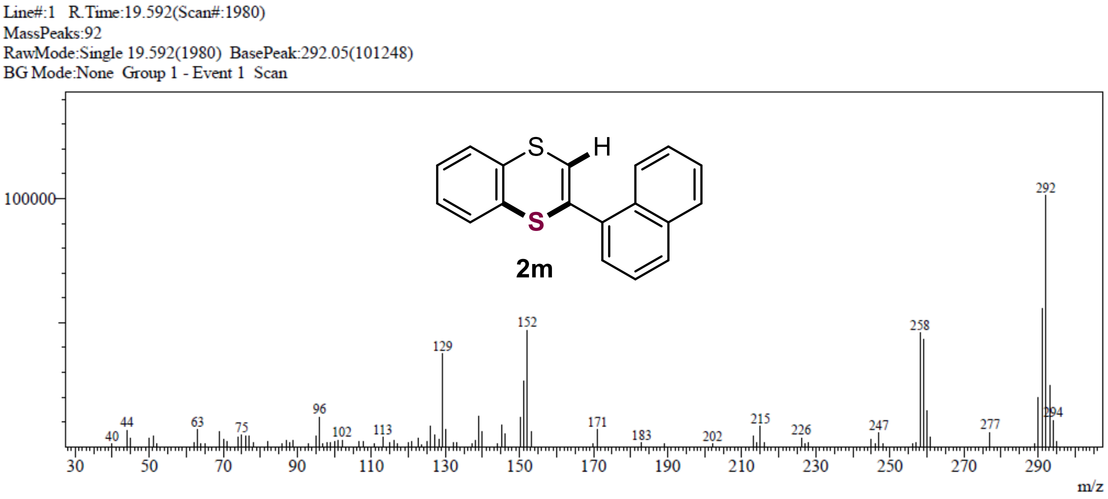

Figure S47. Mass spectrum of **2m**.

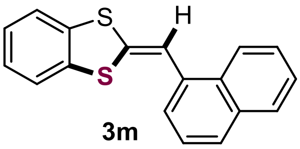

3m

2-(naphthalen-1-ylmethylene)benzo[*d*][1,3]dithiole (**3m**). Following ***General Procedure D***, the product was isolated as a white solid in 61% yield (36 mg) using hexane as eluent. mp 117–119 ºC. **^{1}H NMR** (400 MHz, $CDCl_3$) δ 8.07 – 7.98 (m, 1H), 7.89 – 7.82 (m, 1H), 7.75 (d, *J* = 8.2 Hz, 1H), 7.63 (d, *J* = 7.1 Hz, 1H), 7.55 – 7.46 (m, 3H), 7.26 – 7.21 (m, 1H), 7.19 – 7.13 (m, 1H), 7.13 – 7.00 (m, 3H). **^{13}C{^{1}H} NMR** (100 MHz, $CDCl_3$): δ 135.8, 135.4, 135.0, 134.1, 133.7, 130.8, 128.5, 127.5, 126.1, 126.0, 125.8, 125.6, 125.4, 124.5, 124.0, 121.6, 121.0, 112.2. **HRMS** (ESI) *m*/*z*: $[M]^+$ calculated for $C_{18}H_{12}S_2$, 292.0375; found, 292.0371.

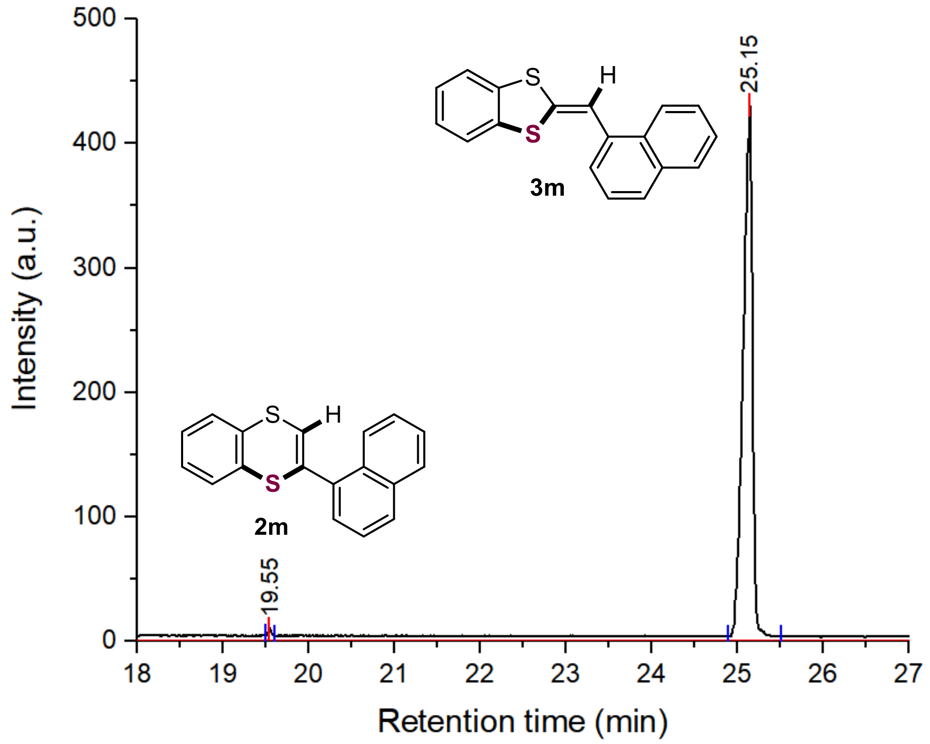

Figure S48. GC-MS expanded chromatogram of the crude reaction mixture for the synthesis of **3m**.

Peak#	Retention time (min)	Area	Area %	Height	Height %
**2m**	19.550	587	1.05	11	2.49
**3m**	25.150	55299	98.95	430	97.51
		55886	100	441	100

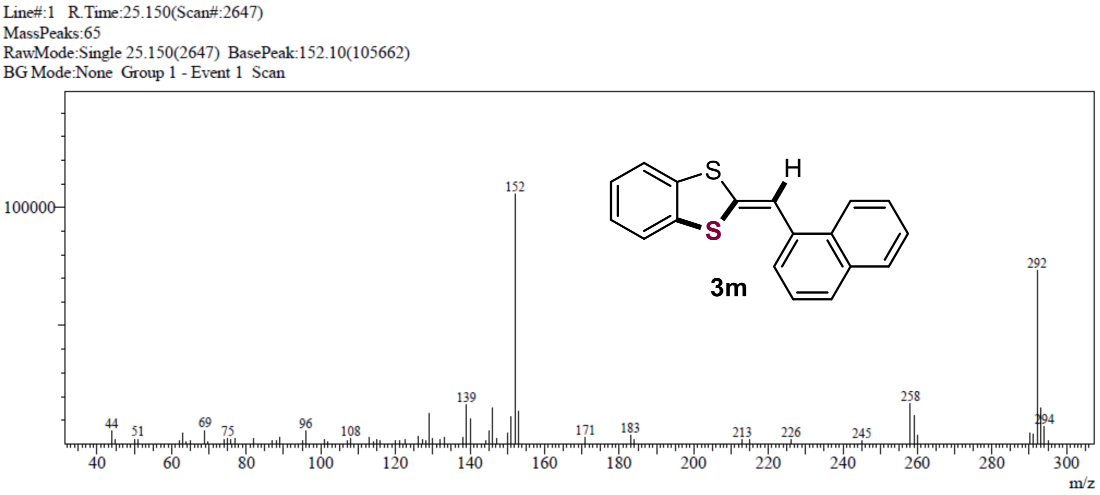

Figure S49. Mass spectrum of **3m**.

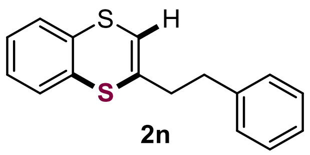

2n

2-phenethylbenzo[*b*][1,4]dithiine (**2n**). Following ***General Procedure C***, the product was isolated as a light-yellow oil in 30% yield (16.5 mg) using hexane as eluent. **^{1}H NMR** (400 MHz, $CDCl_3$) δ 7.38 – 7.30 (m, 2H), 7.28 – 7.15 (m, 5H), 7.14 – 7.07 (m, 2H), 6.12 (s, 1H), 2.93 – 2.84 (m, 2H), 2.76 – 2.68 (m, 2H). **^{13}C{^{1}H} NMR** (100 MHz, $CDCl_3$) δ: 140.54, 140.53, 134.7, 134.4, 128.4, 128.35, 128.30, 127.9, 127.5, 127.4, 126.1, 117.6, 39.1, 35.0. **HRMS** (ESI) *m*/*z*: $[M]^+$ calculated for $C_{16}H_{14}S_2$, 270.0531; found, 270.0531.

3.5. *General procedure E*: base-promoted ring contraction of **2a** into **3a**.

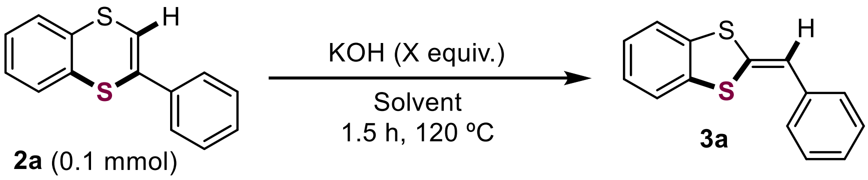

Solvent	KOH	**2a:3a**	**3a**
DMSO	2 equiv.	0:100	70% yield
DMF	2 equiv.	0:100	78% yield
DMF	1 equiv.	0:100	76% yield

In a flame-dried 10 mL Schlenk tube equipped with a magnetic stirring bar, KOH powder (X equiv), 1,4-benzodithiin **2a** (0.024 g, 0.1 mmol) and solvent (0.5 mL) were added. The Schlenk tube was then placed in an oil bath preheated to 120 ºC and stirred for 1.5 h. Afterward, the reaction was cooled to room temperature and diluted with ethyl acetate (90 mL). The mixture was washed with saturated aqueous NaCl solution (2 x 60 mL). The organic layer was dried over anhydrous $MgSO_4$, filtered and concentrated under reduced pressure. The crude product was further purified by automated flash chromatography on silica gel using a Biotage® Selekt flash chromatography system to afford **3a**.

4. ***General procedure F***: synthesis of **4a** through dimerization of **3a**.[8]

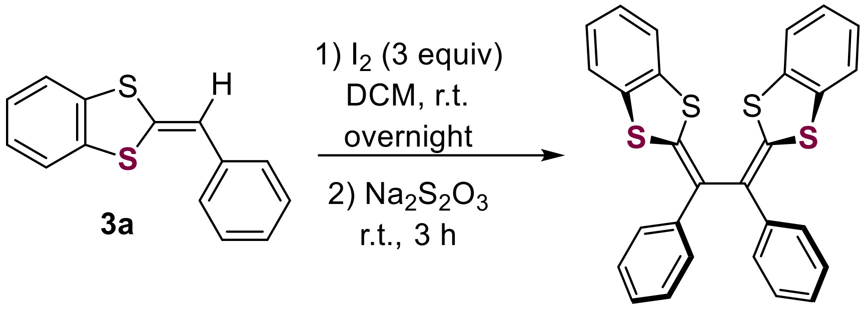

In a 100 mL single-neck flask equipped with a magnetic stirring bar, a mixture of **3a** (0.048 g, 0.2 mmol) and I_2 (0.152 g, 0.6 mmol) in dichloromethane (30 mL) was stirred overnight at room temperature. Afterwards, a saturated aqueous $Na_2S_2O_3$ solution (25 mL) was added, and the mixture was stirred for an additional 3 h. The organic layer was separated, washed with saturated aqueous NaCl solution (2 × 60 mL), dried over anhydrous $MgSO_4$, filtered, and concentrated under reduced pressure. The crude product was further purified by automated flash chromatography on silica gel using a Biotage® Selekt flash chromatography system affording compound **4a** in 42% yield (20.5 mg).

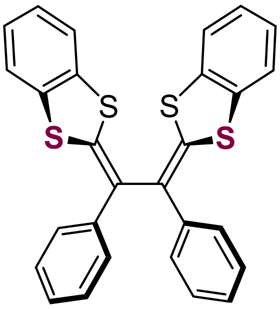

4a

1,2-bis(benzo[*d*][1,3]dithiol-2-ylidene)-1,2-diphenylethane (**4a**).[6] Following ***General Procedure F***, the product was isolated as a light-yellow solid in 42% yield (20.5 mg) using hexane as eluent. mp 206–208 °C. **^{1}H NMR** (400 MHz, $CDCl_3$): δ 7.57 – 7.51 (m, 4H), 7.36 – 7.29 (m, 4H), 7.24 – 7.14 (m, 6H), 7.11 – 7.05 (m, 4H). **^{13}C{^{1}H} NMR** (100 MHz, $CDCl_3$): δ137.8, 136.6, 136.5, 136.2, 128.6, 127.1, 127.0, 125.8, 125.6, 125.5, 121.5, 121.2. **HRMS** (ESI) *m/z*: $[M]^+$ calculated for $C_{28}H_{18}S_4$, 482.0286; found, 482.0274.

5. Colorimetric Analyses

5.1. Colorimetric studies of NaSH·xH$_2$O in DMF and DMSO before and after KOH addition

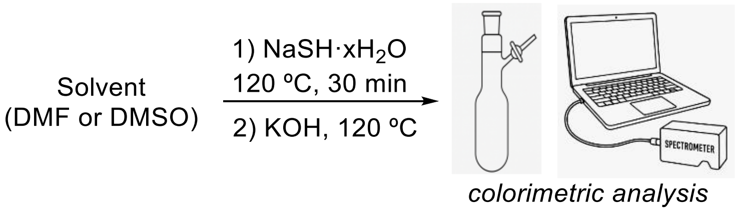

<u>*Reaction setup*</u>: In a flame-dried 25 mL Schlenk tube equipped with a magnetic stirring bar and under an argon atmosphere, solvent (DMF or DMSO, 8 mL) and NaSH·xH$_2$O (60.3% flakes, 0.185 g, 2.0 mmol) were added. The Schlenk tube was then placed in an oil bath preheated to 120 ºC and stirred for 30 min under argon. After this period, KOH (0.179 g, 3.2 mmol) was added, and the reaction was further stirred and monitored as described below.

Color monitoring: Time-dependent color changes were monitored using an Ocean Optics FLAME-S-XR1 spectrophotometer calibrated with a DH-3 Plus Enhanced Calibration UV-Vis-NIR light source coupled to a QP600-1-UV-Vis optical fiber. The distance between the external wall of the Schlenk tube and the light source was maintained at 5.0 cm. All measurements were carried out at 25 °C. The recorded colors of the solutions in both solvents DMF and DMSO (Figures S50 and S51) reflect the visually perceived color evolution over time, which can be correlated with the formation of sulfur-containing species exhibiting absorption bands in the visible region.[9]

For the DMF solutions, at t = 0 min, a broad band is observed centered near 530 nm, which corresponds to the green region of the visible spectrum. As the reaction progressed to t = 10 min, the maximum remained near 530-540 nm, indicating no significant shift toward shorter (green-cyan) or longer (yellow-orange) wavelengths. At t = 20 min, a slight shift was observed, with the maximum still located within the green region. Similar behavior was observed at t = 30 min, with the band maximum appearing near 540-550 nm. In the presence of base, at

t = 0 min, a broader band with maximum around 550 nm is observed, corresponding to green-yellow region. At t = 10 min, the band shifted toward wavelengths below 500 nm. At these early reaction times, the observed shifts suggest that equilibrium between the sulfur-containing species had not yet been established. At t = 20-30 min, the maximum converged again to the 540-550 nm range, consistent with yellow-green to yellow light coloration. Overall, these time-dependent spectral changes within the green-to-yellow region reinforce that the species remain confined to a narrow chromatic window through the experiment, without the evolution toward orange or red (>580 nm), nor into blue or cyan (<500 nm) colors. Notably, after an additional 30 min, the solution became colorless (Figure S52), indicating deactivation or consumption of the species responsible for the observed coloration.

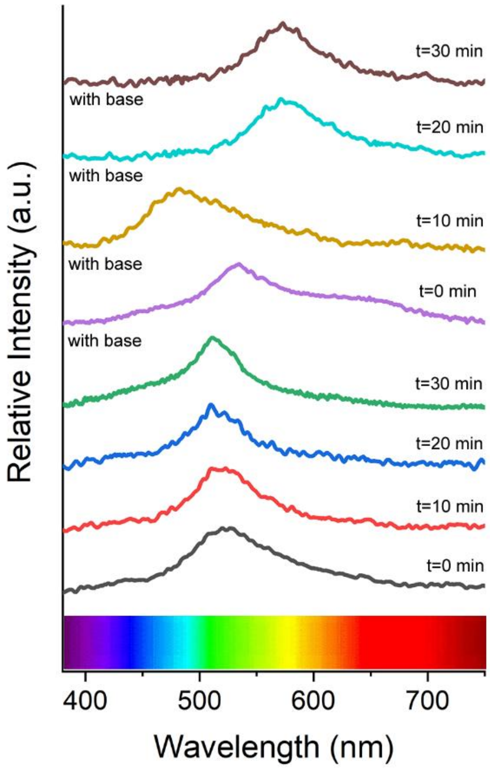

Figure S50. Spectra of the reaction in DMF at different times (0-30 min), with and without base.

Regarding the DMSO solutions, at t = 0 min, a very broad band between 500-650 nm was observed, with a maximum near 550 nm, corresponding to the yellow-green region of the visible spectrum. At this initial stage, this spectral

feature suggests that equilibrium among the species had not yet been established. By t = 10 min, the maximum shifted slightly to 525-530 nm, characteristic of green wavelengths. At t = 20 min, the spectrum remains centered around 530-535 nm. At t = 30 min, the band broadened substantially toward longer wavelengths (550-650 nm), with the maximum shifting above 550 nm, consistent with yellow-green coloration, while still remaining well separated from the orange (>580 nm) or blue-green (<500 nm) colors. These progressive spectral changes indicate that the species in solution evolve exclusively within the green-to-yellow-green range throughout the experiment. In the presence of base, the initial maximum at t = 0 appeared near 520-525 nm, corresponding to the green coloration. At longer reaction times, however, the band becomes broader and the maximum shifts to wavelengths just below 600 nm, within the yellow region. This controlled progression from green to yellow-green, and finally to yellow, without crossing into orange or cyan regions reinforces the restricted photophysical evolution of the system in DMSO. Notably, in contrast to the behavior observed in DMF, no complete loss of coloration was detected even after an additional 30 min under basic conditions, suggesting a sustained persistence of chromophoric sulfur species in DMSO (Figure S52).

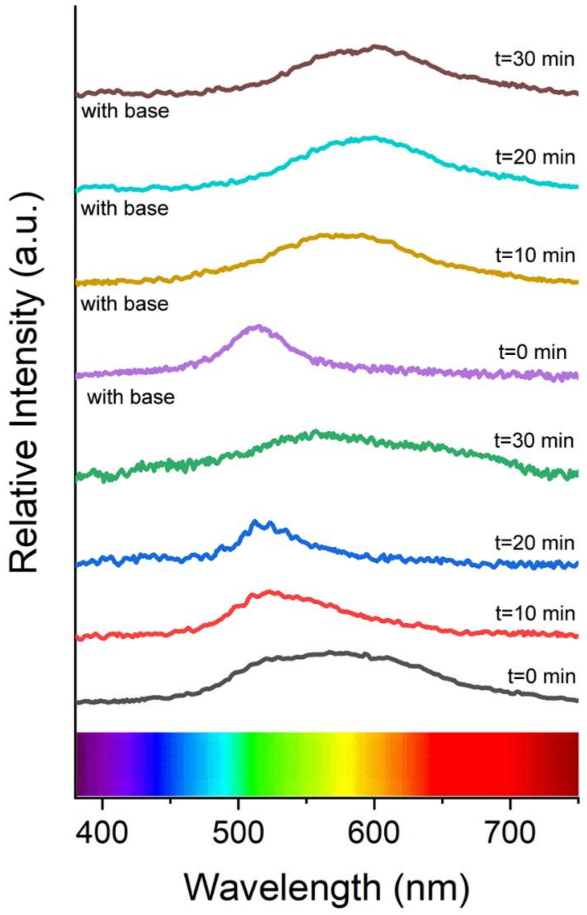

Figure S51. Spectra of the reaction in DMSO at different times (0-30 min), with and without base.

The evolution of the colorimetric spectra for the solutions in DMF and DMSO is consistent with the visual appearance of the solutions photographed throughout the course of the reaction (Figure S52). Minor visual variations can be attributed to the higher concentrations used for imaging and to the broad nature of the colorimetric bands, which may result in slight discrepancies between spectral maxima and the perceived coloration of the samples.

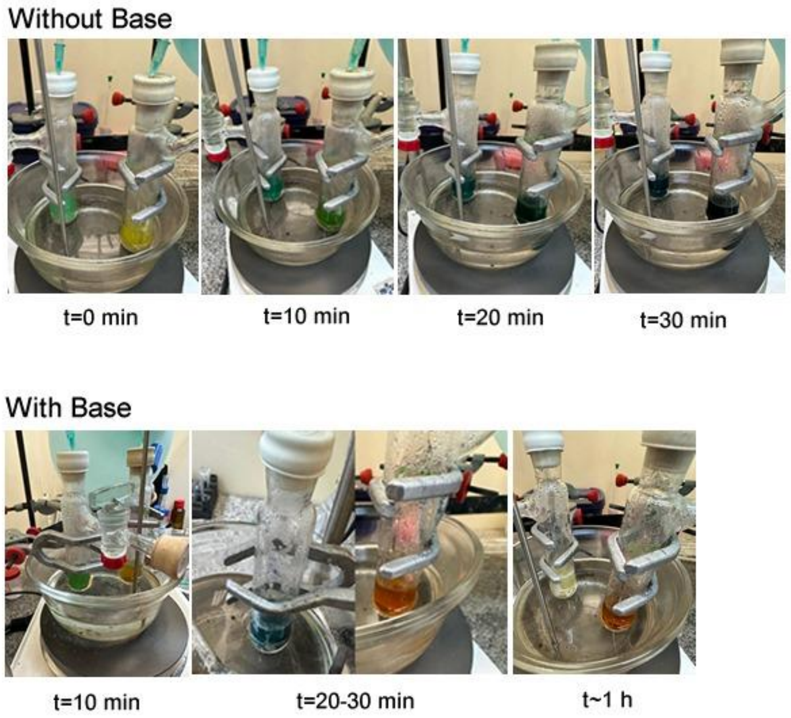

Figure S52. Photographs of the reaction in DMF (left Schlenk) and DMSO (right Schlenk) solutions at different times (0-30 min), with and without base.

Overall, the combined colorimetric and visual data support the establishment of a dynamic equilibrium between polysulfide radical anions ($S_y^{\bullet -}$, y > 1, typically associated with blue/green solutions) and polysulfide dianions (S_x^{2}, x > 1, yellow solutions) upon heating NaSH in both solvents,[10] consistent with the oxidation of HS^-/S^{2-} species in polar aprotic media.[11] In DMF, the subsequent addition of KOH induces only a modest perturbation of this equilibrium, likely accompanied by a gradual deactivation of radical species.[14] In contrast, in DMSO the sulfur-speciation equilibrium appears to shift upon base addition toward polysulfide dianions,[12] as evidenced by the pronounced yellow coloration and corresponding spectral changes.

6. UV-Vis Analyses

6.1 UV-Vis spectra studies of NaSH·xH_2O in DMF and DMSO before and after KOH addition

In a flame-dried 25 mL Schlenk tube equipped with a magnetic stirring bar and under an argon atmosphere, solvent (DMF or DMSO, 1 mL) and NaSH·xH_2O (60.3% flakes, 0.023 g, 0.25 mmol) were added. The Schlenk tube was then placed in an oil bath preheated to 120 ºC and stirred for 25 min under argon. After this period, KOH (0.022 g, 0.4 mmol) was added, and the reaction mixture was stirred and monitored as described below.

The presence of sulfur-based species in the reaction mixture was investigated by UV-Vis absorption spectroscopy and by visual monitoring of the solution color over time in the different solvents studied (DMSO and DMF). UV-Vis spectra were recorded on a Shimadzu UV-2450 spectrophotometer over a spectral range of 250-750 nm. For these experiments, aliquots of the DMSO and DMF solutions (100 μL), both in the absence and presence of added base, were withdrawn at defined time intervals under an argon atmosphere and injected into capped cuvettes prefilled with the corresponding degassed solvent (3.0 mL).

Figure S53 presents two sets of spectra recorded in DMF and DMSO, allowing a direct comparison of the spectral profiles before and after base addition in each solvent. In both media, two main absorption regions are observed: (i) an intense band in the UV/near-UV region (ca. 300-400 nm), attributed to sulfide and polysulfide dianionic species (S_x^{2-}), and (ii) a broad absorption in the visible region (ca. 550-700 nm), with a maximum around 610 nm,[10,11] characteristic of $S_3^{\bullet -}$ species and responsible for the blue coloration of the initial samples.

In DMF, the NaSH solution prior to base addition displays a pronounced absorption band centered at ca. 610 nm, consistent with the intense blue color observed visually and indicative of a significant population of $S_3^{\bullet -}$ radical anions. Upon addition of KOH, this visible-region band progressively decreases in intensity, while absorption in the UV region becomes dominant. Over time, the UV band increases slightly in intensity and broadens, reflecting the gradual predominance of non-colored sulfide or polysulfide dianionic species. At extended reaction times, the solution becomes colorless, consistent with the

deactivation or consumption of the radical species initially responsible for the visible absorption.[14]

In DMSO, the overall trend is similar but more pronounced. The initial sample also displays significant absorption in the visible region, consistent with its blue coloration and attributable to the presence of $S_3^{\bullet-}$ species. Upon addition of base, this visible-region band is nearly completely suppressed within 25 min, accompanied by a marked increase in absorption in the 330-380 nm region and the emergence of a pale-yellow coloration. In particular, the growth of the absorption band near 320 nm upon base addition indicates that the higher basicity of the medium favors the formation of polysulfide species, with this feature assigned to S_4^{2-}.[9] Thus, in DMSO the conversion toward exclusively UV-absorbing species occurs more rapidly and with greater spectral contrast than in DMF. Taken together, both solvents exhibit the same overarching behavior: base addition shifts the sulfur-speciation equilibrium toward species that no longer absorb in the visible region and instead absorbs exclusively in the UV, leading to a distinct color change. The key difference lies in the intensity and kinetics of the transformation, which is gradual in DMF but rapid and essentially complete in DMSO. This behavior indicates a longer persistence of $S_3^{\bullet-}$ species in DMF, consistent with their reported higher stability in this solvent,[13] whereas in DMSO sulfur radical anions are rapidly converted into polysulfide dianions upon base addition,[12] in agreement with the colorimetric results obtained.

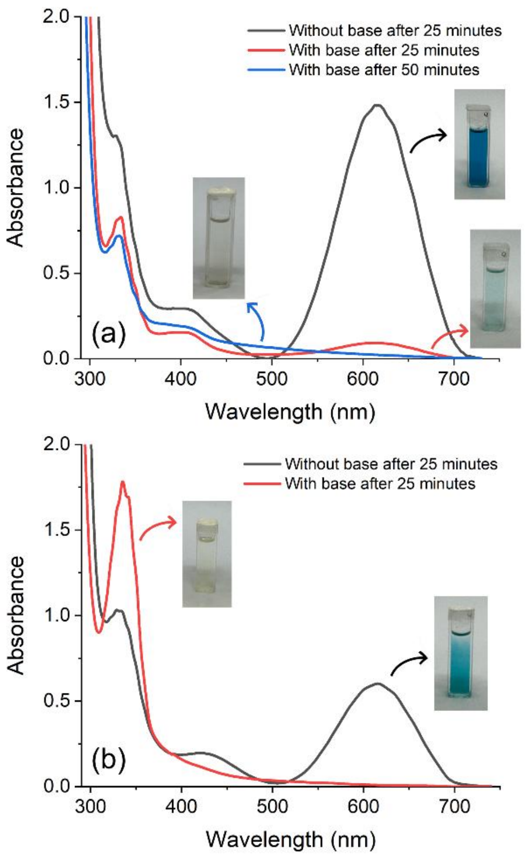

Figure S53. UV-Vis absorption spectra of different reaction aliquots collected with and without added base in (a) DMF and (b) DMSO.

7. HRMS analysis of NaSH·xH_2O in DMF and DMSO after KOH addition

In a flame-dried 25 mL Schlenk tube equipped with a magnetic stirring bar and under an argon atmosphere, solvent (DMF or DMSO, 0.5 mL) and NaSH·xH_2O (60.3% flakes, 0.012 g, 0.125 mmol) were added. The Schlenk tube was then placed in an oil bath preheated to 120 ºC and stirred for 15 min under argon. After this period, KOH (0.011 g, 0.2 mmol) was added and the reaction mixture was stirred for an additional 15 min. Afterward, the reaction was cooled to room temperature. A small aliquot of the crude reaction mixture was diluted in MeCN and analyzed by HRMS.

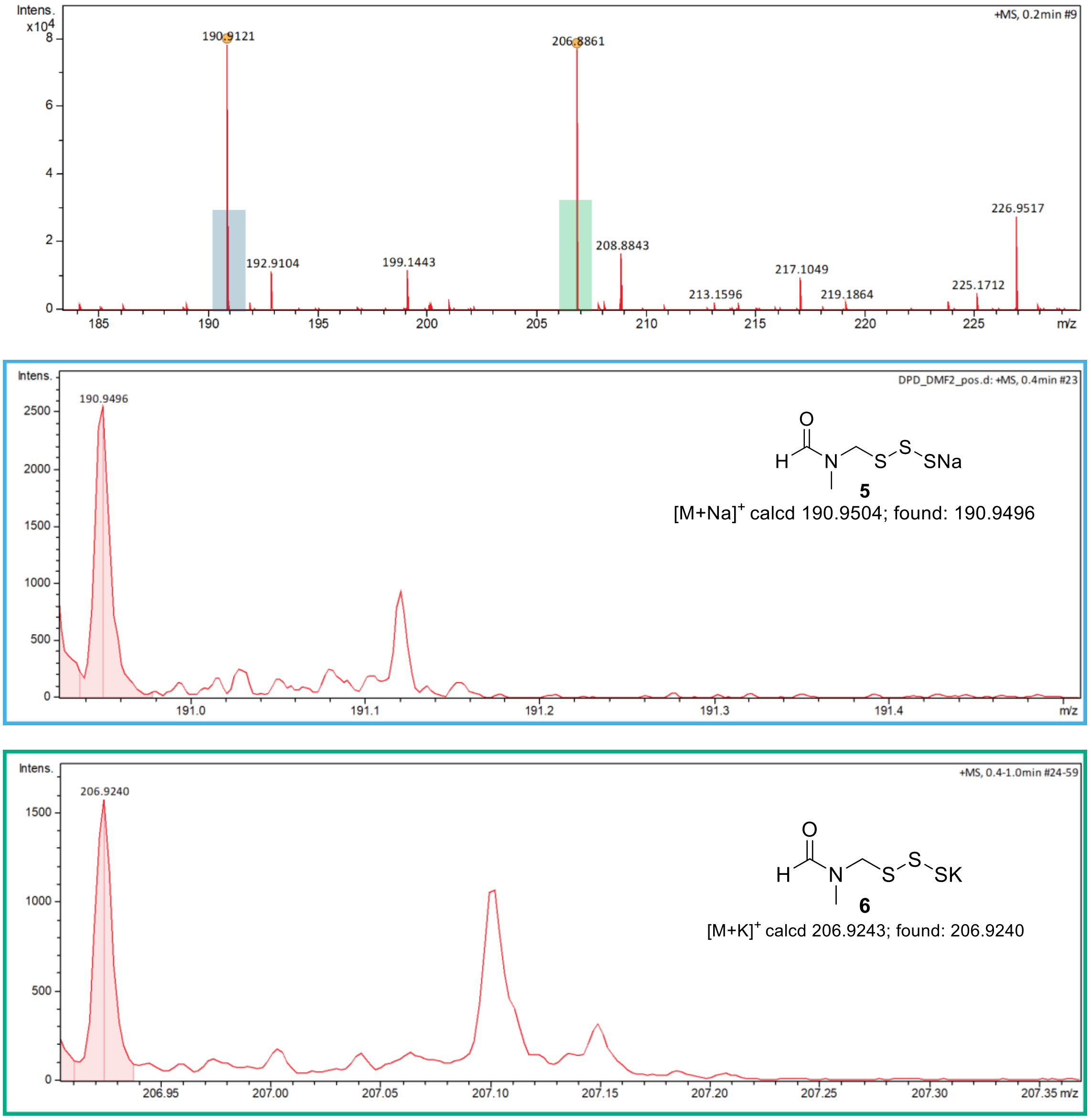

Meas. m/z	Ion Formula	m/z	err [ppm]	mSigma	rdb	e^- Conf	N-Rule
190.9496	$C_3H_6NNaOS_3$	190.9504	4.3	n.a.	1.0	odd	ok
206.9240	$C_3H_6KNOS_3$	206.9243	1.4	n.a.	1.0	odd	ok

Figure S54. HRMS spectrum of the reaction mixture obtained in DMF and signals corresponding to adducts **5** and **6**.

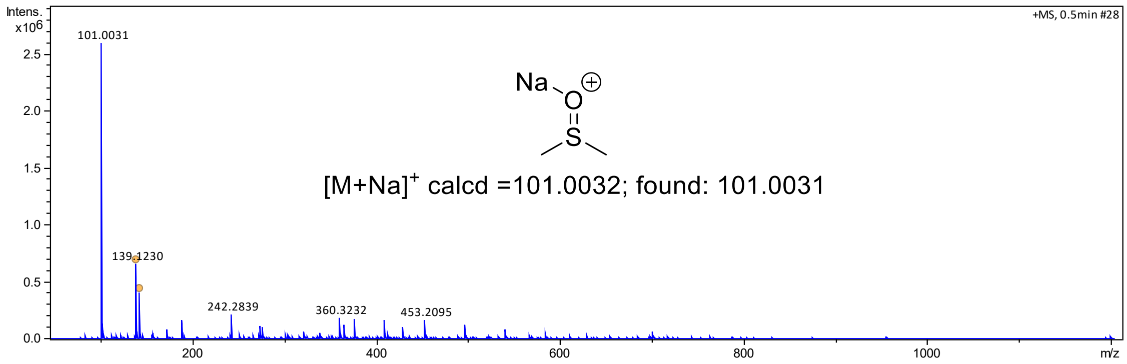

Meas. m/z	Ion Formula	m/z	err [ppm]	mSigma	rdb	e¯ Conf	N-Rule
101.0031	C_2H_6NaOS	101.0032	0.5	3.5	-0.5	even	ok

Figure S55. HRMS spectrum of the reaction mixture obtained in DMSO.

8. Radical Trapping Experiments

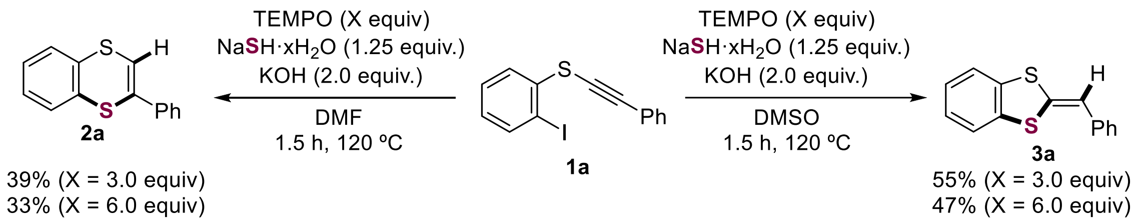

In a flame-dried 25 mL Schlenk tube equipped with a magnetic stirring bar and under an argon atmosphere, $NaSH \cdot xH_2O$ (60.3% flakes, 0.012 g, 0.125 mmol), KOH (0.011 g, 0.2 mmol), 2,2,6,6-tetramethylpiperidin-1-oxyl (TEMPO) (X equiv.) **1a** (0.034 g, 0.1 mmol) and solvent (DMF or DMSO, 0.5 mL) were added. The Schlenk tube was placed in a preheated oil bath at 120 ºC and stirred for 1.5 h under an argon atmosphere. Afterward, the reaction was cooled to room temperature and diluted with ethyl acetate (60 mL). The mixture was washed with saturated aqueous NaCl solution (2 × 30 mL). The organic layer was dried over anhydrous $MgSO_4$, filtered, and concentrated under reduced pressure. The crude product was further purified by automated flash chromatography on silica gel using a Biotage® Selekt flash chromatography system to afford the respective products **2a** and **3a**.

The Addition of 3.0 or 6.0 equivalents of TEMPO decreased the yields but did not suppress either reaction completely. These results indicate that TEMPO probably

intercepts $S_3^{\bullet-}$ within the dynamic S^{2-}/ $S_3^{\bullet-}$ equilibrium,[14] but they also support that the actual nucleophilic species responsible for addition to the alkyne is S^{2-}, in agreement with the mechanistic proposal.

9. Single crystal X-ray diffraction

Yellow irregular-shaped crystals of compound **4a** suitable for single-crystal X-ray diffraction were obtained by dissolving the compound in a minimal amount of CH_2Cl_2 and allowing the solvent to slowly evaporate. A Rigaku XtaLAB Synergy Dualflex diffractometer equipped with HyPix detector and microfocus sealed X-ray tube was used to collect X-ray data for the structural analysis. Data were collected at 100 K by using Cu-Kα radiation (λ = 1.54184 Å). The structure was solved with ShelXT[15] program using the Intrinsic Phasing solution method and by using Olex2[16] as the graphical interface. The model was refined with SHELXL[17] using Least Squares minimization. Anisotropic displacement parameters were applied to all non-hydrogen atoms. Hydrogen atoms were placed in ideal positions and refined as riding atoms with relative isotropic displacement parameters.

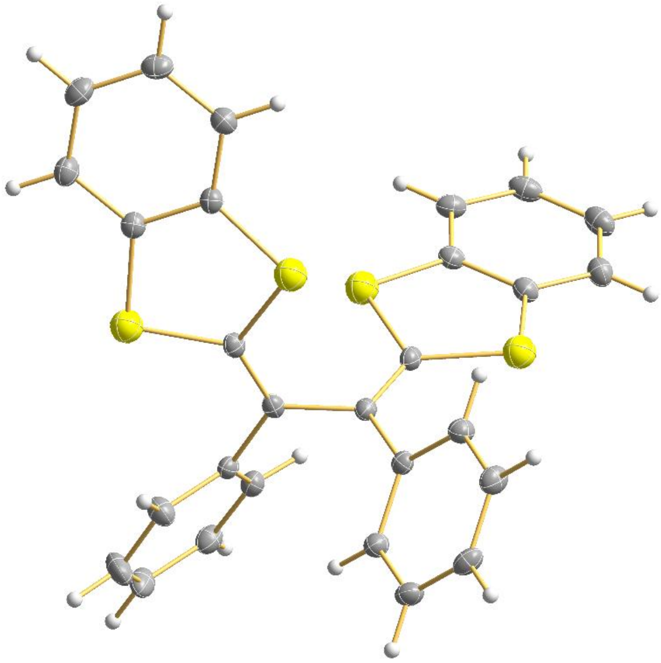

Figure S56. Molecular structure of **4a** with thermal ellipsoids drawn at 50% probability level (carbon = gray, sulfur = yellow, hydrogen = white). $C_{dithiole}$-C_{sp2}-C_{sp2}-$C_{dithiole}$ torsion angle = 81.57 °.

Deposition Number(s) <url href="https://dx.doi.org/10.5517/ccdc.csd.cc2nbsf7">2453229 contain(s) the supplementary crystallographic data for this paper. These data are provided free of charge by the joint Cambridge Crystallographic Data Centre and Fachinformationszentrum Karlsruhe <url href="http://www.ccdc.cam.ac.uk/structures">Access Structures service</url>.

Table 1. Crystallographic data and structure refinement parameters for **4a**.

Molecular formula	$C_{28}H_{18}S_4$
Formula weight (g mol^{-1})	482.66
T (K)	100.01(10)
Crystal system	Monoclinic
Space group	$P2_1/n$
a (Å)	11.37400(8)
b (Å)	15.12768(11)
c (Å)	13.37695(8)
α (°)	90
β (°)	102.5651(7)
γ (°)	90
V (Å^3)	2246.54(3)
Z	4
Radiation type	Cu $K\alpha$
ρ_{calcd} (g cm^{-3})	1.427
μ (mm^{-1})	3.990
F(000)	1000
Crystal size (mm)	0.16 × 0.11 × 0.10
θ range (°)	8.946 to 158.968
Limiting indices (*h, k, l*)	$-14 \leq h \leq 14$
	$-19 \leq k \leq 19$
	$-16 \leq l \leq 13$
Reflections collected	28138
Reflections unique (R_{int})	4863 (0.0384)
Completeness to θ_{max} (%)	100.00
Data / restraints / param.	4863 / 0 / 289
Absorption correction	Multi-scan
Min. and max. transmission	0.811 and 1.000
R_1 [I > 2σ(I)]	0.0300
wR_2 [I > 2σ(I)]	0.0769
R_1 (all data)	0.0317
wR_2 (all data)	0.0781
S on F^2	1.064
Largest diff. peak and hole (e Å^{-3})	0.33 and −0.28

10. NMR Spectra

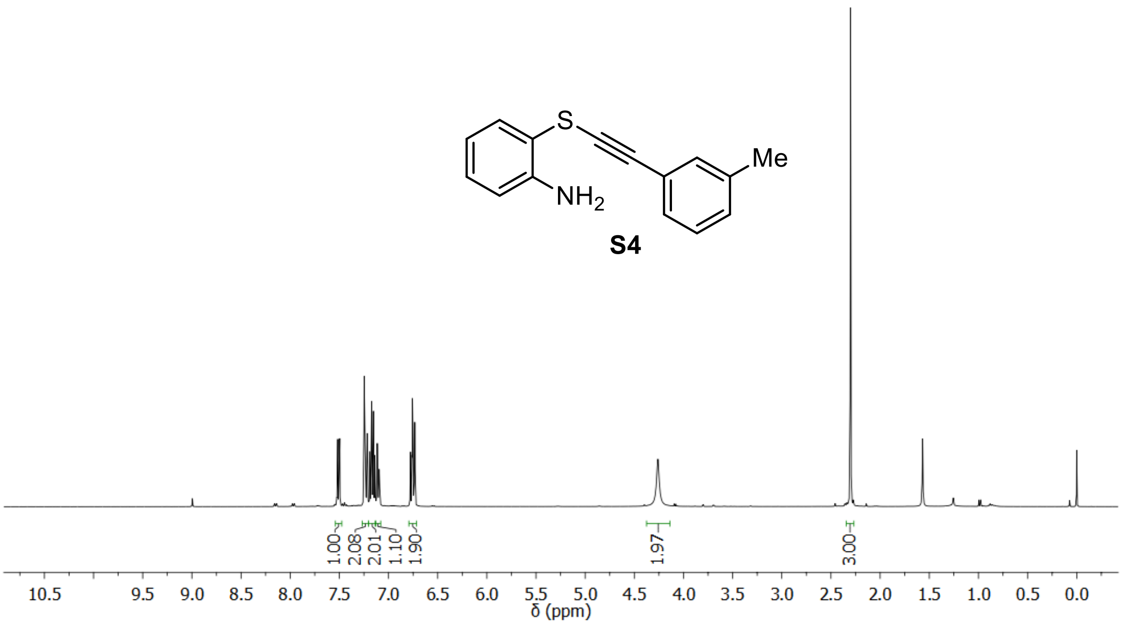

Figure S57. ^{1}H NMR (400 MHz, $CDCl_3$) spectrum of **S4.**

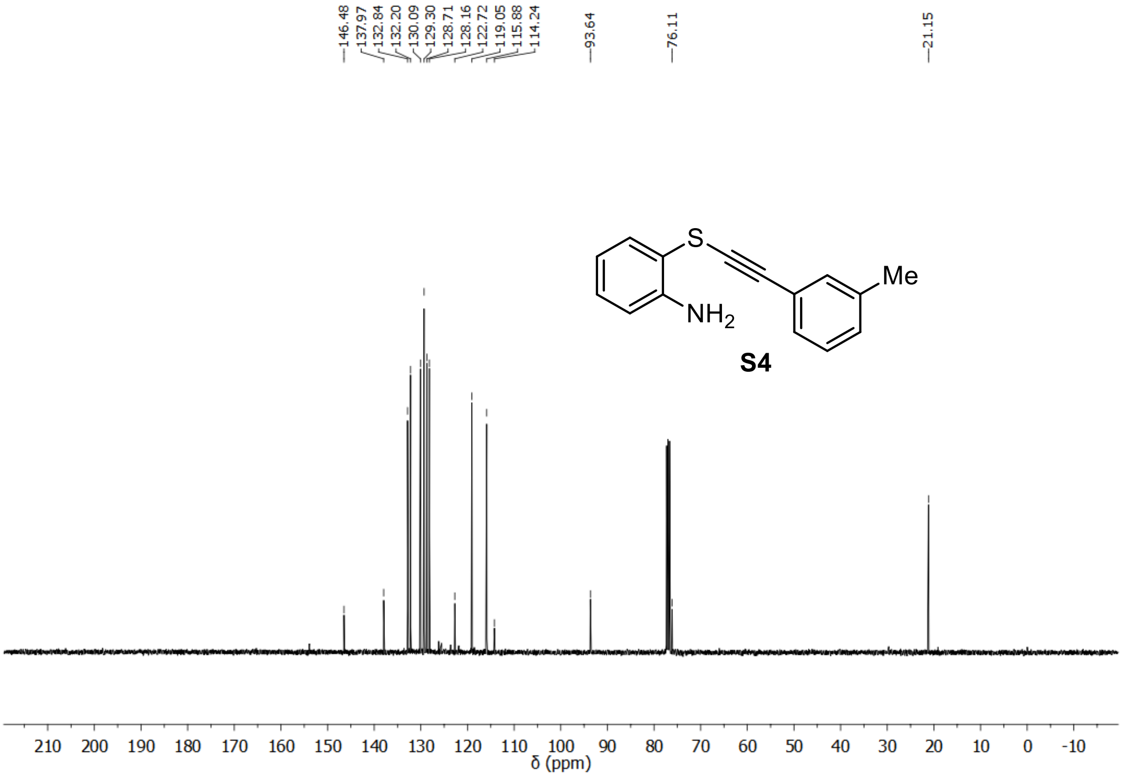

Figure S58. ^{13}C{^{1}H} NMR (100 MHz, $CDCl_3$) spectrum of **S4.**

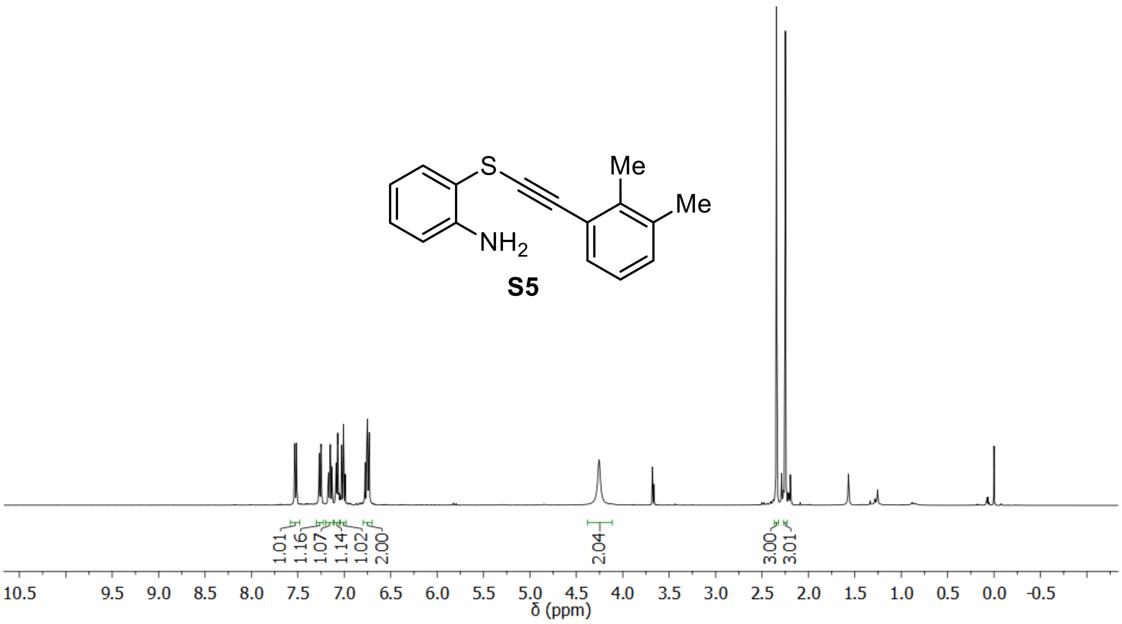

Figure S59. ^{1}H NMR (400 MHz, $CDCl_3$) spectrum of **S5.**

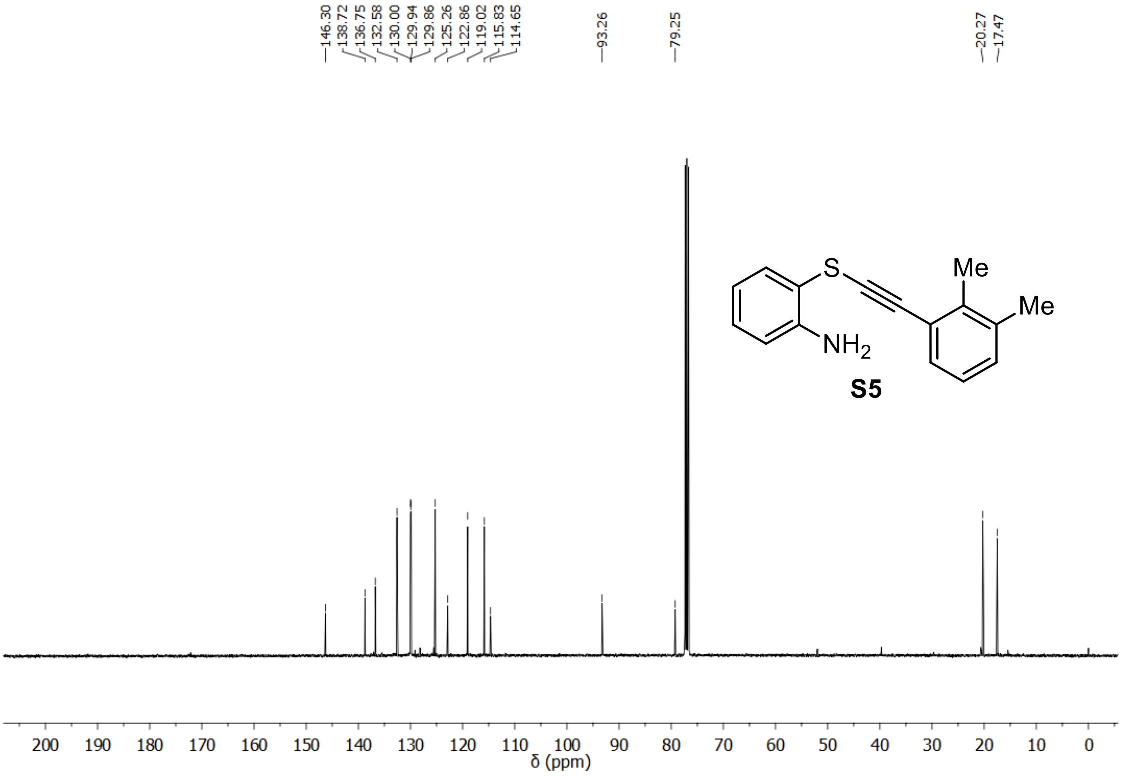

Figure S60. $^{13}C\{^1H\}$ NMR (100 MHz, $CDCl_3$) spectrum of **S5.**

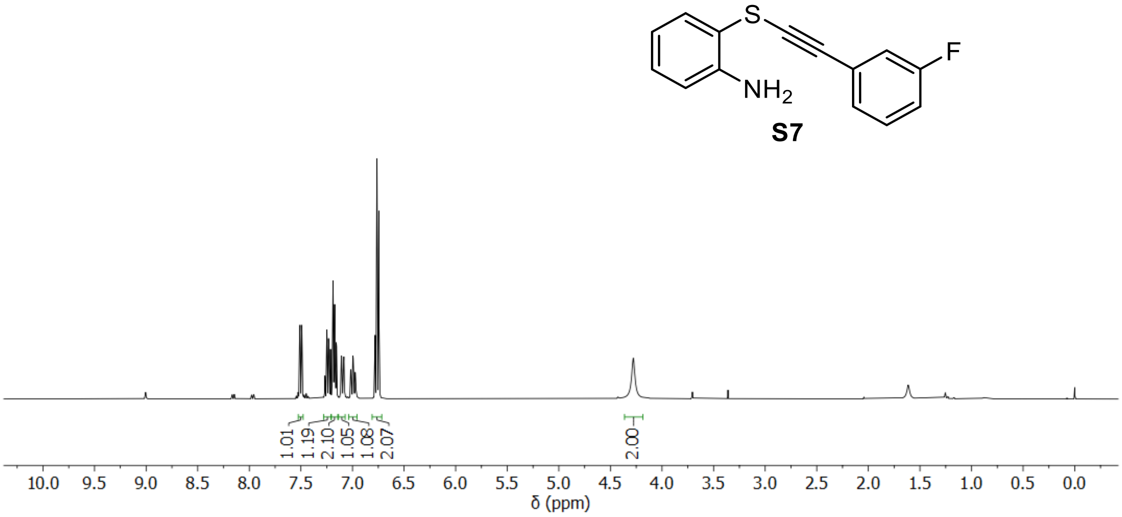

Figure S61. ^{1}H NMR (400 MHz, $CDCl_3$) spectrum of **S7.**

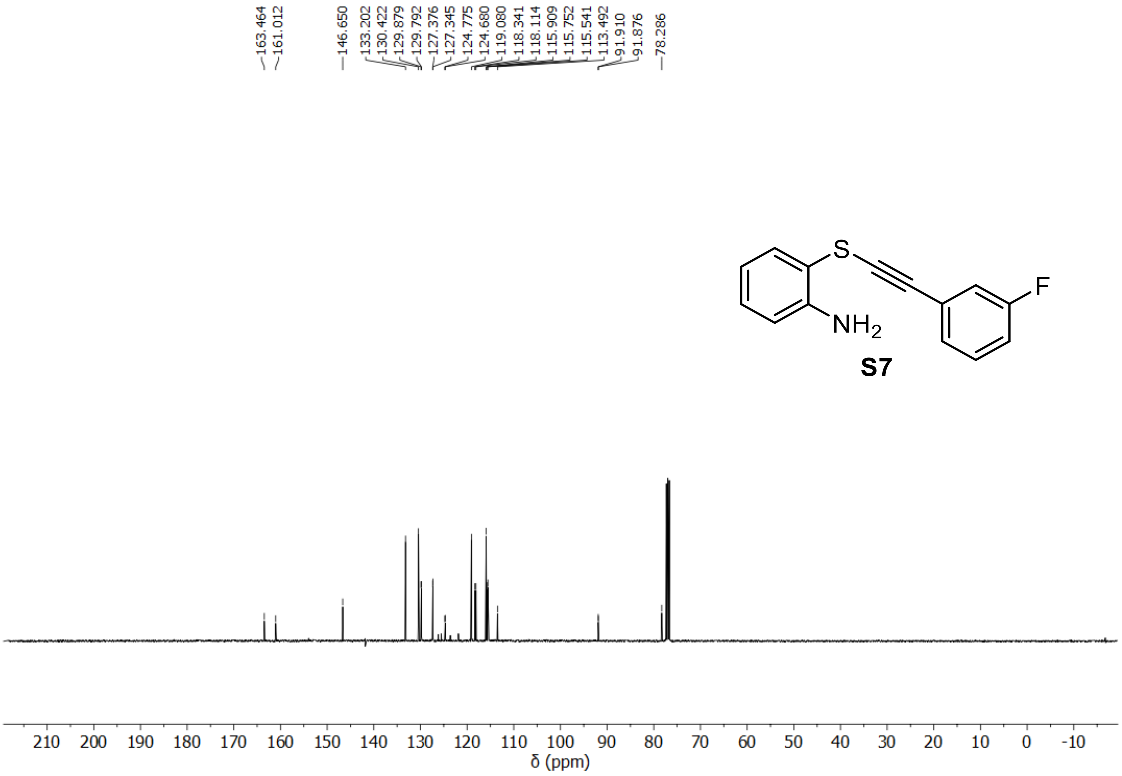

Figure S62. $^{13}C\{^{1}H\}$ NMR (100 MHz, $CDCl_3$) spectrum of **S7.**

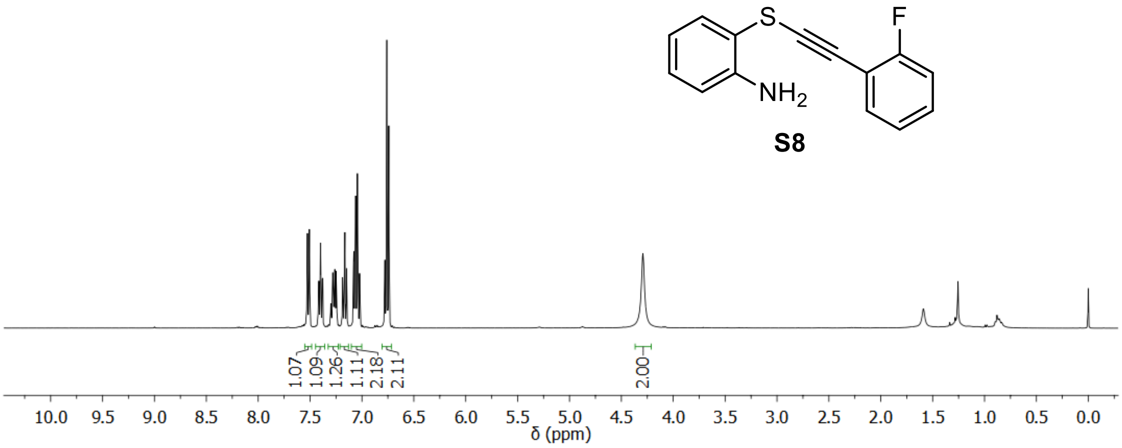

Figure S63. ¹H NMR (400 MHz, $CDCl_3$) spectrum of **S8**.

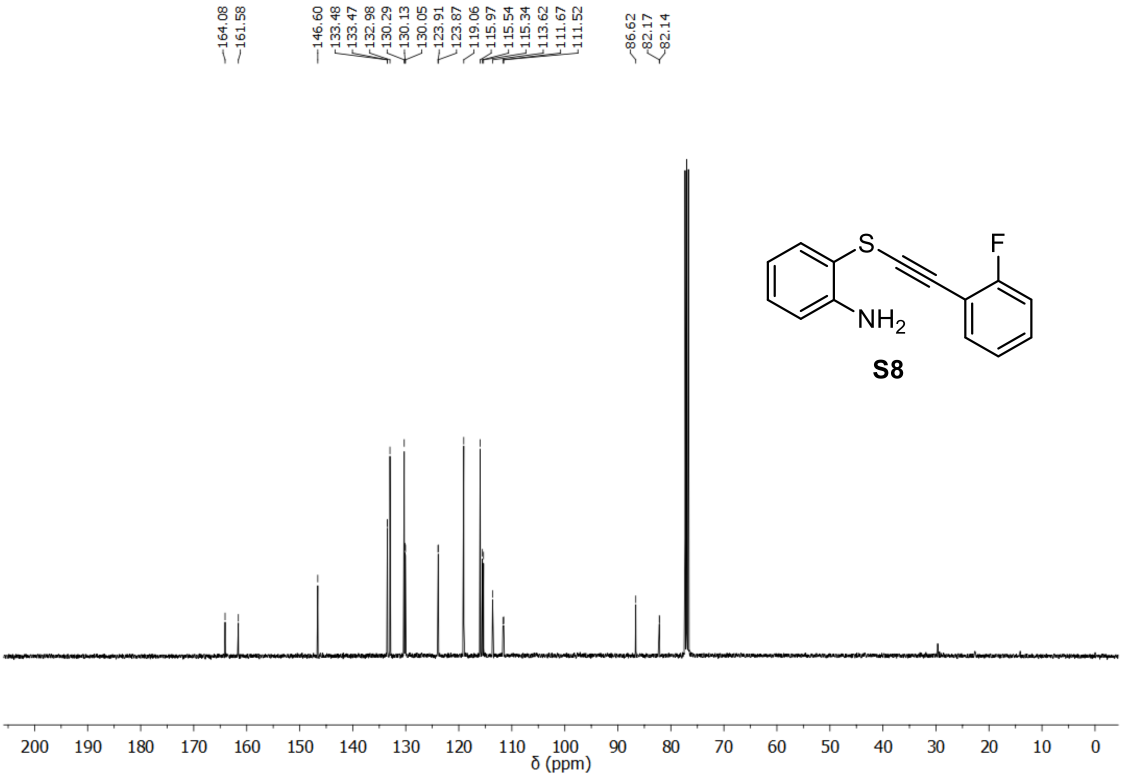

Figure S64. $^{13}C\{^{1}H\}$ NMR (100 MHz, $CDCl_3$) spectrum of **S8**.

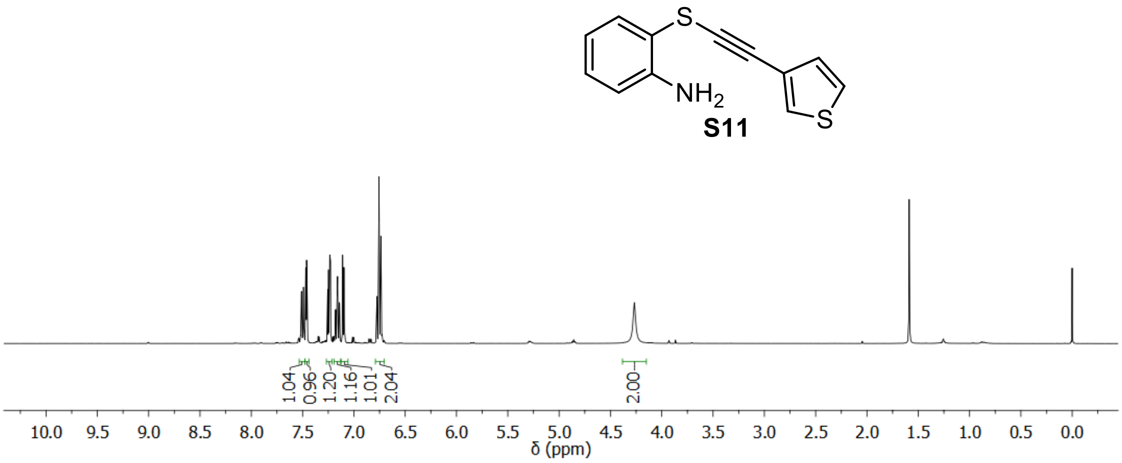

Figure S65. ^{1}H NMR (400 MHz, $CDCl_3$) spectrum of **S11**.

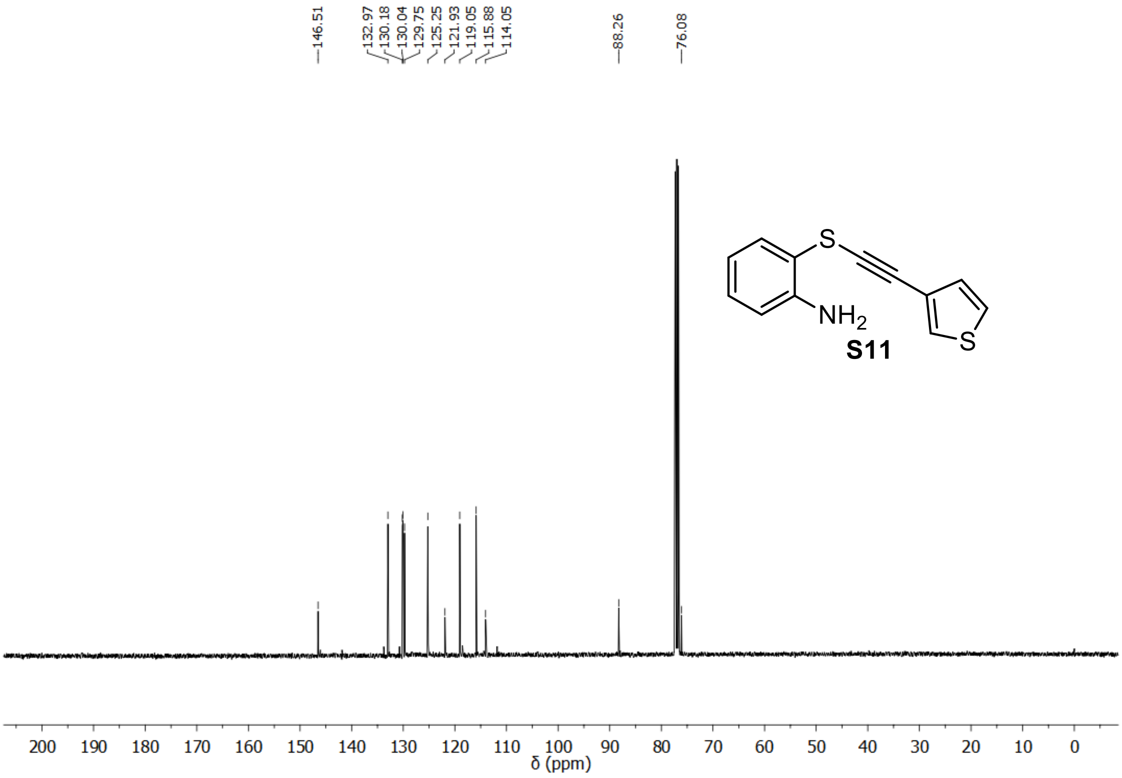

Figure S66. $^{13}C\{^{1}H\}$ NMR (100 MHz, $CDCl_3$) spectrum of **S11**.

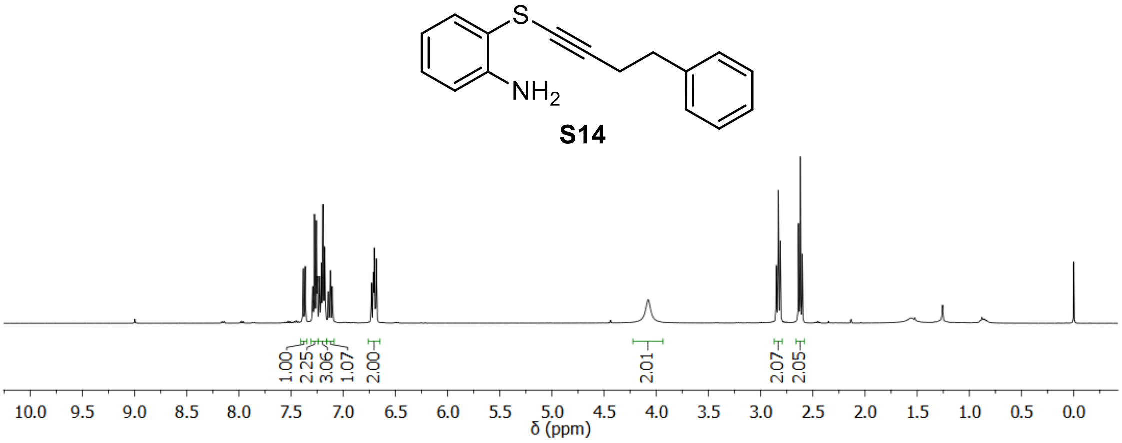

Figure S67. ^{1}H NMR (400 MHz, $CDCl_3$) spectrum of **S14**.

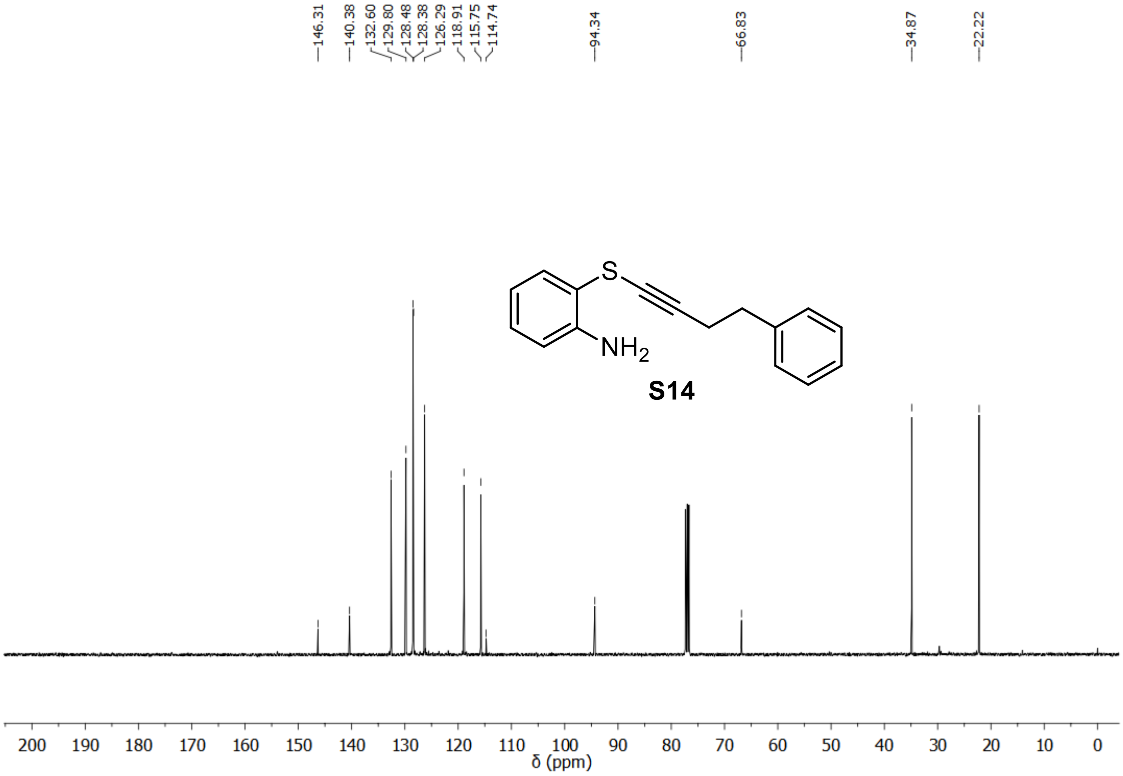

Figure S68. $^{13}C\{^{1}H\}$ NMR (100 MHz, $CDCl_3$) spectrum of **S14**.

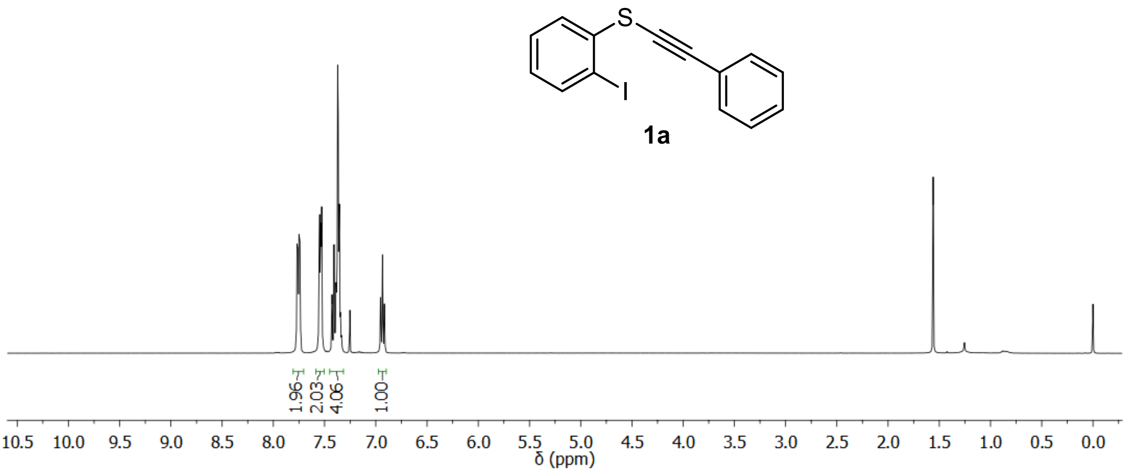

Figure S69. ^{1}H NMR (400 MHz, $CDCl_3$) spectrum of **1a.**

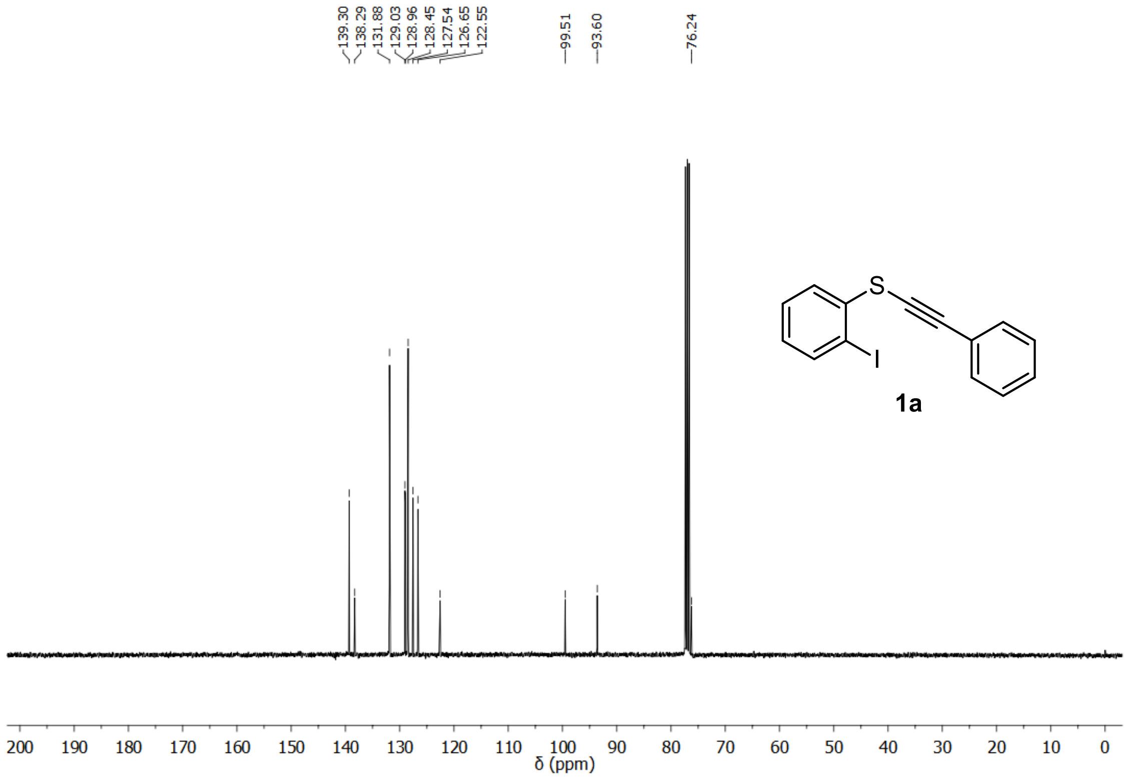

Figure S70. $^{13}C\{^{1}H\}$ NMR (100 MHz, $CDCl_3$) spectrum of **1a.**

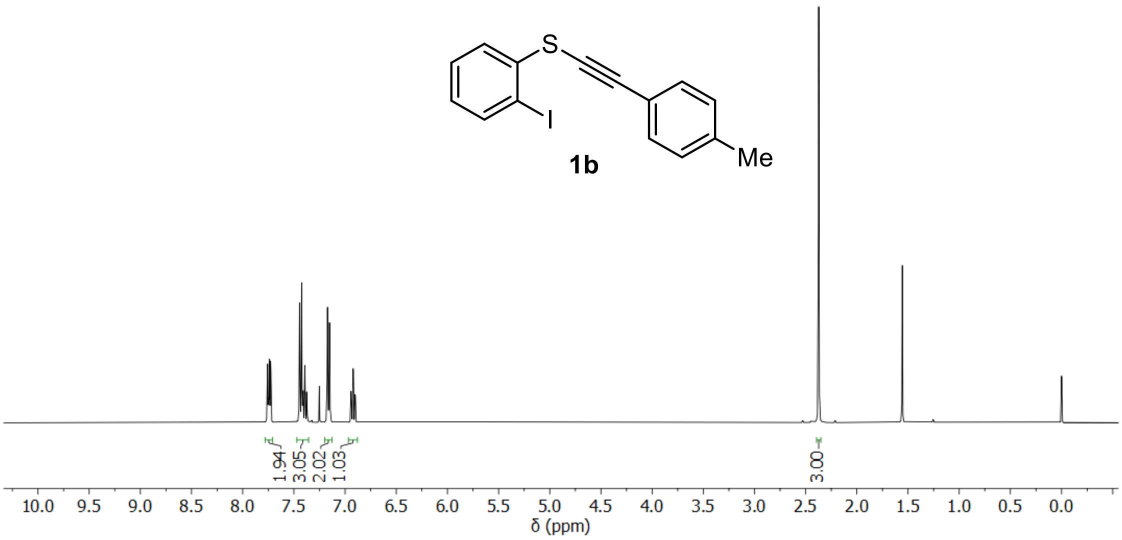

Figure S71. ^{1}H NMR (400 MHz, $CDCl_3$) spectrum of **1b.**

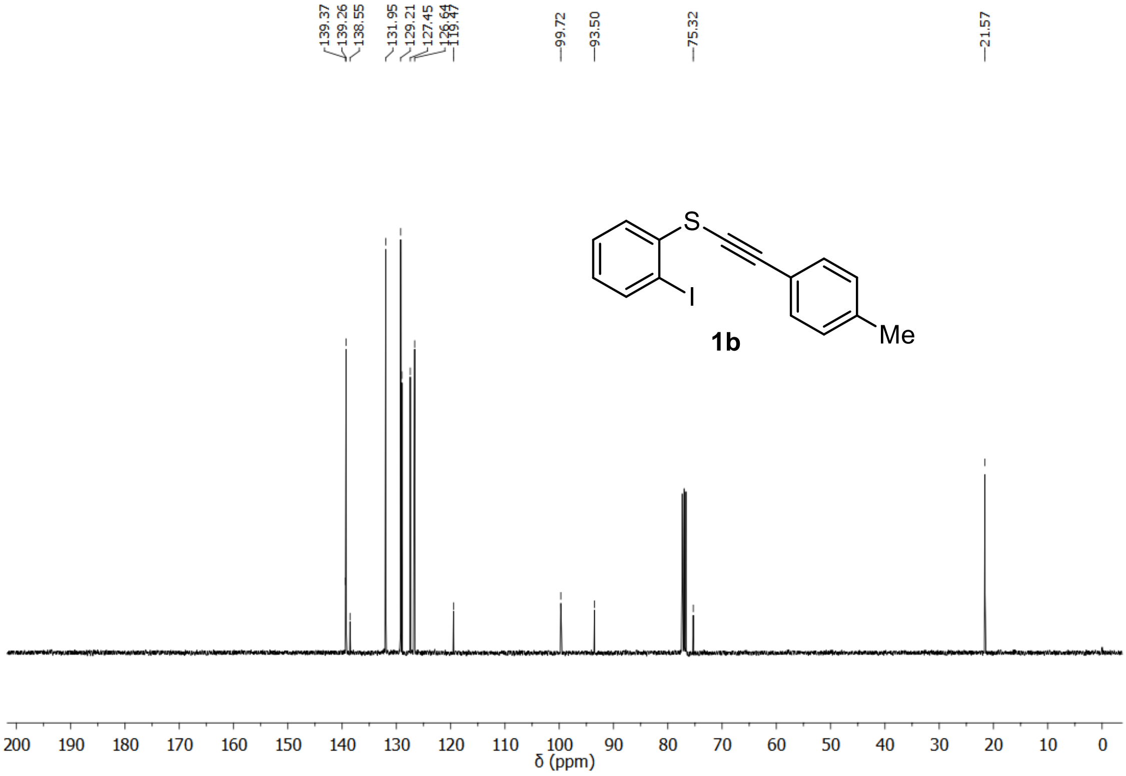

Figure S72. $^{13}C\{^{1}H\}$ NMR (100 MHz, $CDCl_3$) spectrum of **1b.**

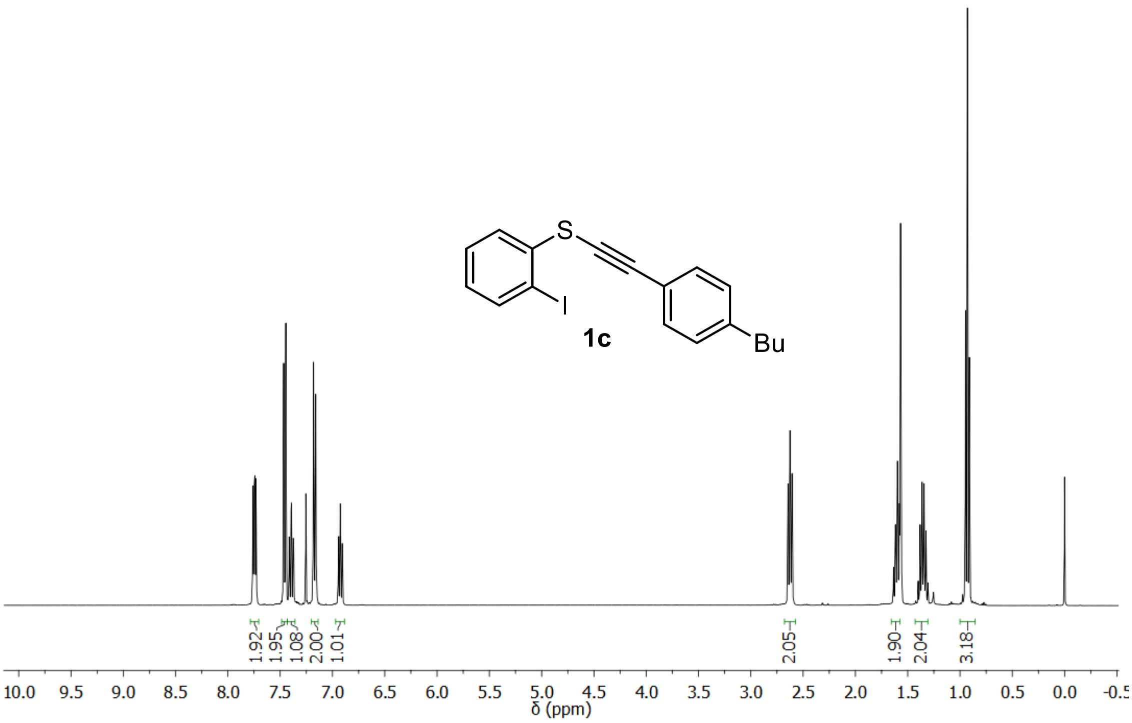

Figure S73. ^{1}H NMR (400 MHz, $CDCl_3$) spectrum of **1c.**

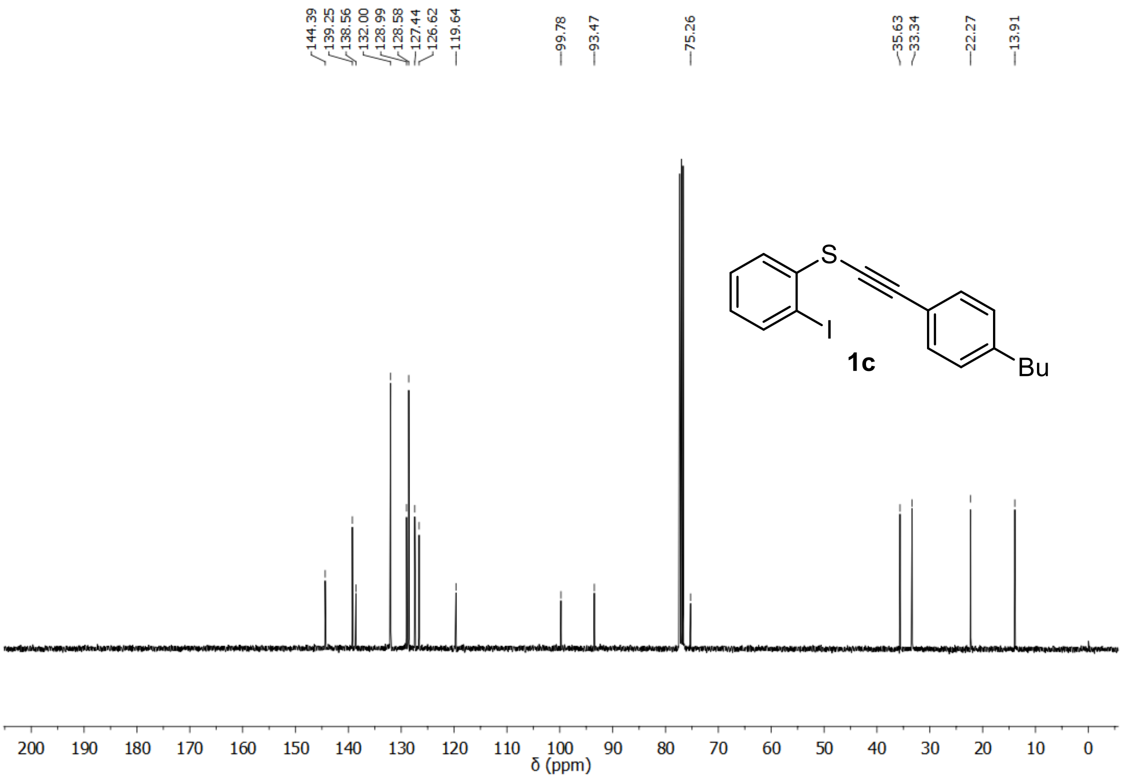

Figure S74. $^{13}C\{^{1}H\}$ NMR (100 MHz, $CDCl_3$) spectrum of **1c.**

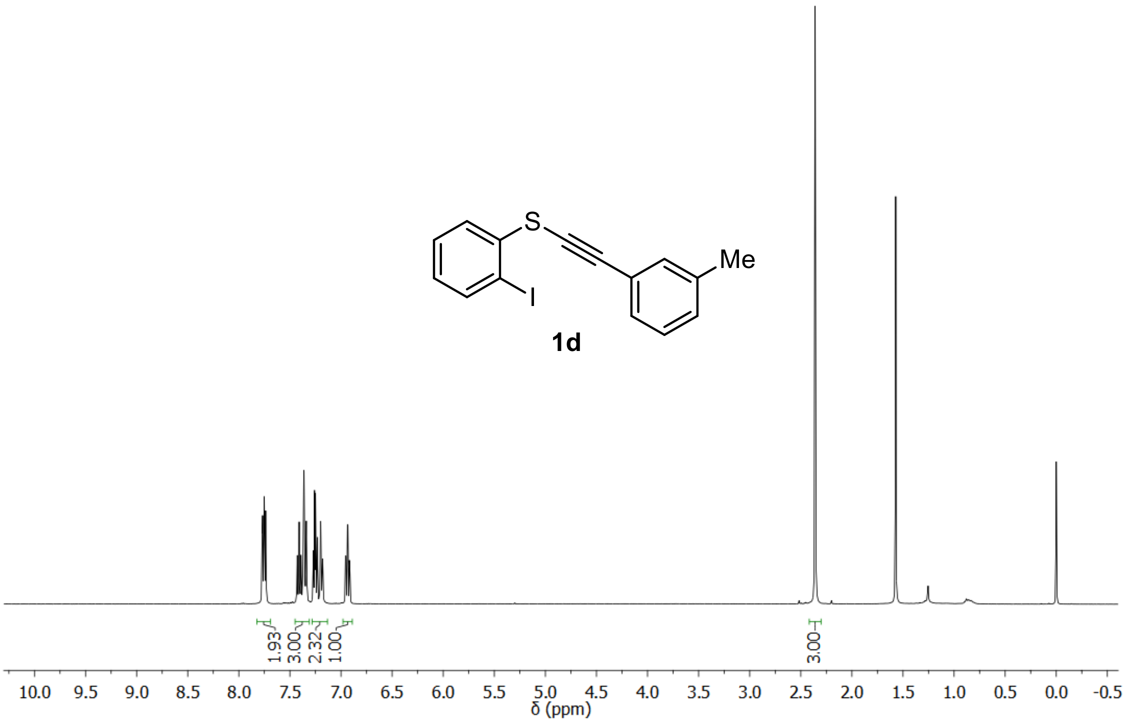

Figure S75. ^{1}H NMR (400 MHz, $CDCl_3$) spectrum of **1d.**

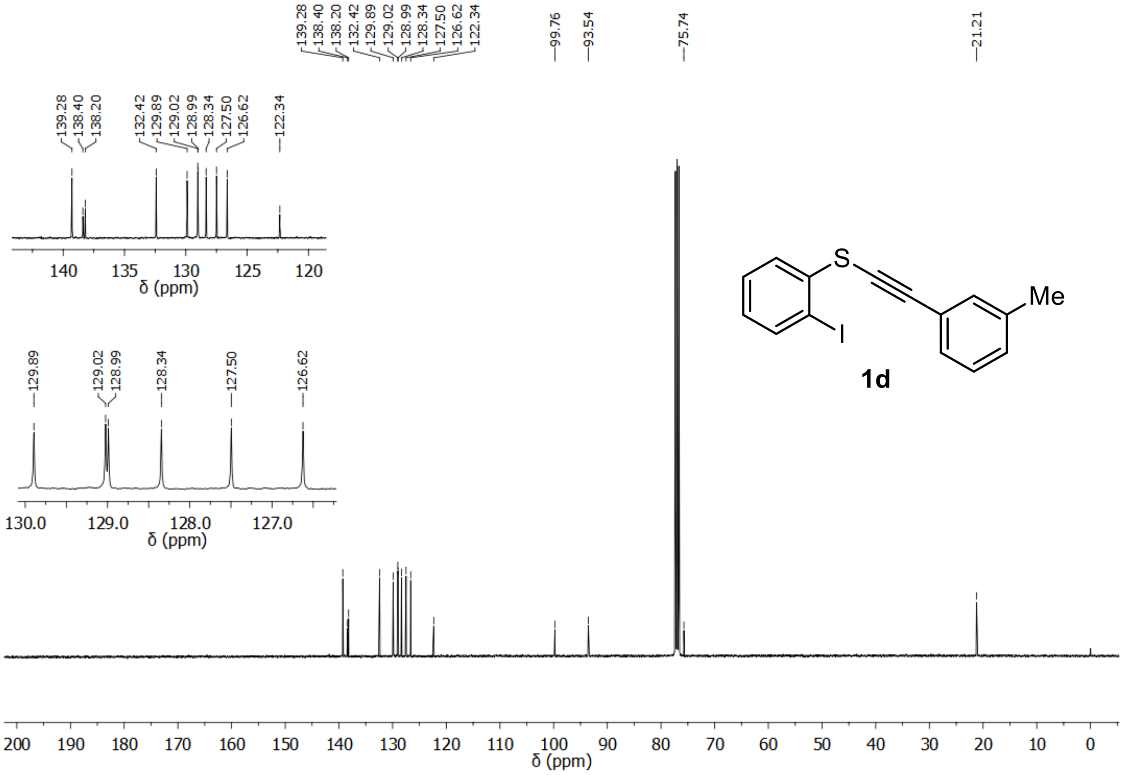

Figure S76. ^{13}C{^{1}H} NMR (100 MHz, $CDCl_3$) spectrum of **1d.**

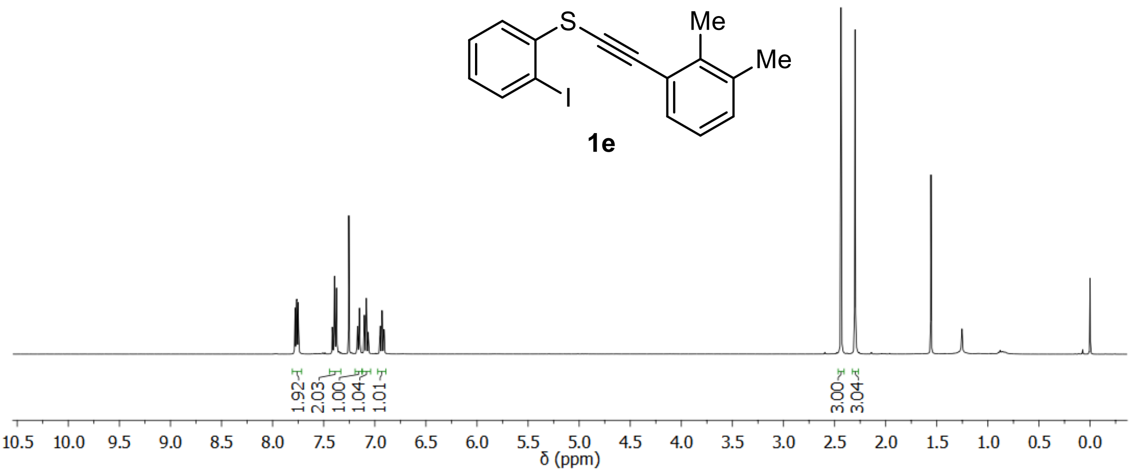

Figure S77. ^{1}H NMR (400 MHz, $CDCl_3$) spectrum of **1e.**

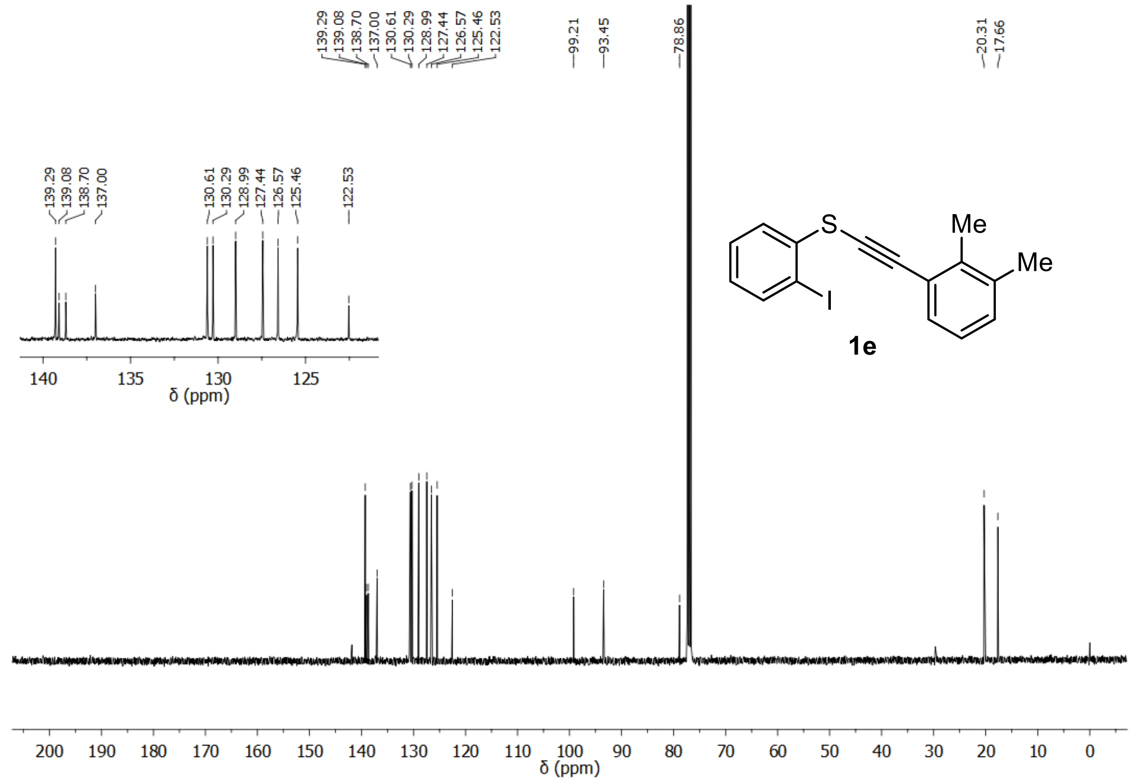

Figure S78. $^{13}C\{^1H\}$ NMR (100 MHz, $CDCl_3$) spectrum of **1e.**

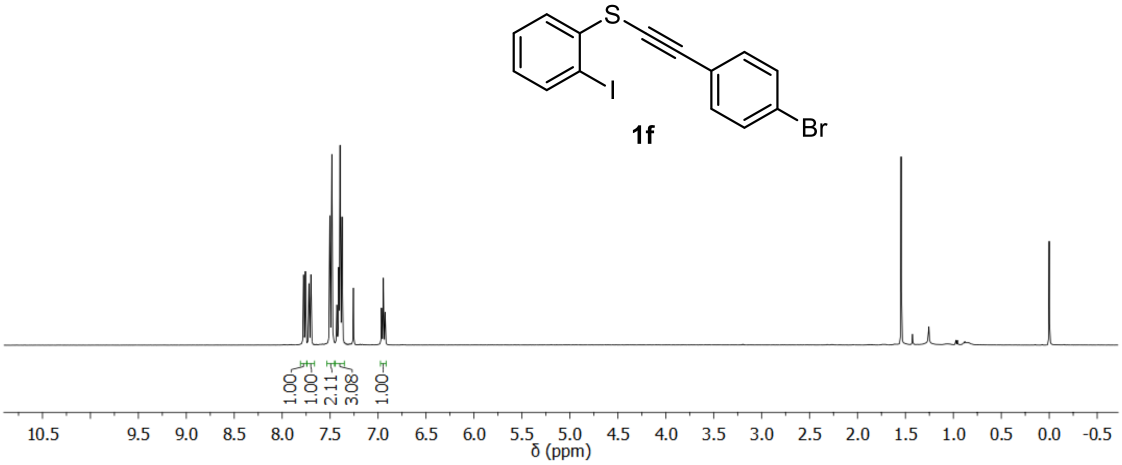

Figure S79. ^{1}H NMR (400 MHz, $CDCl_3$) spectrum of **1f.**

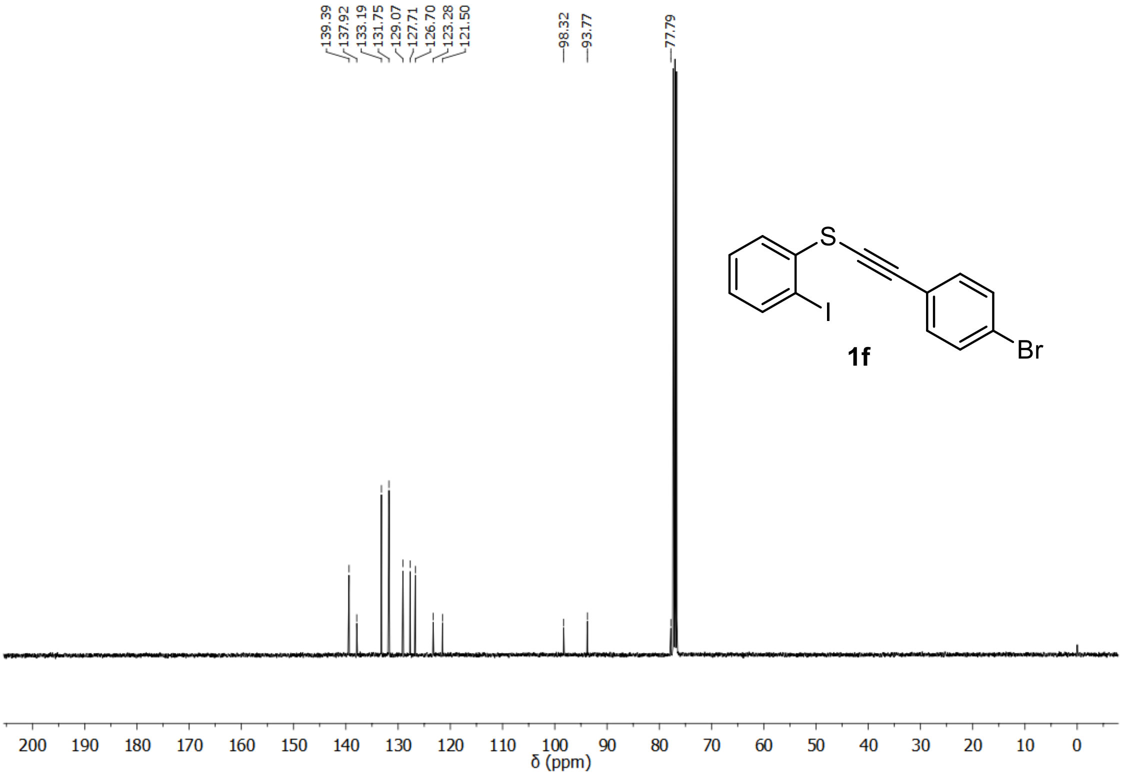

Figure S80. $^{13}C\{^{1}H\}$ NMR (100 MHz, $CDCl_3$) spectrum of **1f**.

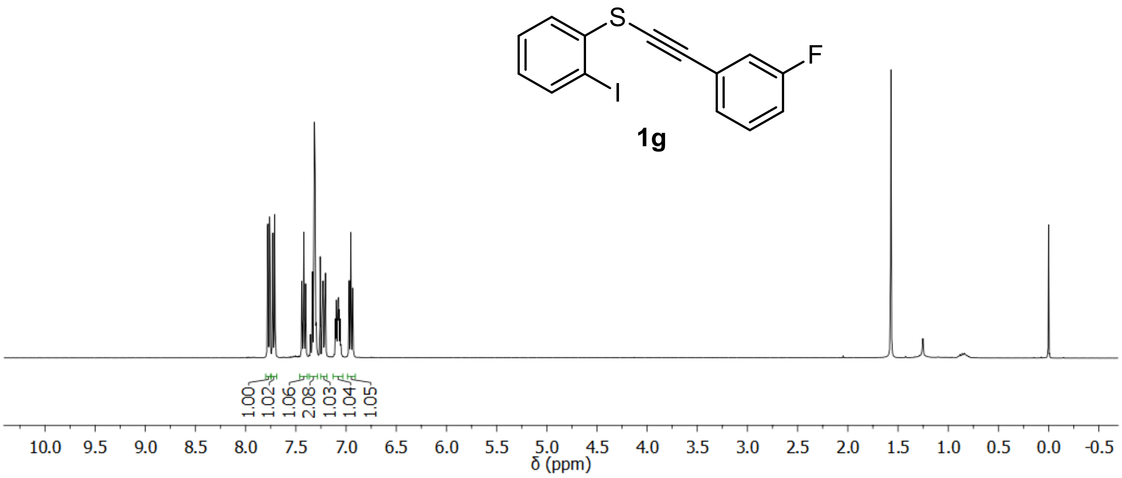

Figure S81. ^{1}H NMR (400 MHz, CDCl3) spectrum of **1g**.

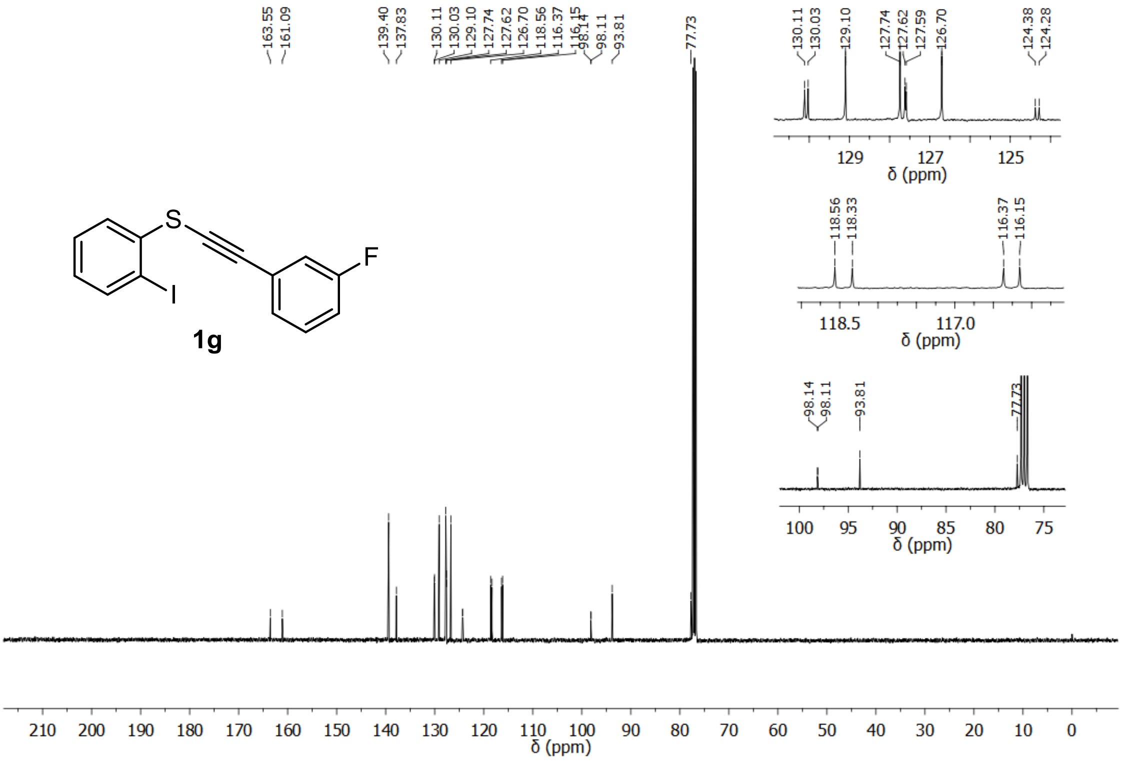

Figure S82. ^{13}C{^{1}H} NMR (100 MHz, CDCl3) spectrum of **1g.**

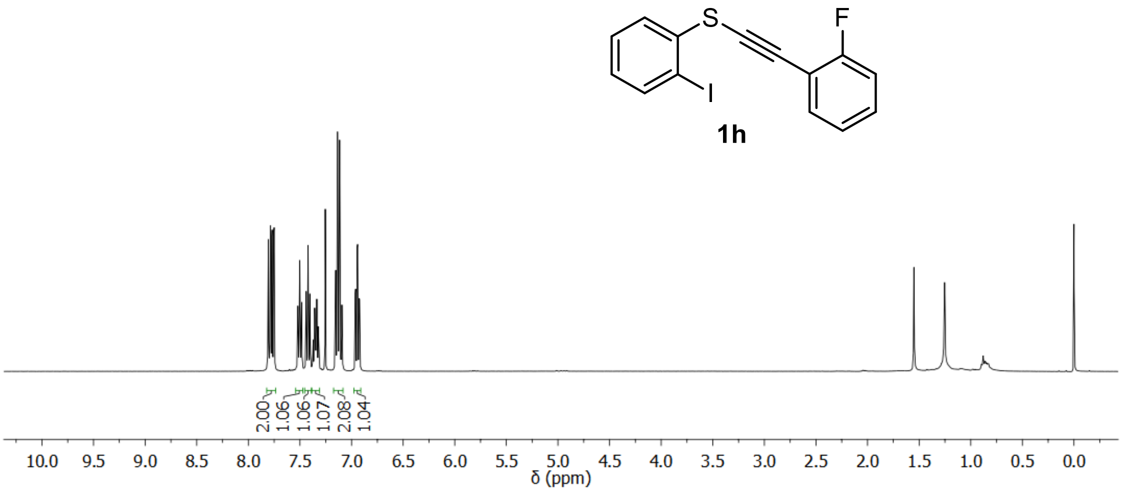

Figure S83. ^{1}H NMR (400 MHz, $CDCl_3$) spectrum of **1h**.

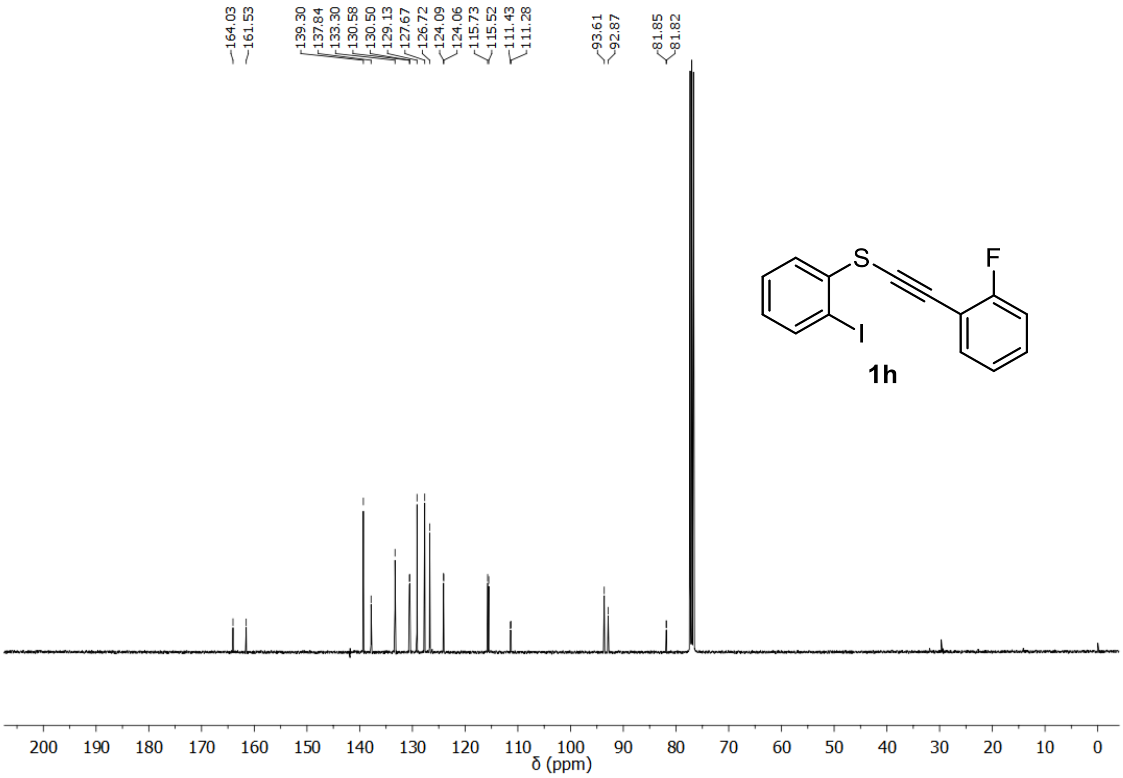

Figure S84. $^{13}C\{^{1}H\}$ NMR (100 MHz, $CDCl_3$) spectrum of **1h.**

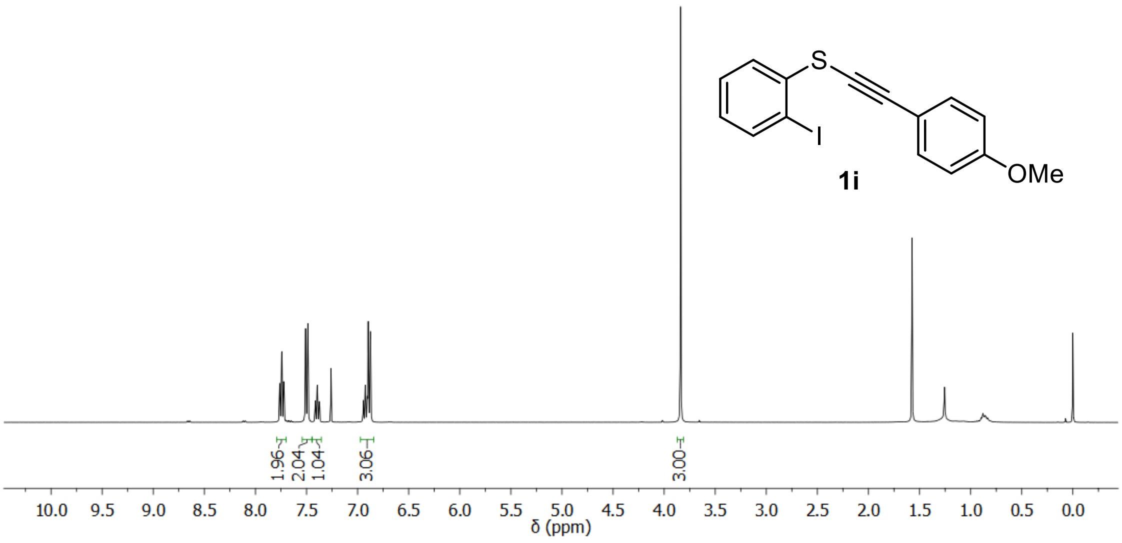

Figure S85. ^{1}H NMR (400 MHz, $CDCl_3$) spectrum of **1i**.

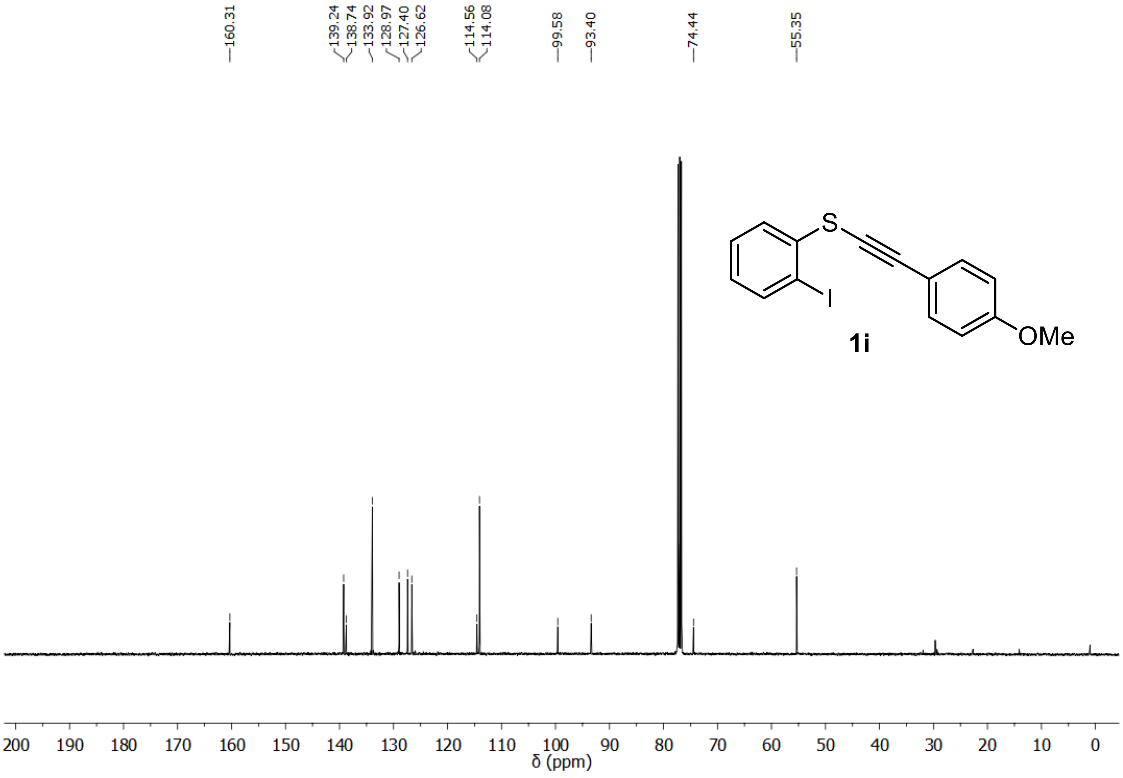

Figure S86. $^{13}C\{^{1}H\}$ NMR (100 MHz, $CDCl_3$) spectrum of **1i.**

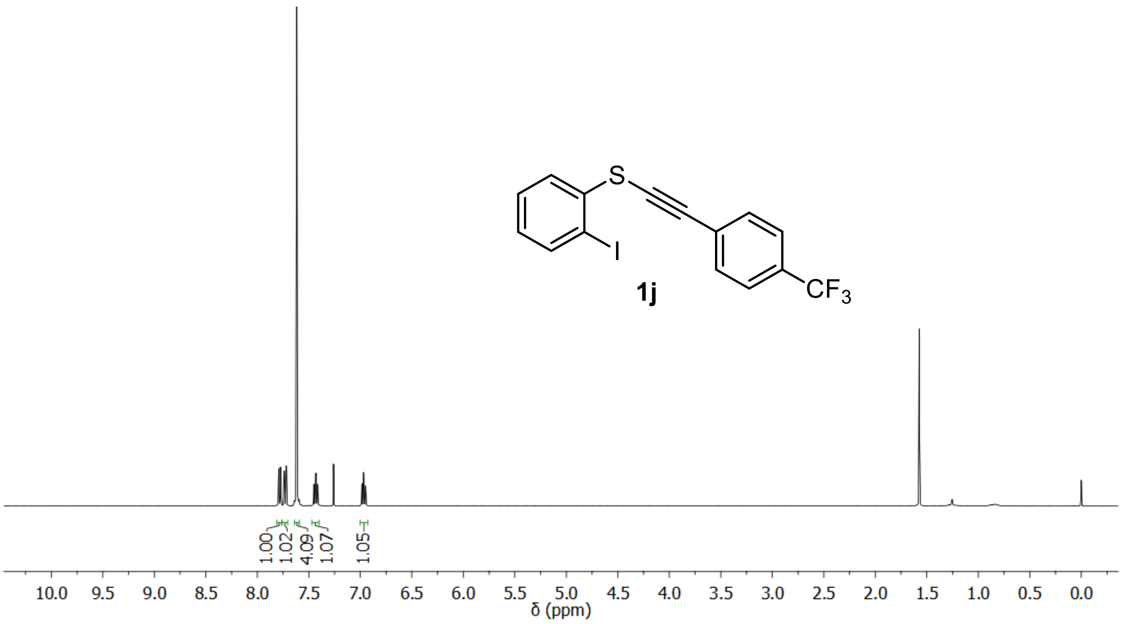

Figure S87. ^{1}H NMR (400 MHz, $CDCl_3$) spectrum of **1j**.

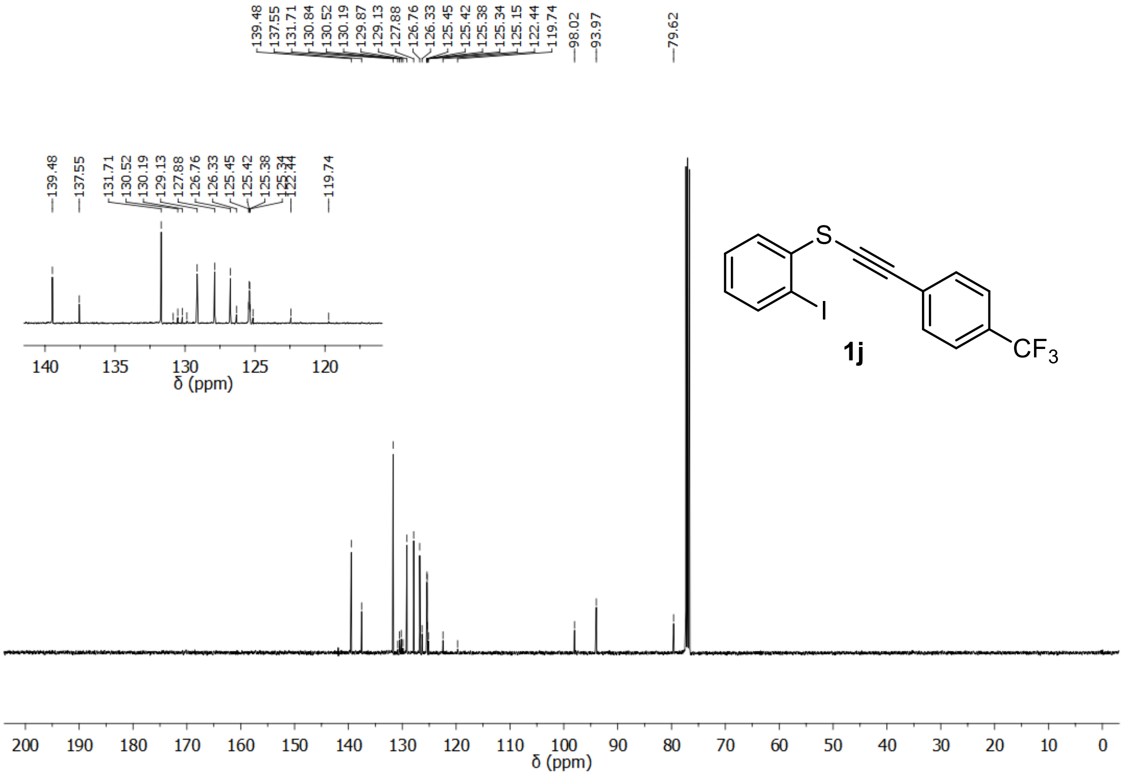

Figure S88. $^{13}C\{^{1}H\}$ NMR (100 MHz, $CDCl_3$) spectrum of **1j**.

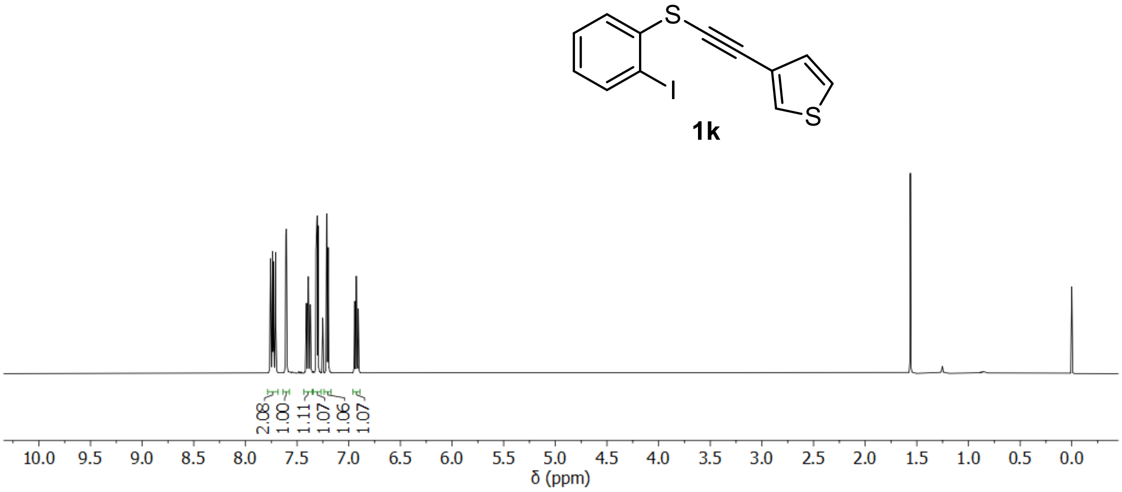

Figure S89. ^{1}H NMR (400 MHz, $CDCl_3$) spectrum of **1k**.

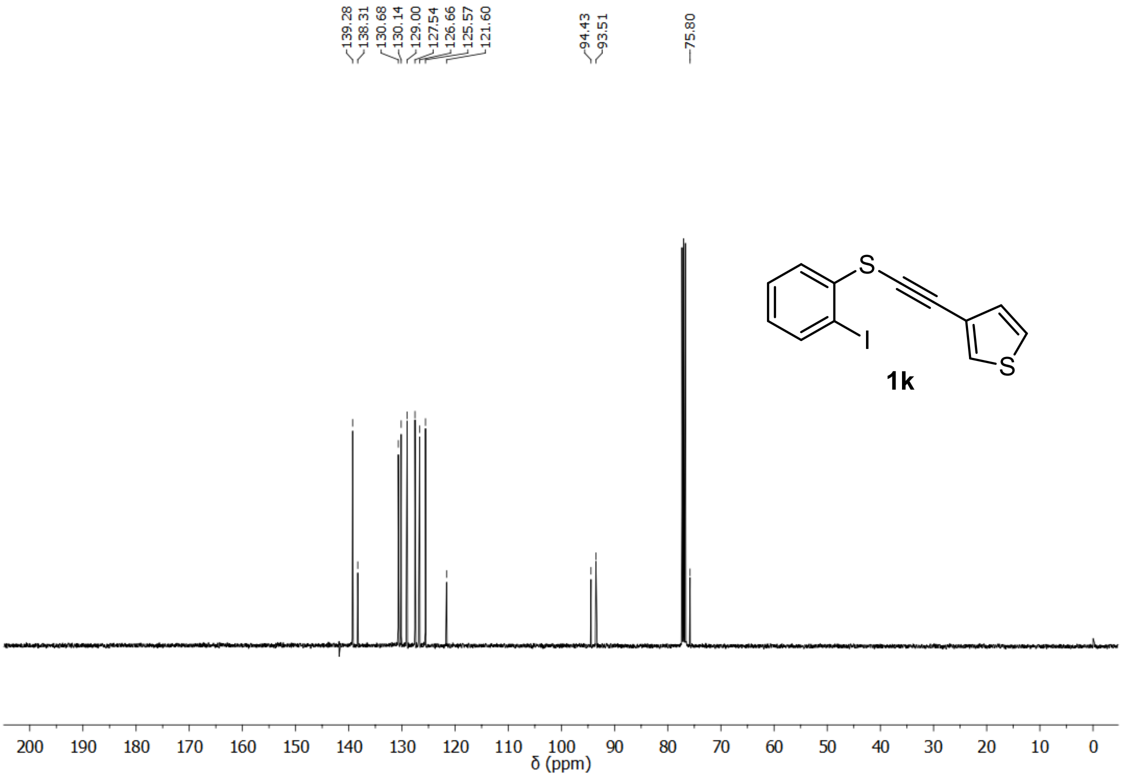

Figure S90. $^{13}C\{^1H\}$ NMR (100 MHz, $CDCl_3$) spectrum of **1k**.

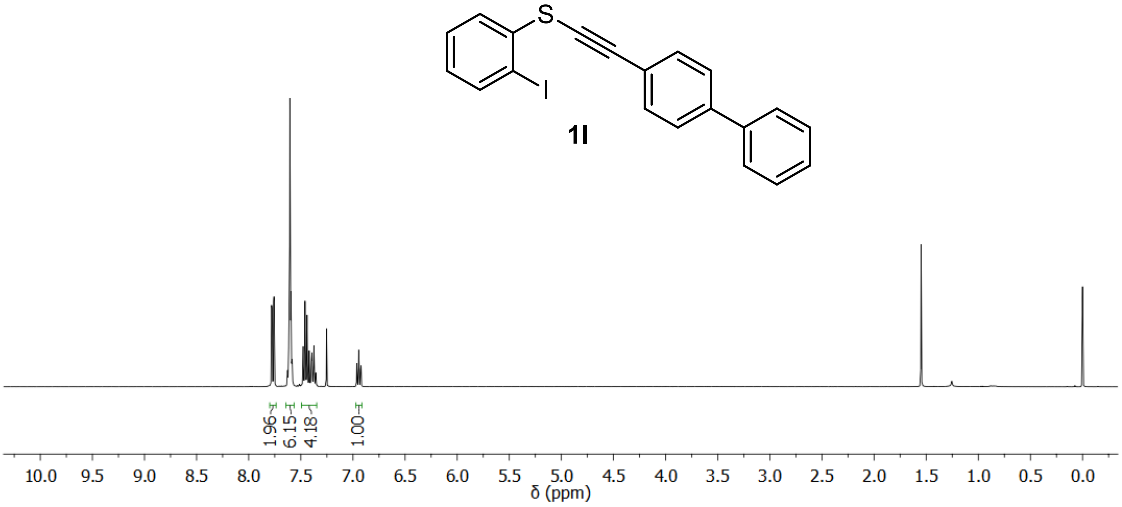

Figure S91. ^{1}H NMR (400 MHz, $CDCl_3$) spectrum of **1l**.

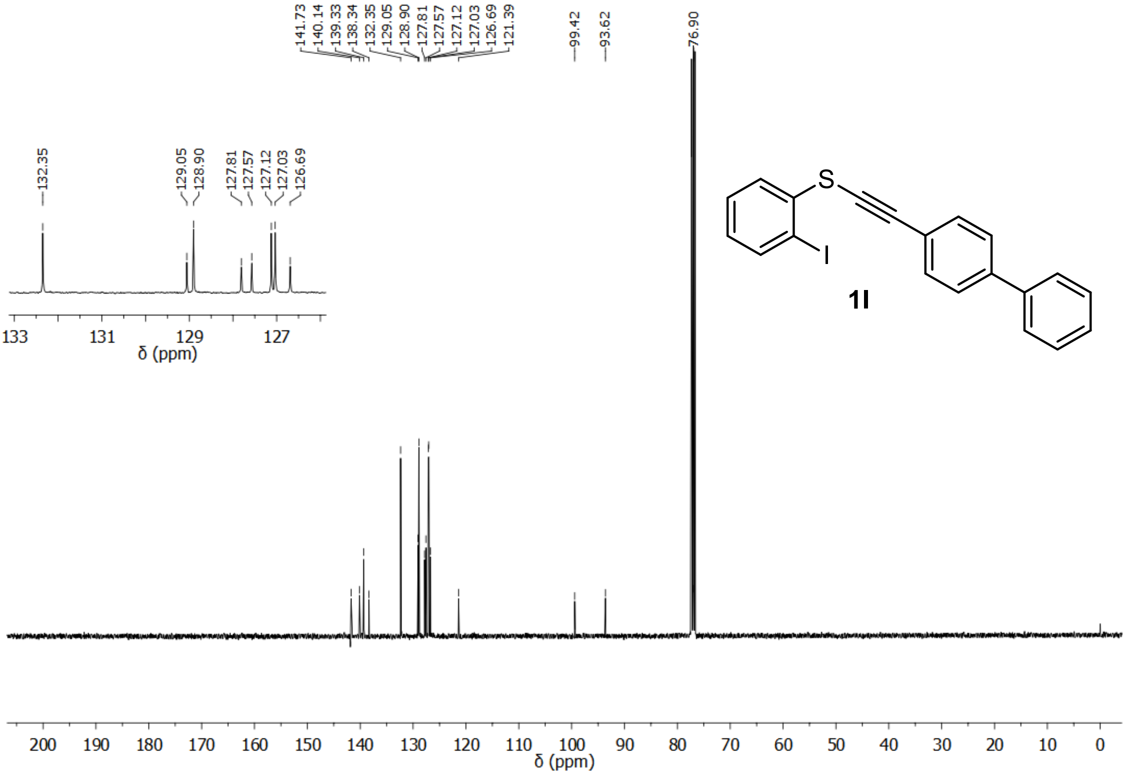

Figure S92. $^{13}C\{^{1}H\}$ NMR (100 MHz, $CDCl_3$) spectrum of **1l**.

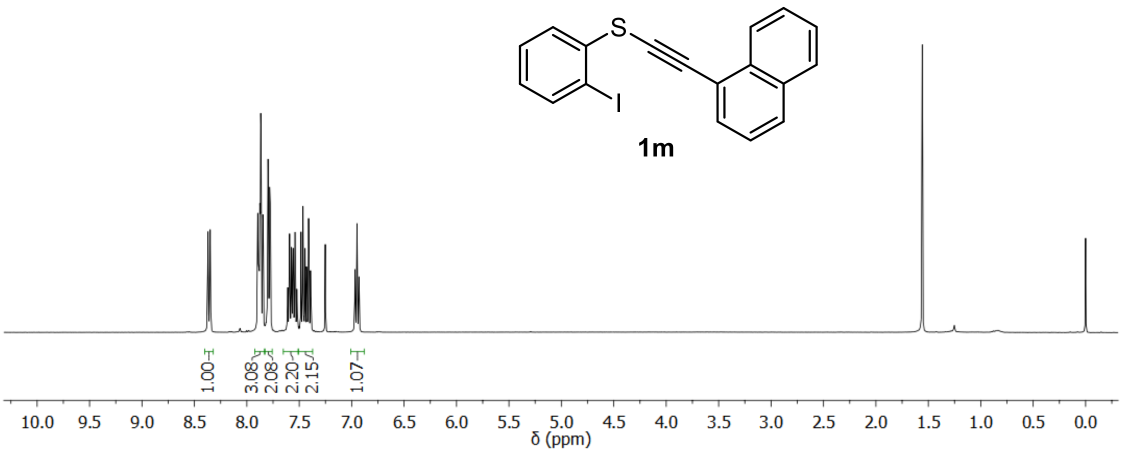

Figure S93. ^{1}H NMR (400 MHz, $CDCl_3$) spectrum of **1m**.

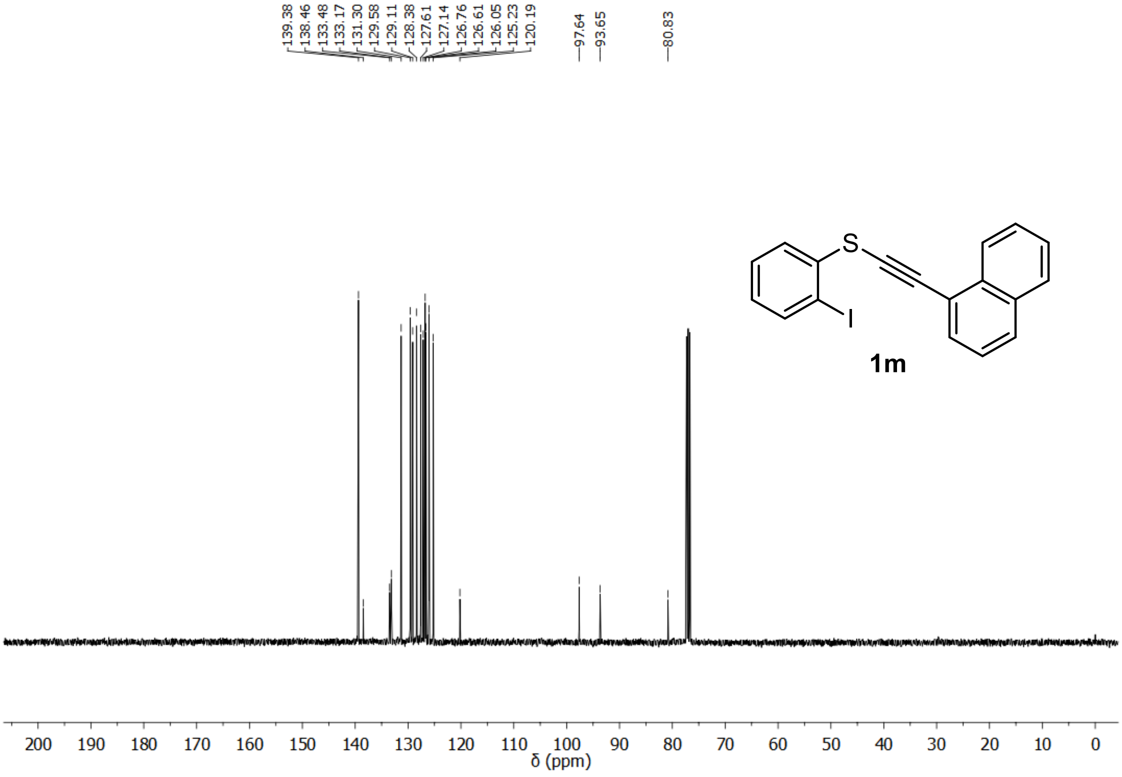

Figure S94. ^{13}C{^{1}H} NMR (100 MHz, $CDCl_3$) spectrum of **1m**.

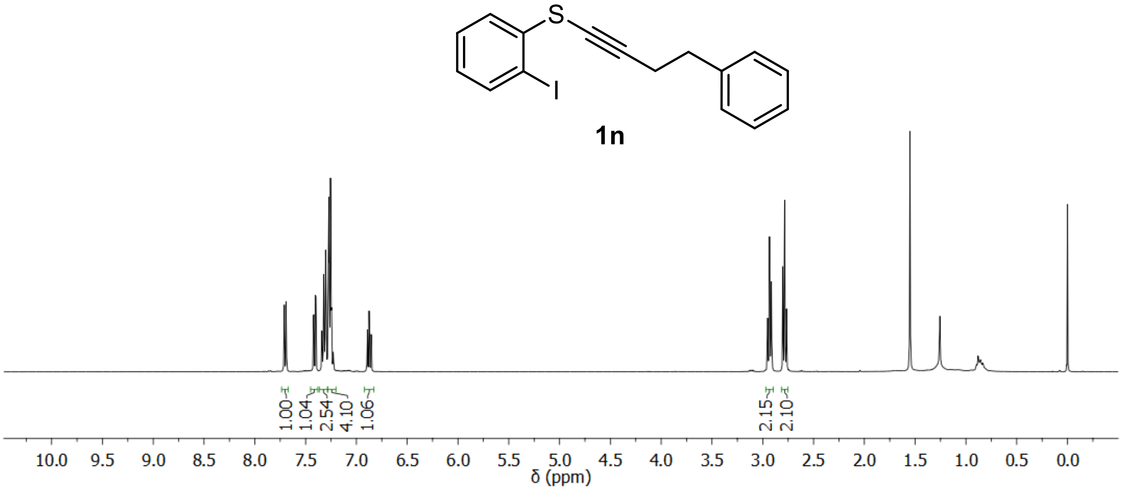

Figure S95. ^{1}H NMR (400 MHz, $CDCl_3$) spectrum of **1n**.

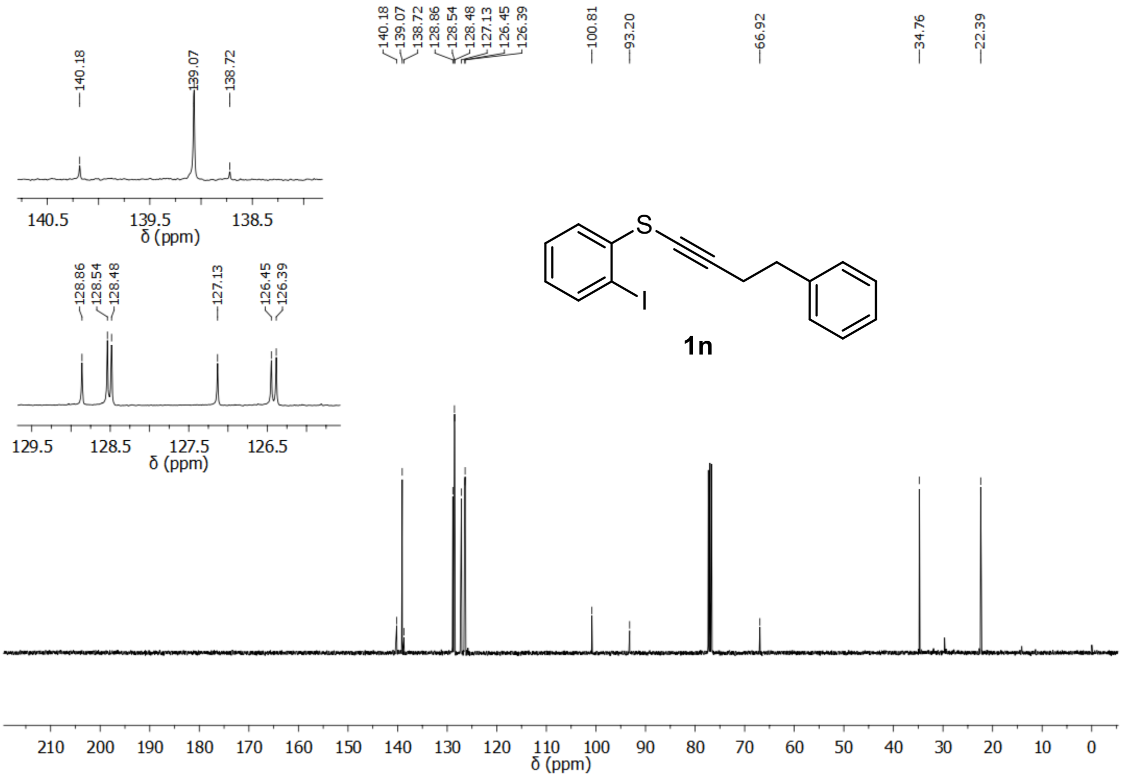

Figure S96. $^{13}C\{^{1}H\}$ NMR (100 MHz, $CDCl_3$) spectrum of **1n**.

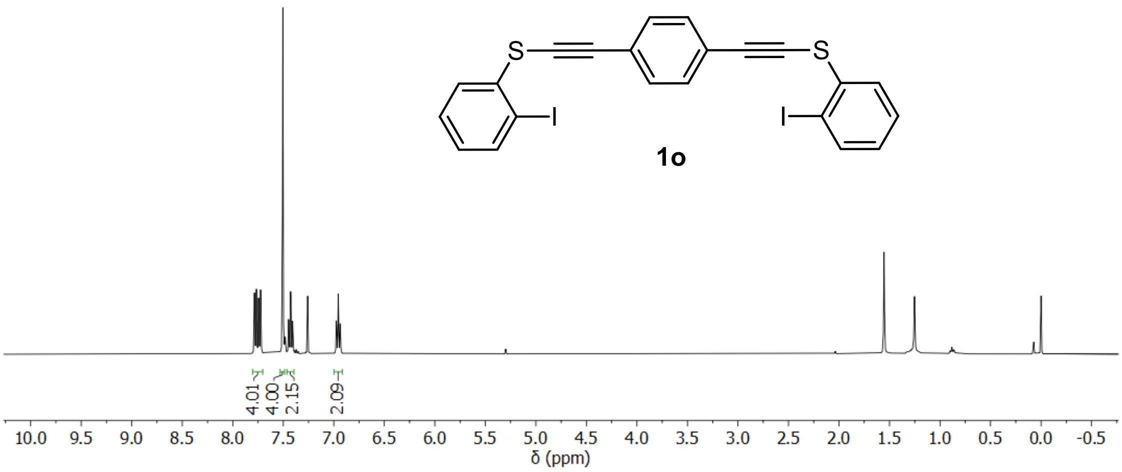

Figure S97. ^{1}H NMR (400 MHz, $CDCl_3$) spectrum of **1o**.

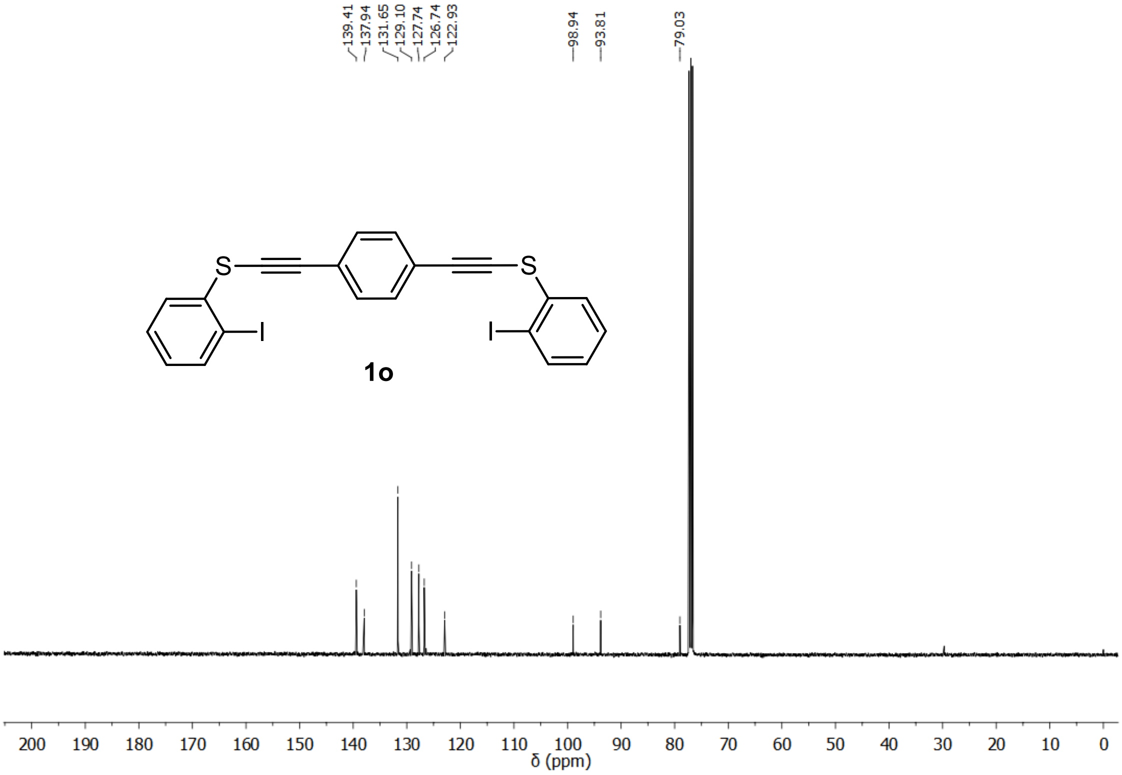

Figure S98. $^{13}C\{^{1}H\}$ NMR (100 MHz, $CDCl_3$) spectrum of **1o**.

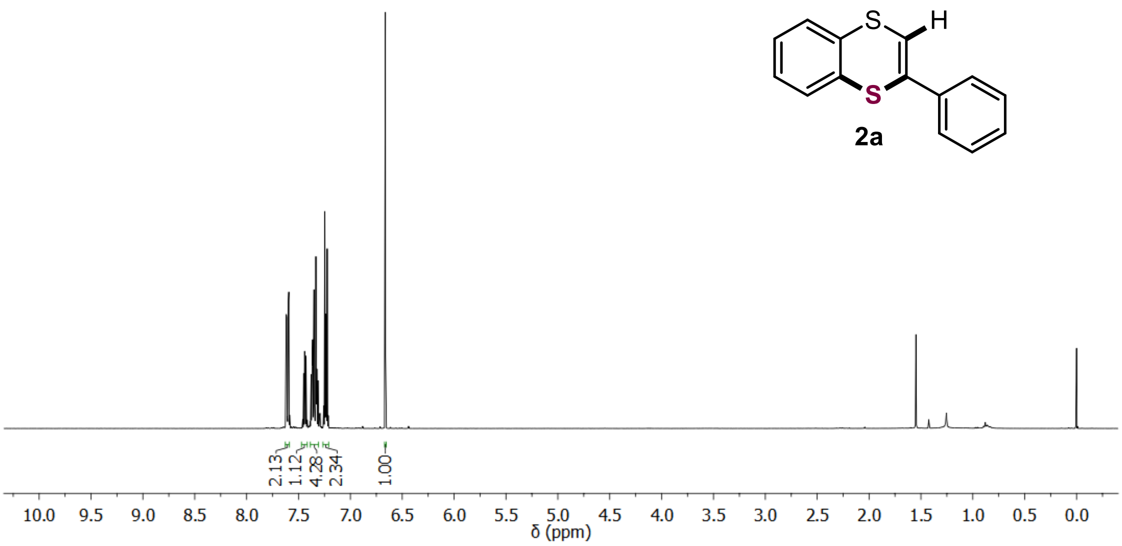

Figure S99. ^{1}H NMR (400 MHz, $CDCl_3$) spectrum of **2a.**

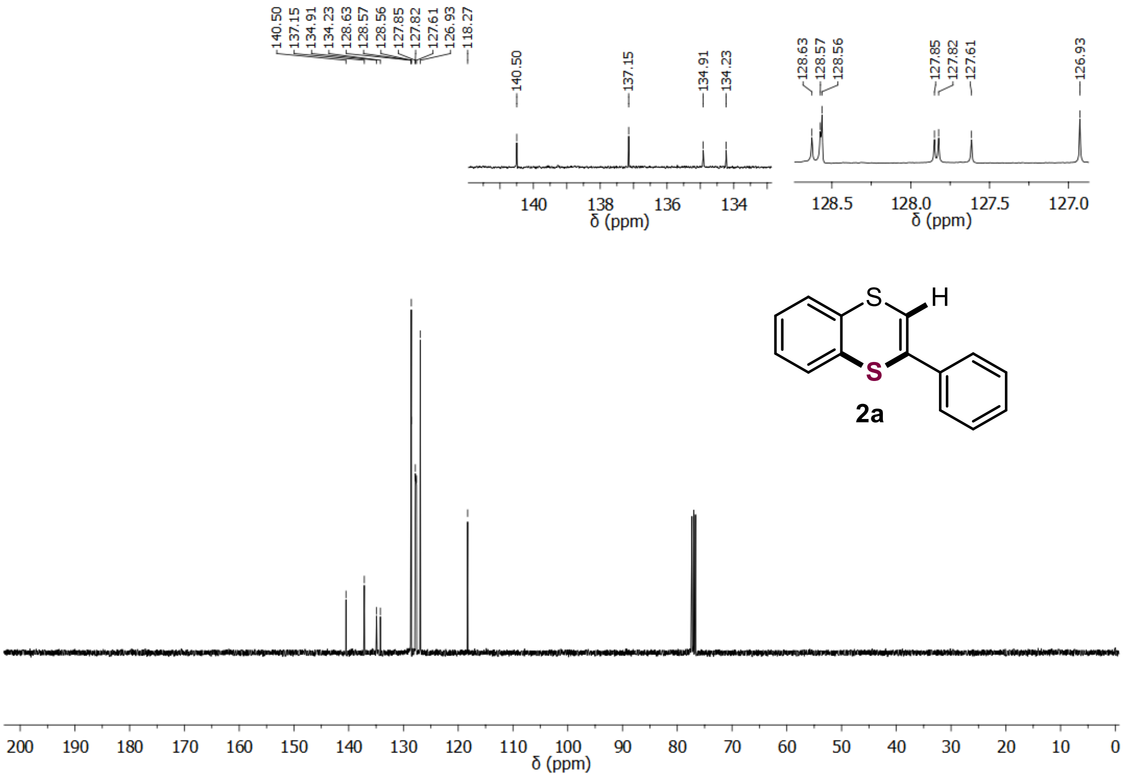

Figure S100. $^{13}C\{^{1}H\}$ NMR (100 MHz, $CDCl_3$) spectrum of **2a.**

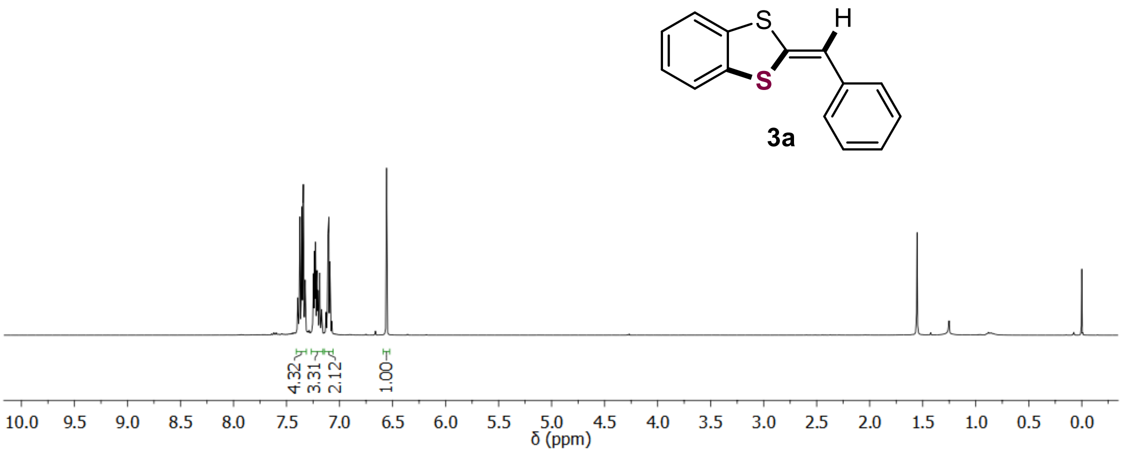

Figure S101. ^{1}H NMR (400 MHz, $CDCl_3$) spectrum of **3a.**

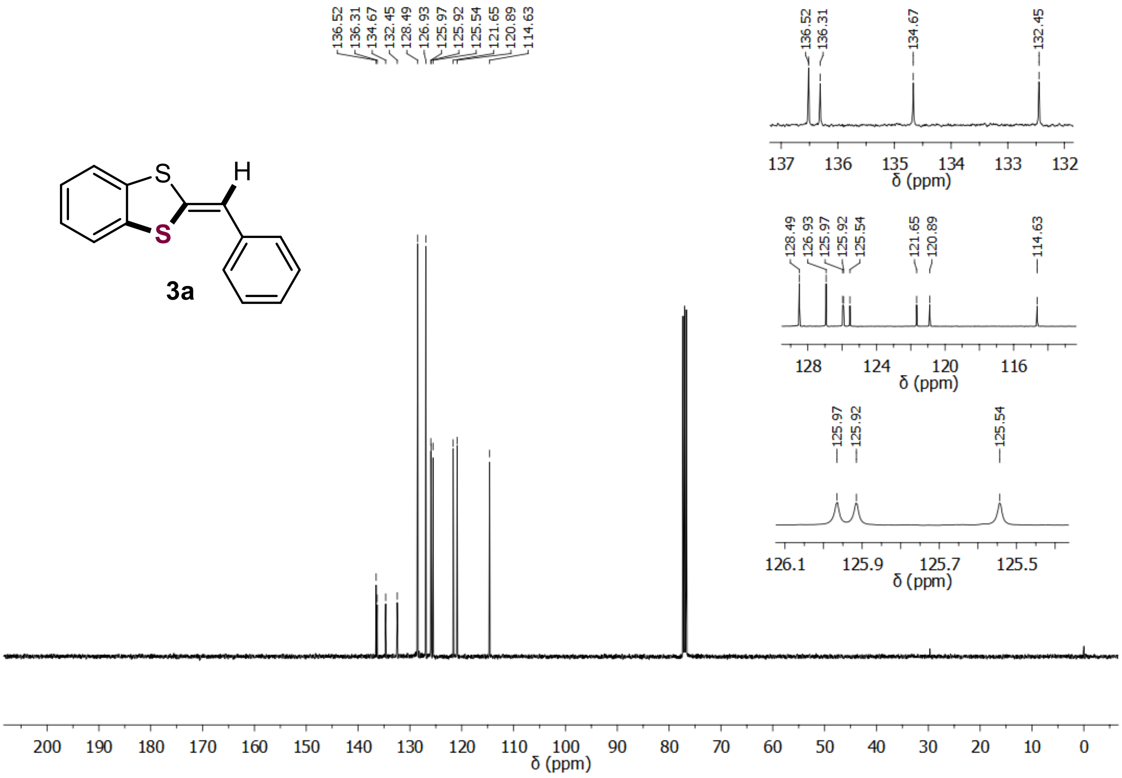

Figure S102. $^{13}C\{^{1}H\}$ NMR (100 MHz, $CDCl_3$) spectrum of **3a.**

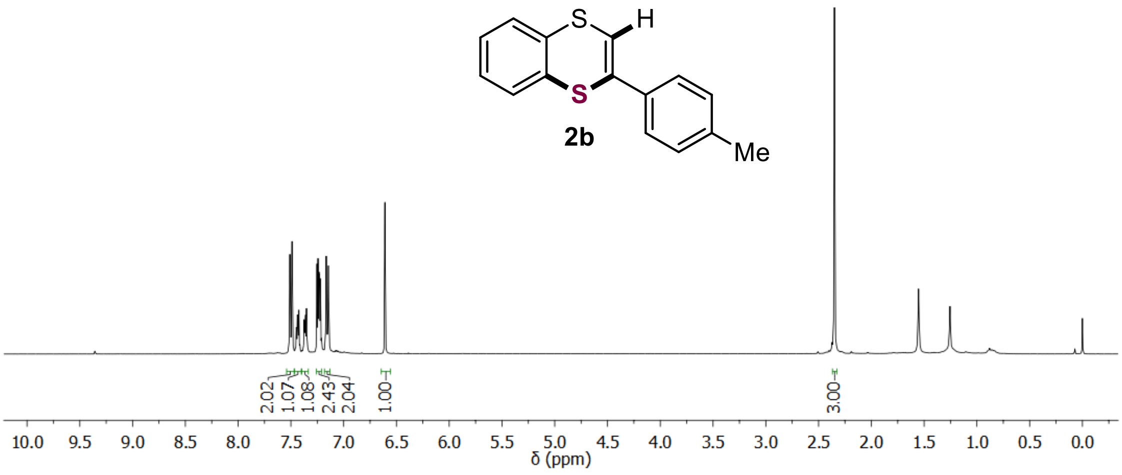

Figure S103. ^{1}H NMR (400 MHz, $CDCl_3$) spectrum of **2b.**

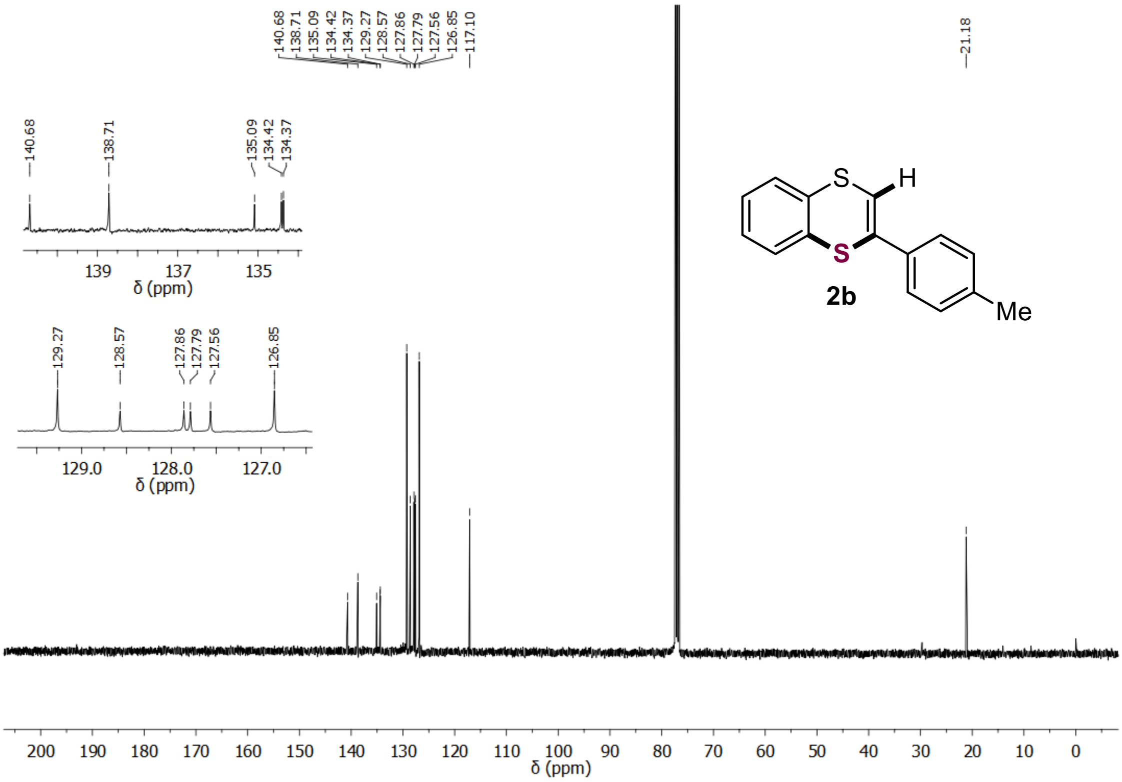

Figure S104. ^{13}C{^{1}H} NMR (100 MHz, $CDCl_3$) spectrum of **2b.**

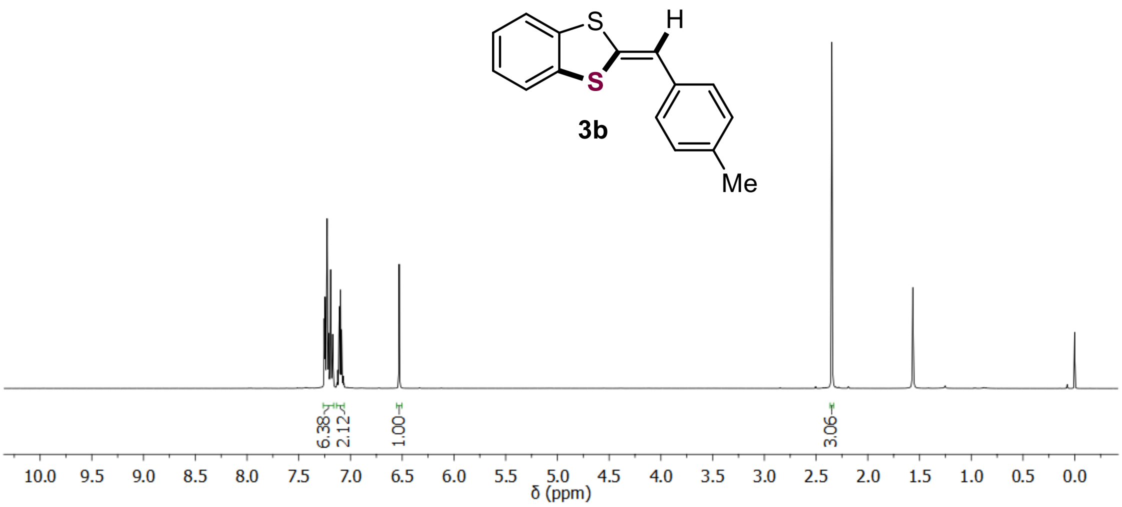

Figure S105. ^{1}H NMR (400 MHz, $CDCl_3$) spectrum of **3b.**

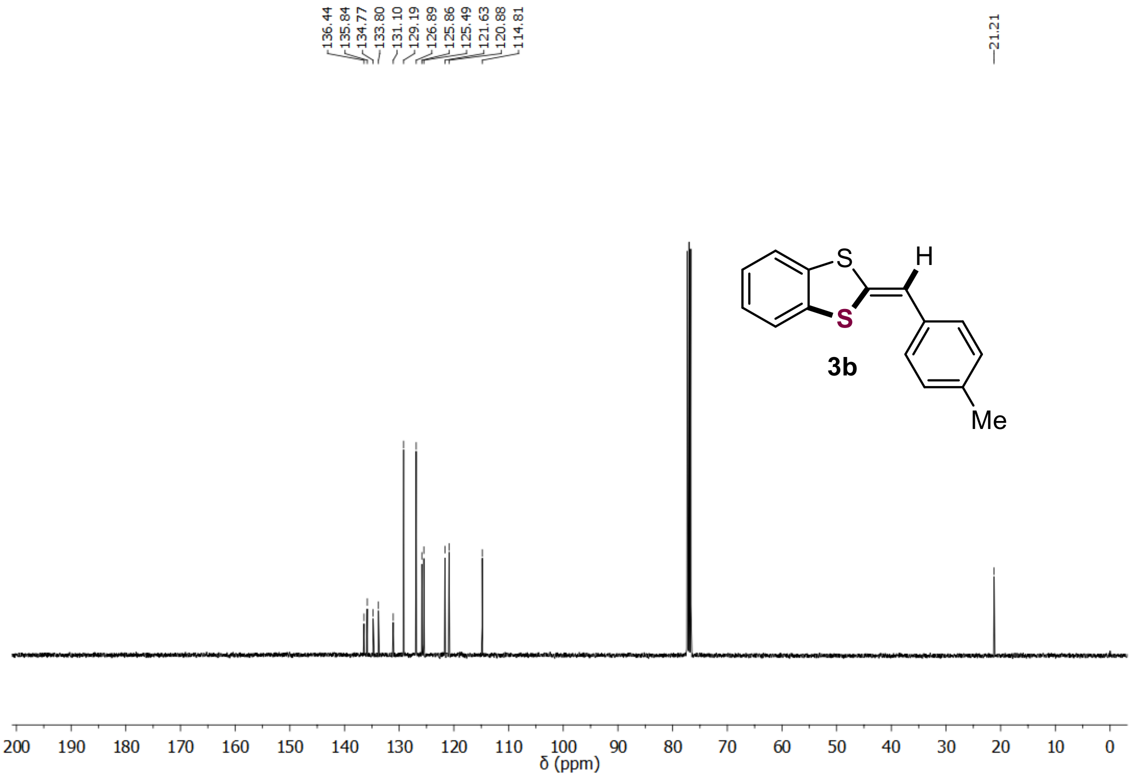

Figure S106. $^{13}C\{^{1}H\}$ NMR (100 MHz, $CDCl_3$) spectrum of **3b.**

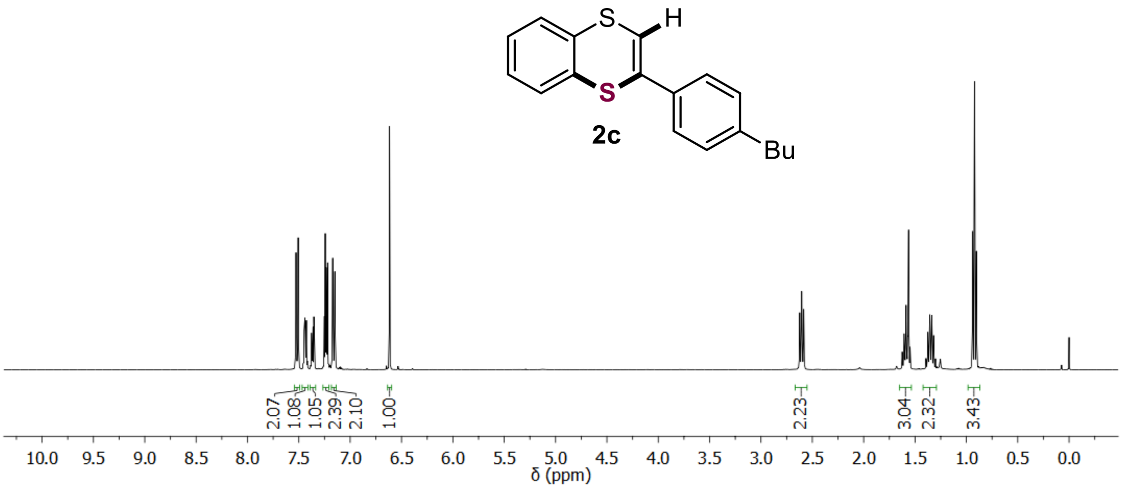

Figure S107. ^{1}H NMR (400 MHz, $CDCl_3$) spectrum of **2c.**

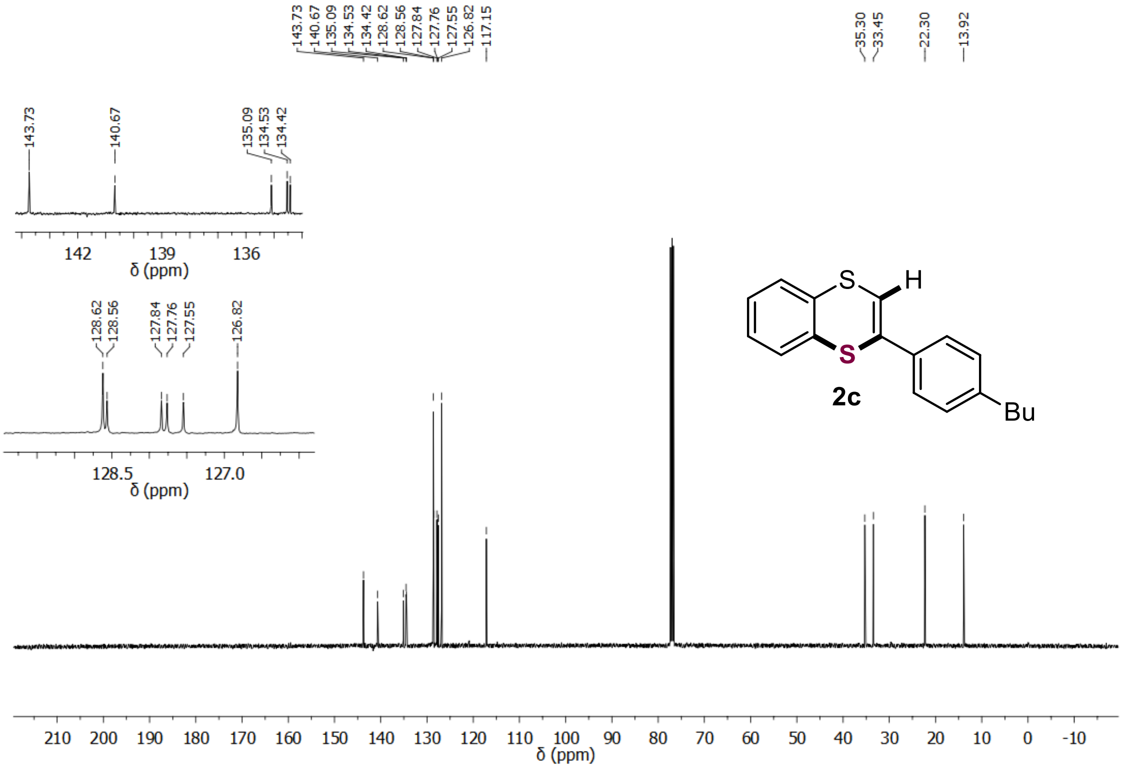

Figure S108. ^{1}H NMR (400 MHz, $CDCl_3$) spectrum of **2c.**

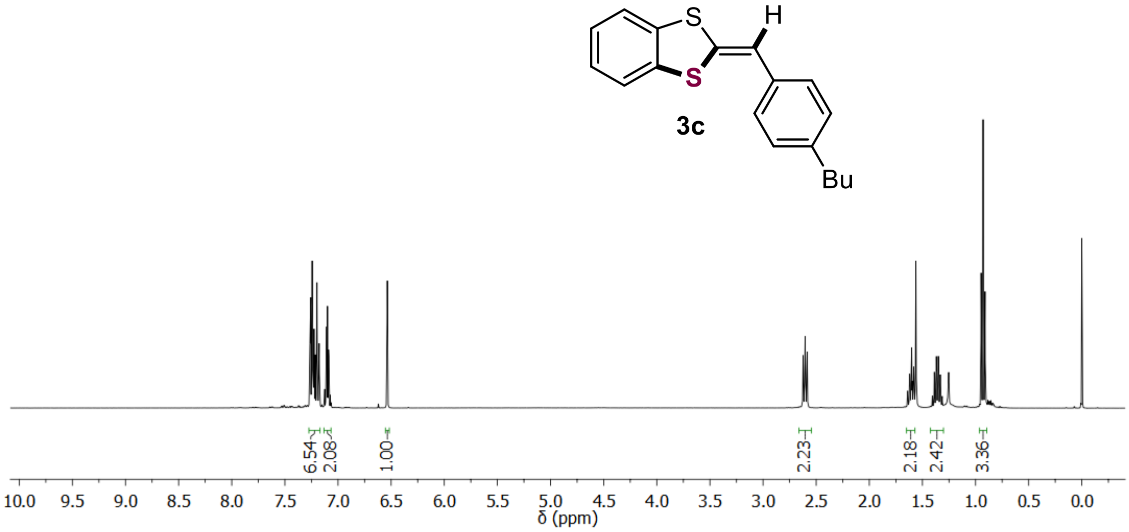

Figure S109. ^{1}H NMR (400 MHz, $CDCl_3$) spectrum of **3c.**

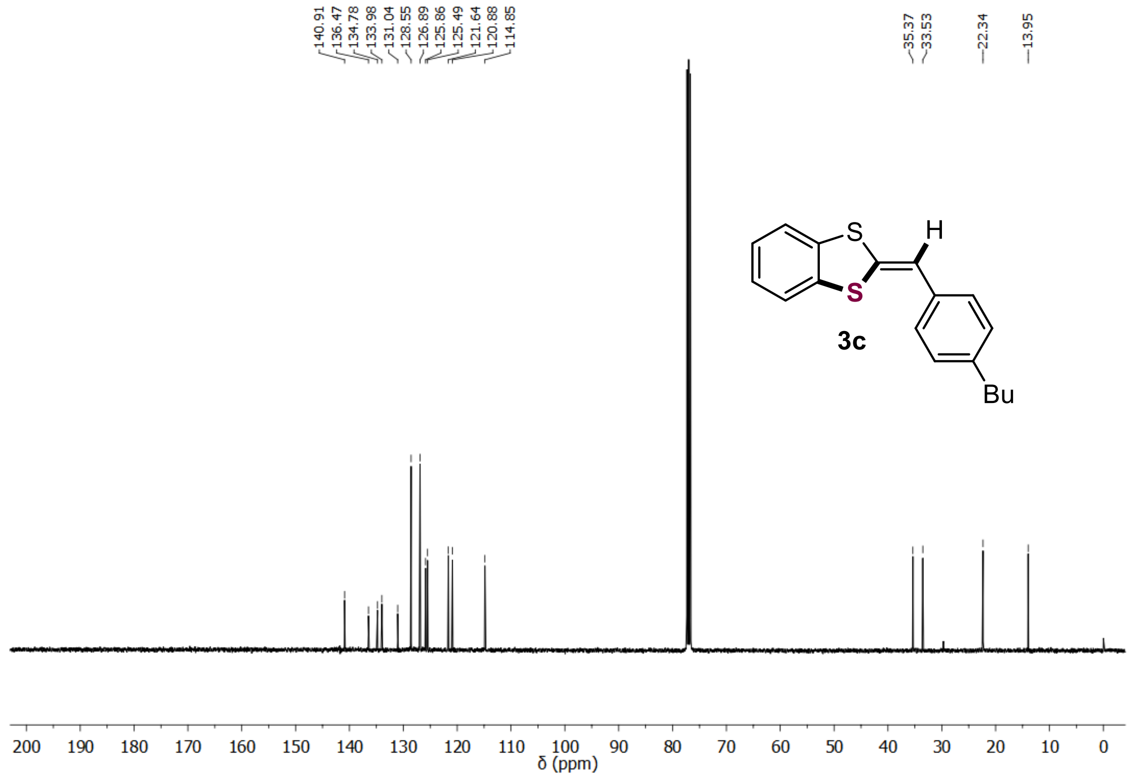

Figure S110. $^{13}C\{^1H\}$ NMR (100 MHz, $CDCl_3$) spectrum of **3c.**

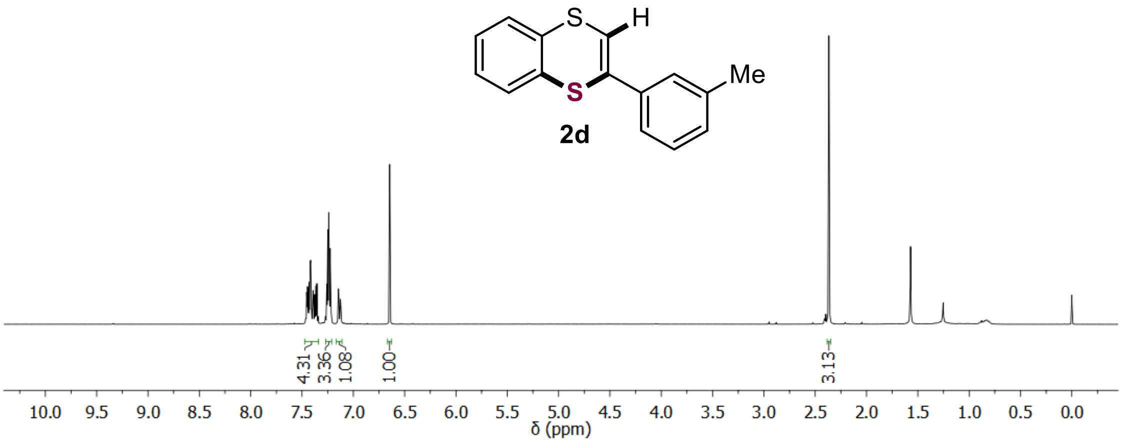

Figure S111. ^{1}H NMR (400 MHz, CDCl$_3$) spectrum of **2d.**

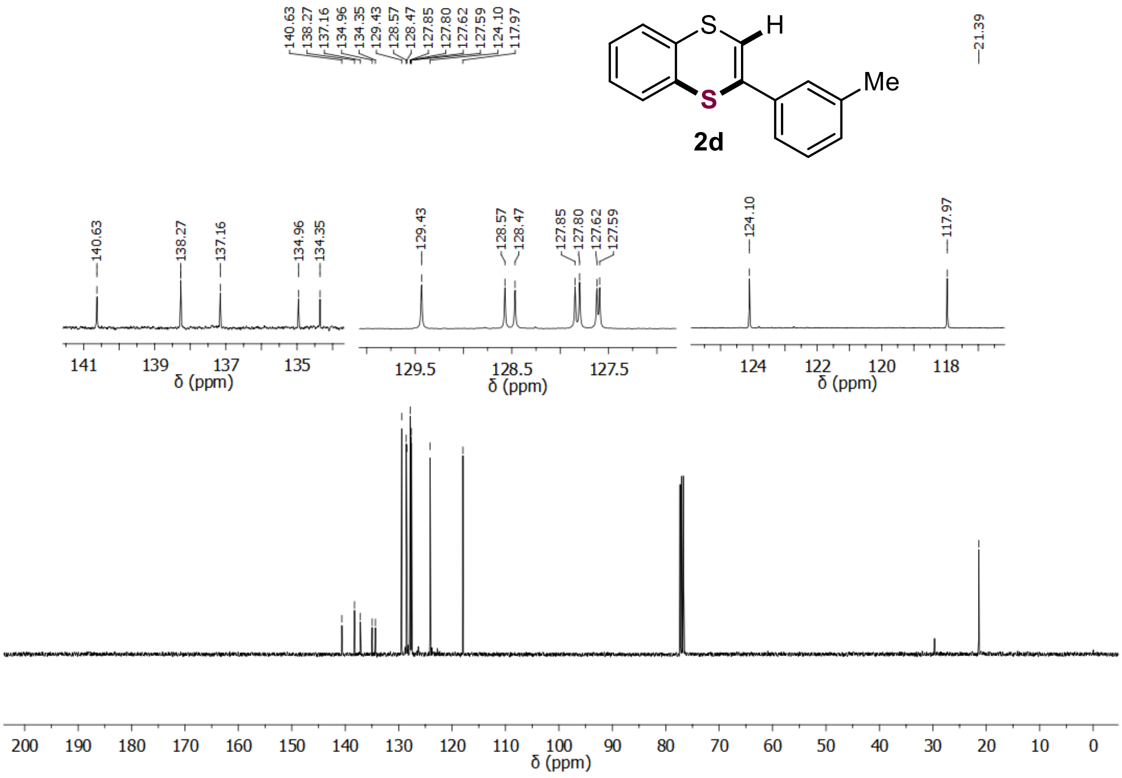

Figure S112. ^{13}C{^{1}H} NMR (100 MHz, CDCl$_3$) spectrum of **2d.**

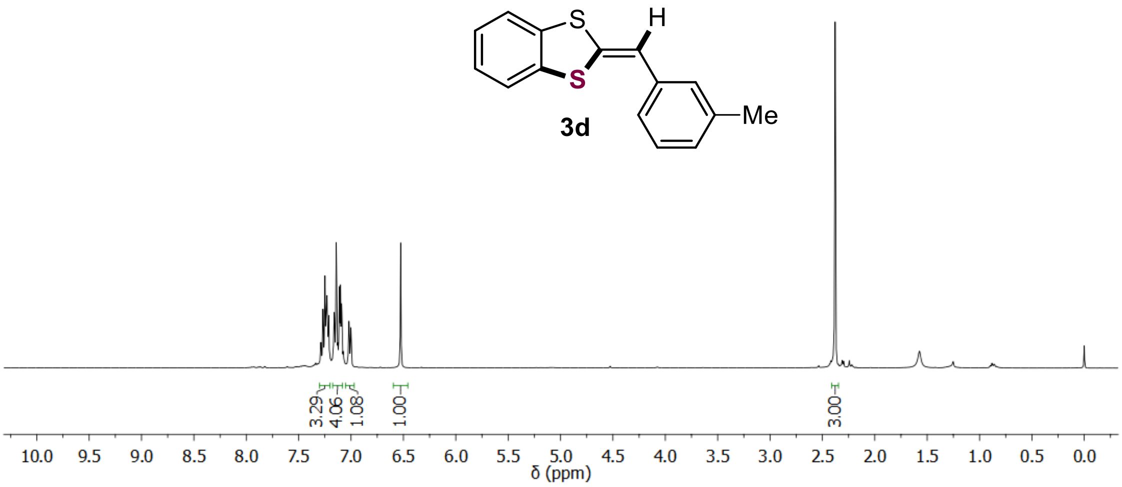

Figure S113. ^{1}H NMR (400 MHz, $CDCl_3$) spectrum of **3d.**

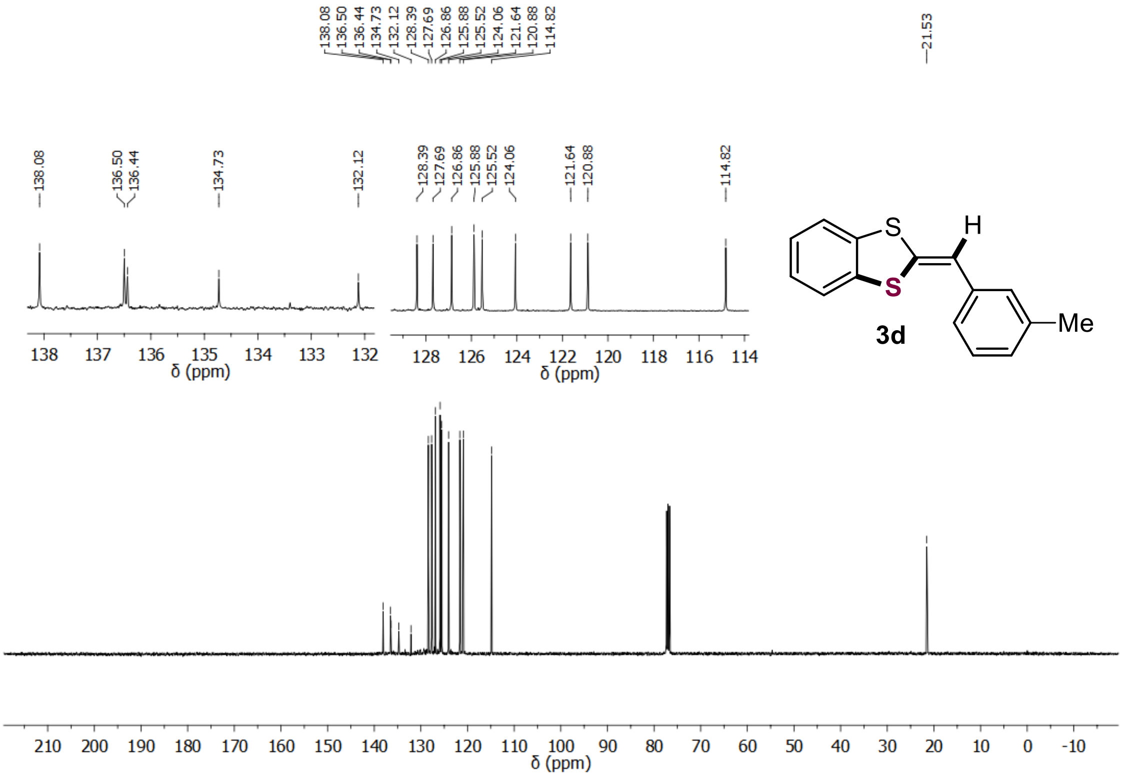

Figure S114. $^{13}C\{^{1}H\}$ NMR (100 MHz, $CDCl_3$) spectrum of **3d.**

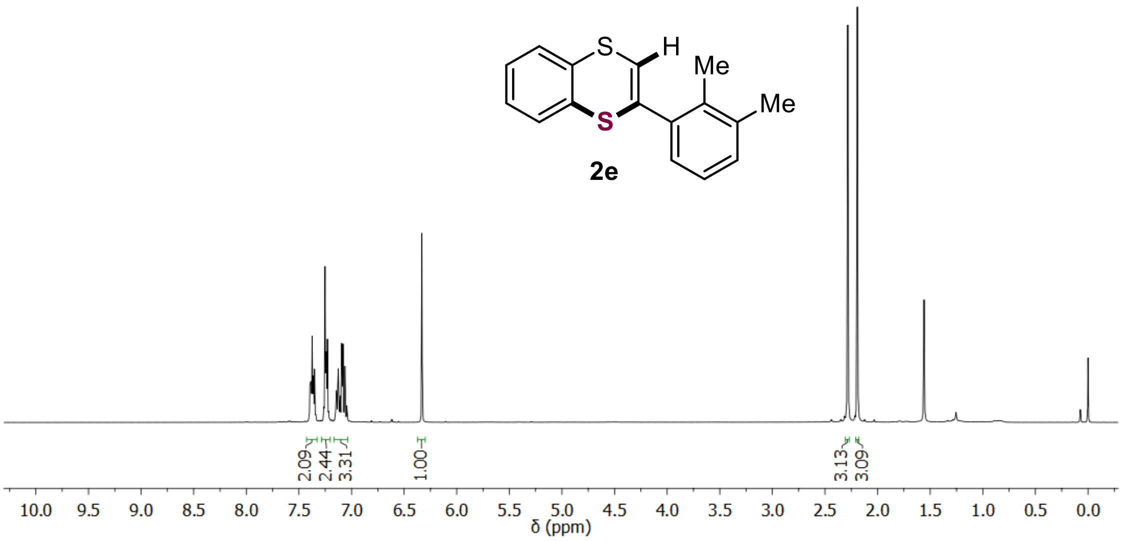

Figure S115. ^{1}H NMR (400 MHz, CDCl$_3$) spectrum of **2e.**

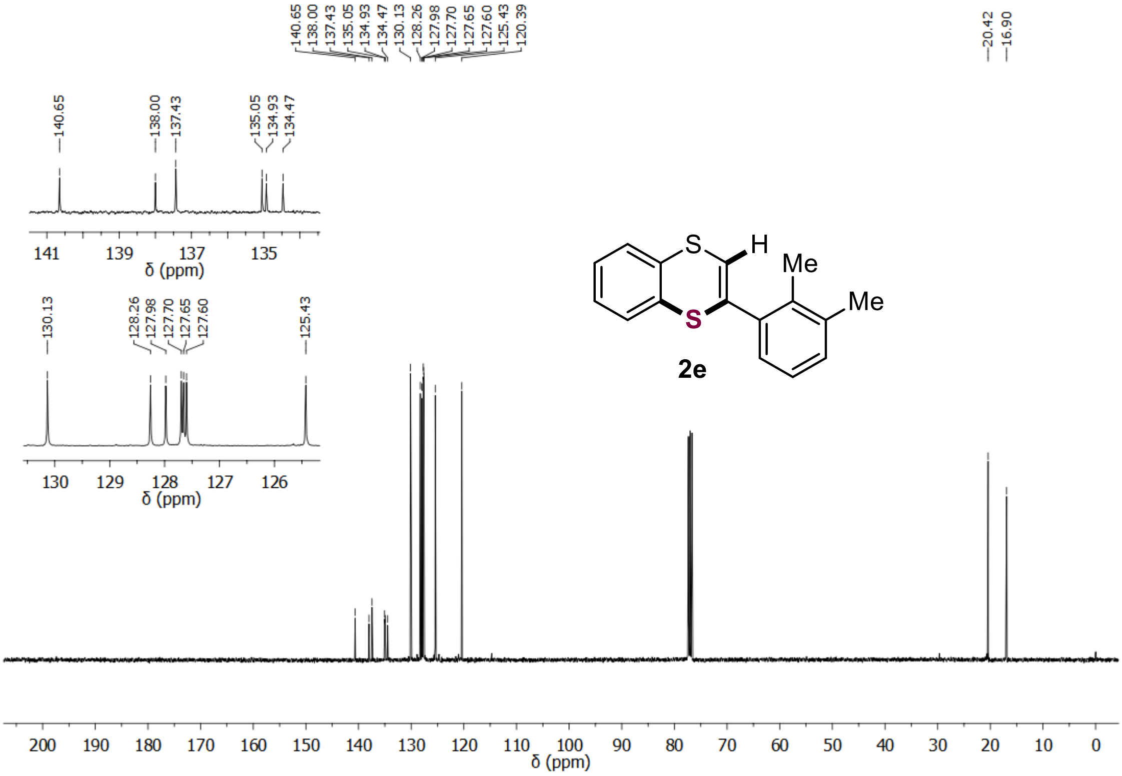

Figure S116. ^{13}C{^{1}H} NMR (100 MHz, CDCl$_3$) spectrum of **2e.**

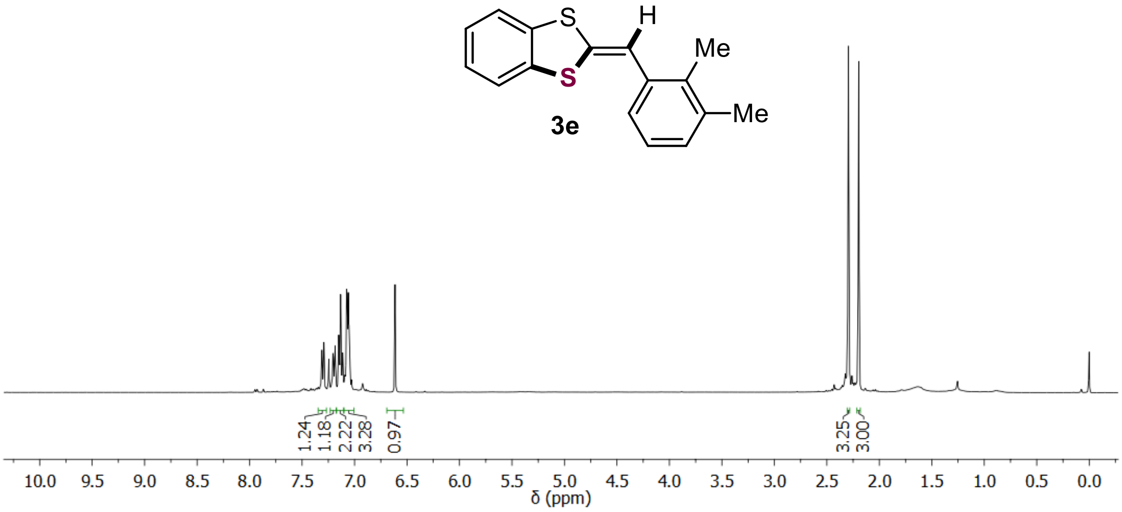

Figure S117. ^{1}H NMR (400 MHz, $CDCl_3$) spectrum of **3e.**

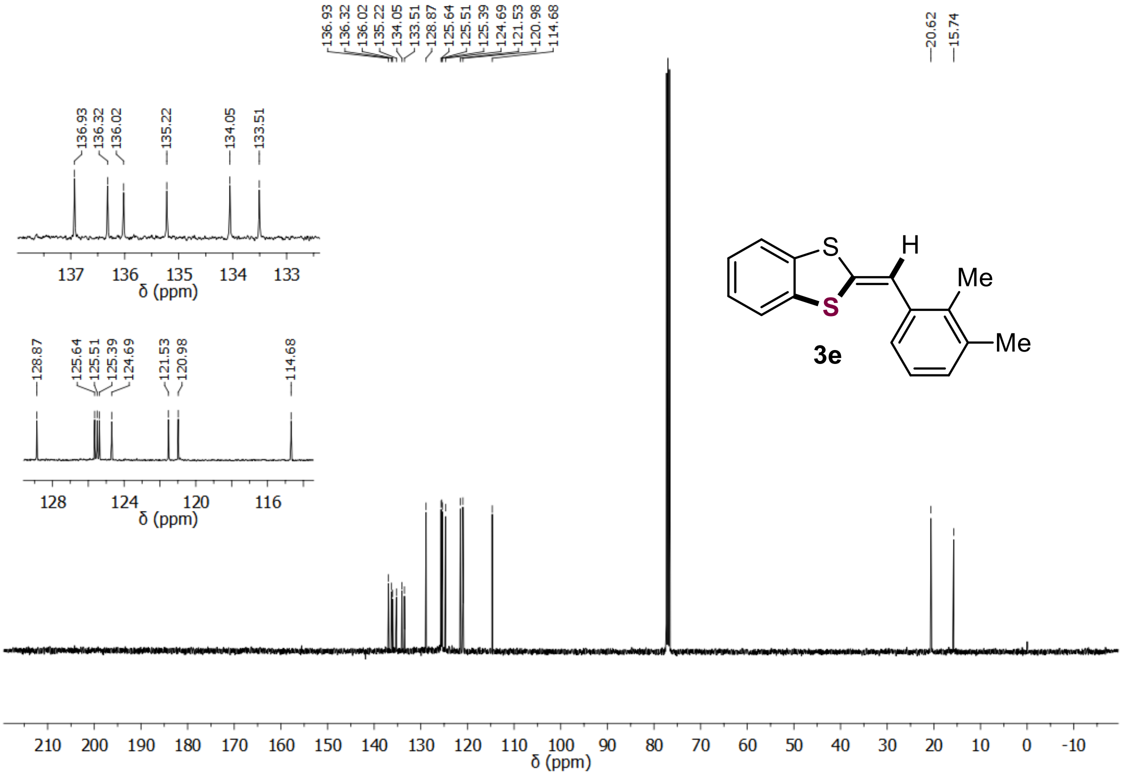

Figure S118. $^{13}C\{^{1}H\}$ NMR (100 MHz, $CDCl_3$) spectrum of **3e**.

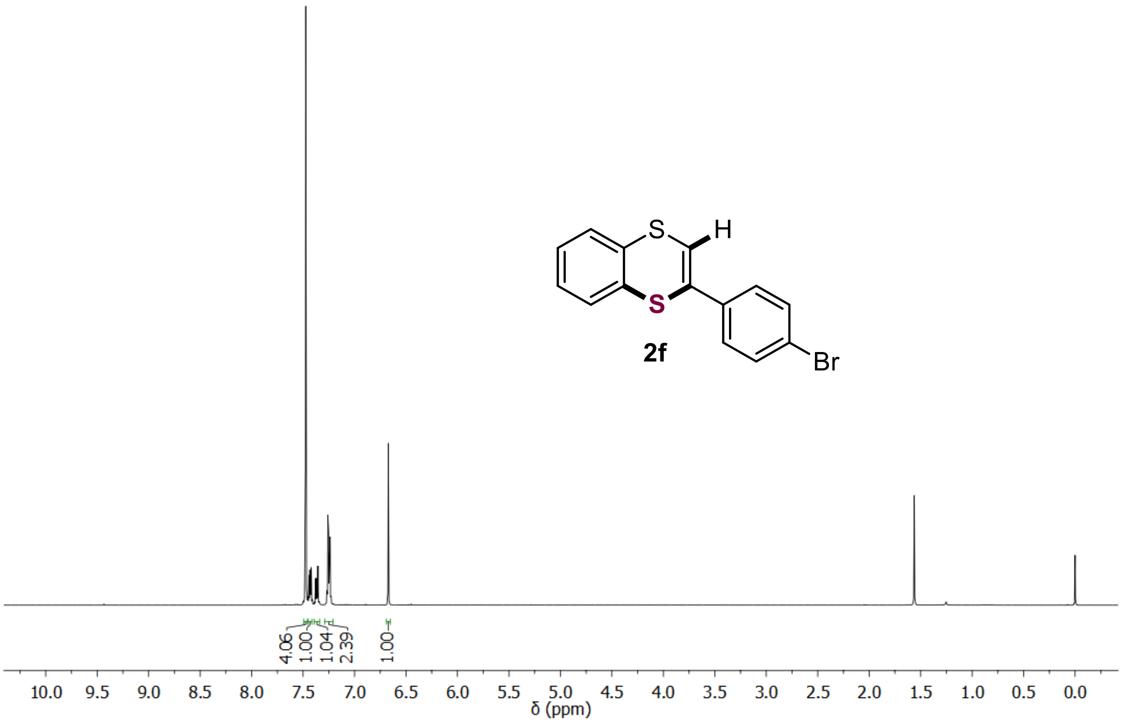

Figure S119. ^{1}H NMR (400 MHz, $CDCl_3$) spectrum of **2f**.

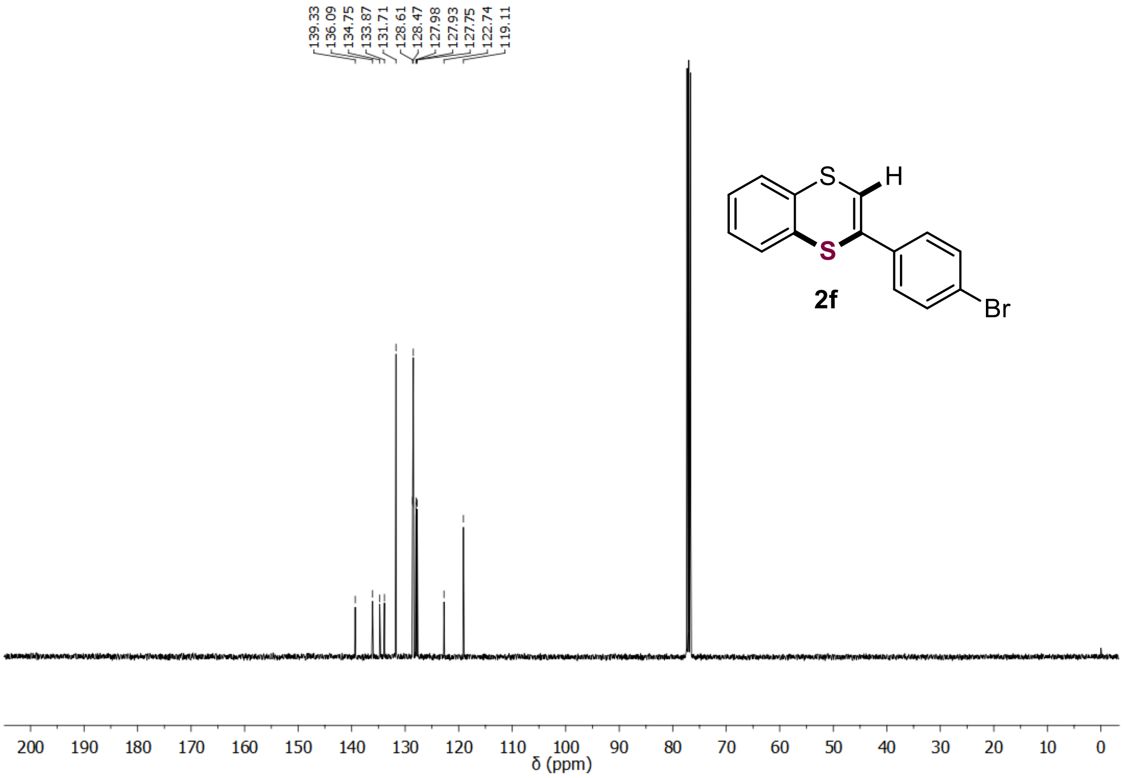

Figure S120. $^{13}C\{^1H\}$ NMR (100 MHz, $CDCl_3$) spectrum of **2f**.

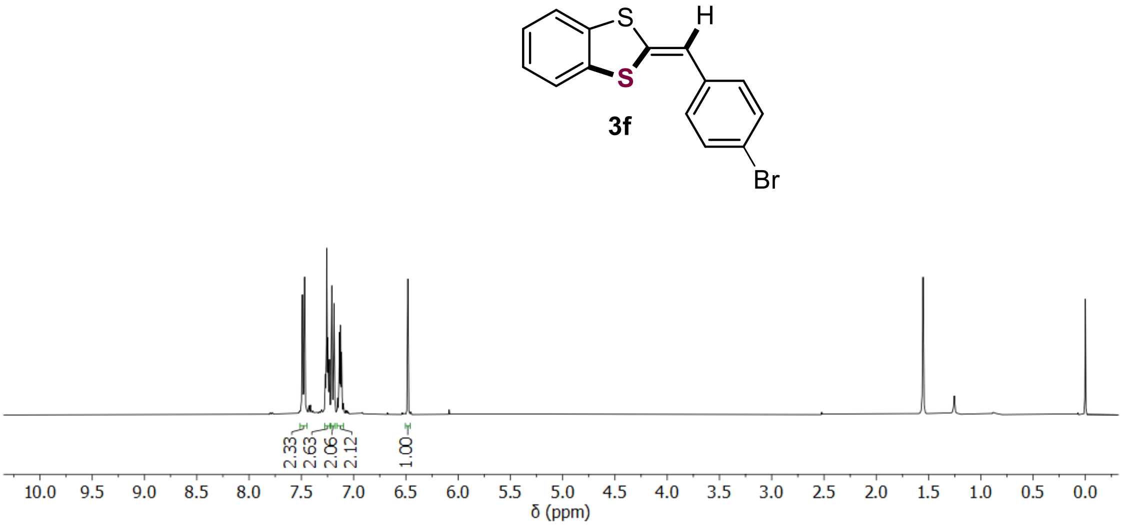

Figure S121. ^{1}H NMR (400 MHz, $CDCl_3$) spectrum of **3f**.

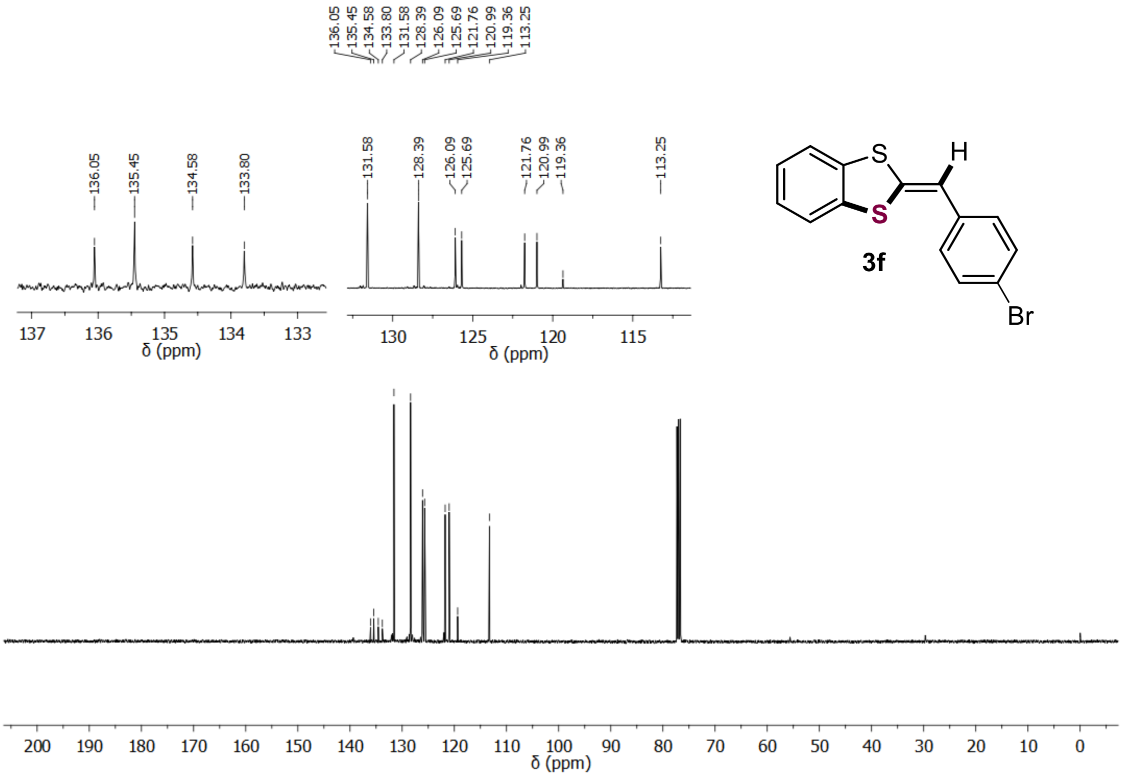

Figure S122. $^{13}C\{^{1}H\}$ NMR (100 MHz, $CDCl_3$) spectrum of **3f**.

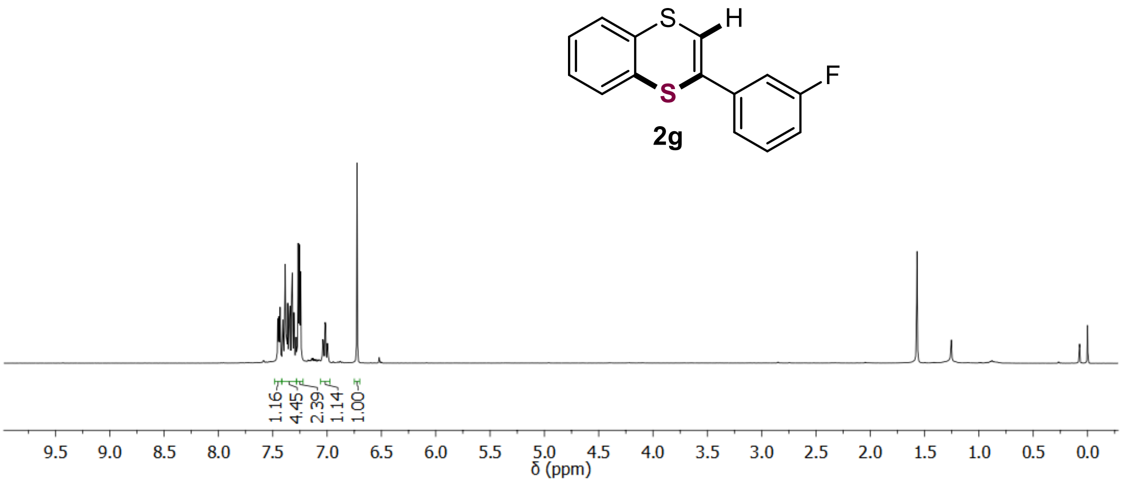

Figure S123. ^{1}H NMR (400 MHz, $CDCl_3$) spectrum of **2g**.

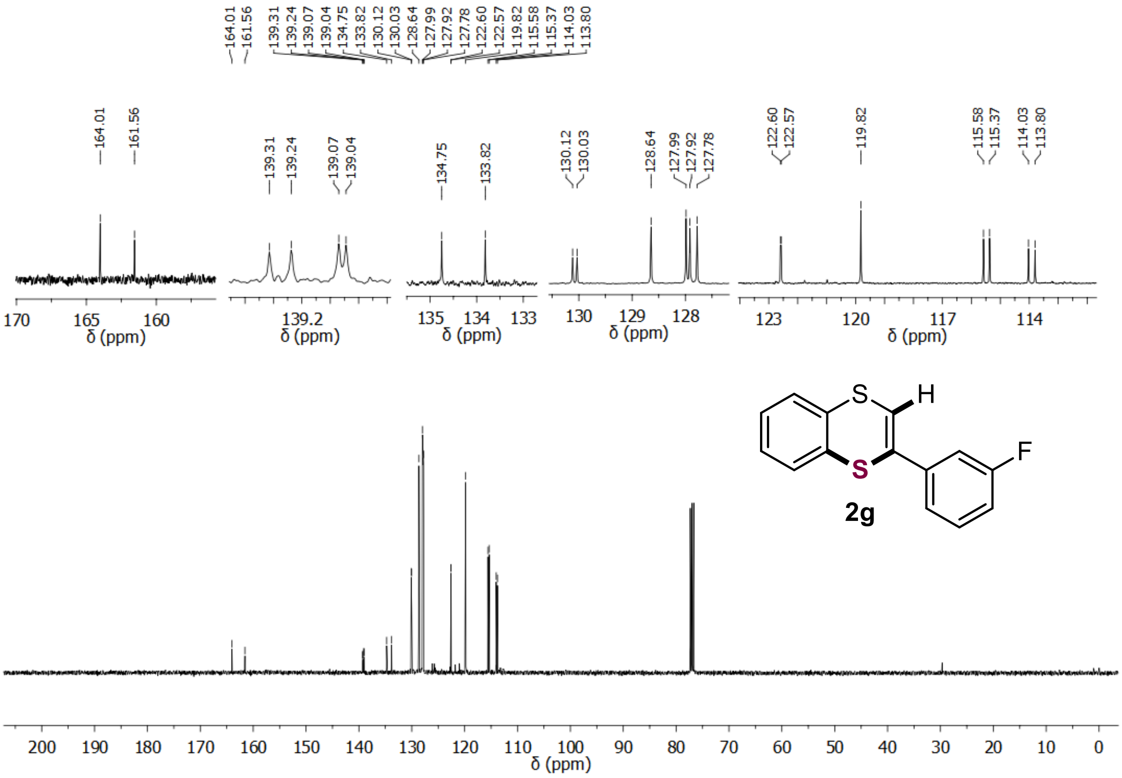

Figure S124. $^{13}C\{^{1}H\}$ NMR (100 MHz, $CDCl_3$) spectrum of **2g**.

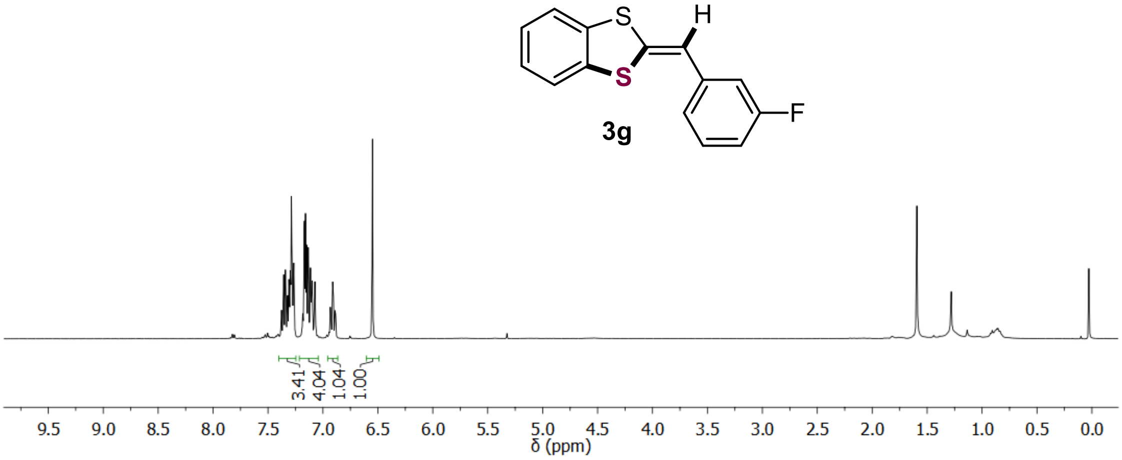

Figure S125. ^{1}H NMR (400 MHz, $CDCl_3$) spectrum of **3g.**

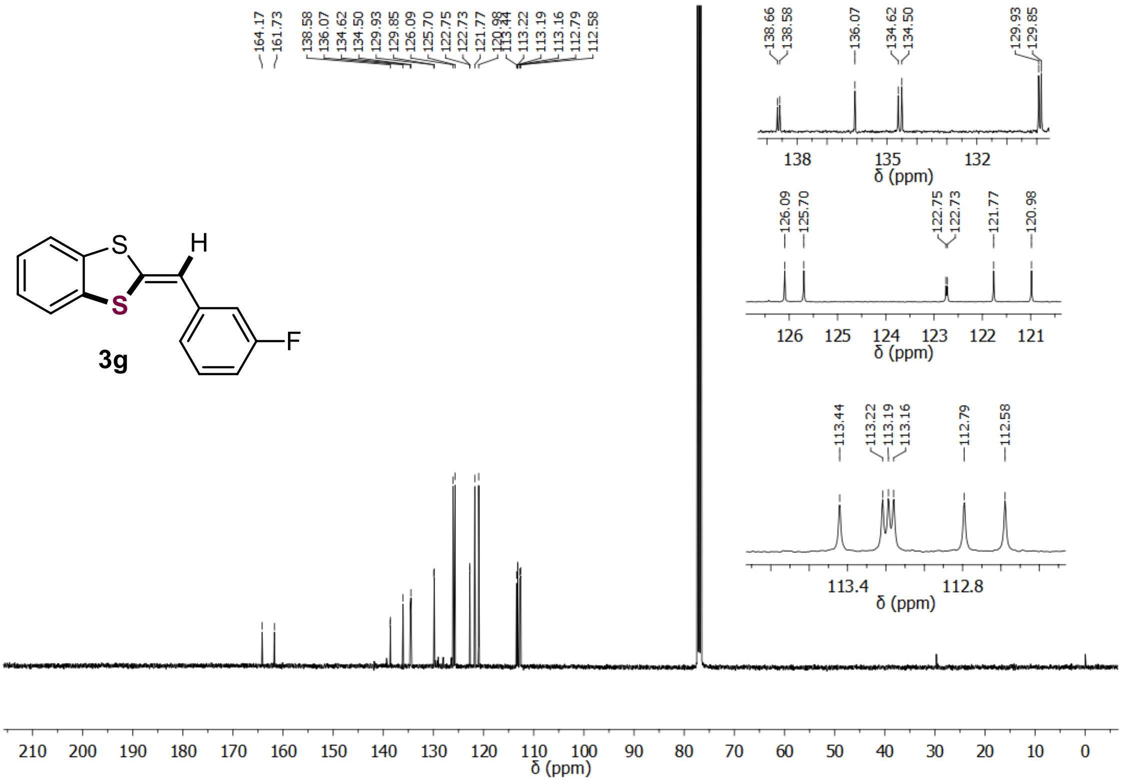

Figure S1267. ^{13}C{^{1}H} NMR (100 MHz, $CDCl_3$) spectrum of **3g.**

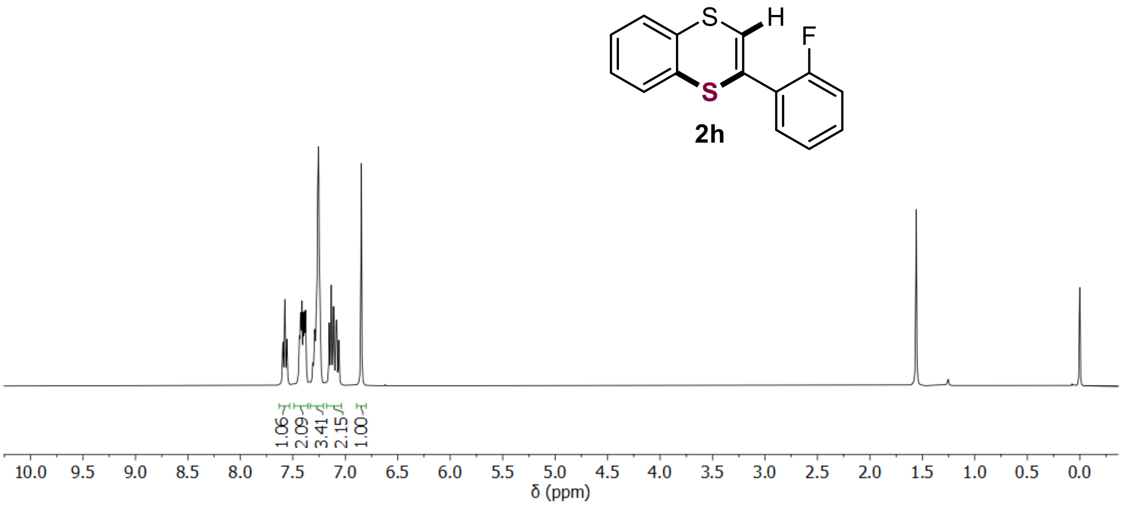

Figure S127. ^{1}H NMR (400 MHz, $CDCl_3$) spectrum of **2h**.

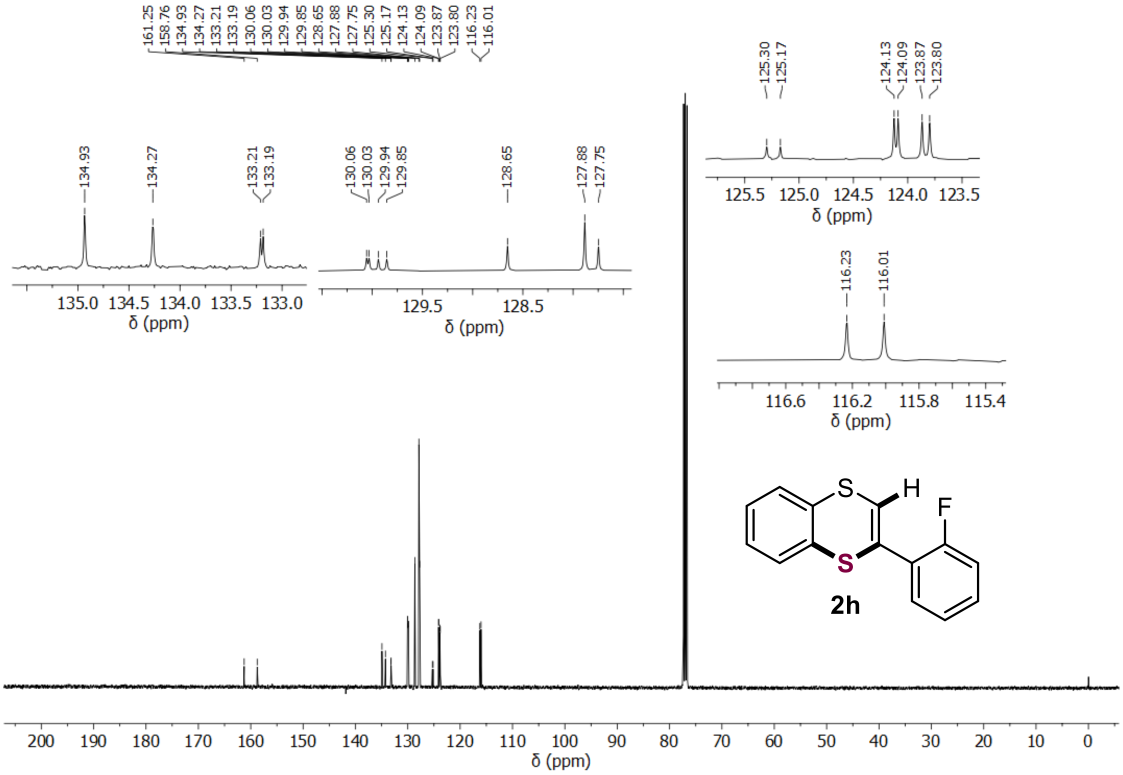

Figure S128. $^{13}C\{^{1}H\}$ NMR (100 MHz, $CDCl_3$) spectrum of **2h.**

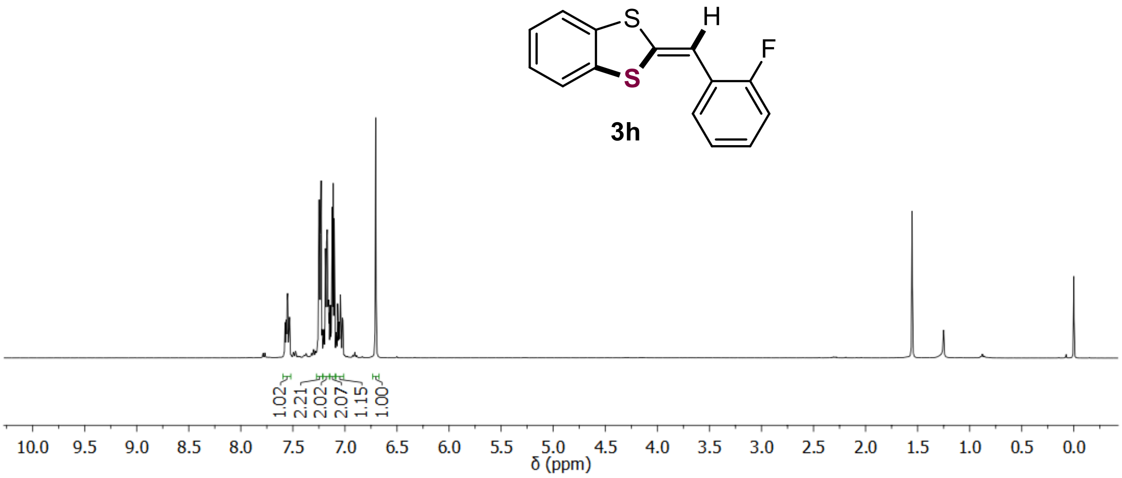

Figure S129. ^{1}H NMR (400 MHz, $CDCl_3$) spectrum of **3h**.

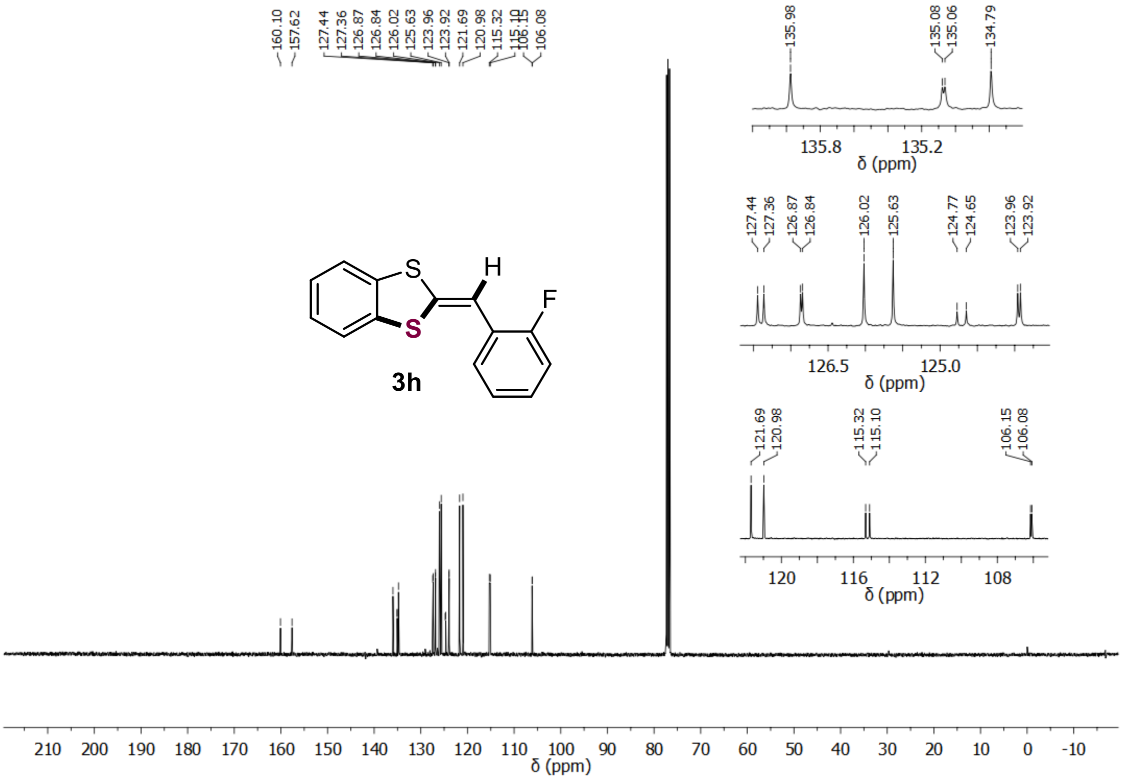

Figure S130. $^{13}C\{^{1}H\}$ NMR (100 MHz, $CDCl_3$) spectrum of **3h**.

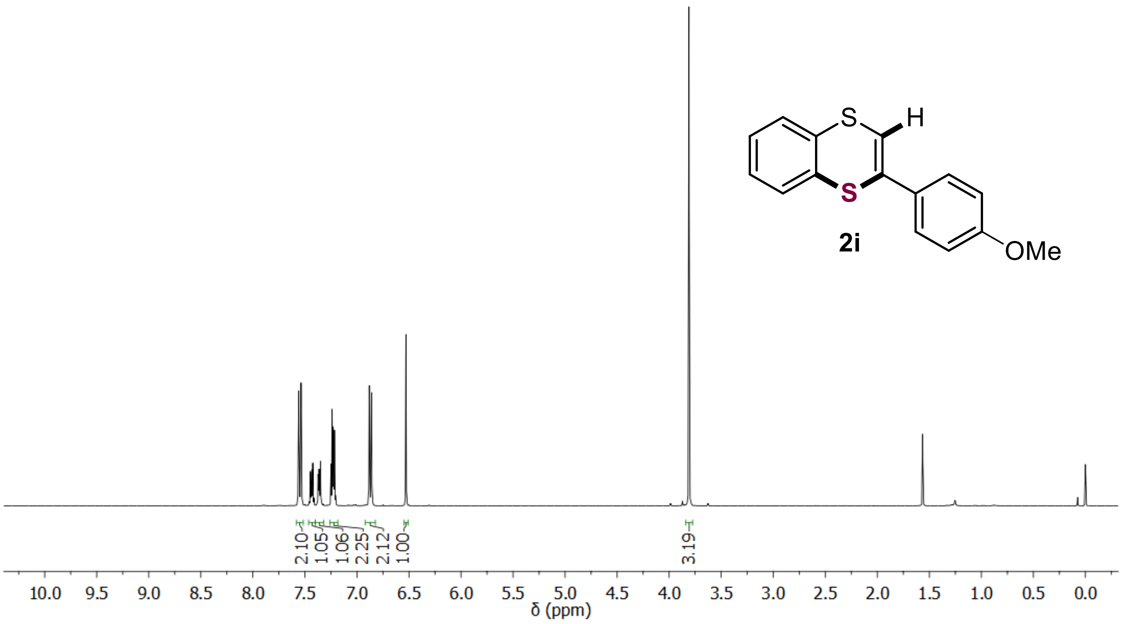

Figure S131. ^{1}H NMR (400 MHz, $CDCl_3$) spectrum of **2i**.

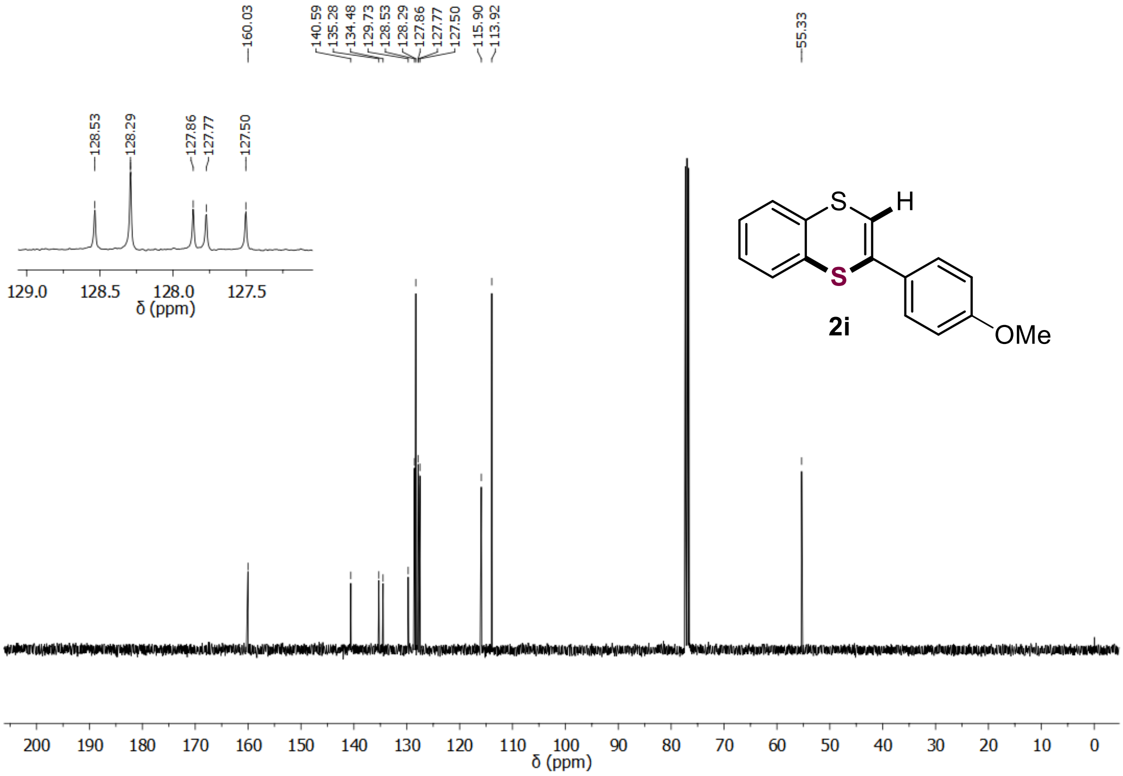

Figure S132. ^{13}C{^{1}H} NMR (100 MHz, $CDCl_3$) spectrum of **2i**.

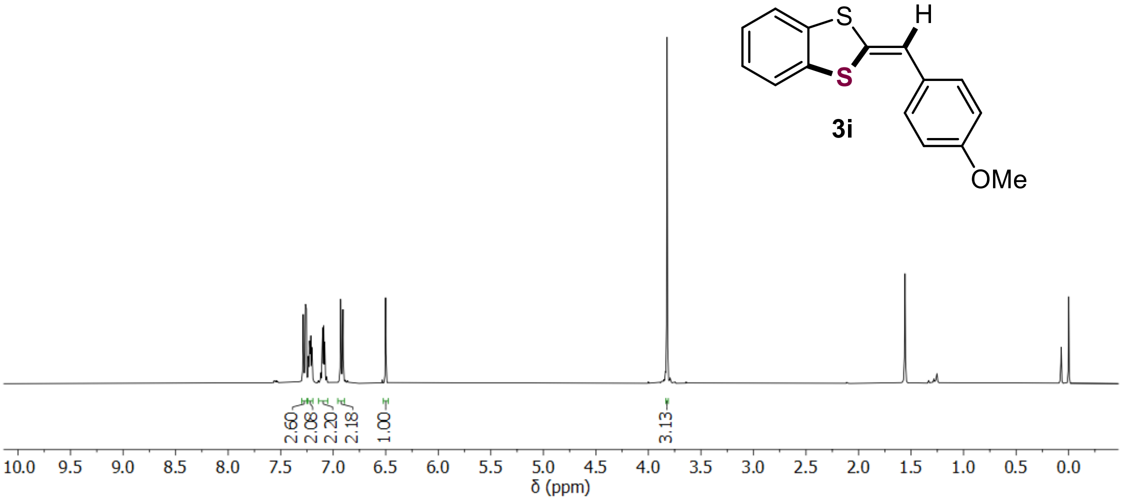

Figure S133. ¹H NMR (400 MHz, $CDCl_3$) spectrum of **3i**.

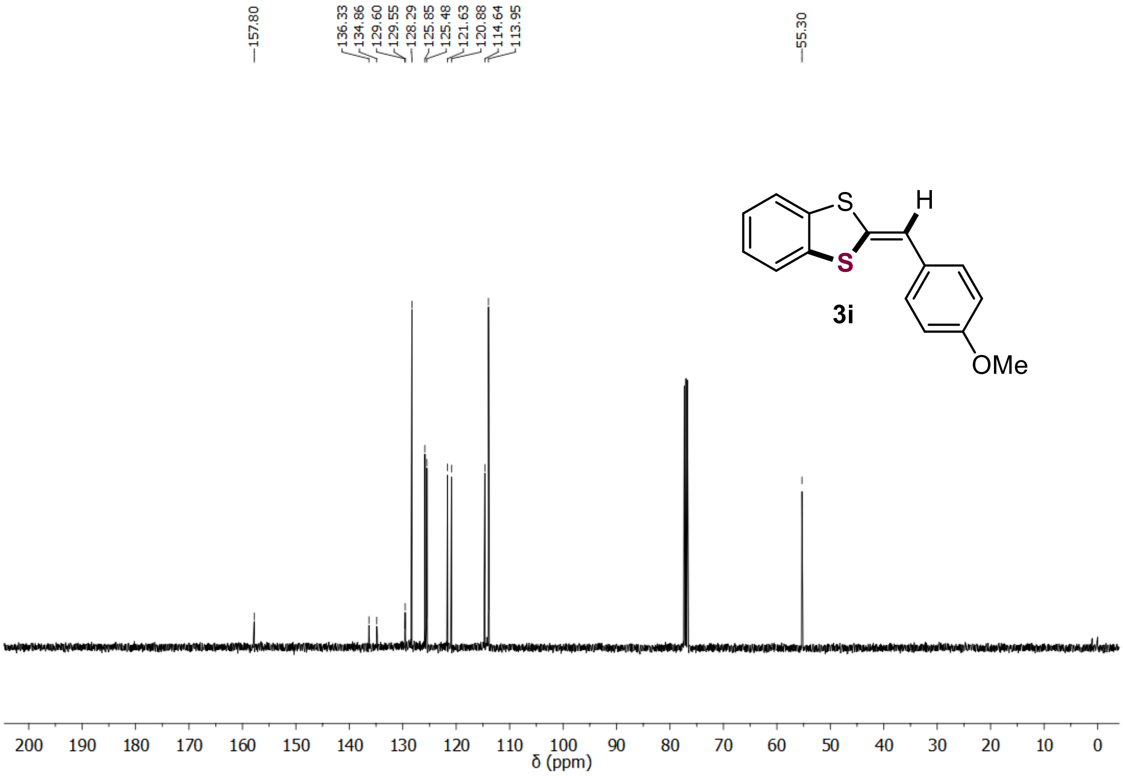

Figure S134. $^{13}C\{^{1}H\}$ NMR (100 MHz, $CDCl_3$) spectrum of **3i**.

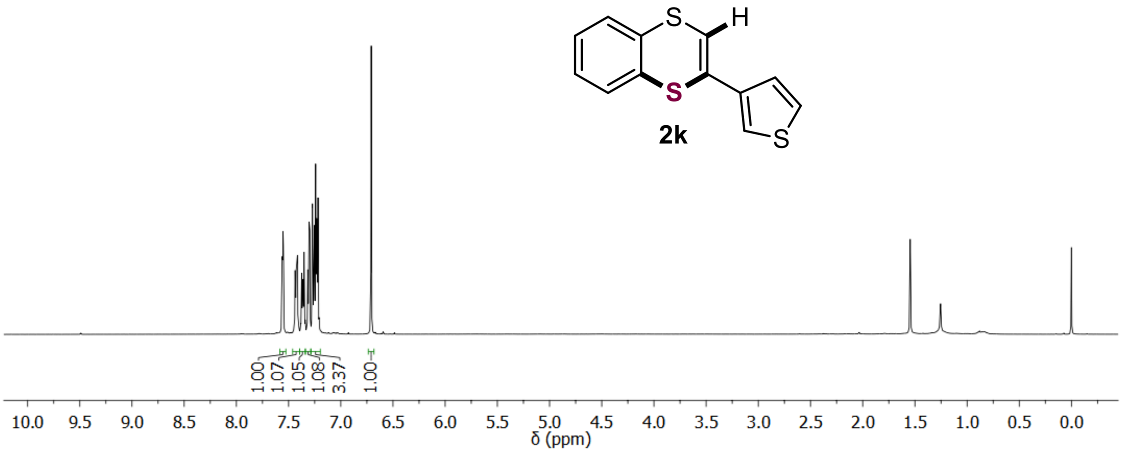

Figure S135. ^{1}H NMR (400 MHz, $CDCl_3$) spectrum of **2k**.

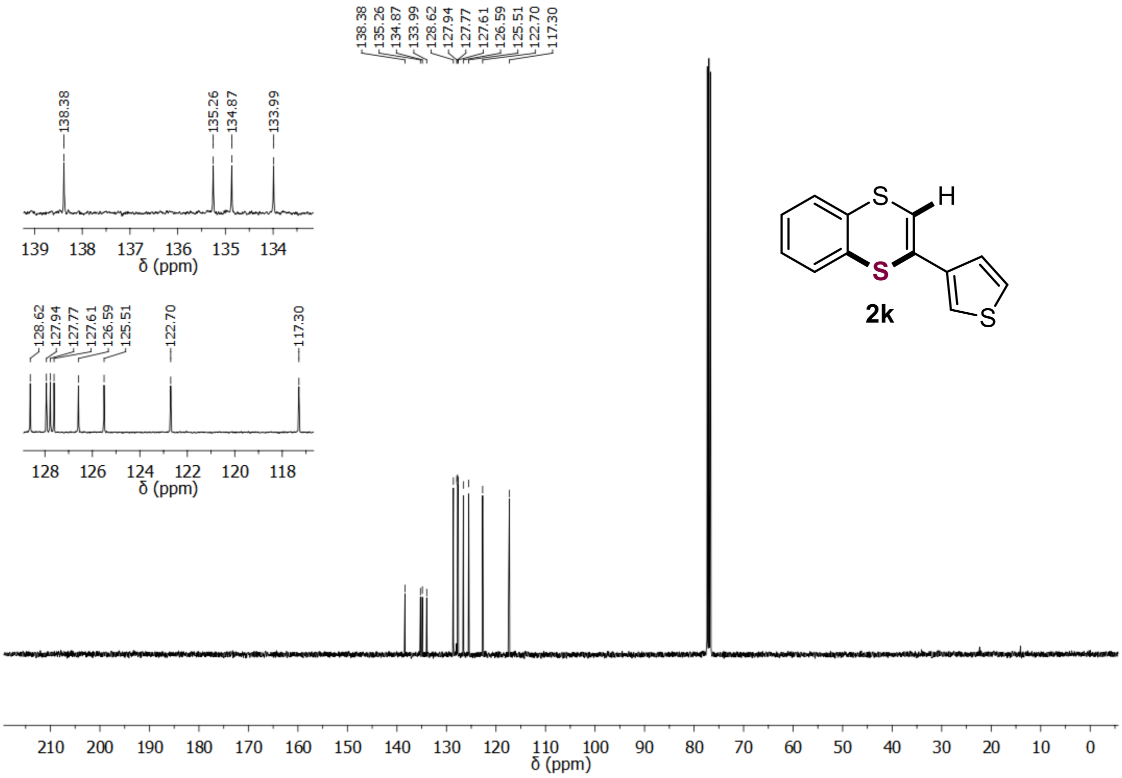

Figure S136. $^{13}C\{^{1}H\}$ NMR (100 MHz, $CDCl_3$) spectrum of **2k**.

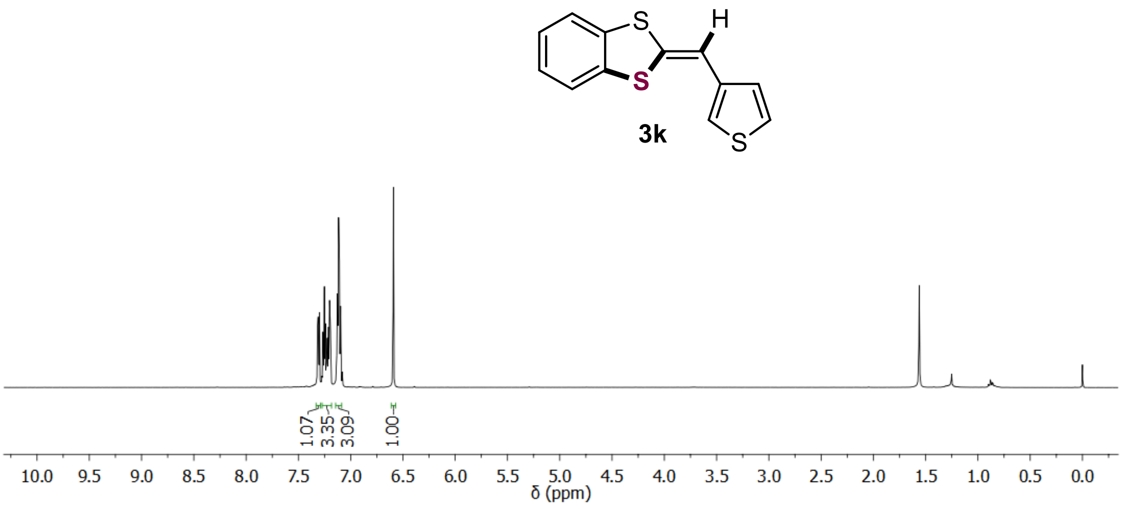

Figure S137. ^{1}H NMR (400 MHz, $CDCl_3$) spectrum of **3k**.

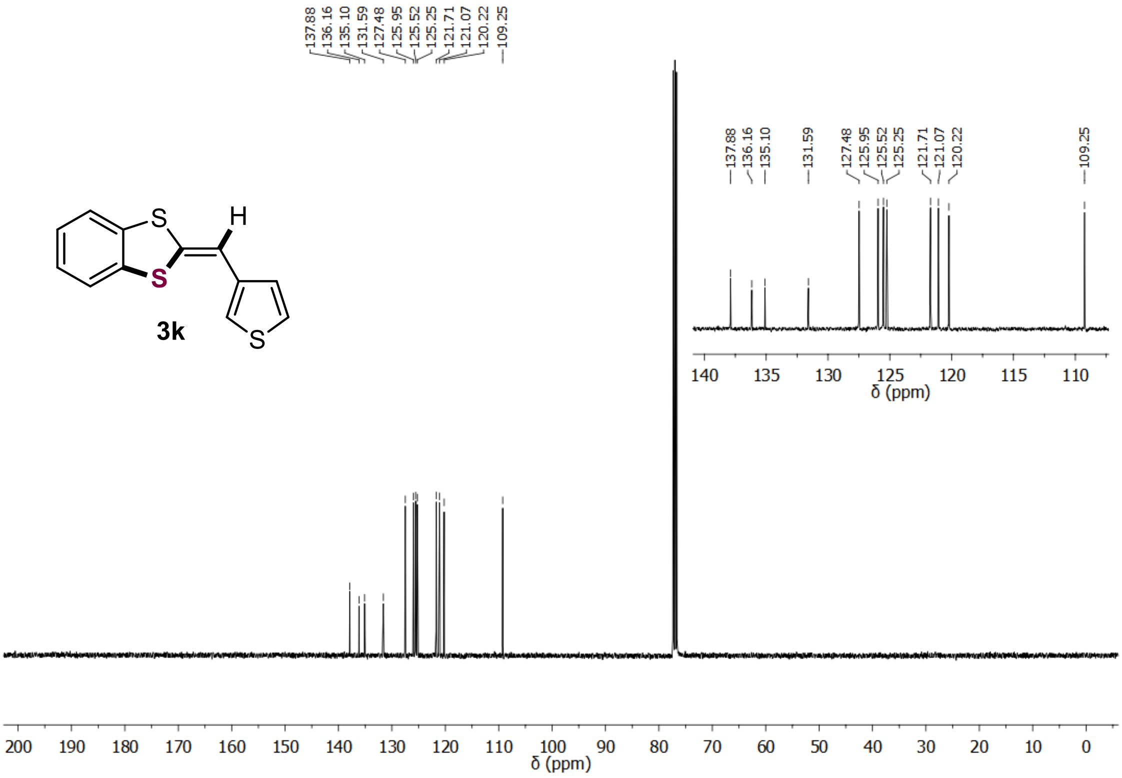

Figure S138. $^{13}C\{^{1}H\}$ NMR (100 MHz, $CDCl_3$) spectrum of **3k**.

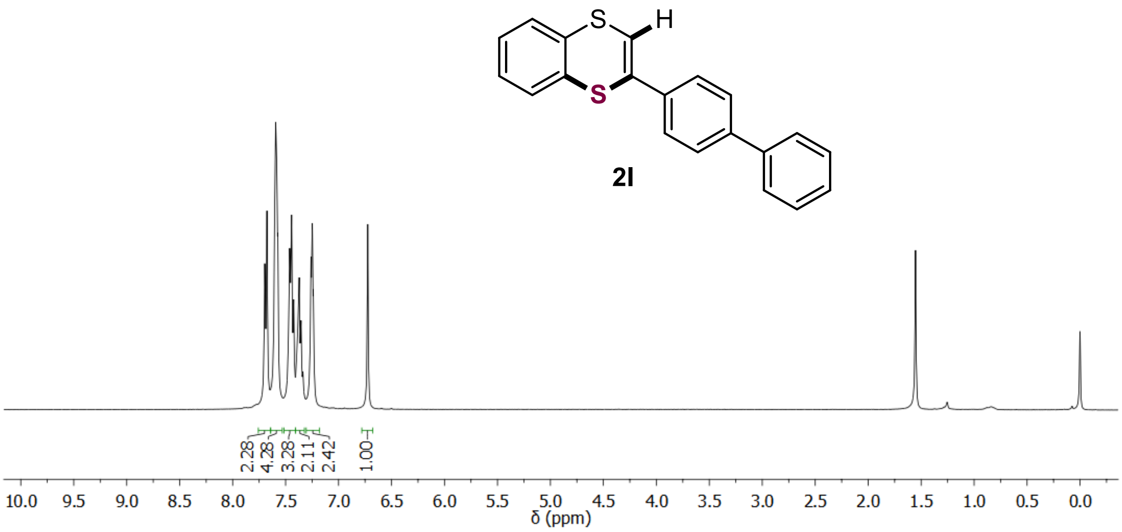

Figure S139. ^{1}H NMR (400 MHz, $CDCl_3$) spectrum of **2l**.

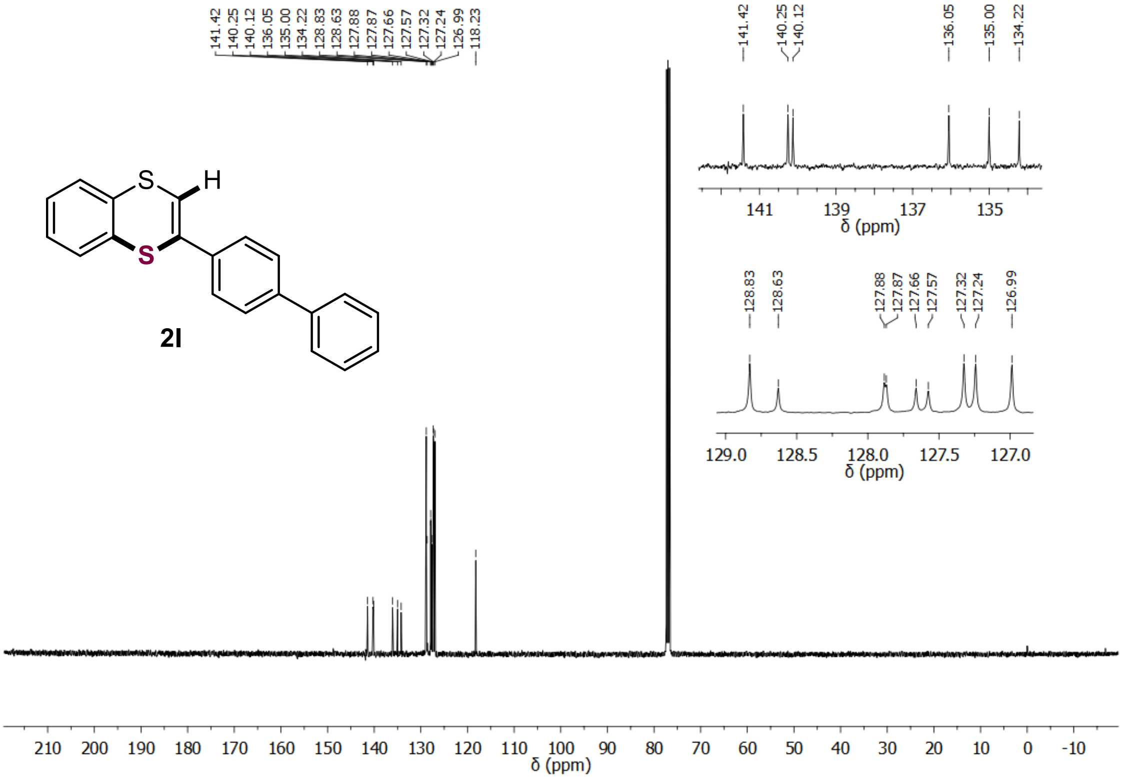

Figure S140. $^{13}C\{^{1}H\}$ NMR (100 MHz, $CDCl_3$) spectrum of **2l**.

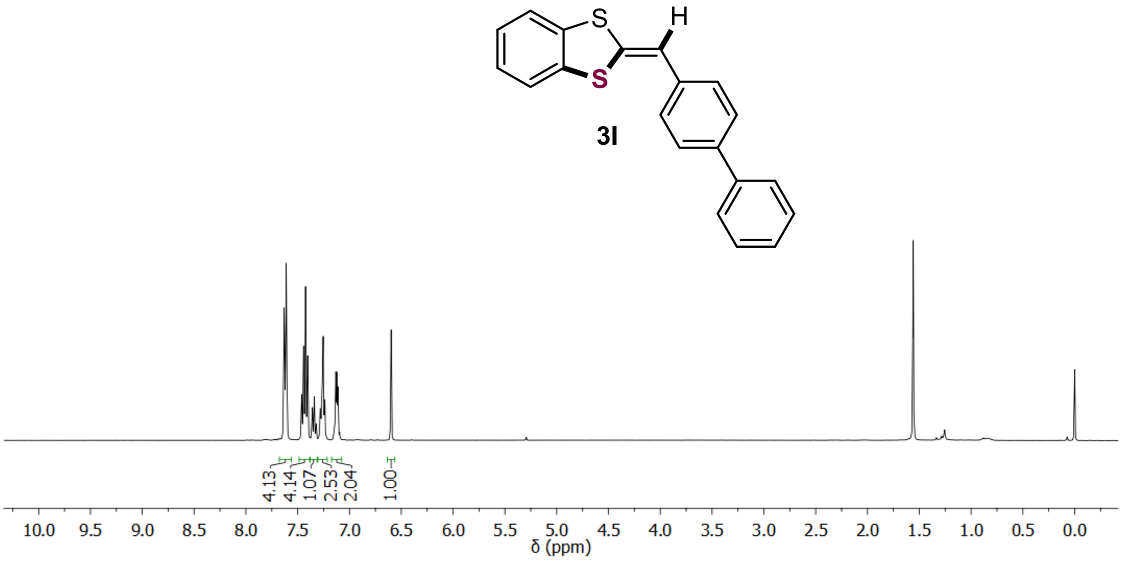

Figure S141. ^{1}H NMR (400 MHz, $CDCl_3$) spectrum of **3l**.

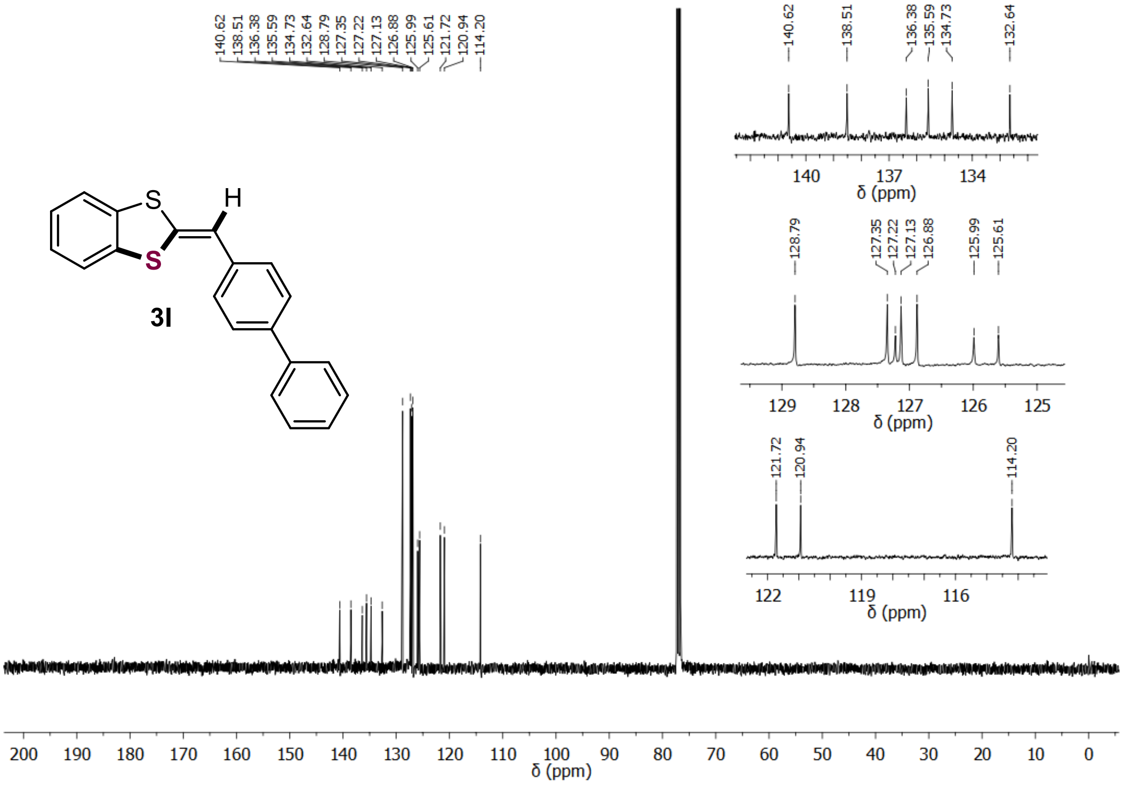

Figure S142. $^{13}C\{^{1}H\}$ NMR (100 MHz, $CDCl_3$) spectrum of **3l**.

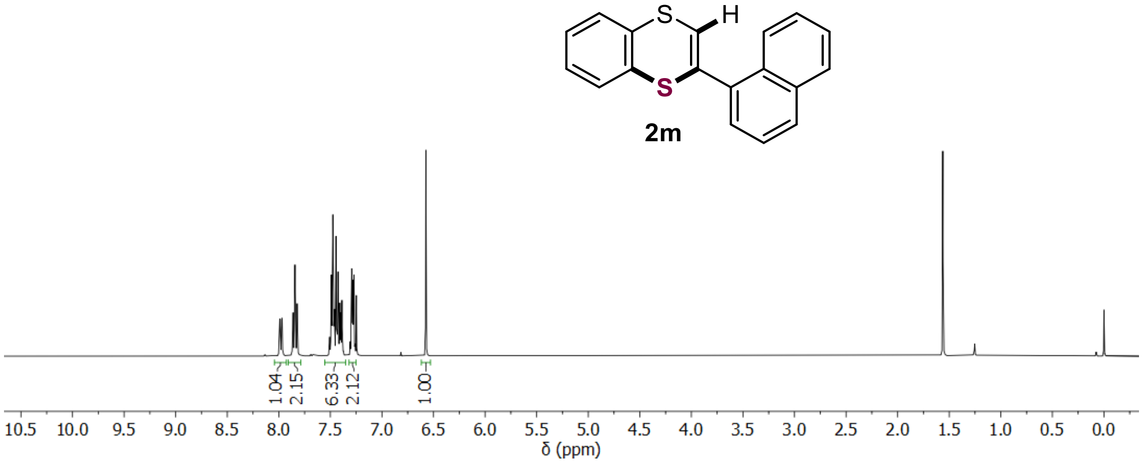

Figure S143. ^{1}H NMR (400 MHz, $CDCl_3$) spectrum of **2m**

.

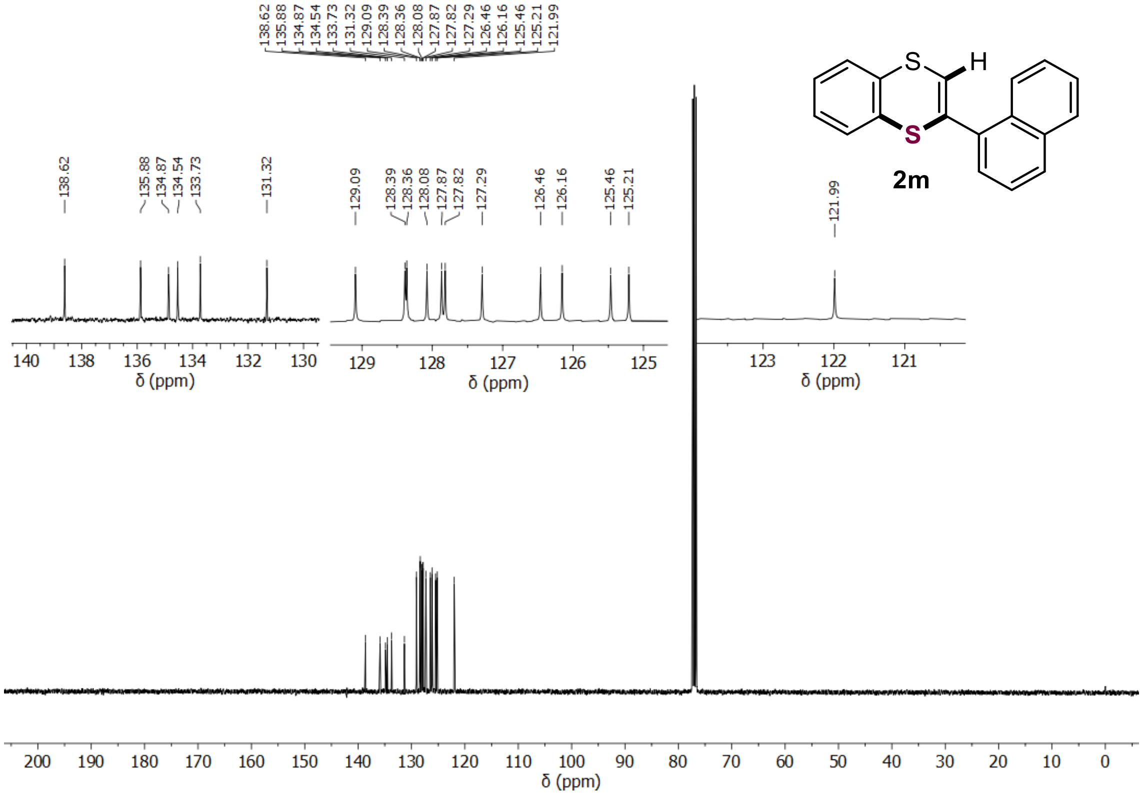

Figure S144. ^{13}C{^{1}H} NMR (100 MHz, $CDCl_3$) spectrum of **2m**.

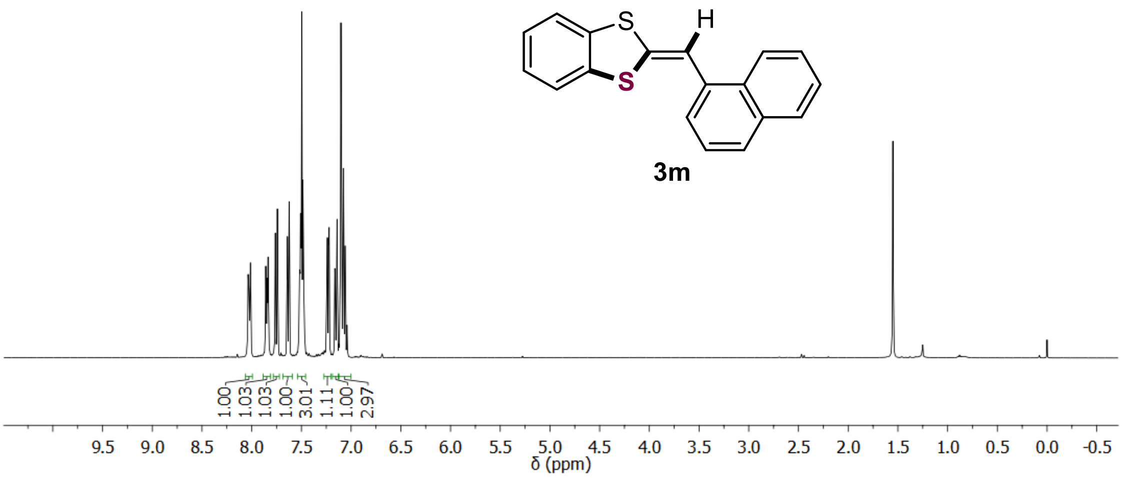

Figure S145. ^{1}H NMR (400 MHz, $CDCl_3$) spectrum of **3m**.

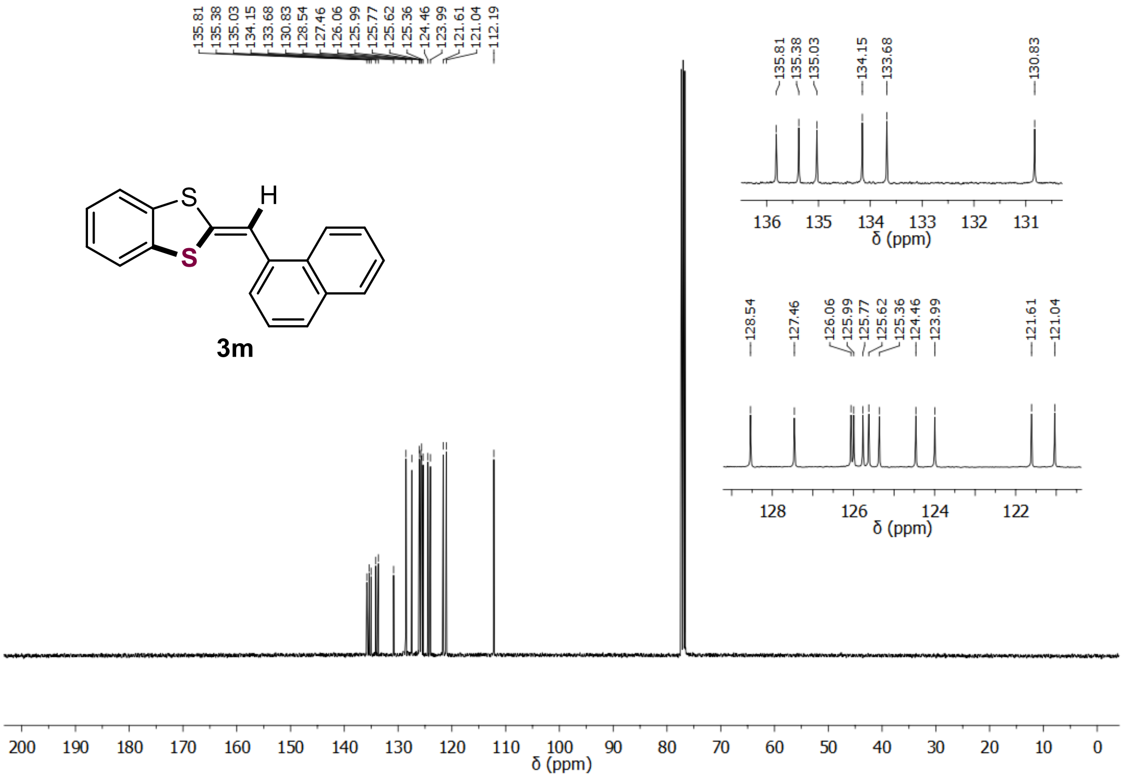

Figure S146. ^{13}C{^{1}H} NMR (100 MHz, $CDCl_3$) spectrum of **3m**.

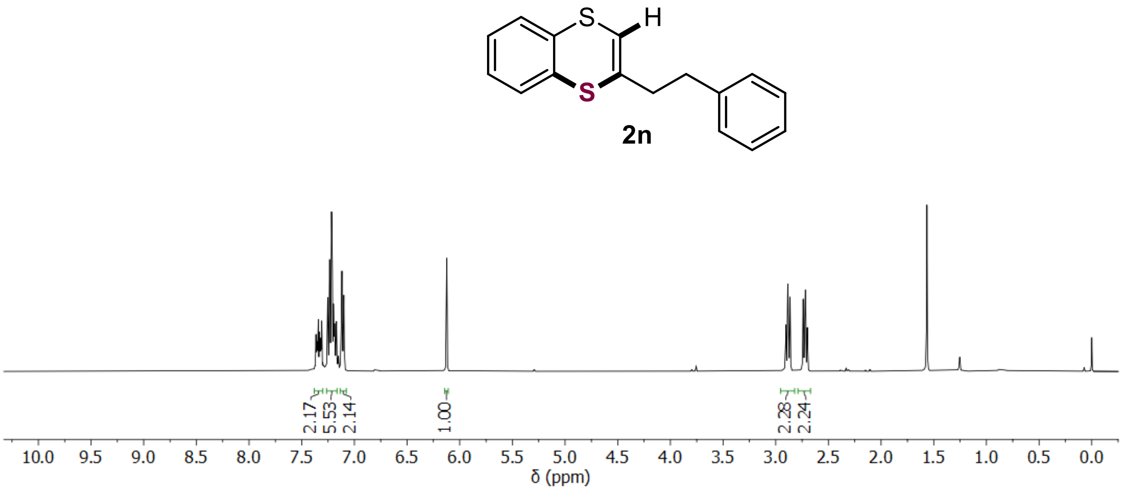

Figure S147. ^{1}H NMR (400 MHz, $CDCl_3$) spectrum of **2n**.

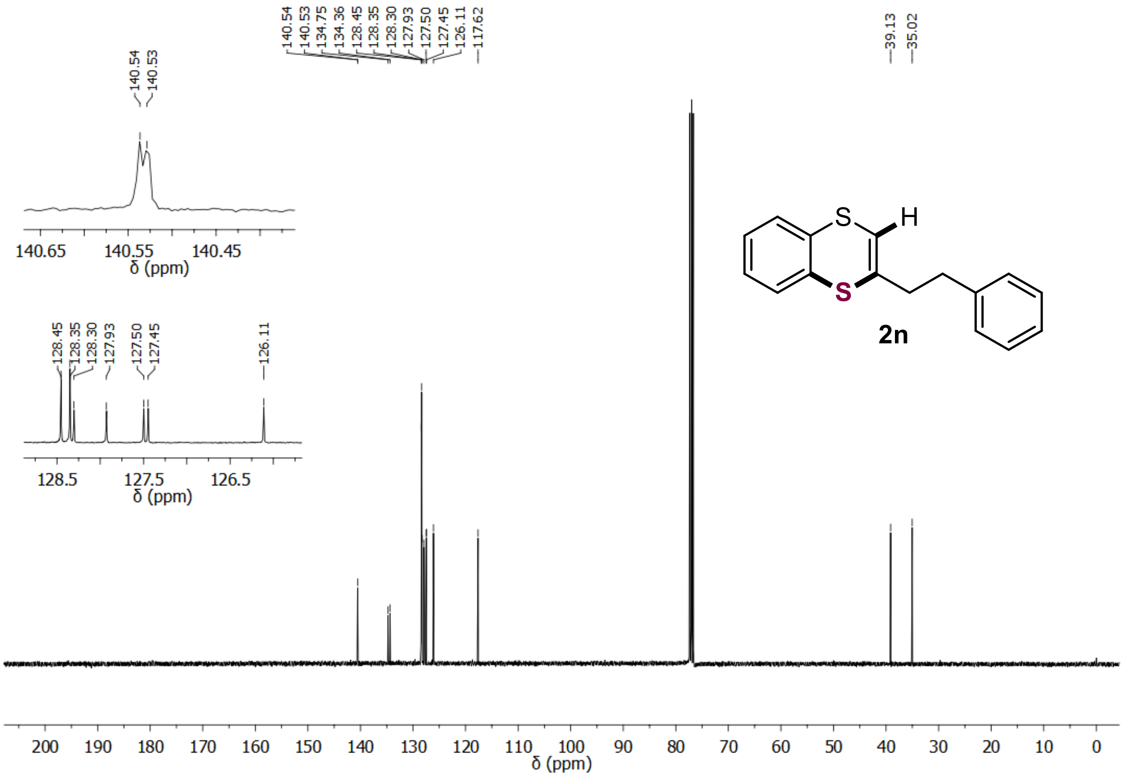

Figure S148. $^{13}C\{^{1}H\}$ NMR (100 MHz, $CDCl_3$) spectrum of **2n**.

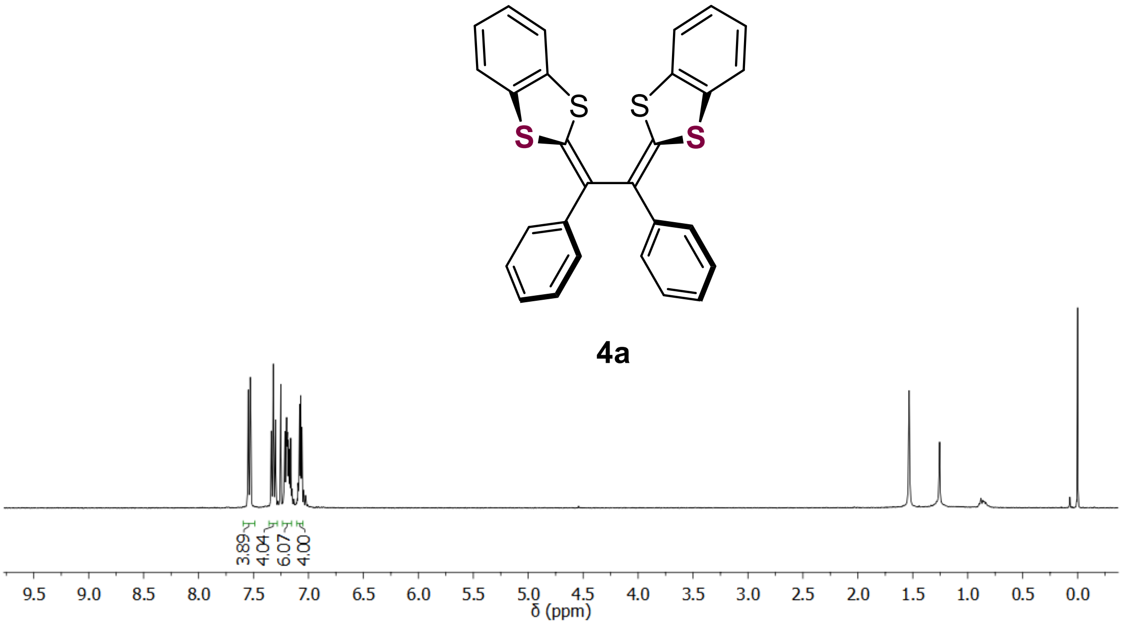

Figure S149. ^{1}H NMR (400 MHz, $CDCl_3$) spectrum of **4a.**

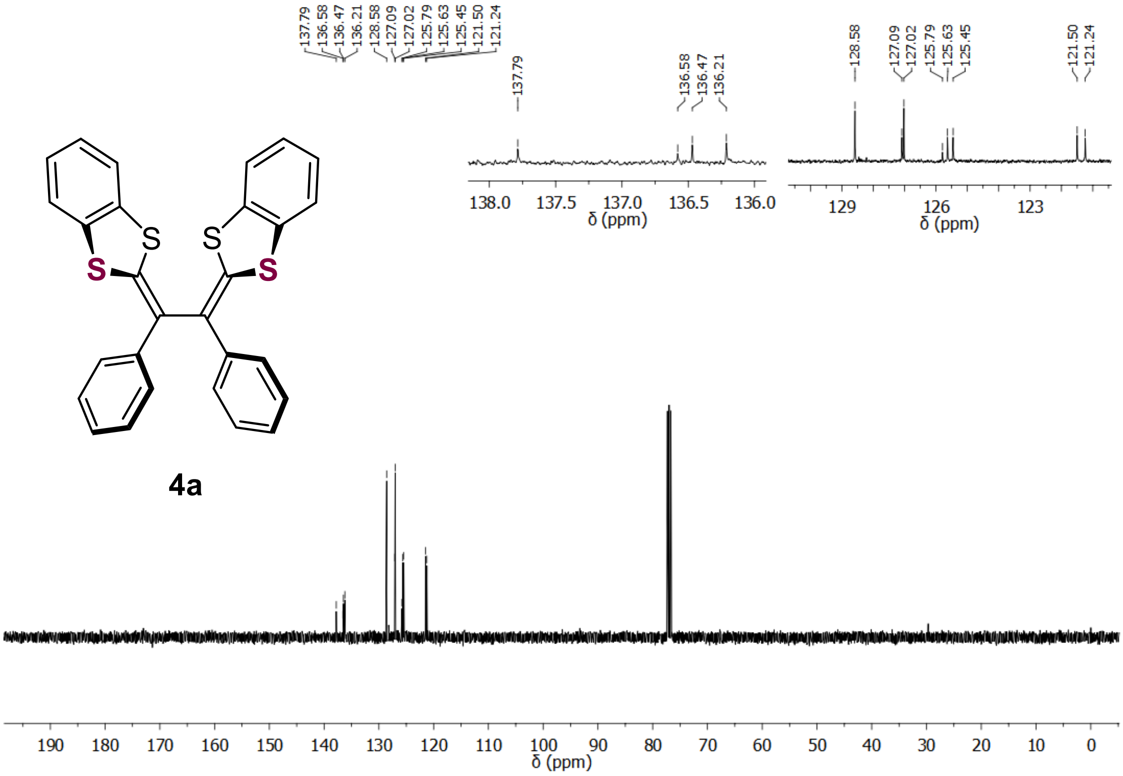

Figure S150. $^{13}C\{^{1}H\}$ NMR (100 MHz, $CDCl_3$) spectrum of **4a**.

11. References

[1] Reddy, M. B.; Anandhan, R. Visible light initiated amino group ortho-directed copper(i)-catalysed aerobic oxidative C(sp)–S coupling reaction: synthesis of substituted 2-phenylbenzothiazoles via thia-Wolff rearrangement. *Chem. Commun.* **2020**, *56*, 3781–3784.

[2] Ye, R.; Ruan, H.; Xu, H.; Li, Z.; Meng, L.-G.; Wang, L. Amino-assisted synthesis of alkynylthioethers via a visible-light-induced C(sp)-SII coupling between bromoalkynes and 2,2'-diaminodiaryldisulfides. *Org. Chem. Front.* **2021**, *8*, 5345–5351.

[3] Shen, J.; Yuan, D.; Qiao, Y.; Shen, X.; Zhang, Z.; Zhong, Y.; Yi, Y.; Zhu, X. Diaceno[a,e]pentalenes from Homoannulations of o-Alkynylaryliodides Utilizing a Unique $Pd(OAc)_2$/n-Bu_4NOAc Catalytic Combination. *Org. Lett.* **2014**, *16*, 4924–4927.

[4] Kobayashi, K.; Koyama, E.; Goto, M.; Noda, C.; Furukawa, N. Dealkylation of a 1,2-bis(benzylthio)benzene derivative: generation of benzodithiete or its equivalent via a dithia dication. *Chem. Commun.* **2000**, 1667–1668.

[5] Bellesia, F.; Boni, M.; Ghelfi, F.; Pagnoni, U. M. Synthesis of 2-(1-chloroalkyl)-1,3-benzodithioles and 2-alkylidene-1,3-benzodithioles. *J. Heterocycl. Chem.* **1994**, *31*, 1721–1723.

[6] Y. Fujishita, M. Uchida, T. Nakano, K. Furukawa, *U.S. Patent* n. 6,617,053, **2003.**

[7] Liu, B.; Alegre-Requena, J. V.; Paton, R. S.; Miyake, G. M. Unconventional reactivity of ethynylbenziodoxolone reagents and thiols: scope and mechanism. *Chem. Eur. J.* **2020**, *26*, 2386–2394.

[8] Wang, Y.; Zhao, Y. Carboxylated dithiafulvenes and tetrathiafulvalene vinylogues: synthesis, electronic properties, and complexation with zinc ions. *Beilstein J. Org. Chem.* **2015**, *11*, 957–965.

[9] Häcker, J.; Nguyen, D. H.; Rommel, T.; Zhao-Karger, Z.; Wagner, N.; Friedrich, K. A. Operando UV/vis Spectroscopy Providing Insights into the Sulfur and Polysulfide Dissolution in Magnesium-Sulfur Batteries. *ACS Energy Lett.* **2022**, *7*, 1–9.

[10] (a) Tobishima, S. I.; Yamamoto, H.; Matsuda, M. Study on the Reduction Species of Sulfur by Alkali Metals in Nonaqueous Solvents. *Electrochim. Acta*

1997, *42*, 1019–1029. (b) Steudal, R.; Chivers, T. The role of polysulfide dianions and radical anions in the chemical, physical and biological sciences, including sulfur-based batteries. *Chem. Soc. Rev.* **2019**, *48*, 3279–3319.

[11] Chivers, T.; Elder, P. J. W. Ubiquitous trisulfur radical anion: fundamentals and applications in materials science, electrochemistry, analytical chemistry and geochemistry. *Chem. Soc. Rev.* **2013**, *42*, 5996–6005.

[12] Hojo, M.; Sawyer, D. Hydroxide-induced reduction of elemental sulfur (S8) to trisulfur anion radical (S3.bul.-). *Inorg. Chem.* **1989**, *28*, 1201–1202.

[13] Zou, Q.; Lu, Y.-C. Solvent-Dictated Lithium–Sulfur Redox Reactions: An Operando UV–Vis Spectroscopic Study. *J. Phys. Chem. Lett.* **2016**, *7*, 1518–1525.

[14] Mai, Y.-Z.; Xie, Y.-Z.; Zheng, M.-H.; Zhou, X.; Jin, J.-Y. Facile Synthesis of Pyronin-9-Thione via a Trisulfur Radical Anion Mechanism. *New J. Chem.* **2021**, *45*, 19–22.

[15] Sheldrick, G. M. SHELXT – Integrated space-group and crystal-structure determination. *Acta Crystallogr. A* **2015**, *71*, 3–8.

[16] Dolomanov, O. V.; Bourhis, L. J.; Gildea, R. J.; Howard, J. A. K.; Puschmann, H. OLEX2: a complete structure solution, refinement and analysis program. *J. Appl. Crystallogr.* **2009**, *42*, 339–341.

[17] Sheldrick, G. M. Crystal structure refinement with SHELXL. *Acta Crystallogr. C* **2015**, *71*, 3–8.
